# Nanopore sequencing and de novo assembly of a misidentified Camelpox vaccine reveals putative epigenetic modifications and alternate protein signal peptides

**DOI:** 10.1038/s41598-021-97158-x

**Published:** 2021-09-07

**Authors:** Zack Saud, Matthew D. Hitchings, Tariq M. Butt

**Affiliations:** 1 Department of Biosciences, College of Science, Swansea University, Singleton Park, Swansea, SA2 8PP Wales UK; 2 Swansea University Medical School, Swansea University, Singleton Park, Swansea, Sa2 8PP Wales UK

**Keywords:** Epigenetics, Epigenomics, Genome, Microbial genetics, Sequencing

## Abstract

DNA viruses can exploit host cellular epigenetic processes to their advantage; however, the epigenome status of most DNA viruses remains undetermined. Third generation sequencing technologies allow for the identification of modified nucleotides from sequencing experiments without specialized sample preparation, permitting the detection of non-canonical epigenetic modifications that may distinguish viral nucleic acid from that of their host, thus identifying attractive targets for advanced therapeutics and diagnostics. We present a novel nanopore de novo assembly pipeline used to assemble a misidentified Camelpox vaccine. Two confirmed deletions of this vaccine strain in comparison to the closely related Vaccinia virus strain modified vaccinia Ankara make it one of the smallest non-vector derived orthopoxvirus genomes to be reported. Annotation of the assembly revealed a previously unreported signal peptide at the start of protein A38 and several predicted signal peptides that were found to differ from those previously described. Putative epigenetic modifications around various motifs have been identified and the assembly confirmed previous work showing the vaccine genome to most closely resemble that of Vaccinia virus strain Modified Vaccinia Ankara. The pipeline may be used for other DNA viruses, increasing the understanding of DNA virus evolution, virulence, host preference, and epigenomics.

## Introduction

DNA viruses include those which have DNA genomes and replicate using DNA-dependent DNA polymerase. They are grouped into two classes, comprising single stranded DNA viruses and double stranded DNA viruses. The latter group contains the infamous Variola Virus (VARV), the causative agent of smallpox, which belongs to the family *Poxviradae*, subfamily *Chordopoxvirinae* and genus *Orthopoxvirus*. There are currently 12 accepted species within the genus, the other notable members including; Vaccinia virus (VACV)—the prototype *Orthopoxvirus* used as a vaccine to eradicate human smallpox and which has no known natural host^1^, Cowpox virus (CPXV)—administered successfully by Edward Jenner as the first documented successful vaccine^2^, Monkeypox virus (MPXV)—a zoonotic virus endemic to the African subcontinent^3^, and Camelpox (CMLV)—the most genetically similar extant species to VARV^4^.

Poxviruses have linear, double-stranded DNA genomes that vary from 130 to 230 kbp^5^. The telomere ends of the genome form covalently closed hairpin structures at the termini^6^. The hairpin is at the end of a long, inverted terminal repetition (ITR) containing sets of short, tandemly repeated sequences^5^. For orthopoxviruses, the size of the ITRs range from approximately 200–500 base pairs for variola viruses, to almost 12,000 base pairs for several vaccinia virus strains^7^. Large ITR regions can pose problems for first generation Sanger sequencing^8^ and second-generation Illumina sequencing^9^, which are capable of producing sequence read lengths of up to around 1000 bp and 300 bp (or around 500 bp linked pair-end) respectively. Such tracts of repetitive sequences in a genome can be resolved by third-generation long read sequencing technologies^10–12^, which are capable of producing read lengths in excess of 100,000 bp.

The central portions of most poxvirus genomes are highly conserved, and contain essential genes involved in key functions such as transcription, DNA replication and virion assembly^13^. In contrast, genes that cluster at the ends of the genome are usually species or host specific, and encode virulence factors that modulate the host immune system^13,14^. Various proteins encoded by the genome have been shown to interact with DNA or precursor nucleotides^5^. The K7 protein has been shown to promote histone methylation associated with heterochromatin formation^15^. Furthermore, vaccinia virus (VACV) C4^16^, C6^17^, C16^18^, B14^19^, E3^20^, F16^21^, and N2^22^ gene products can be detected in the host nucleus, thus implicating them in some form of transcriptional regulation. To our knowledge, no research has been aimed towards assessing whether these proteins epigenetically modify the viral DNA. Furthermore, despite what is known of the capability of DNA viruses to exploit host cellular epigenetic processes to their advantage during infection^23,24^, the epigenome status of most DNA viruses remains unknown.

Third generation sequencing technologies have advanced epigenomic research by providing platforms that allow for the identification of modified nucleotides from sequencing experiments without the need for specialized secondary sample preparation protocols^25–27^. Such a direct approach for interrogating an epigenome is particularly beneficial for viral epigenetic research, as samples often contain high amounts of contaminating host DNA, which can complicate specialized DNA methylation probing techniques such as bisulfite sequencing^28^ and antibody based approached^29^. Furthermore, motifs with non-canonical epigenetic modifications can be identified by distinguishing a deviation of the raw signal from that of a standard model at a given nucleotide sequence^26,30^. Such non-canonical epigenetic modifications would distinguish viral DNA from that of host DNA, making them attractive targets for advanced therapeutics and diagnostics^31^. A drawback of Nanopore sequencing technology is that reads generally suffer from a comparatively high error rate (particularly in regions containing homopolymers) in comparison to other sequencing technologies, although advances in library preparation chemistry, pore technology and algorithms (basecalling, assembly and polishing) have greatly improved overall assembly error rates^32^.

In this study, we use nanopore sequencing to assemble the genome of a live attenuated CMLV strain, Ducapox, that was stated to comprise a CMLV isolate from the United Arab Emirates (CaPV298-2)^33^. The vaccine has since been found to contain two gene regions that more closely resembled that of VACV strain Modified Vaccinia Ankara (VACV-MVA)^34^. A separate study of the strain using second generation WGS found the vaccine genome matches that of VACV-MVA, with the exception of two genomic deletions (5195 and 890 bp in size), however, the authors questioned the authenticity of these genomic deletions due to both the reference-based assembly approach adopted, and the low sequencing coverage of the genome^35^. We present a sequencing and annotation pipeline for long read de novo assembly of Poxvirus genomes and identify putative epigenetic modifications within the genome. Using the latest version of signal peptide predication software, we identify a predicted protein with a previously undescribed signal peptide, and present several predicted signal peptides that were found to differ from previously described sequences. The pipeline may be used for other DNA viruses, increasing the understanding of DNA virus epigenomics.

## Results

### Sequencing statistics and de novo assembly

**Figure 1:**
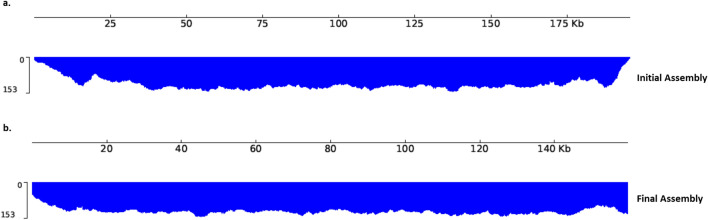
Read mapping coverage of genome assemblies for a. the initial Flye assembly and b. the final polished assembly. Read coverage was found to be more uniformly distributed in the final assembly in comparison to the initial assembly (Flye assembly using > 3000 Viral DNA Read Set), which was found to have uneven read coverage distributions at the contig ends. This is indicative of the final polished assembled containing terminal repeat sequence lengths that more closely match that of the ground truth.

A total of 405,925 base called sequences were produced from the MinION sequencing run, of which 16,059 (3.95%) remained after size filtering and removal of non-viral DNA (Table 1). Most of the non-viral DNA was found to be of simian origin, consistent with the virus having been propagated in Vero cells. The Flye assembler produced a viral contig that was 195,695 bp in length. After ITR correction and all polishing steps, the assembly was 159,696 bp in length. Read coverage was found to be more uniformly distributed in the final assembly in comparison to the initial assembly (Flye assembly using > 3000 Viral DNA Read Set), the latter of which was found to have uneven read coverage distributions at the contig ends (Fig. 1). This is indicative of the final polished assembly containing terminal repeat sequence lengths that more closely match that of ground truth. Furthermore, a large coverage of reads had mapped to the ITR at the 3′ end of the genomes, indicative of poor ITR assembly, when reads were mapped to the Ducapox short-read assembly (supplementary information 1a). The mappings highlight the short-comings of adopting reference-based alignment assemblies using short-reads, as the large coverage of mapped reads to the 3′ ITR region was also observed when the same > 3000 Viral DNA read set was mapped to VACV Acambis 3000 MVA (supplementary information 1b).

**Table 1:** Read metrics of sequencing before and after non-viral DNA removal.

Metric	Raw reads	> 3000 viral DNA read set
Number of reads	405,925	16,059
Cumulative size (bp)	828,487,274	94,298,074
Average read length (bp)	2,041	5,872
N50 (bp)	6,507	6,174
> Q12	301,538 (74.3%)	13,599 (84.7%)

### Whole genome sequence comparisons

**Figure 2:**
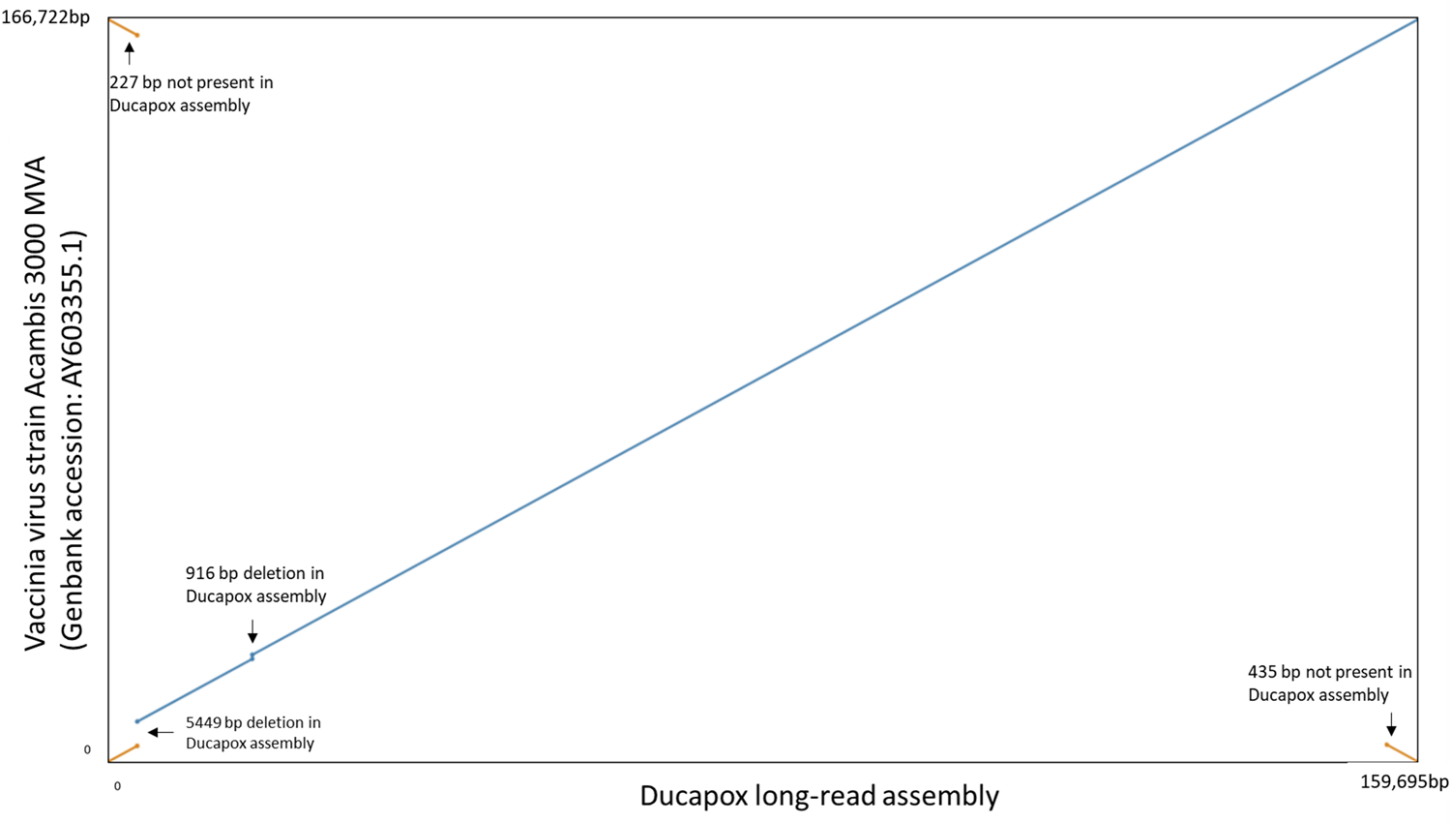
Dotplot comparison of the Ducapox long read assembly vs the closest matching viral genome, that of VACV Acambis 3000 MVA. Genomic deletions of 5449 bp and 916 bp in size are illustrated. The VACV Acambis 3000 MVA was also found to be 227 bp and 435 bp longer at its ends, with respect to the Ducapox genome.

A blast search of the final polished assembly revealed the genome to most closely match that of Vaccinia virus strain Acambis 3000 MVA (Genbank Accession: AY603355.1), with a blast percentage identity score of 99.99%. A dotplot comparison of the Ducapox long read assembly vs VACV Acambis 3000 MVA revealed genomic deletions of 5449 bp and 916 bp in size in the Ducapox genome, corresponding to VACV Acambis 3000 MVA genome positions 3735–9183, and 23,219–24,134, respectively (Fig. 2). These deletions were confirmed by visualizing the mapping of reads to the genome assembly, and confirming that unbroken reads traversed the deletion sites (supplementary information 2a and 2b). The VACV Acambis 3000 MVA was also found to be 227 bp and 435 bp longer at its ends, in comparison to the Ducapox genome. The deletions in the Ducapox genome are further contrasted by a multiple sequence alignment between the Ducapox long read genome assembly, the Ducapox short read genome assembly, and the VACV Acambis 3000 MVA genome in supplementary information 2c. Both average and median identity scores were found to be higher, and error rates lower, when the > 3000 Viral DNA read set was mapped to the Ducapox genome than when mapped to VACV Acambis 3000 MVA (Table 2). 2 proteins predicted in the initial long-read assembly were found to be a single protein in the short-read assembly, as a result of a frameshift caused by the insertion of an additional adenine residue in a homopolymer track wherein the length of the homopolymer was 6 adenine residues in the short read assembly, and 7 adenine resides in the long-read assembly causing a truncation of the first protein (supplementary information 2d). Remarkably, in the long-read protein set, a second open reading frame within the first protein that frameshifted resulted in the formation of a second protein that was in-frame with the end portion of the truncated protein (supplementary information 2d).

**Table 2:** Read alignment identity and error metrics of exclusive viral read set to the long-read assembly and to VACV Acambis 3000 MVA.

Read-mapping metric	Ducapox long-read assembly	VACV Acambis 3000 MVA
Average percent identity	95.3	85.9
Median percent identity	96.7	84.3
Error rate (# mismatches/bases mapped)	0.046	0.067

### Genome annotations and functional analyses

**Figure 3:**
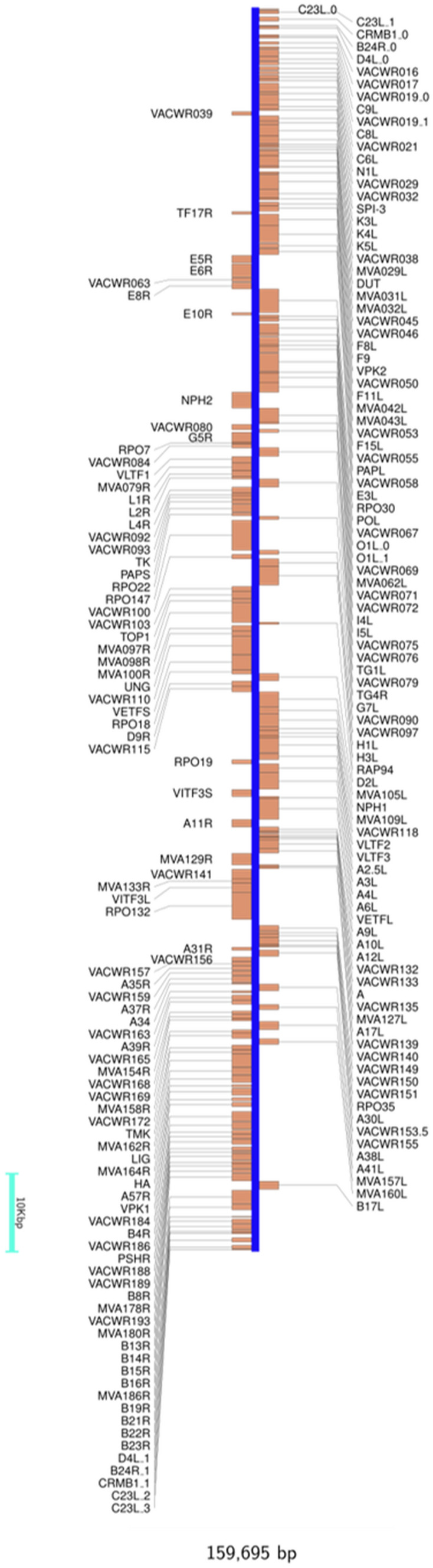
Annotated Ducapox gene map. The genome contained a total of 186 predicted genes.

The Ducapox genome was found to contain a total of 186 predicted protein coding genes (Fig. 3). A total of 194 genes were initially predicted by Prodigal, however, 8 of these predicted genes were found to contain no functional domain, and had no significant percentage identity to any protein in the Swissprot database, hence were removed from subsequent analyses. 13 out of these 186 proteins were found to contain predicted signal peptides (Table 3, supplementary information 3). A comparison of the proteins predicted by SignalP v5.0 (the latest version) and the signal peptides listed in the Uniprot database revealed that SignalP v5.0 predicted one previously unreported signal peptide in the protein A38L. Two proteins (A39R and HA) were found to have signal peptides predicted by SignalP v5.0 that matched those in the Uniprot database. The remaining 10 proteins contained signal peptides predicted by SignalP v5.0 that differed from those in the Uniprot database (predicted mature protein sequences in supplementary information 4). StructRNAfinder predicted a single structural RNA—the Pox_AX_element (RF00385), whis is involved in directing the efficient production and orientation-dependent formation of late RNAs^36,37^. A comparison of the predicted proteins from the long-read assembly against those generated from short read assembly was conducted using a protein blast, by aligning two or more sequences (BLOSUM62 comparison matrix; Gap costs: Existence 11, Extension 1). A total of 176 proteins were found to have equal length and 100% percentage identity between the two genome protein sets. An additional 7 proteins were found to have equal lengths and 100% identity, excepting for the fact that the short-read protein set contained letters that allowed for multiple amino acids to occupy the positions bringing the total identical proteins to 183 (supplementary information 5a). Of the remaining 3 proteins, 2 from the long-read assembly protein set were found to have better hit scores to VACV proteins in the UniProt database, and a single short read protein set had better hit scores to VACV proteins in the UniProt database (supplementary information 5a). Of the additional 10 proteins in the short-read protein set, 13 were found to either have no hit to VACV proteins in the UniProt database, or had hits that were less than half the length of a given protein.

**Table 3:** Predicted proteins containing predicted signal peptides. For each predicted protein, the conventional signal peptide as stated by Uniprot is listed, as well as the signal peptide predicted by SignalP v5.0. A novel signal peptide was predicted by SignalP v5.0 for the protein A38L.

Protein	Amino acid sequence	SignalP v5.0 prediction	Uniprot prediction
A38L	MSRVRISLIYLYTLVVITTTKTIEYTACNDTIIIPCTIDNPTKYIRWKLDNHDILTYNKTSKTTILSKWHTSARLHSLSDSDVSLIMEYKDILPGTYTCGDNTGIKSTVKLVQLHTNWFNDYQTMLMFIFTGITLFLLFLEITYTSISVVFSTNLGILQVFGCVIAMIELCGAFLFYPSMFTLRHIIGLLMMTLPSIFLIITKVFSFWLLCKSSCAVHLIIYYQLAGYILTVLGLGLSLKECVDGTLLLSGLGTIMVSEHFSLLFLVCFPSTQRDYY	MSRVRISLIYLYTLVVITTTKT	No signal peptide predicted
C8L	MSAIRFIACLYLISIFGNCHEDPYYQPFDKLNITLDIYTYEDLVPYTVDNDTTSFVKIYFKNFWITVMTKWCAPFIDTVSVYTSHDNLNIQFYSRDEYDTQSEDKICTIDVKARCKHLTKREVTVQQEAYRYSLSSDLSCFDSIDLEIDLIETNSTDTTVLKSYELMLPKRAKSIHN	MSAIRFIACLYLISIFGNC	MSAIRFIACLYLISIFGNCHE
HA	MTRLPILLLLISLVYATPFPQTSKKIGDDATLSCNRNNTNDYVVMSAWYKEPNSIILLAAKSDVLYFDNYTKDKISYDSPYDDLVTTITIKSLTARDAGTYVCAFFMTSPTNDTDKVDYEEYSTELIVNTDSESTIDIILSGSTHSPETSSEKPDYIDNSNCSSVFEIATPEPITDNVEDHTDTVTYTSDSINTVSASSGESTTDETPEPITDKEEDHTVTDTVSYTTVSTSSGIVTTKSTTDDADLYDTYNDNDTVPSTTVGGSTTSISNYKTKDFVEIFGITALIILSAVAIFCITYYIYNKRSRKYKTENKV	MTRLPILLLLISLVYA	MTRLPILLLLISLVYA
B19R	MKMTMKMMVHIYFVSLLLLLFHSYAIDIENEITEFFNKMRDTLPAKDSKWLNPACMFGGTMNDIAALGEPFSAKCPPIEDSLLSHRYKDYVVKWERLEKNRRRQVSNKRVKHGDLWIANYTSKFSNRRYLCTVTTKNGDCVQGIVRSHIKKPPSCIPKTYELGTHDKYGIDLYCGILYAKHYNNITWYKDNKEINIDDIKYSQTGKKLIIHNPELEDSGRYNCYVHYDDVRIKM	MKMTMKMMVHIYFVSLLLLLFHSYA	MTMKMMVHIYFVSLLLLLF
E10R	MNPKHWGRAVWTIIFIVLSQAGLDGNIEACKRKLYTIVSTLPCPACRRHATIAIEDNNVMSSDDLNYIYYFFIRLFNNLASDPKYAIDVTKVNPL	MNPKHWGRAVWTIIFIVLSQAGLDG	MNPKHWGR
B8R	MRYIIILAVLFINSIHAKITSYKFESVNFDSKIEWTGDGLYNISLKNYGIKTWQTMYTNVPEGTYDISAFPKNDFVSFWVKFEQGDYKVEEYCTGPPTVTLTEYDDHPYATRGSKKIPIYKRGDMCDIYLLYTANFTFGDSKEPVPYDIDDYDCTSTGCSIDFVTTEKVCVTAQGATEGFLEKITPWSSKVCLTPKKSVYTCAIRSKEDVPNFKDKMARVIKRKFN	MRYIIILAVLFINSIHA	MRYIIILAVLFIN
B7R	MYKKLITFLFVIGALASYSNNEYTPFNKLSVKLYIDGVDNIENSYTDDNNELVLNFKEYTISIITESCDVGFDSIDIDVINDYKIIDMSTIQRRGHTCRISTKLSCHYDKYPYIHKYDGDERQYSITAEGKCYKGIKYEISMINDDTLLRKHTLKIGSTYIFDRHGHSNTYYSKYDF	MYKKLITFLFVIGALASYS	MYKKLITFLFVIGALA
A28L	MNSLSIFFIVVATAAVCLLFIQGYSIYENYGNIKEFNATHAAFEYSKSIGGTPALDRRVQDVNDTISDVKQKWRCVVYPGNGFVSASIFGFQAEVGPNNTRSIRKFNTMQQCIDFTFSDVININIYNPCVVPNINNAECQFLKSVL	MNSLSIFFIVVATAAVCLLFIQG	MNSLSIFFIVVATAAVCLLFI
B16R	MSILPVIFLSIFFYSSFVQTFNAPECIDKGQYFASFMELENEPVILPCPQINTLSSGYNILDILWEKRGADNDRIIPIDNGSNMLILNPTQSDSGIYICITTNETYCDMMSLNLTIVSVSESNIDLISYPQIVNERSTGEMVCPNINAFIASNVNADIIWSGHRRLRNKRLKQRTPGIITIEDVRKNDAGYYTCVLEYIYGGKTYNVTRIVKLEVRDKIIPSTMQLPEGVVTSIGSNLTIACRVSLRPPTTDADVFWISNGMYYEEDDGDGDGRISVANKIYMTDKRRVITSRLNINPVKEEDATTFTCMAFTIPSISKTVTVSIT	MSILPVIFLSIFFYSSFVQT	MSILPVIFLSIFFYSSFV
SPI-3	MIALLILSLTCSASTYRLQGFTNAGIVAYKNIQDDNIVFSPFGYSFSMFMSLLPASGNTRIELLKTMDLRKRDLGPAFTELISGLAKLKTSKYTYTDLTYQSFVDNTVCIKPSYYQQYHRFGLYRLNFRRDAVNKINSIVERRSGMSNVVDSNMLDNNTLWAIINTIYFKGIWQYPFDITKTRNASFTNKYGTKTVPMMNVVTKLQGNTITIDDKEYDMVRLPYKDANISMYLAIGDNMTHFTDSITAAKLDYWSFQLGNKVYNLKLPKFSIENKRDIKSIAEMMAPSMFNPDNASFKHMTRDPLYIYKMFQNAKIDVDEQGTVAEASTIMVATARSSPEKLEFNTPFVFIIRHDITGFILFMGKVESP	MIALLILSLTCSA	MIALLILSLTCSAST
A39	MIPLLFILFYFANGIEWHKFETSEEIISTYLLDDVLYTGVNGAVYTFSNNKLNKTGLTNNNYITTSIKVEDAEPITEIPNVGK	MIPLLFILFYFANG	MIPLLFILFYFANG
PS/HR	MKTISVVTLLCVLPAVVYSTCTVPTMNNAKLTSTETSFNNNQKVTFTCDQGYHSSDPNAVCETDKWKYENPCKKMCTVSDYISELYNKPLYEVNSTMTLSCNGETKYFRCEEKNGNTSWNDTVTCPNAECQPLQLEHGSCQPVKEKYSFGEYITINCDVGYEVIGASYISCTANSWNVIPSCQQKCDIPSLSNGLISGSTFSIGGVIHLSCKSGFILTGSPSSTCIDGKWNPILPTCVRSNEKFDPVDDGPDDETDLSKLSKDVVQYEQEIESLEATYHIIIVALTIMGVIFLISVIVLVCSCDKNNDQY	MKTISVVTLLCVLPAVVYS	MKTISVVTLLCVLPAVV
A43R	MMMMKWIISILTMSIMPVLAYSSSIFRFHSEDVELCYGHLYFDRIYNVVNIKYNPHIPYRYNFINRTLTVDELDDNVFFTHGYFLKHKYGSLNPSLIVSLSGNLKYNDIQCSVNVSCLIKNLATSTSTILTSKHKTYSLHRSTCITIIGYDSIIWYKDINDIYDFTAICMLIASTLIVTIYVFKKIKMNS	MMMMKWIISILTMSIMPVLA	MMMMKWIISILTMSIMPVLAYS

### Assessment of putative epigenetic modification sites

**Figure 4:**
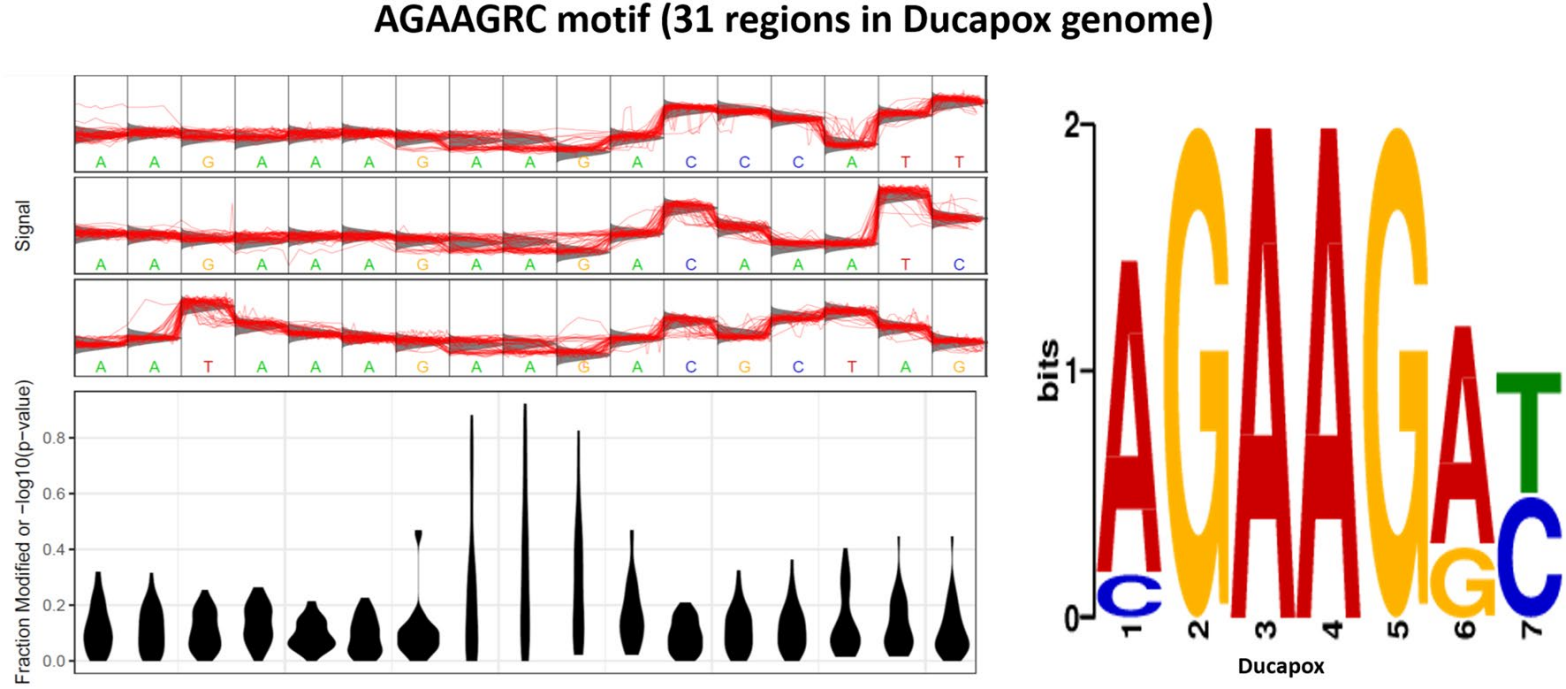
Statistical plot and sequence logo of the AGAAGRC motif. The statistical plot is based on 17 regions within the genome that contain the motif sequence. Signal fluctuation away from the canonical model can be seen around the central AAG nucleotides.

**Figure 5:**
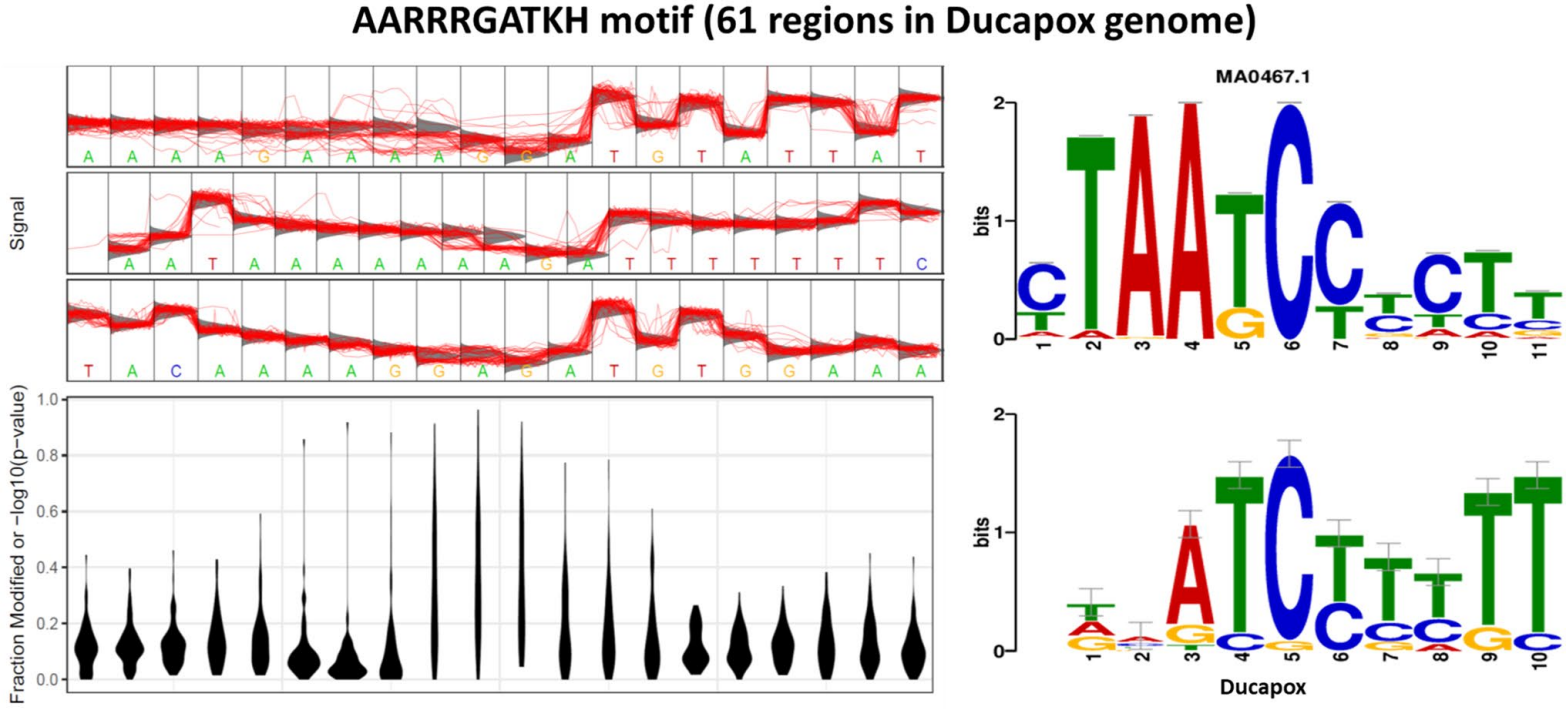
Statistical plot and sequence logo of the AARRRGATKH motif. The statistical plot is based on 42 regions within the genome that contain the motif sequence. Signal fluctuation away from the canonical model can be seen around the central GA nucleotides.

A total of three motifs were identified in the Ducapox genome that consistently produced raw signals that diverged from the standard model. The AGAAGRC motif was found at 31 regions within the genome of which 24 regions had a coverage > 50. Signal fluctuations differing from the canonical model were observed around the central AAG nucleotides (Fig. 4). A Tomtom search of the motif detected no similar known motifs. The AARRRGATKH motif was found at 61 regions within the genome of which 48 regions had a coverage > 50. Signal fluctuations differing from the canonical model were observed around the central GA nucleotides (Fig. 5). A Tomtom search of the motif showed the reverse-complement to most closely match MA0467.1 (Crx binding motif; Mus musculus) in the JASPAR database.

**Figure 6:**
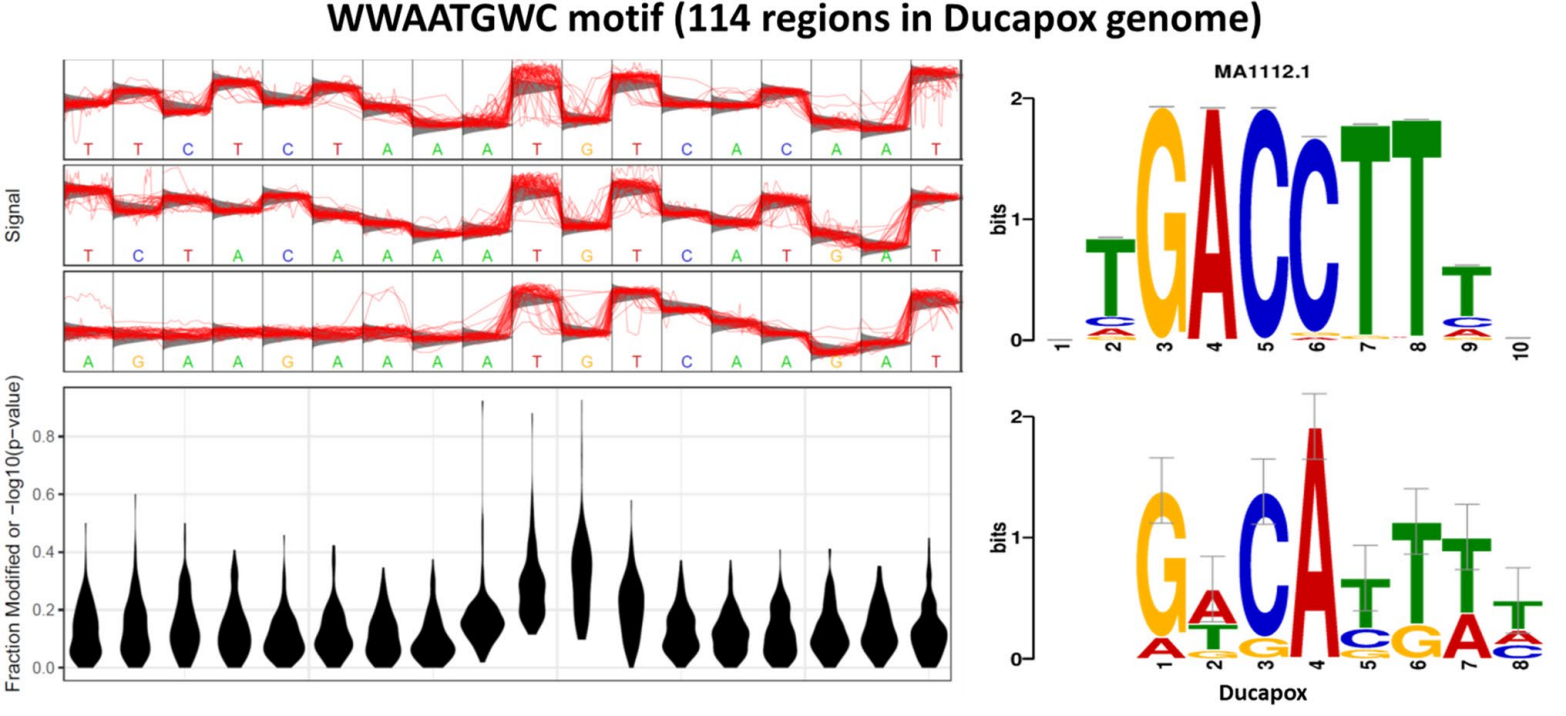
Statistical plot and sequence logo of the WWAATGWC motif. The statistical plot is based on 77 regions within the genome that contain the motif sequence. Signal fluctuation away from the canonical model can be seen around the central TGT nucleotides.

The WWAATGWC motif was found to be present at 114 regions within the genome of which 90 regions had a coverage > 50. Signal fluctuations differing from that of the canonical model were observed around the central TGT nucleotides (Fig. 6). A Tomtom search of the motif showed the reverse-complement to most closely match MA1112.1 (NR4A1; Homo sapiens) in the JASPAR database. For each putatively modified motif detected by Tombo, the coverage, genomic position, signal fluctuations compared to a standard model, and number of regions containing each motif can be found in the TomboResultsOutput folder of the project Git (https://github.com/zacksaud/Ducapox-Assembly-Project/tree/master/TomboResultsOutput). No methylation sites with a frequency above 0.5 were detected with Nanopolish (supplementary information 6). No evidence of 5mC methylation was detected by Megalodon (supplementary information 7).

## Discussion

Except for two confirmed genomic deletions, the whole genome sequence of this vaccine was shown to closely resemble that of VACV-MVA, supporting our earlier study in which we reported that two gene regions of this vaccine most closely resembled those of the aforementioned strain^34^. Our findings also corroborate with a previous study that used short read Illumina sequencing, and a reference guided assembly to generate a partial Ducapox genome, wherein the authors noted the putative deletions, but could not confirm the validity of the deletions due to the both the assembly pipeline and sequencing technology used^35^. At 159,695 bp in length, the vaccine genome, to our knowledge, is the smallest amongst the non-vector derived orthopoxviruses. We postulate that the deletions may have been a result of passage of a misidentified VACV-MVA strain, as it is known that poxvirus genomes tend to decrease in size with serial passage^38^. It has been demonstrated that VACV has a defined origin of replication, which supports a model for poxvirus genome replication that involves leading and lagging strand synthesis^39^. Studies on poxvirus DNA replication described putative Okazaki fragments of about 1,000 nt in length (suspiciously similar in size to the 916 bp deletion of the Ducapox sequence) and RNA primers on the 5′-ends of newly made chains of VACV DNA^40,41^.

We predicted a previously unreported signal peptide in protein A38L. The A38L gene product is a 33 kDa integral membrane glycoprotein^42^. Overexpression of the protein has been shown to promote Ca^2+^ influx into infected cells^43^. The latest version of SignalP predicted alternate peptide signals for 10 other proteins. These include; the gene product of C8L—the function of which remains unknown, the gene product of B19R—a type 1 interferon decoy^44^, the gene product of E10R—associated with membranes of intracellular mature virions and plays a role in morphogenesis^45^, the gene product of B8R- another interferon decoy^44^, the gene product of B7R- which is involved with virulence^46^, the gene product of B16R- an IL-1β binding protein^47^, the gene product of SPI-3- a cell fusion inhibitor protein^48^, the gene product of PS/HR—which plays a role in the dissolution of the outermost membrane of extracellular enveloped virions to allow virion entry into host cells and also participates in wrapping mature virions to form enveloped virions^49^, and finally the gene product of A43R—which enhances intradermal lesion formation^50^. Signal peptides play a range of different roles within cells that include marking proteins for secretion, intracellular translocation, and keeping catalytic proteins in an inactive precursor form until the signal peptide is cleaved^51^. Further research is needed to determine whether biochemical analyses of these new mature proteins yield any further insight into protein function.

We have presented regions within the Ducapox genome that contain motifs wherein the Nanopore signal diverges from the standard model, which may be indicative of bases within these regions containing epigenetic modifications. Although the Nanopore sequencing is a valuable tool for identifying putative epigenetic sites within a genome, the device does not allow for the identification of either the individual base that is modified, nor does it allow for the identification of the modifying chemical group. Thus, further analyses are required to confirm the results, such as isolation and purification of the motifs containing the putative epigenetic modifications and generating amplicons that could be Nanopore sequenced to confirm reversion of the amplicon raw signal to that of the standard model. Modifications that distinguish viral DNA from that of the host may be targets for advanced therapeutics. Should these epigenetic modifications be confirmed and chemically characterized, another important question would concern whether the modifications were the result of a viral protein, or the result of a host protein, and whether the base modifications are exclusive to the isolate of Vaccinia virus, or more widely distributed amongst poxviruses.

Given the relative cheapness of Nanopore sequencing, future research could investigate the evolutionary trajectory of orthopoxviruses with continued passage. Experiments such as determining whether different evolutionary trajectories occur when a seed stock of a virus is passaged in differing permissive cell lines would be of great interest. Furthermore, the Nanopore would allow for the assessment of differing epigenome modifications with continued passage. Such studies would assist in providing further evidence towards efforts to better understand the origins of Vaccinia virus^52^. Additionally, long read sequencing transcriptomics techniques have recently shed light on the high variation in transcript lengths at certain Vaccinia genome loci, termed chaotic regions^53,54^. Long read sequencing coupled with these transcriptomics techniques could provide greater insight into the loss of Poxvirus virulence with passage. Much research has gone into the elucidation of nucleic acid modifying proteins of Vaccinia virus, for instance, Vaccinia virus K7R protein has been shown to promote histone methylation associated with heterochromatin formation^15^. Furthermore, it is postulated that epigenetic and genetic mechanisms may also lead to VACV-induced transcription silencing, and VACV infection induces a global degradation of host and viral mRNA^55^. Also, VACV mRNA capping is carried out in three reactions performed by viral enzymes wherein guanine N-7 methylation occurs, and VACV encodes the VP39 protein (J3R) that is known to add a methyl group at the 2′-O position of the first transcribed nucleotide adjacent to the 5′ cap^55^. Poxviruses are unique among most DNA viruses in that DNA replication occurs in the cytoplasm, independent of the nucleus of the infected host cell, and accordingly, its genome encodes for factors required for both cytoplasmic transcription as well as DNA replication^5^. Hence should the putative epigenetic modifications of the viral DNA be validated, it would be likely that either viral proteins, or host cytoplasmic proteins would be implicated in the base modification process, as opposed to host nuclear proteins. Many mammalian cytoplasmic proteins are known to bind viral nucleic acids^56^.

To conclude, we have developed a novel assembly pipeline for long read sequencing of Poxvirus genomes, that corrects the lengths of terminal ends. The two confirmed deletions of this vaccine strain in comparison to VACV-MVA make it one of the smallest non-vector derived orthopoxvirus genomes to be reported. We have used the latest software for signal peptide prediction to discover a novel predicted signal peptide in a VACV protein that has not been previously reported, as well as discovering 10 alternate predicted signal peptides in comparisons to those previously reported. We have presented putative epigenetic modifications within the Ducapox genome, based on divergence of the raw signals from a standard model for given sequence motifs. The methods we have detailed may be used for other viral genomes, thus aiding the understanding of the molecular mechanisms underpinning viral virulence, evolution and host preferences.

## Methods

### Source and composition of vaccine

A commercial live attenuated ‘Ducapox’ vaccine was sourced from Al Bashayer Veterinary Supplies (Dubai, United Arab Emirates), manufactured by Design Biologix (Pretoria, South Africa) and commercialized by Highveld Biological Ltd (Johannesburg, South Africa). The CMLV strain CaPV298-2, the parent strain of this vaccine, was originally isolated in the United Arab Emirates and attenuated through serial passage in Vero cell culture^33^. Manufacture and expiry dates were 07–2018 and June 2019, respectively and the batch number was DPV0818.

### DNA extraction

DNA was extracted using the QIAamp DNA Mini kit (Catalog # 51304, Qiagen, Hilden, Germany), following the DNA purification from tissues protocol, adding 180 μL of Buffer ATL to 25 mg of lyophilized vaccine and following the manufacturer's guidelines with the addition of adding 5 μg of Carrier RNA Poly A (Catalog # 1,017,647, Qiagen, Hilden, Germany) to the 200 μL of Buffer AL solution. The DNA preparation was analyzed for purity on a nanodrop spectrophotometer (ThermoScientific, Rochester, USA), and the concentration was determined using a Qubit dsDNA assay kit (ThermoScientific, Rochester, USA) and a Qubit 4 fluorometer (ThermoScientific, Rochester, USA).

### Preparation of nanopore library and sequencing

400 ng of genomic DNA was used for Nanopore library preparation using a Rapid Sequencing Kit (SQK-RAD004, Oxford Nanopore Technologies) and barcode 18 of the Native Barcoding Expansion kit (EXP-NBD114, Oxford Nanopore Technologies). Multiplexed sequencing was performed on a MinION device (Oxford Nanopore Technologies), equipped with a R9.4.1 MinION flow cell. Base calling was performed offline with ONT’s Guppy software pipeline version 4.0.11, enabling the—pt_scaling flag, setting—trim_strategy to DNA, loading the dna_r9.4.1_450bps_hac configuration files, and setting—barcode_kits EXP-NBD114.

### Long read— pre-processing, assembly, and polishing

**Figure 7:**
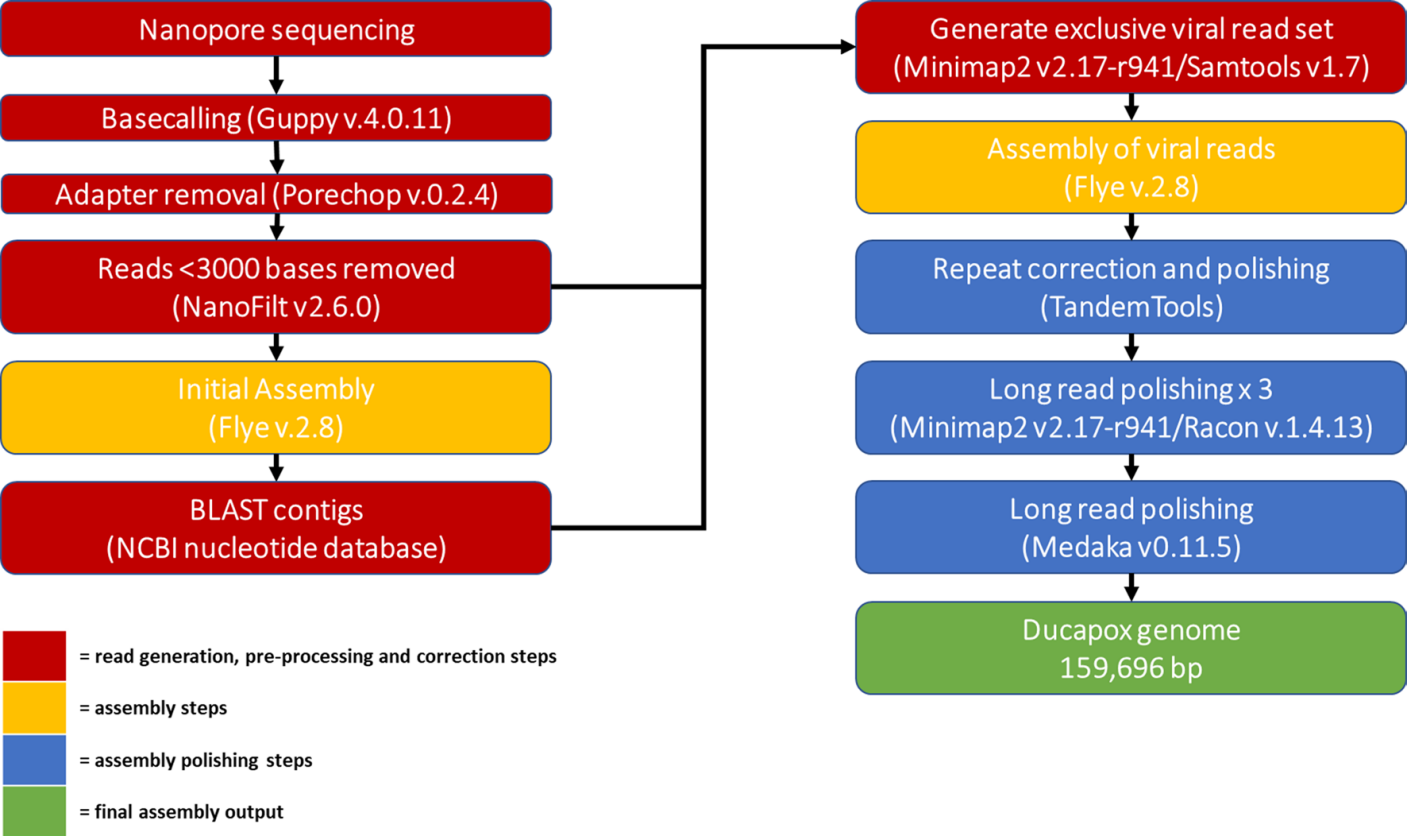
Bioinformatics pipeline used for the long-read only assembly of the Ducapox genome. Basecalling of reads was performed using Guppy v4.0.11. Adapter sequences in reads were removed using Porechop v.0.2.4. Reads were subsequently filtered to a minimum length of 3000 bases using Nanofilt v2.6.0. An initial assembly was performed using Flye v.2.8 (using reads containing both viral and non-viral DNA sequences), after which a BLAST search for each contig generated was performed against the NCBI nucleotide database. A file containing all non-viral reads was used to generate an exclusive viral read set by mapping reads to the non-viral contigs using Minimap2 v 2.17-r941, followed by extraction of the unmapped reads using Samtools v1.7. A Flye assembly was performed on the exclusive viral reads set, which was subsequently polished with TandemTools, followed by 3 rounds of Racon v.1.4.13 polishing, and a final polishing round using Medaka v0.11.5 to generate a 159,696 bp genome. An incorrect insertion within an adenine homopolymer region of this assembly was corrected, producing a final genome sequence length of 159,695 bp.

Long read adapter trimming was performed with Porechop version 0.2.4 (www.github.com/rrwick/Porechop), setting both the—adapter_threshold and—barcode_threshold to 98. The trimmed long reads were filtered to remove reads under 3000 bases in length using NanoFilt version 2.6.0^57^. The adapter trimmed, filtered long reads were assembled using Flye version 2.8^58^ using the—nano-raw,—meta,—trestle and—keep-haplotypes flags. A fasta file of non-viral assembled contigs (identified using a blast search) was made from the assembly output using Bandage version 0.8.1^59^. The adapter trimmed, filtered long reads were mapped to the non-viral assembled contigs using minimap2 version 2.17-r941^60^, and the unmapped reads were extracted from the alignment file and converted to FASTQ using samtools^61^, thus generating a read set exclusively containing viral DNA. The virus specific reads were assembled using Flye version 2.8, enabling the—nano-raw, setting the minimum overlap to 5000 using the -m 5000 flag, and conducting 3 polishing iterations by setting the -i 3 flag. The assembly was polished, correcting the ITR regions, using the—only-polish flag of the tandemquast tool of the TandemTools package^62^. Long reads were mapped to the assembly using minimap2 version 2.17-r941, and the resulting alignment file was used to polish the assembly with Racon version v1.4.13^63^ using the following parameters: -m 8 -x -6 -g -8 -w 500 -no-trimming. A total of 3 rounds of mapping and polishing with Racon were done on the assembly, after which no changes were observed. The corrected consensus was further polished with the same long read set using Medaka version 0.11.5 (https://github.com/nanoporetech/medaka), setting the—m r941_min_high_g360 flag. Figure 7 shows a graphical representation of the full assembly pipeline.

### Assessment of assemblies and whole genome comparisons

The non-viral-DNA-free, adapter trimmed, filtered long reads were mapped to both the initial Flye assembly, and the final polished assembly in order to manually assess for the absence of read mapping breaks by plotting read mapping coverage of genome assemblies using pyGenomeTracks version 3.5^64^. Genome comparisons were performed using the nucmer tool of Mummer 3^65^. The final polished assembly was compared against the short-read Ducapox assembly (Genbank accession: MT648498.1) and Vaccinia virus strain Acambis 3000 MVA (Genbank accession: AY603355.1), the closest matching genome to the long-read assembly as determined by an online BLAST search.

### Genome annotation

The polished assembly was annotated using Prodigal v2.6.3^66^. The annotation gff3 file was loaded into GenSAS suite version 6.0^67^, after which functional analyses were conducted in the suite using InterProScan version 5.25–68.0^68^ and the ab initio predicted proteins were identified using blastp^69^ by conducting a protein vs protein search against the SwissProt protein data set to determine best matches. Protein sequences were analyzed for predicted signal peptides using the SignalP v5.0^70^. Non-coding RNAs were detected using StructRNAfinder^71^.

### Assessment of putative epigenetic modification sites

A total of 2214 Fast5 files (599.9 MB) that mapped to the long-read assembly were extracted using the fast5seek tool (github.com/mbhall88/fast5seek). The Tombo suite^26^ was used to detect Nanopore raw signals that diverged from the standard model, which could signify epigenetic modification sites. After running Tombo’s resquiggle function using the final polished genome, the detect_modifications function was run using the de_novo model with default parameters (dampened fraction estimation [2, 0]). The results of the stats file was converted to a FASTA file using the text_output function of Tombo, setting—num-regions 1000 and—num-bases 15. The central 7 nucleotides of each entry of the fasta file was plotted using the motif_with_stats (plotting the standard model, and default dampened fraction estimation [2, 0]) in Tombo, using the maximum—num-statistics number that would produce a plot for each fasta entry (determined empirically) for all entries with scores > 0.7 for “Frac. Alternate” in the fasta file. The motif_with_stats plots were assessed manually, and the motifs from plots containing increases in the fraction of modified bases (− log10(P-value) exclusively around the central motif only were kept, and these were used to create a separate fasta file containing all motifs for each of the four modified bases that were manually detected from the plots. Meme v5.1.1^72^ was used on each individual fasta file using the—dna and—mod zoops flags to determine motifs. Motifs were compared to known motifs using Tomtom v5.1.1^73^. Nanopolish v0.13.3 was used to assess for 5mC and 6 mA epigenetic modifications (75), setting a methylation frequency of above 0.5 as indicative of evidence for methylation. The presence of 5 mC epigenetic modifications were also assessed using Megalodon (github.com/nanoporetech/megalodon).

## Supplementary Information

Supplementary Information 1.

Supplementary Information 2.

Supplementary Information 3.

Supplementary Information 4.

Supplementary Information 5.

Supplementary Information 6.

Supplementary Information 7.

## Data Availability

All data generated in this study has been deposited at the NCBI under Bioproject PRJNA663037. Nanopore sequencing read data can be accessed at the NCBI SRA using the accession number SRR12667950. Sample information can be accessed at the NCBI BioSample repository using the accession number SAMN16115327. The long-read Ducapox genome assembly generated in this study can be accessed using GenBank accession number MT946551 (The 159,696 bp assembly as version MT946551.1 and the corrected 159,695 bp assembly as version MT946551.2). The short-read Ducapox assembly and protein sequences can be accessed using GenBank accession number MT648498.1. The Vaccinia Virus strain Acambis 3000 MVA genomes can be accessed using GenBank accession number AY603355.1. Gene and protein names, and functional annotations (GO terms, InterPro, PFAM) are included in GenBank entries. Bioinformatics tool output files have been deposited in the following GitHub repository-https://github.com/zacksaud/Ducapox-Assembly-Project, as well as in the supplementary information.
