# Supplementary material for: Nanopore sequencing and de novo assembly of a misidentified Camelpox vaccine reveals putative epigenetic modifications and alternate protein signal peptides
Source: Sci Rep. 2021 Sep 7;11:17758. doi: 10.1038/s41598-021-97158-x (PMC8423768; doi:10.1038/s41598-021-97158-x)
Supplement: Supplementary file 1 — Supplementary Information 1. [file 41598_2021_97158_MOESM1_ESM.docx]

**Nanopore sequencing and *de novo* assembly of a misidentified Camelpox vaccine reveals putative epigenetic modifications and alternate protein signal peptides**

**Zack Saud^1^*, Matthew D. Hitchings^2^, Tariq M. Butt^1^**

*^1^ Department of Biosciences, College of Science, Swansea University, Singleton Park, Swansea, SA2 8PP, Wales, United Kingdom*

*^2^ Swansea University Medical School, Swansea University, Singleton Park, Swansea, Sa2 8PP, Wales, United Kingdom*

*** Corresponding author

* Z. Saud: [zack.saud@swansea.ac.uk](mailto:zack.saud@swansea.ac.uk)

**Supplementary Information 1- Read mapping coverage of genome assemblies for a. the Ducapox short-read assembly and b. Vaccinia Virus Acambis 3000 MVA. In both cases, a large coverage of reads had mapped to the ITR at the 3’ end of the genomes, indicative of poor ITR assembly. The mappings also highlight the short-comings of adopting reference-based alignment assemblies using short-reads.**

**a.**

**
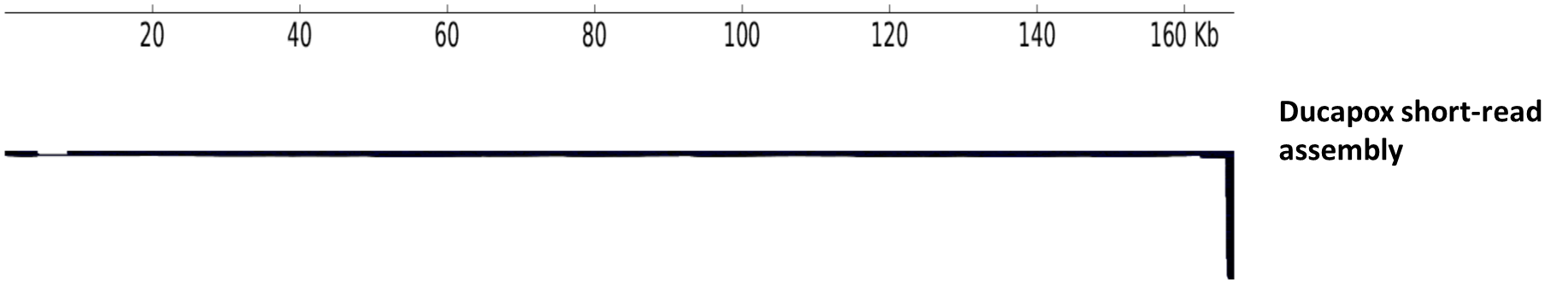
**

Relative Coverage

**b.**

**
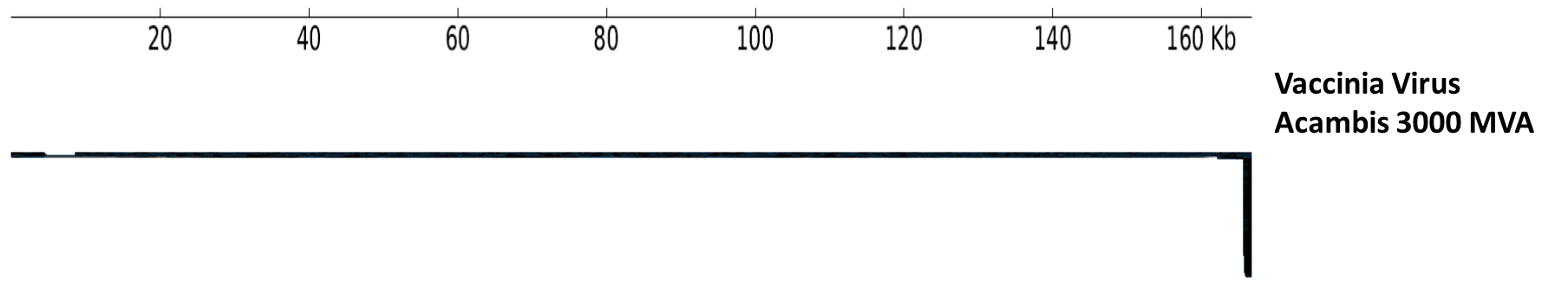
**

Relative Coverage
