# Supplementary material for: Nanopore sequencing and de novo assembly of a misidentified Camelpox vaccine reveals putative epigenetic modifications and alternate protein signal peptides
Source: Sci Rep. 2021 Sep 7;11:17758. doi: 10.1038/s41598-021-97158-x (PMC8423768; doi:10.1038/s41598-021-97158-x)

**Nanopore sequencing and *de novo* assembly of a misidentified Camelpox vaccine reveals putative epigenetic modifications and alternate protein signal peptides**

**Zack Saud^1^*, Matthew D. Hitchings^2^, Tariq M. Butt^1^**

*^1^ Department of Biosciences, College of Science, Swansea University, Singleton Park, Swansea, SA2 8PP, Wales, United Kingdom*

*^2^ Swansea University Medical School, Swansea University, Singleton Park, Swansea, Sa2 8PP, Wales, United Kingdom*

*** Corresponding author

* Z. Saud: [zack.saud@swansea.ac.uk](mailto:zack.saud@swansea.ac.uk)

**Supplementary Information 2- Evidence confirming the two deletions within the Ducapox genome**

1. Reads traversing the 5449 bp deletion site.
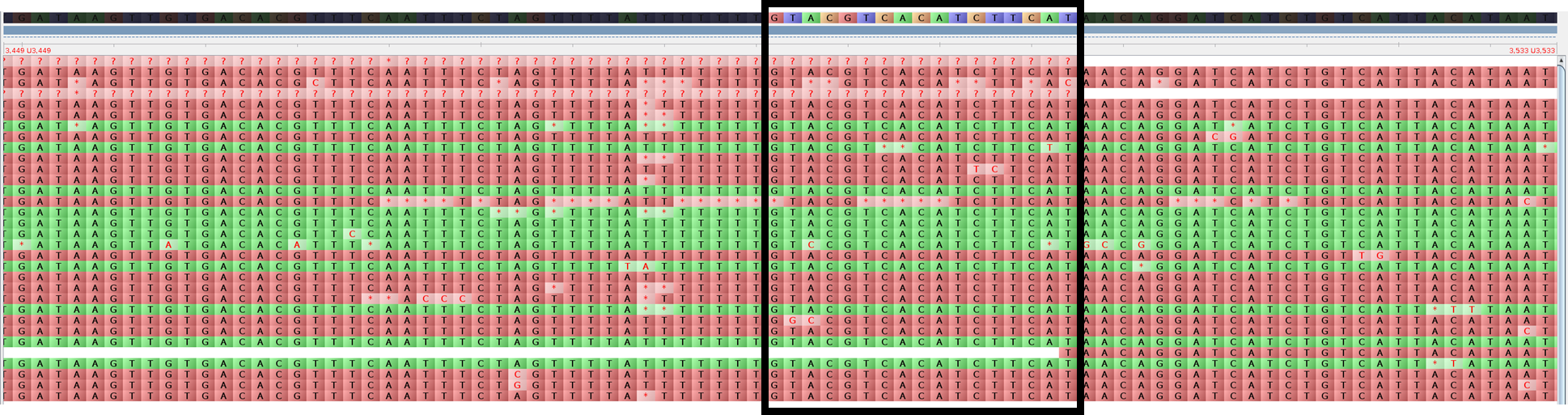

2. Reads traversing the 916 bp deletion site.
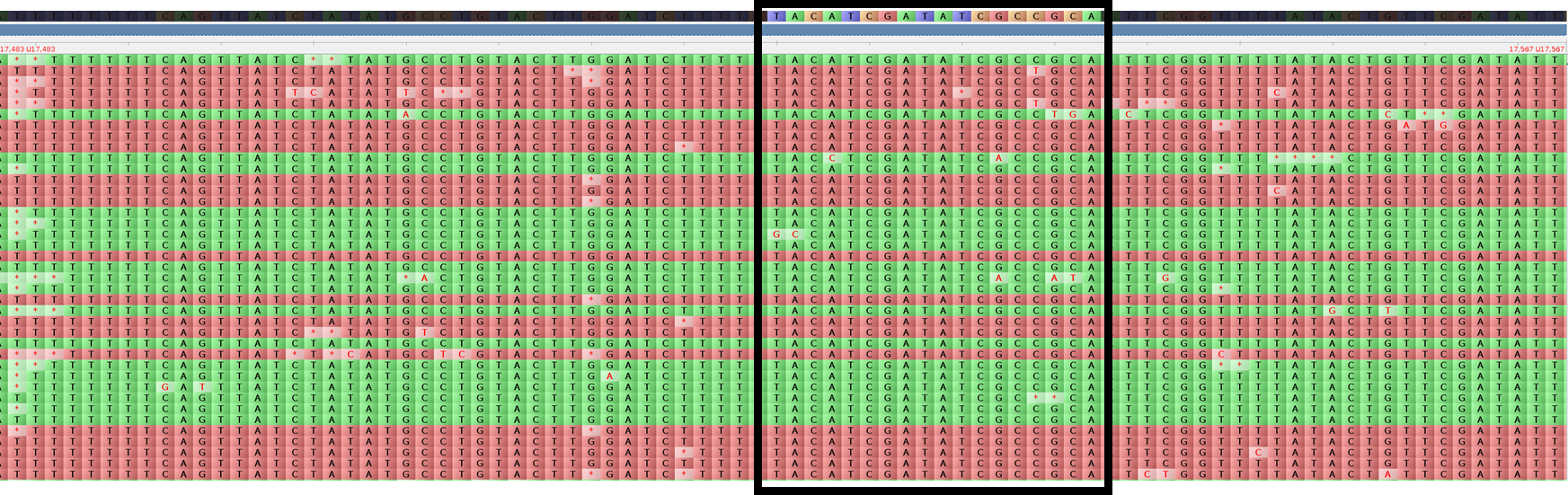


c. EMBL-EBI Clustal Omega multiple sequence alignment of short read Ducapox assembly (MT648498.1), long read Ducapox assembly (Vaccinia) and VACV Acambis 3000 MVA (AY603355.1) genomes.

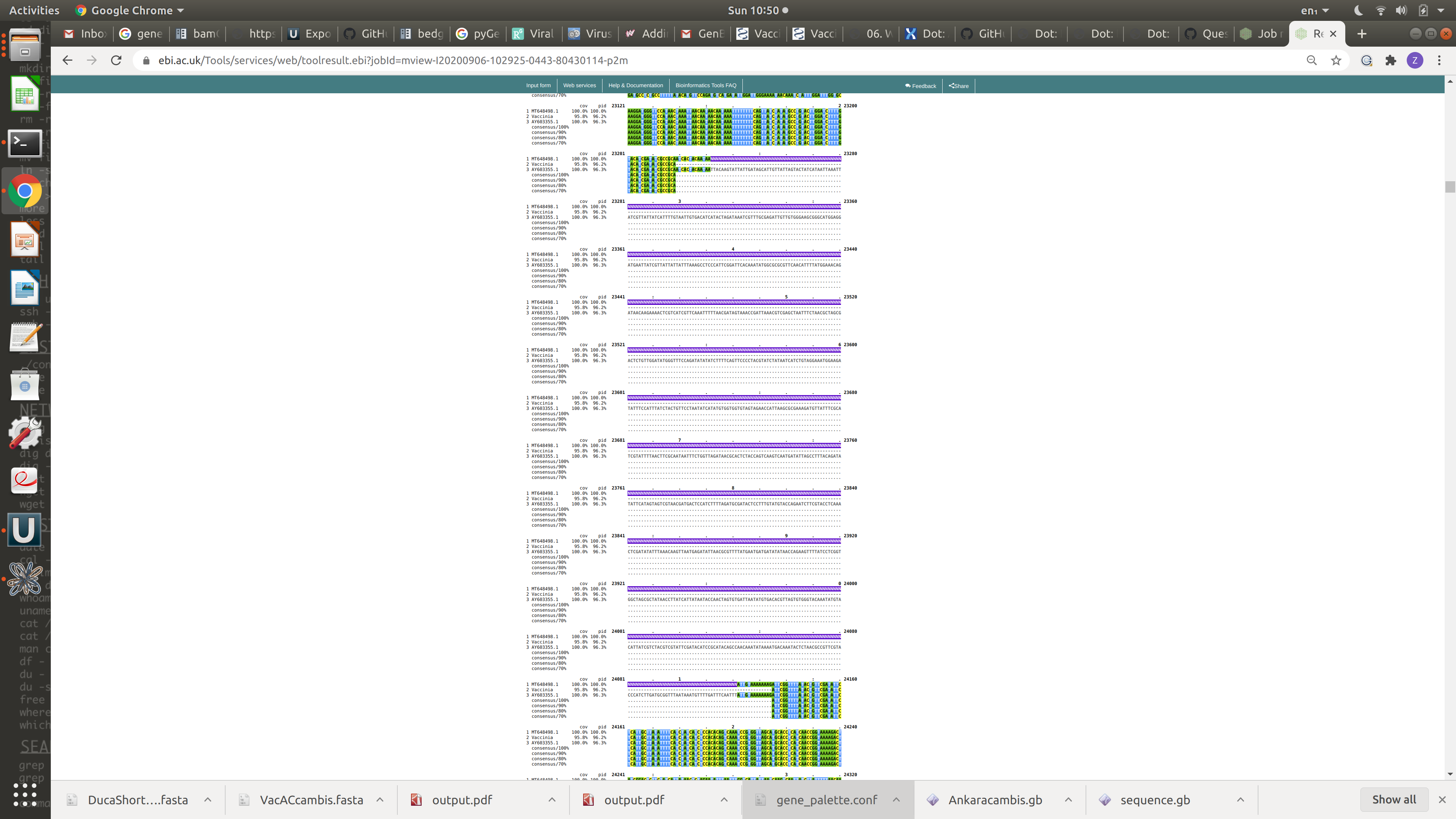


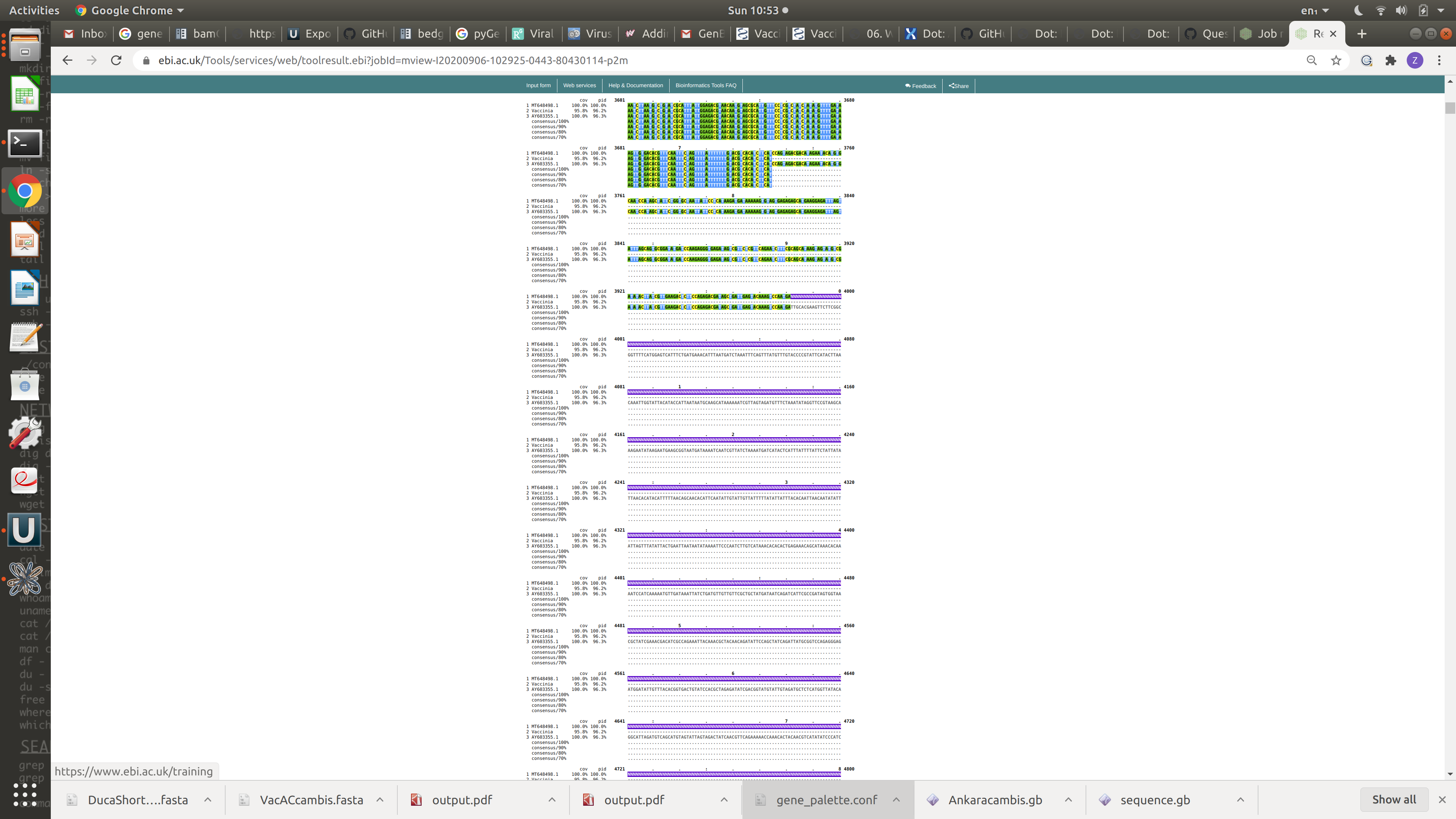

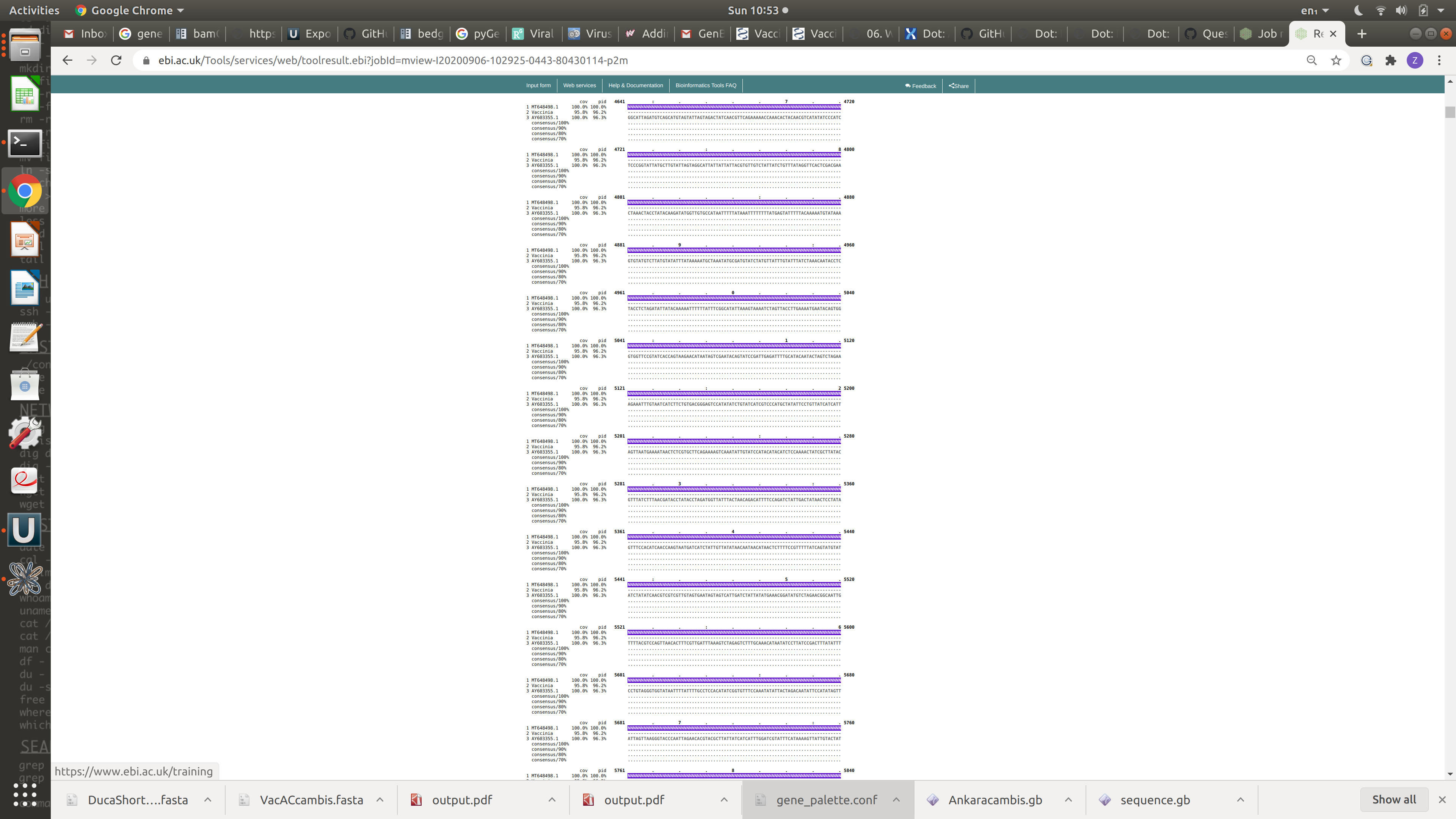

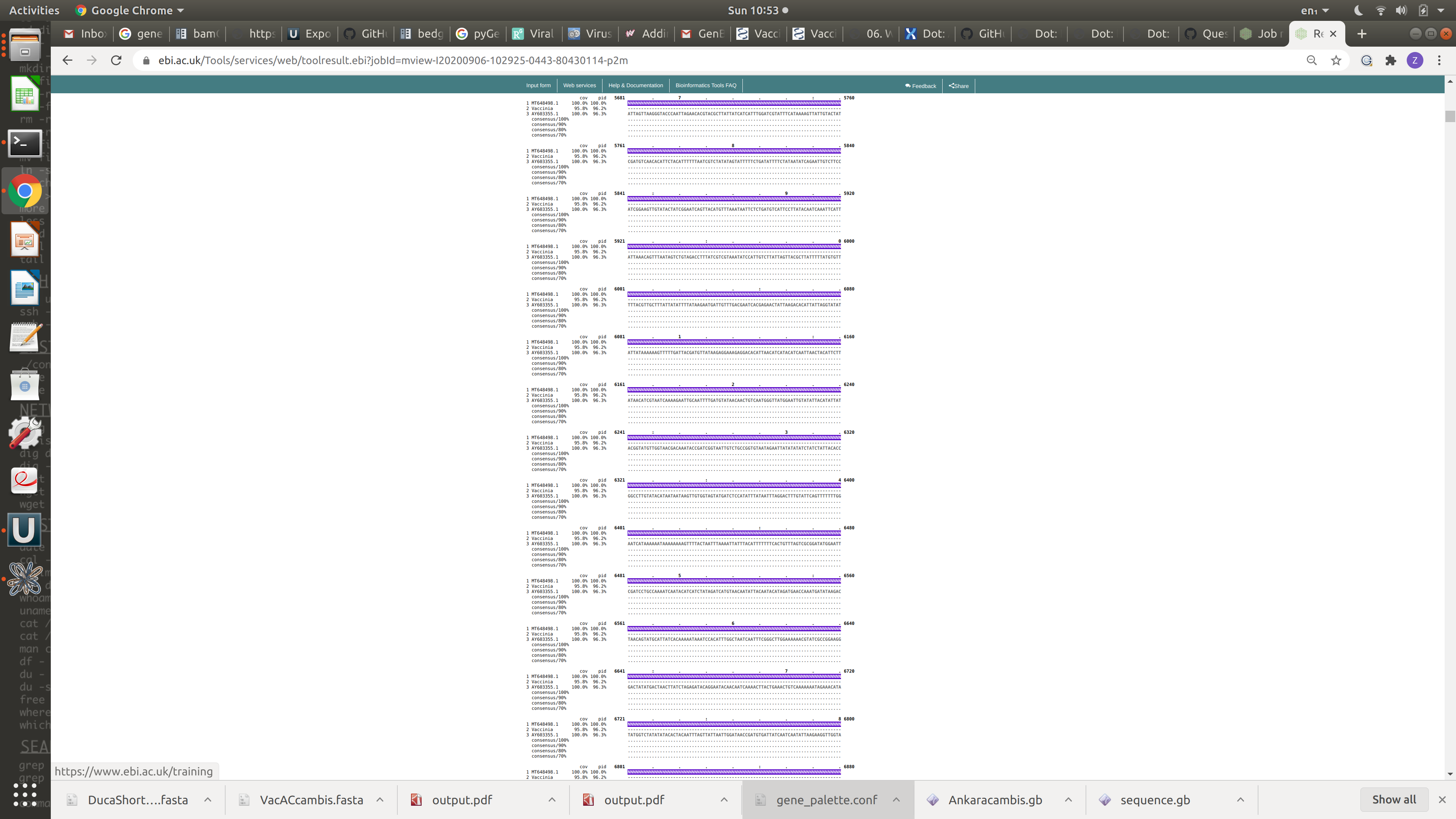

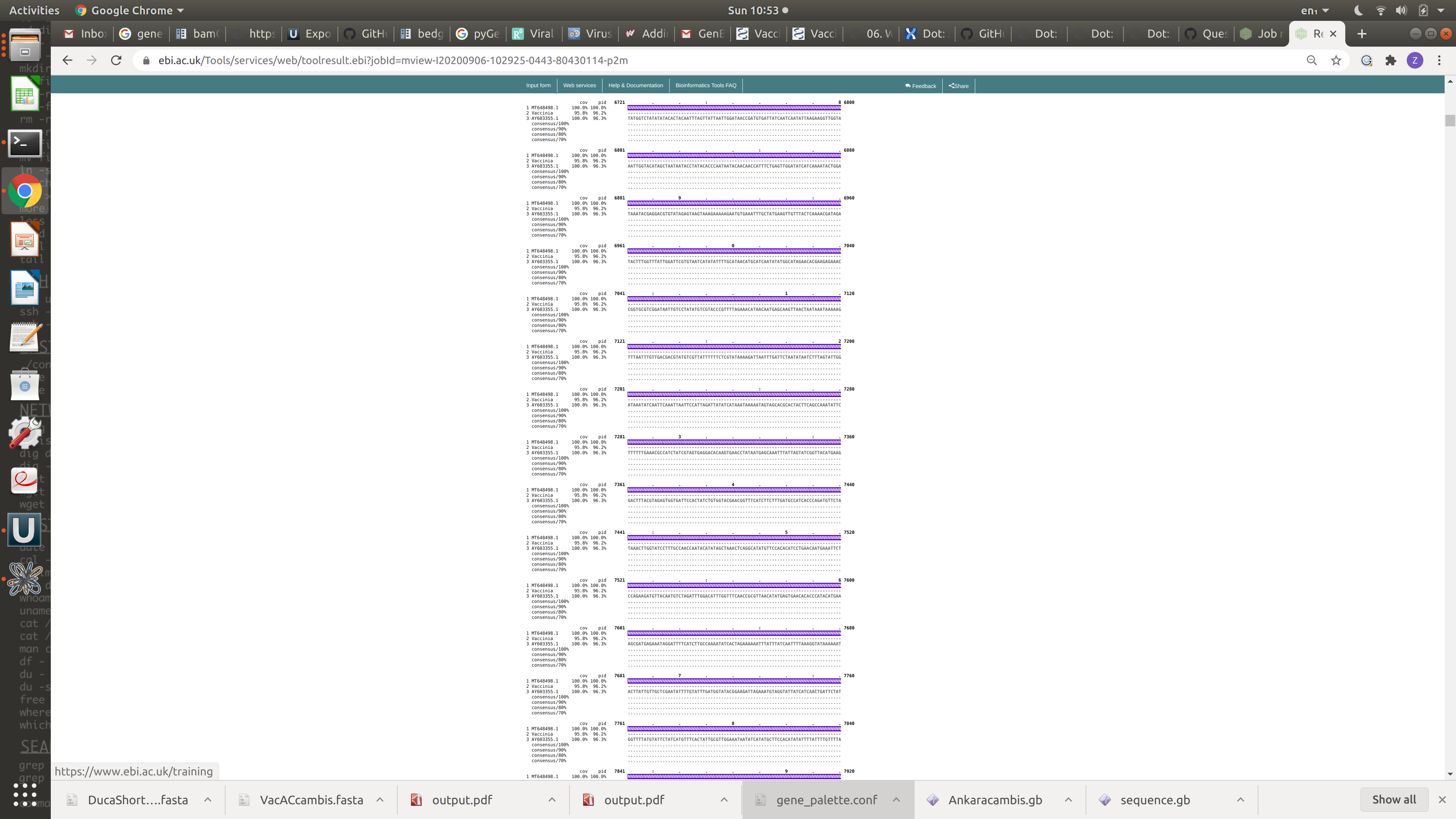

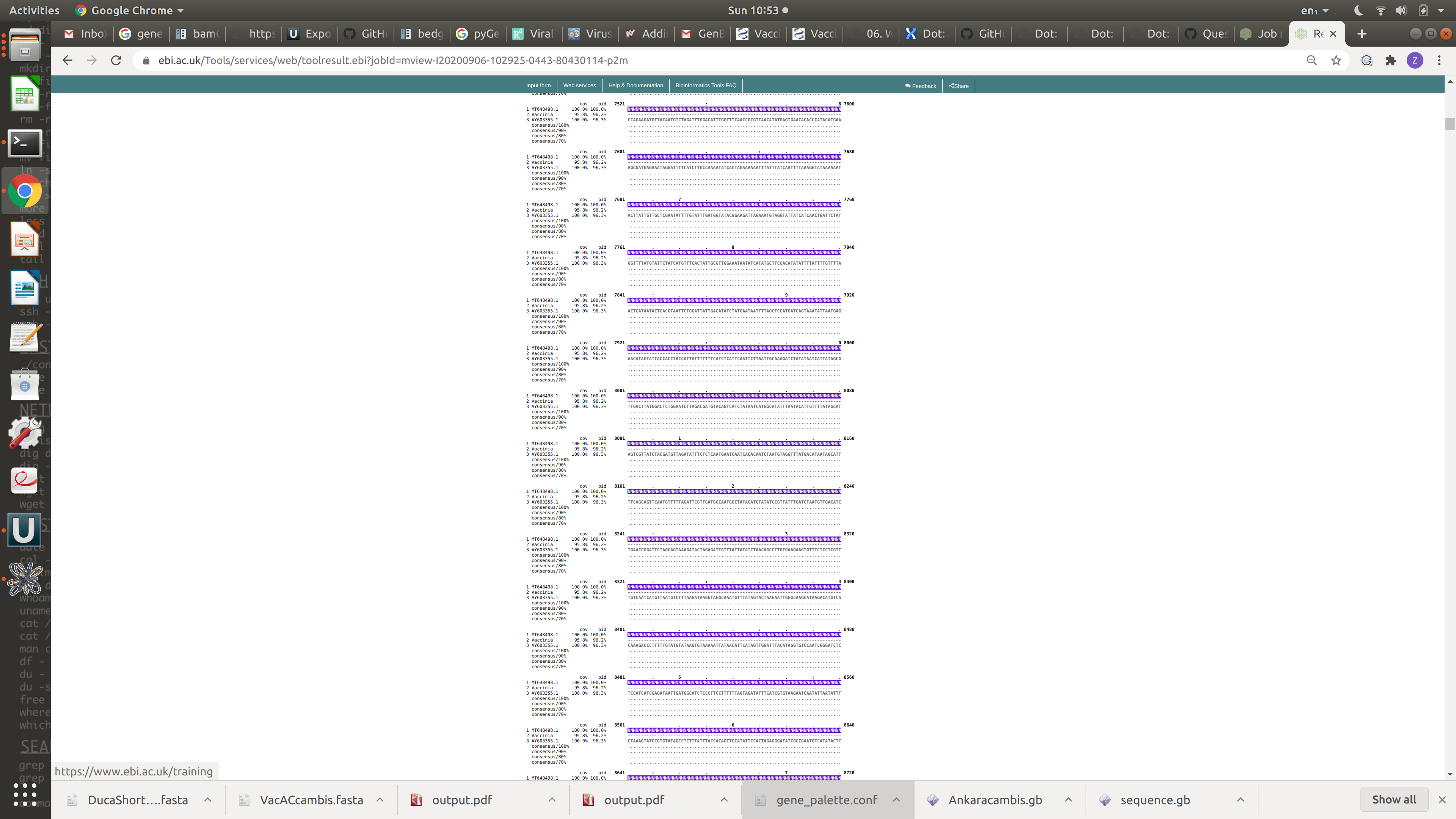

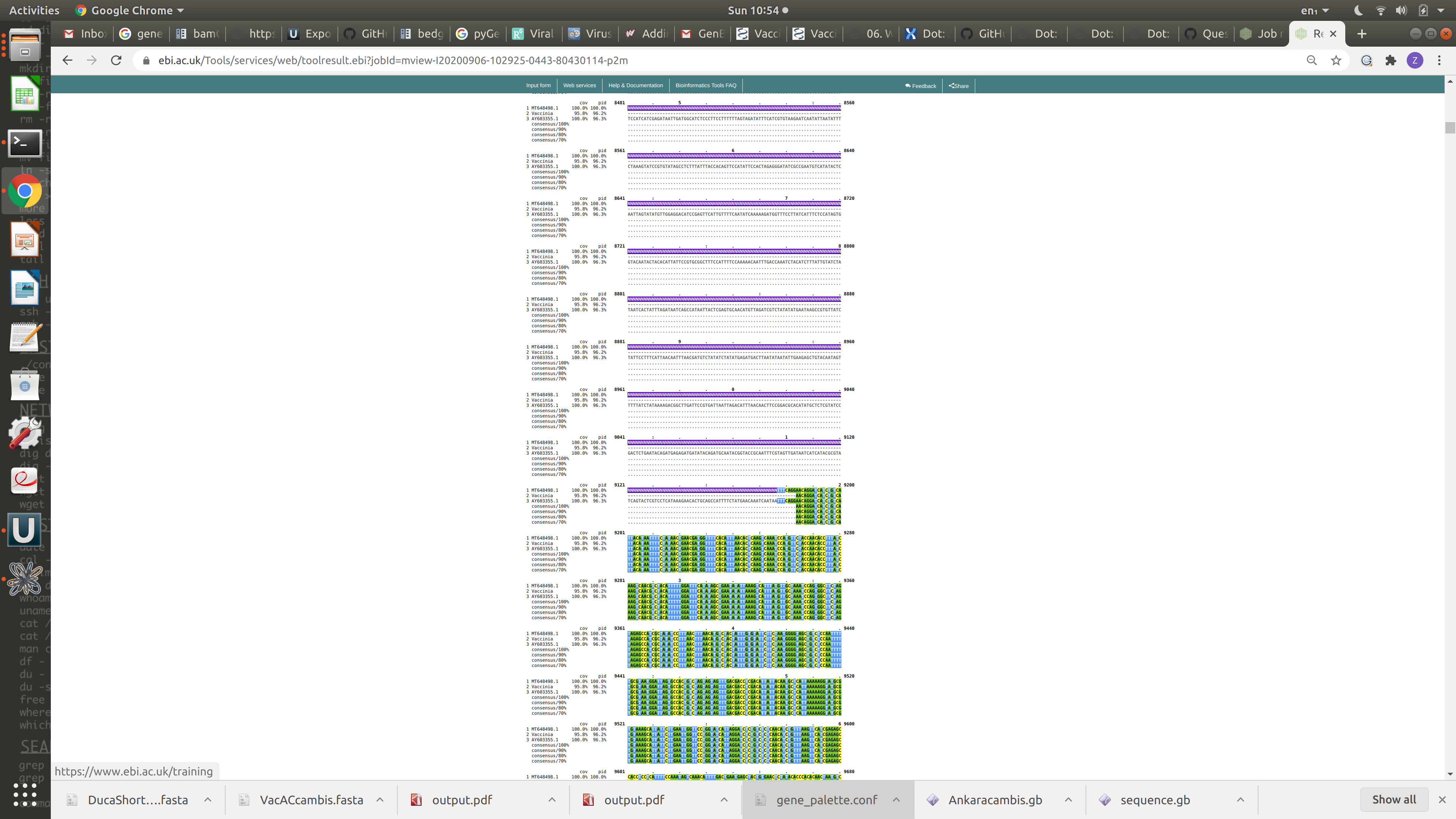


1. **Additional adenine reside which resulted in split protein D5R in long read assembly. A new open reading frame with a frameshift (red arrow) compensated the addition in the second protein.**


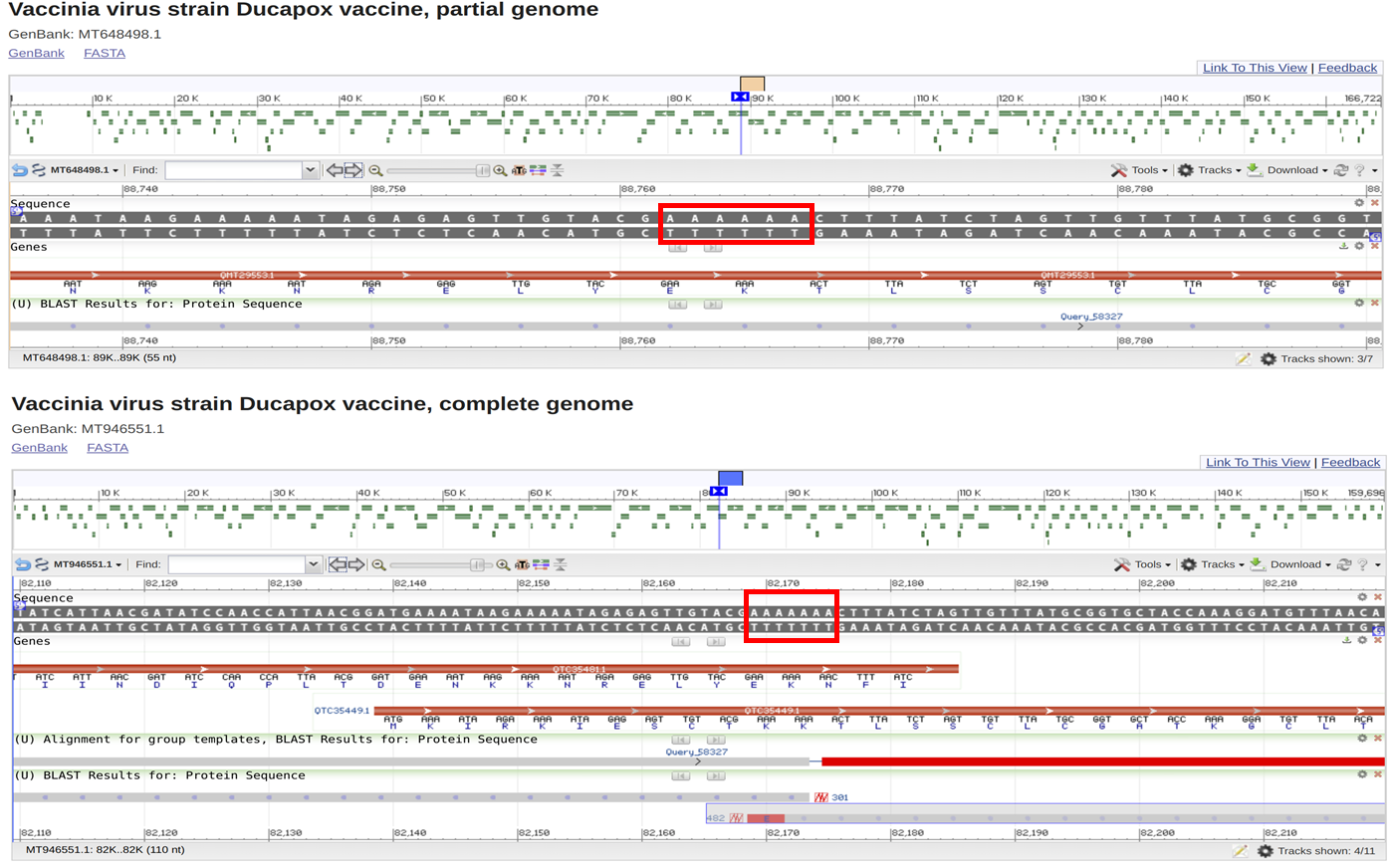

Supplement: Supplementary file 2 — Supplementary Information 2. [file 41598_2021_97158_MOESM2_ESM.docx]
