# Supplementary material for: Nanopore sequencing and de novo assembly of a misidentified Camelpox vaccine reveals putative epigenetic modifications and alternate protein signal peptides
Source: Sci Rep. 2021 Sep 7;11:17758. doi: 10.1038/s41598-021-97158-x (PMC8423768; doi:10.1038/s41598-021-97158-x)
Supplement: Supplementary file 3 — Supplementary Information 3. [file 41598_2021_97158_MOESM3_ESM.docx]

**Nanopore sequencing and *de novo* assembly of a misidentified Camelpox vaccine reveals putative epigenetic modifications and alternate protein signal peptides**

**Zack Saud^1^*, Matthew D. Hitchings^2^, Tariq M. Butt^1^**

*^1^ Department of Biosciences, College of Science, Swansea University, Singleton Park, Swansea, SA2 8PP, Wales, United Kingdom*

*^2^ Swansea University Medical School, Swansea University, Singleton Park, Swansea, Sa2 8PP, Wales, United Kingdom*

*** Corresponding author

* Z. Saud: [zack.saud@swansea.ac.uk](mailto:zack.saud@swansea.ac.uk)

**Supplementary Information 3- SignalP v5.0 output summary**

# SignalP-5.0 Organism: euk Timestamp: 20210725214845

# ID Prediction SP(Sec/SPI) OTHER CS Position

MT946551.1_1 OTHER 0.005333 0.994667

MT946551.1_2 OTHER 0.001733 0.998267

MT946551.1_3 OTHER 0.002231 0.997769

MT946551.1_5 OTHER 0.004228 0.995772

MT946551.1_6 OTHER 0.001072 0.998928

MT946551.1_7 OTHER 0.014762 0.985238

MT946551.1_8 OTHER 0.000621 0.999379

MT946551.1_9 OTHER 0.001058 0.998942

MT946551.1_10 OTHER 0.000514 0.999486

MT946551.1_11 OTHER 0.001276 0.998724

MT946551.1_12 SP(Sec/SPI) 0.970245 0.029755 CS pos: 19-20. GNC-HE. Pr: 0.8400

MT946551.1_13 OTHER 0.000669 0.999331

MT946551.1_15 OTHER 0.000782 0.999218

MT946551.1_16 OTHER 0.001182 0.998818

MT946551.1_17 OTHER 0.000600 0.999400

MT946551.1_18 OTHER 0.000738 0.999262

MT946551.1_19 SP(Sec/SPI) 0.701809 0.298191 CS pos: 13-14. CSA-ST. Pr: 0.3726

MT946551.1_20 OTHER 0.002402 0.997598

MT946551.1_21 OTHER 0.001077 0.998923

MT946551.1_22 OTHER 0.069371 0.930629

MT946551.1_23 OTHER 0.012827 0.987173

MT946551.1_24 OTHER 0.000369 0.999631

MT946551.1_25 OTHER 0.000855 0.999145

MT946551.1_26 OTHER 0.002162 0.997838

MT946551.1_27 OTHER 0.001029 0.998971

MT946551.1_28 OTHER 0.002058 0.997942

MT946551.1_29 OTHER 0.000180 0.999820

MT946551.1_30 OTHER 0.003846 0.996154

MT946551.1_31 OTHER 0.003395 0.996605

MT946551.1_32 OTHER 0.000903 0.999097

MT946551.1_33 OTHER 0.000549 0.999451

MT946551.1_34 OTHER 0.000830 0.999170

MT946551.1_36 OTHER 0.000525 0.999475

MT946551.1_37 OTHER 0.000696 0.999304

MT946551.1_38 OTHER 0.004508 0.995492

MT946551.1_39 OTHER 0.000666 0.999334

MT946551.1_41 OTHER 0.001270 0.998730

MT946551.1_42 OTHER 0.478891 0.521109

MT946551.1_43 OTHER 0.000897 0.999103

MT946551.1_44 OTHER 0.000226 0.999774

MT946551.1_45 OTHER 0.001790 0.998210

MT946551.1_46 OTHER 0.000610 0.999390

MT946551.1_47 OTHER 0.000477 0.999523

MT946551.1_48 OTHER 0.000351 0.999649

MT946551.1_49 OTHER 0.000650 0.999350

MT946551.1_50 OTHER 0.000322 0.999678

MT946551.1_51 OTHER 0.000584 0.999416

MT946551.1_52 OTHER 0.000246 0.999754

MT946551.1_53 SP(Sec/SPI) 0.952870 0.047130 CS pos: 25-26. LDG-NI. Pr: 0.3430

MT946551.1_54 OTHER 0.000962 0.999038

MT946551.1_55 OTHER 0.000537 0.999463

MT946551.1_56 OTHER 0.000740 0.999260

MT946551.1_57 OTHER 0.000335 0.999665

MT946551.1_58 OTHER 0.000509 0.999491

MT946551.1_59 OTHER 0.013660 0.986340

MT946551.1_60 OTHER 0.001590 0.998410

MT946551.1_61 OTHER 0.001588 0.998412

MT946551.1_62 OTHER 0.036052 0.963948

MT946551.1_63 OTHER 0.001954 0.998046

MT946551.1_64 OTHER 0.016068 0.983932

MT946551.1_65 OTHER 0.000953 0.999047

MT946551.1_66 OTHER 0.001253 0.998747

MT946551.1_67 OTHER 0.000443 0.999557

MT946551.1_68 OTHER 0.005461 0.994539

MT946551.1_69 OTHER 0.018252 0.981748

MT946551.1_70 OTHER 0.000331 0.999669

MT946551.1_71 OTHER 0.001449 0.998551

MT946551.1_72 OTHER 0.001482 0.998518

MT946551.1_73 OTHER 0.000933 0.999067

MT946551.1_74 OTHER 0.000847 0.999153

MT946551.1_75 OTHER 0.000882 0.999118

MT946551.1_76 OTHER 0.001814 0.998186

MT946551.1_77 OTHER 0.000103 0.999897

MT946551.1_78 OTHER 0.001609 0.998391

MT946551.1_79 OTHER 0.000679 0.999321

MT946551.1_80 OTHER 0.000084 0.999916

MT946551.1_81 OTHER 0.000909 0.999091

MT946551.1_82 OTHER 0.002711 0.997289

MT946551.1_83 OTHER 0.000440 0.999560

MT946551.1_84 OTHER 0.001068 0.998932

MT946551.1_85 OTHER 0.002841 0.997159

MT946551.1_86 OTHER 0.002403 0.997597

MT946551.1_87 OTHER 0.002645 0.997355

MT946551.1_88 OTHER 0.001149 0.998851

MT946551.1_89 OTHER 0.001771 0.998229

MT946551.1_90 OTHER 0.000453 0.999547

MT946551.1_91 OTHER 0.000430 0.999570

MT946551.1_92 OTHER 0.001486 0.998514

MT946551.1_93 OTHER 0.005226 0.994774

MT946551.1_94 OTHER 0.001334 0.998666

MT946551.1_95 OTHER 0.000657 0.999343

MT946551.1_96 OTHER 0.000281 0.999719

MT946551.1_97 OTHER 0.000836 0.999164

MT946551.1_98 OTHER 0.002721 0.997279

MT946551.1_99 OTHER 0.000565 0.999435

MT946551.1_100 OTHER 0.000799 0.999201

MT946551.1_101 OTHER 0.001696 0.998304

MT946551.1_102 OTHER 0.000321 0.999679

MT946551.1_103 OTHER 0.001089 0.998911

MT946551.1_104 OTHER 0.000406 0.999594

MT946551.1_105 OTHER 0.000733 0.999267

MT946551.1_106 OTHER 0.002584 0.997416

MT946551.1_107 OTHER 0.001484 0.998516

MT946551.1_108 OTHER 0.001175 0.998825

MT946551.1_109 OTHER 0.000278 0.999722

MT946551.1_110 OTHER 0.000644 0.999356

MT946551.1_111 OTHER 0.000710 0.999290

MT946551.1_112 OTHER 0.000256 0.999744

MT946551.1_113 OTHER 0.000615 0.999385

MT946551.1_114 OTHER 0.013384 0.986616

MT946551.1_115 OTHER 0.000523 0.999477

MT946551.1_116 OTHER 0.028723 0.971277

MT946551.1_117 OTHER 0.000570 0.999430

MT946551.1_118 OTHER 0.000634 0.999366

MT946551.1_119 OTHER 0.001223 0.998777

MT946551.1_120 OTHER 0.185160 0.814840

MT946551.1_121 OTHER 0.000820 0.999180

MT946551.1_122 OTHER 0.035769 0.964231

MT946551.1_123 OTHER 0.001750 0.998250

MT946551.1_124 OTHER 0.001918 0.998082

MT946551.1_125 OTHER 0.001960 0.998040

MT946551.1_126 OTHER 0.001337 0.998663

MT946551.1_127 OTHER 0.000949 0.999051

MT946551.1_128 OTHER 0.001017 0.998983

MT946551.1_129 OTHER 0.000736 0.999264

MT946551.1_130 OTHER 0.000823 0.999177

MT946551.1_131 OTHER 0.000455 0.999545

MT946551.1_132 OTHER 0.000276 0.999724

MT946551.1_133 OTHER 0.000802 0.999198

MT946551.1_134 OTHER 0.000239 0.999761

MT946551.1_135 SP(Sec/SPI) 0.590206 0.409794 CS pos: 23-24. IQG-YS. Pr: 0.3193

MT946551.1_136 OTHER 0.000652 0.999348

MT946551.1_137 OTHER 0.000231 0.999769

MT946551.1_138 OTHER 0.002570 0.997430

MT946551.1_139 OTHER 0.000558 0.999442

MT946551.1_140 OTHER 0.001727 0.998273

MT946551.1_141 OTHER 0.000518 0.999482

MT946551.1_142 OTHER 0.273429 0.726571

MT946551.1_143 OTHER 0.013187 0.986813

MT946551.1_144 OTHER 0.018782 0.981218

MT946551.1_145 OTHER 0.001293 0.998707

MT946551.1_146 OTHER 0.022109 0.977891

MT946551.1_147 SP(Sec/SPI) 0.851345 0.148655 CS pos: 22-23. TKT-IE. Pr: 0.4898

MT946551.1_148 SP(Sec/SPI) 0.768902 0.231098 CS pos: 14-15. ANG-IE. Pr: 0.5814

MT946551.1_149 OTHER 0.001079 0.998921

MT946551.1_150 OTHER 0.021459 0.978541

MT946551.1_151 OTHER 0.001799 0.998201

MT946551.1_152 OTHER 0.000348 0.999652

MT946551.1_153 SP(Sec/SPI) 0.986466 0.013534 CS pos: 20-21. VLA-YS. Pr: 0.6823

MT946551.1_154 OTHER 0.000248 0.999752

MT946551.1_155 OTHER 0.008044 0.991956

MT946551.1_156 OTHER 0.001371 0.998629

MT946551.1_157 OTHER 0.000507 0.999493

MT946551.1_158 OTHER 0.000205 0.999795

MT946551.1_159 OTHER 0.002476 0.997524

MT946551.1_160 OTHER 0.000494 0.999506

MT946551.1_161 OTHER 0.000487 0.999513

MT946551.1_162 OTHER 0.014678 0.985322

MT946551.1_163 SP(Sec/SPI) 0.997751 0.002249 CS pos: 16-17. VYA-TP. Pr: 0.7838

MT946551.1_165 OTHER 0.000285 0.999715

MT946551.1_166 OTHER 0.001957 0.998043

MT946551.1_167 OTHER 0.001506 0.998494

MT946551.1_168 OTHER 0.000708 0.999292

MT946551.1_169 OTHER 0.002042 0.997958

MT946551.1_170 SP(Sec/SPI) 0.998684 0.001316 CS pos: 19-20. VYS-TC. Pr: 0.8980

MT946551.1_171 OTHER 0.000359 0.999641

MT946551.1_172 SP(Sec/SPI) 0.991470 0.008530 CS pos: 19-20. SYS-NN. Pr: 0.4641

MT946551.1_173 SP(Sec/SPI) 0.998541 0.001459 CS pos: 17-18. IHA-KI. Pr: 0.9731

MT946551.1_174 OTHER 0.000508 0.999492

MT946551.1_175 OTHER 0.000208 0.999792

MT946551.1_176 OTHER 0.000337 0.999663

MT946551.1_177 OTHER 0.084984 0.915016

MT946551.1_178 OTHER 0.071366 0.928634

MT946551.1_179 OTHER 0.000827 0.999173

MT946551.1_180 SP(Sec/SPI) 0.972533 0.027467 CS pos: 20-21. VQT-FN. Pr: 0.7526

MT946551.1_181 OTHER 0.000376 0.999624

MT946551.1_182 OTHER 0.001899 0.998101

MT946551.1_183 SP(Sec/SPI) 0.985282 0.014718 CS pos: 25-26. SYA-ID. Pr: 0.9651

MT946551.1_185 OTHER 0.085628 0.914372

MT946551.1_187 OTHER 0.000998 0.999002

MT946551.1_188 OTHER 0.001293 0.998707

MT946551.1_189 OTHER 0.001072 0.998928

MT946551.1_190 OTHER 0.004228 0.995772

MT946551.1_192 OTHER 0.002231 0.997769

MT946551.1_193 OTHER 0.001733 0.998267

MT946551.1_194 OTHER 0.005333 0.994667
