# Supplementary material for: Nanopore sequencing and de novo assembly of a misidentified Camelpox vaccine reveals putative epigenetic modifications and alternate protein signal peptides
Source: Sci Rep. 2021 Sep 7;11:17758. doi: 10.1038/s41598-021-97158-x (PMC8423768; doi:10.1038/s41598-021-97158-x)
Supplement: Supplementary file 4 — Supplementary Information 4. [file 41598_2021_97158_MOESM4_ESM.docx]

**Nanopore sequencing and *de novo* assembly of a misidentified Camelpox vaccine reveals putative epigenetic modifications and alternate protein signal peptides**

**Zack Saud^1^*, Matthew D. Hitchings^2^, Tariq M. Butt^1^**

*^1^ Department of Biosciences, College of Science, Swansea University, Singleton Park, Swansea, SA2 8PP, Wales, United Kingdom*

*^2^ Swansea University Medical School, Swansea University, Singleton Park, Swansea, Sa2 8PP, Wales, United Kingdom*

*** Corresponding author

* Z. Saud: [zack.saud@swansea.ac.uk](mailto:zack.saud@swansea.ac.uk)

**Supplementary Information 4- List of mature proteins with the SignalP 5.0 predicted signal peptides removed**

>A38L

IEYTACNDTIIIPCTIDNPTKYIRWKLDNHDILTYNKTSKTTILSKWHTSARLHSLSDSDVSLIMEYKDILPGTYTCGDNTGIKSTVKLVQLHTNWFNDYQTMLMFIFTGITLFLLFLEITYTSISVVFSTNLGILQVFGCVIAMIELCGAFLFYPSMFTLRHIIGLLMMTLPSIFLIITKVFSFWLLCKSSCAVHLIIYYQLAGYILTVLGLGLSLKECVDGTLLLSGLGTIMVSEHFSLLFLVCFPSTQRDYY*

>C8L

HEDPYYQPFDKLNITLDIYTYEDLVPYTVDNDTTSFVKIYFKNFWITVMTKWCAPFIDTVSVYTSHDNLNIQFYSRDEYDTQSEDKICTIDVKARCKHLTKREVTVQQEAYRYSLSSDLSCFDSIDLEIDLIETNSTDTTVLKSYELMLPKRAKSIHN*

>HA

TPFPQTSKKIGDDATLSCNRNNTNDYVVMSAWYKEPNSIILLAAKSDVLYFDNYTKDKISYDSPYDDLVTTITIKSLTARDAGTYVCAFFMTSPTNDTDKVDYEEYSTELIVNTDSESTIDIILSGSTHSPETSSEKPDYIDNSNCSSVFEIATPEPITDNVEDHTDTVTYTSDSINTVSASSGESTTDETPEPITDKEEDHTVTDTVSYTTVSTSSGIVTTKSTTDDADLYDTYNDNDTVPSTTVGGSTTSISNYKTKDFVEIFGITALIILSAVAIFCITYYIYNKRSRKYKTENKV*

>B19R

IDIENEITEFFNKMRDTLPAKDSKWLNPACMFGGTMNDIAALGEPFSAKCPPIEDSLLSHRYKDYVVKWERLEKNRRRQVSNKRVKHGDLWIANYTSKFSNRRYLCTVTTKNGDCVQGIVRSHIKKPPSCIPKTYELGTHDKYGIDLYCGILYAKHYNNITWYKDNKEINIDDIKYSQTGKKLIIHNPELEDSGRYNCYVHYDDVRIKM*

>E10R

NIEACKRKLYTIVSTLPCPACRRHATIAIEDNNVMSSDDLNYIYYFFIRLFNNLASDPKYAIDVTKVNPL*

>B8R

KITSYKFESVNFDSKIEWTGDGLYNISLKNYGIKTWQTMYTNVPEGTYDISAFPKNDFVSFWVKFEQGDYKVEEYCTGPPTVTLTEYDDHPYATRGSKKIPIYKRGDMCDIYLLYTANFTFGDSKEPVPYDIDDYDCTSTGCSIDFVTTEKVCVTAQGATEGFLEKITPWSSKVCLTPKKSVYTCAIRSKEDVPNFKDKMARVIKRKFN*

>B7R

NNEYTPFNKLSVKLYIDGVDNIENSYTDDNNELVLNFKEYTISIITESCDVGFDSIDIDVINDYKIIDMSTIQRRGHTCRISTKLSCHYDKYPYIHKYDGDERQYSITAEGKCYKGIKYEISMINDDTLLRKHTLKIGSTYIFDRHGHSNTYYSKYDF*

>A28L

YSIYENYGNIKEFNATHAAFEYSKSIGGTPALDRRVQDVNDTISDVKQKWRCVVYPGNGFVSASIFGFQAEVGPNNTRSIRKFNTMQQCIDFTFSDVININIYNPCVVPNINNAECQFLKSVL*

>B16R

FNAPECIDKGQYFASFMELENEPVILPCPQINTLSSGYNILDILWEKRGADNDRIIPIDNGSNMLILNPTQSDSGIYICITTNETYCDMMSLNLTIVSVSESNIDLISYPQIVNERSTGEMVCPNINAFIASNVNADIIWSGHRRLRNKRLKQRTPGIITIEDVRKNDAGYYTCVLEYIYGGKTYNVTRIVKLEVRDKIIPSTMQLPEGVVTSIGSNLTIACRVSLRPPTTDADVFWISNGMYYEEDDGDGDGRISVANKIYMTDKRRVITSRLNINPVKEEDATTFTCMAFTIPSISKTVTVSIT*

>SPI-3

STYRLQGFTNAGIVAYKNIQDDNIVFSPFGYSFSMFMSLLPASGNTRIELLKTMDLRKRDLGPAFTELISGLAKLKTSKYTYTDLTYQSFVDNTVCIKPSYYQQYHRFGLYRLNFRRDAVNKINSIVERRSGMSNVVDSNMLDNNTLWAIINTIYFKGIWQYPFDITKTRNASFTNKYGTKTVPMMNVVTKLQGNTITIDDKEYDMVRLPYKDANISMYLAIGDNMTHFTDSITAAKLDYWSFQLGNKVYNLKLPKFSIENKRDIKSIAEMMAPSMFNPDNASFKHMTRDPLYIYKMFQNAKIDVDEQGTVAEASTIMVATARSSPEKLEFNTPFVFIIRHDITGFILFMGKVESP*

>A39

IEWHKFETSEEIISTYLLDDVLYTGVNGAVYTFSNNKLNKTGLTNNNYITTSIKVEDAEPITEIPNVGK*

>PS/HR

TCTVPTMNNAKLTSTETSFNNNQKVTFTCDQGYHSSDPNAVCETDKWKYENPCKKMCTVSDYISELYNKPLYEVNSTMTLSCNGETKYFRCEEKNGNTSWNDTVTCPNAECQPLQLEHGSCQPVKEKYSFGEYITINCDVGYEVIGASYISCTANSWNVIPSCQQKCDIPSLSNGLISGSTFSIGGVIHLSCKSGFILTGSPSSTCIDGKWNPILPTCVRSNEKFDPVDDGPDDETDLSKLSKDVVQYEQEIESLEATYHIIIVALTIMGVIFLISVIVLVCSCDKNNDQY*

>A43R

YSSSIFRFHSEDVELCYGHLYFDRIYNVVNIKYNPHIPYRYNFINRTLTVDELDDNVFFTHGYFLKHKYGSLNPSLIVSLSGNLKYNDIQCSVNVSCLIKNLATSTSTILTSKHKTYSLHRSTCITIIGYDSIIWYKDINDIYDFTAICMLIASTLIVTIYVFKKIKMNS*
