# Supplementary material for: Nanopore sequencing and de novo assembly of a misidentified Camelpox vaccine reveals putative epigenetic modifications and alternate protein signal peptides
Source: Sci Rep. 2021 Sep 7;11:17758. doi: 10.1038/s41598-021-97158-x (PMC8423768; doi:10.1038/s41598-021-97158-x)
Supplement: Supplementary file 5 — Supplementary Information 5. [file 41598_2021_97158_MOESM5_ESM.docx]

**Nanopore sequencing and *de novo* assembly of a misidentified Camelpox vaccine reveals putative epigenetic modifications and alternate protein signal peptides**

**Zack Saud^1^*, Matthew D. Hitchings^2^, Tariq M. Butt^1^**

*^1^ Department of Biosciences, College of Science, Swansea University, Singleton Park, Swansea, SA2 8PP, Wales, United Kingdom*

*^2^ Swansea University Medical School, Swansea University, Singleton Park, Swansea, Sa2 8PP, Wales, United Kingdom*

*** Corresponding author

* Z. Saud: [zack.saud@swansea.ac.uk](mailto:zack.saud@swansea.ac.uk)

**Supplementary Information 5- Predicted protein comparison of Ducapox long-read and short-read assemblies**

1. **Comparison of proteins that were found to differ between the two assemblies**

| **Ducapox Long Read Assembly Protein ID** | **Closest matching Ducapox Short Read Assembly Protein ID** | **Match** | **Long read Assembly Top UniProt hit** | **Short Read Assembly Top Uniprot hit** |
| --- | --- | --- | --- | --- |
| [product=Protein F6] [length=82] | No hit | - | 100% identity, 82% query coverage to P68601.1 [8AA longer] | - |
| [product=PAPL] [length=479] | QMT29502.1 [length=479] | 99.79% identity, 100% query coverage, X (any) instead of R at position 342 | 100% identity, 100% query coverage to O57184.1 | 99.79% identity, 100% query coverage to O57184.1 |
| [product=I5L] [length=79] | QMT29517.1 [length=79] | 98.73% identity, 100% query coverage, X (any) instead of A at position 25 | 100% identity, 100% query coverage to P20500.1 | 98.73% identity, 100% query coverage to P20500.1 |
| [product=I6L] [length=393] | QMT29518.1 [length=382] | 99.18% identity, 92% query coverage | 99.18% identity, 92% query coverage to P68462.1 [11AA longer] | 100% identity, 100% query coverage to P68462.1 |
| [product=NPH2] [length=676] | QMT29520.1 [length=676] | 99.85% identity, 100% query coverage, X (any) instead of F at position 11 | 100% identity, 100% query coverage to O57193.1 | 99.85% identity, 100% query coverage to O57193.1 |
| [product=RPO147] [length=1286] | QMT29541.1 [length=1286] | 99.92% identity, 100% query coverage, B (Aspartic Acid or Asparagine) instead of D at position 962 | 99.92% identity, 100% query coverage to O57204.1 | 99.92% identity, 100% query coverage to O57204.1 |
| [product=RPO18] [length=161] | QMT29555 [length=161] | 99.38% identity, 100% query coverage, X (any) instead of A at position 130 | 99.38% identity, 100% query coverage to P04310.1, (T as oppose to P at position 16) | 98.76% identity, 100% query coverage to P04310.1, (T as oppose to P at position 16) |
| [product=RPO132] [length=1156] | QMT29587 [length=1156] | 99.83% identity, 100% query coverage, J (Leucine or Isoleucine) instead of L at position 399 | 100% identity, 100% query coverage to O57230.1 | 100% identity, 99.83% query coverage to O57230.1 |
| [product=A43R] [length=190] | QMT29609.1 [length=187] | 100% identity, 98% query coverage | 97.94% identity, 100% query coverage to P26671.1 (4AA deletion) | 97.91% identity, 100% query coverage to P26671.1 (3AA shorter and 4 AA deletion) |
| [product=B17L] [length=340] | QMT29638.1 [length=340] | 99.71% identity, 100% query coverage, X (any) instead of A at position 223 | 98.53% identity, 100% query coverage to P21075.1 | 98.24% identity, 100% query coverage to P21075.1 |

1. **Evaluation of predicted proteins from short-assembly that were absent from long-read assembly**

| **Short read assembly protein ID** | **Top UniProt hit** | **Notes** |
| --- | --- | --- |
| QMT29459.1 | No hit |  |
| QMT29463.1 | No hit |  |
| QMT29470.1 | No hit |  |
| QMT29490.1 | No hit |  |
| QMT29495.1 | No hit |  |
| QMT29588.1 | No hit |  |
| QMT29620.1 | No hit |  |
| QMT29641.1 | No hit |  |
| QMT29643.1 | No hit |  |
| QMT29648.1 | No hit |  |
