# Supplementary material for: Nanopore sequencing and de novo assembly of a misidentified Camelpox vaccine reveals putative epigenetic modifications and alternate protein signal peptides
Source: Sci Rep. 2021 Sep 7;11:17758. doi: 10.1038/s41598-021-97158-x (PMC8423768; doi:10.1038/s41598-021-97158-x)
Supplement: Supplementary file 6 — Supplementary Information 6. [file 41598_2021_97158_MOESM6_ESM.docx]

**Nanopore sequencing and *de novo* assembly of a misidentified Camelpox vaccine reveals putative epigenetic modifications and alternate protein signal peptides**

**Zack Saud^1^*, Matthew D. Hitchings^2^, Tariq M. Butt^1^**

*^1^ Department of Biosciences, College of Science, Swansea University, Singleton Park, Swansea, SA2 8PP, Wales, United Kingdom*

*^2^ Swansea University Medical School, Swansea University, Singleton Park, Swansea, Sa2 8PP, Wales, United Kingdom*

*** Corresponding author

* Z. Saud: [zack.saud@swansea.ac.uk](mailto:zack.saud@swansea.ac.uk)

**Supplementary 6- Nanopolish modified base-calling output**

chromosome start end num_motifs_in_group called_sites called_sites_methylated methylated_frequency group_sequence

MT946551.1 33366 33366 1 59 28 0.475 TTTCACGGAGG

MT946551.1 99927 99927 1 32 14 0.438 AATTCCGGAAT

MT946551.1 37861 37861 1 78 31 0.397 CTTTCCGAAAG

MT946551.1 148486 148486 1 63 25 0.397 ACCAACGGAAA

MT946551.1 138221 138221 1 63 24 0.381 ATTTACGGAAC

MT946551.1 106197 106197 1 69 26 0.377 CATTCCGACTG

MT946551.1 96263 96263 1 48 17 0.354 GTTGTCGTGGT

MT946551.1 18813 18813 1 49 17 0.347 TTTTCCGGAAG

MT946551.1 100931 100931 1 49 16 0.327 ATATCCGTCAA

MT946551.1 17144 17144 1 62 20 0.323 ATTTCCGAATG

MT946551.1 56806 56806 1 44 14 0.318 TTGGCCGGAAA

MT946551.1 112865 112865 1 61 19 0.311 TTTTCCGGTAA

MT946551.1 54452 54452 1 58 18 0.31 ATACTCGGATT

MT946551.1 26658 26658 1 52 16 0.308 AGATCCGATAA

MT946551.1 115907 115907 1 70 21 0.3 AATTCCGTTGG

MT946551.1 144724 144724 1 77 23 0.299 CACTCCGTTTA

MT946551.1 126767 126767 1 86 25 0.291 AATTCCGTATA

MT946551.1 154227 154227 1 55 16 0.291 ACAAACGGGAA

MT946551.1 26465 26465 1 56 16 0.286 AGTTCCGTTTA

MT946551.1 125200 125200 1 95 27 0.284 CTGTACGGAAT

MT946551.1 35581 35581 1 75 21 0.28 ATTCCCGTATA

MT946551.1 3390 3390 1 47 13 0.277 TGTATCGCATT

MT946551.1 64908 64908 1 50 13 0.26 TACTCCGGTTG

MT946551.1 135034 135034 1 96 25 0.26 AAATACGATGG

MT946551.1 30569 30569 1 47 12 0.255 TTAGCCGGAAT

MT946551.1 42047 42047 1 76 19 0.25 TCATACGCTCC

MT946551.1 150615 150615 1 32 8 0.25 TATAACGTGAA

MT946551.1 73773 73773 1 69 16 0.232 TTTGTCGTAAA

MT946551.1 68455 68455 1 61 14 0.23 CTTTACGGTAT

MT946551.1 81293 81293 1 61 14 0.23 AATTGCGACAC

MT946551.1 67460 67460 1 66 15 0.227 TTATCCGATCA

MT946551.1 76130 76130 1 58 13 0.224 CACTCCGATAT

MT946551.1 25168 25168 1 63 14 0.222 ATACACGTTAA

MT946551.1 61987 61987 1 45 10 0.222 TTTTCCGTCAA

MT946551.1 149420 149420 1 54 12 0.222 ATCATCGGACT

MT946551.1 80566 80566 1 50 11 0.22 ATAGTCGGATA

MT946551.1 99143 99143 1 50 11 0.22 CTCATCGGATC

MT946551.1 158780 158780 1 32 7 0.219 TTTCTCGTAAA

MT946551.1 67040 67040 1 51 11 0.216 TCTCCCGGAAT

MT946551.1 124661 124661 1 74 16 0.216 TGTGTCGTAGT

MT946551.1 130487 130487 1 56 12 0.214 GATCCCGGATA

MT946551.1 43210 43210 1 53 11 0.208 ATGCACGGGCA

MT946551.1 65986 65986 1 59 12 0.203 GAAAACGGAAT

MT946551.1 40668 40668 1 65 13 0.2 TAAGACGGAAA

MT946551.1 50600 50600 1 70 14 0.2 GCATCCGTTGC

MT946551.1 109339 109339 1 45 9 0.2 TTATCCGATCT

MT946551.1 119739 119739 1 61 12 0.197 AAATCCGGATC

MT946551.1 62333 62333 1 51 10 0.196 TATTCCGACAA

MT946551.1 16334 16334 1 62 12 0.194 TTTGACGTAAA

MT946551.1 107178 107178 1 36 7 0.194 ATCTCCGGAAT

MT946551.1 154870 154870 1 36 7 0.194 ATCAACGGAAC

MT946551.1 48063 48063 1 57 11 0.193 TCTGACGGAGA

MT946551.1 44520 44520 1 63 12 0.19 CCATCCGTAAA

MT946551.1 82046 82046 1 58 11 0.19 TCAACCGGATT

MT946551.1 142767 142767 1 58 11 0.19 TCTTTCGTGAA

MT946551.1 73335 73335 1 74 14 0.189 GATGACGATCC

MT946551.1 97443 97443 1 53 10 0.189 GATGTCGTTAT

MT946551.1 149398 149398 1 48 9 0.188 TATACCGGATG

MT946551.1 141813 141813 1 75 14 0.187 AGTTACGGTTG

MT946551.1 20891 20891 1 70 13 0.186 TGGACCGAATT

MT946551.1 64581 64581 1 70 13 0.186 AGGACCGTGTA

MT946551.1 85149 85149 1 70 13 0.186 AATGCCGTATA

MT946551.1 91846 91846 1 70 13 0.186 TCAAACGGAGT

MT946551.1 138400 138400 1 70 13 0.186 TGAAGCGGATG

MT946551.1 89441 89441 1 92 17 0.185 TGGAGCGTATC

MT946551.1 47961 47961 1 49 9 0.184 TCCTCCGGAGT

MT946551.1 68002 68002 1 49 9 0.184 ATTAACGGGTT

MT946551.1 105132 105132 1 60 11 0.183 CAGTCCGGTTC

MT946551.1 25112 25112 1 55 10 0.182 CTCCACGATTA

MT946551.1 127013 127013 1 99 18 0.182 TTCTACGTCAA

MT946551.1 16628 16628 1 50 9 0.18 TCAAACGGATT

MT946551.1 78831 78831 1 62 11 0.177 TAGACCGTCTA

MT946551.1 84479 84479 1 68 12 0.176 TTGGACGATCT

MT946551.1 155885 155885 1 34 6 0.176 ACTTCCGTGGA

MT946551.1 1541 1541 1 40 7 0.175 ATGATCGGGAG

MT946551.1 24897 24897 1 63 11 0.175 AGACACGAATA

MT946551.1 122711 122720 2 114 20 0.175 AGTGTCGTAAATTACGAGCC

MT946551.1 627 627 1 23 4 0.174 CACCTCGGGAT

MT946551.1 7771 7771 1 46 8 0.174 ATCTACGACAA

MT946551.1 18674 18674 1 69 12 0.174 TTCTCCGATAT

MT946551.1 40230 40230 1 69 12 0.174 GTGTCCGTATA

MT946551.1 62181 62181 1 46 8 0.174 ATTTACGGATT

MT946551.1 143606 143606 1 69 12 0.174 TATACCGTCTC

MT946551.1 143631 143631 1 46 8 0.174 ATTTCCGGATC

MT946551.1 81961 81961 1 52 9 0.173 ACTTCCGTTTA

MT946551.1 33606 33606 1 29 5 0.172 TTTCCCGGAGA

MT946551.1 29620 29620 1 70 12 0.171 TATTCCGTATC

MT946551.1 39626 39626 1 53 9 0.17 GGGTTCGTCAA

MT946551.1 153832 153832 1 47 8 0.17 TTCAGCGCAAA

MT946551.1 11515 11515 1 65 11 0.169 GAATACGGAAT

MT946551.1 39218 39218 1 65 11 0.169 AGAGCCGTATG

MT946551.1 114528 114528 1 89 15 0.169 ACACCCGATCA

MT946551.1 131000 131000 1 83 14 0.169 TCTAGCGCTTC

MT946551.1 84785 84785 1 95 16 0.168 ATTCTCGTATT

MT946551.1 1909 1909 1 42 7 0.167 CTTTGCGTATC

MT946551.1 99501 99501 1 60 10 0.167 GGAGTCGCCAA

MT946551.1 75156 75156 1 61 10 0.164 TTTTACGGAAA

MT946551.1 87641 87641 1 55 9 0.164 TTTGTCGGAAG

MT946551.1 92812 92812 1 67 11 0.164 AATTACGGAAC

MT946551.1 118033 118033 1 73 12 0.164 TTGACCGTGTA

MT946551.1 56698 56698 1 43 7 0.163 TTTCCCGTCAA

MT946551.1 100498 100498 1 56 9 0.161 AATAGCGGTTT

MT946551.1 116384 116384 1 87 14 0.161 TAAGCCGTACA

MT946551.1 50179 50179 1 50 8 0.16 TATACCGGAAG

MT946551.1 92365 92365 1 75 12 0.16 AACTCCGTAGA

MT946551.1 136651 136651 1 69 11 0.159 AAGTCCGACTA

MT946551.1 30765 30765 1 89 14 0.157 CTTCTCGCTTC

MT946551.1 47706 47706 1 83 13 0.157 AGTACCGATAG

MT946551.1 53717 53717 1 51 8 0.157 TTTATCGGAGG

MT946551.1 148969 148969 1 51 8 0.157 TCATCCGTTCA

MT946551.1 125674 125674 1 64 10 0.156 ATTGACGGATA

MT946551.1 145316 145316 1 45 7 0.156 GAATCCGTCAA

MT946551.1 12978 12978 1 72 11 0.153 ATTATCGTAAG

MT946551.1 82874 82874 1 59 9 0.153 AATGACGGACC

MT946551.1 83239 83239 1 72 11 0.153 AGAAGCGTGTT

MT946551.1 103899 103899 1 59 9 0.153 GTTTCCGAACT

MT946551.1 123191 123191 1 72 11 0.153 TATTCCGACAT

MT946551.1 50377 50377 1 66 10 0.152 TGCCACGTTAG

MT946551.1 71857 71857 1 92 14 0.152 AAATGCGTATT

MT946551.1 82790 82790 1 46 7 0.152 TACACCGGAAG

MT946551.1 139930 139930 1 66 10 0.152 AGATCCGATCC

MT946551.1 64000 64000 1 73 11 0.151 ATATACGTTAT

MT946551.1 17248 17248 1 60 9 0.15 TATTCCGTCAC

MT946551.1 58834 58834 1 80 12 0.15 AAATGCGATGT

MT946551.1 139324 139324 1 60 9 0.15 AGATTCGCATA

MT946551.1 132847 132847 1 67 10 0.149 TAGTCCGTTTG

MT946551.1 36012 36012 1 61 9 0.148 TGATCCGGTAT

MT946551.1 91056 91056 1 61 9 0.148 GGGATCGTTAA

MT946551.1 61003 61003 1 95 14 0.147 ATGTTCGTTCT

MT946551.1 45634 45634 1 76 11 0.145 TCATCCGGACA

MT946551.1 45657 45657 1 62 9 0.145 CTCTCCGTCAT

MT946551.1 53594 53594 1 69 10 0.145 GGAATCGTTGG

MT946551.1 108961 108961 1 62 9 0.145 ACACACGGTAG

MT946551.1 421 421 1 21 3 0.143 AGACCCGAGTA

MT946551.1 1608 1608 1 28 4 0.143 AGAAACGTGAC

MT946551.1 10302 10302 1 49 7 0.143 TCAAACGGATA

MT946551.1 60639 60639 1 63 9 0.143 ATTATCGCTAA

MT946551.1 96049 96049 1 56 8 0.143 CCATCCGATTG

MT946551.1 104506 104506 1 63 9 0.143 ATTGACGATAC

MT946551.1 104951 104951 1 70 10 0.143 GAAAACGAAAA

MT946551.1 105370 105370 1 63 9 0.143 TTCCACGAACT

MT946551.1 24522 24522 1 71 10 0.141 ATCAACGATTC

MT946551.1 47541 47541 1 64 9 0.141 TCTTTCGGACT

MT946551.1 73489 73489 1 64 9 0.141 TAATCCGTCTA

MT946551.1 74409 74409 1 78 11 0.141 AATAGCGCTGA

MT946551.1 105887 105887 1 64 9 0.141 AAATTCGGACT

MT946551.1 144005 144005 1 64 9 0.141 AAATCCGTTAA

MT946551.1 40318 40318 1 57 8 0.14 GTCAACGGACC

MT946551.1 92208 92208 1 57 8 0.14 AACTCCGGATA

MT946551.1 137472 137472 1 57 8 0.14 TTCACCGGAAA

MT946551.1 157028 157028 1 57 8 0.14 ATCATCGATAA

MT946551.1 142889 142889 1 72 10 0.139 ATGGTCGTGTT

MT946551.1 63418 63418 1 80 11 0.138 GGTATCGATGA

MT946551.1 77664 77664 1 80 11 0.138 AGAATCGTCTA

MT946551.1 53465 53465 1 51 7 0.137 TTGGTCGGATA

MT946551.1 94556 94556 1 73 10 0.137 GATGCCGTTTA

MT946551.1 17636 17636 1 66 9 0.136 TCAACCGGTAA

MT946551.1 67749 67749 1 59 8 0.136 ATATCCGAGAG

MT946551.1 99978 99978 1 89 12 0.135 TCACACGCTCC

MT946551.1 109477 109477 1 74 10 0.135 GTAGACGAGCC

MT946551.1 122102 122102 1 74 10 0.135 GCTAACGAGGC

MT946551.1 138264 138264 1 74 10 0.135 ATAATCGTATT

MT946551.1 82353 82353 1 67 9 0.134 TTTATCGCTAA

MT946551.1 108235 108235 1 67 9 0.134 CTCCACGATAC

MT946551.1 127046 127046 1 82 11 0.134 ATTGCCGTCTC

MT946551.1 463 463 1 30 4 0.133 TTCTTCGCTAC

MT946551.1 58341 58341 1 75 10 0.133 AGAATCGTAAT

MT946551.1 81992 81992 1 60 8 0.133 GTAGACGGAAT

MT946551.1 98477 98477 1 75 10 0.133 TCCATCGGTTG

MT946551.1 126539 126539 1 75 10 0.133 AGTAACGATAA

MT946551.1 159479 159479 1 30 4 0.133 CAAAGCGTGTG

MT946551.1 16136 16136 1 76 10 0.132 AATCTCGTAGG

MT946551.1 24187 24187 1 38 5 0.132 ATATCCGGGCA

MT946551.1 98720 98720 1 76 10 0.132 ATCCTCGTTTA

MT946551.1 100441 100441 1 68 9 0.132 GCCTCCGTAGA

MT946551.1 109033 109033 1 68 9 0.132 GCAGTCGGATT

MT946551.1 133187 133187 1 68 9 0.132 TGTCTCGTGGG

MT946551.1 86092 86092 1 61 8 0.131 ATCATCGTGTT

MT946551.1 100852 100852 1 61 8 0.131 TATCACGTTTA

MT946551.1 150055 150055 1 61 8 0.131 ACTGGCGAAAT

MT946551.1 4140 4140 1 46 6 0.13 TCCACCGCCAC

MT946551.1 120188 120188 1 69 9 0.13 TTTTACGAGAT

MT946551.1 507 507 1 31 4 0.129 ATCAGCGGATT

MT946551.1 66235 66235 1 62 8 0.129 GCAACCGTATC

MT946551.1 75875 75875 1 62 8 0.129 ATCTACGGATG

MT946551.1 127859 127859 1 62 8 0.129 AAAAACGGAAA

MT946551.1 136300 136300 1 70 9 0.129 GATGACGATGA

MT946551.1 2432 2432 1 39 5 0.128 ATAGACGGCCA

MT946551.1 74444 74444 1 39 5 0.128 TTTCCCGGAAG

MT946551.1 76353 76353 1 78 10 0.128 AAAGACGCTTG

MT946551.1 13897 13897 1 55 7 0.127 ATATCCGTGTT

MT946551.1 75411 75411 1 55 7 0.127 AGTCCCGATGA

MT946551.1 80080 80080 1 63 8 0.127 TATCACGATGA

MT946551.1 209 209 1 32 4 0.125 ACACACGCTTT

MT946551.1 478 478 1 40 5 0.125 GATGTCGATAG

MT946551.1 49915 49915 1 56 7 0.125 TTCAGCGTGGA

MT946551.1 52137 52137 1 48 6 0.125 ACTATCGGATG

MT946551.1 67366 67366 1 40 5 0.125 TTATCCGGGTC

MT946551.1 75020 75020 1 88 11 0.125 TCCAGCGTATG

MT946551.1 75677 75677 1 48 6 0.125 TCTTCCGTCAC

MT946551.1 103152 103152 1 72 9 0.125 TCCTTCGCCAA

MT946551.1 109101 109101 1 48 6 0.125 AAGTCCGGATG

MT946551.1 126966 126966 1 72 9 0.125 AAGTCCGCATT

MT946551.1 154923 154923 1 24 3 0.125 TTCCACGGAAG

MT946551.1 156173 156173 1 48 6 0.125 TATGTCGTCTA

MT946551.1 159061 159061 1 16 2 0.125 GATCCCGAGGT

MT946551.1 159553 159553 1 24 3 0.125 CACCACGAATT

MT946551.1 51054 51054 1 97 12 0.124 TATGGCGTACA

MT946551.1 44757 44757 1 73 9 0.123 TCTCTCGGTTA

MT946551.1 47022 47022 1 65 8 0.123 ATTGACGAAAC

MT946551.1 55946 55946 1 65 8 0.123 GTACTCGTCAG

MT946551.1 73820 73820 1 73 9 0.123 TTATACGTCAT

MT946551.1 85394 85394 1 57 7 0.123 AGATTCGGGTA

MT946551.1 85757 85757 1 81 10 0.123 TTATACGGATT

MT946551.1 87875 87875 1 81 10 0.123 ACCATCGTGTA

MT946551.1 118910 118910 1 65 8 0.123 AGTTCCGTCCA

MT946551.1 144706 144706 1 73 9 0.123 CTATTCGAATA

MT946551.1 45385 45385 1 74 9 0.122 GCATCCGCATA

MT946551.1 141729 141729 1 98 12 0.122 ATGGACGTAGA

MT946551.1 157546 157546 1 41 5 0.122 ATTGACGCATT

MT946551.1 35294 35294 1 66 8 0.121 AAAAGCGGTAG

MT946551.1 6647 6647 1 50 6 0.12 ATAAACGGAGC

MT946551.1 152238 152238 1 50 6 0.12 AATCTCGTTAC

MT946551.1 2480 2480 1 42 5 0.119 TTCCACGATAA

MT946551.1 23860 23860 1 67 8 0.119 TGTTCCGGAGA

MT946551.1 38294 38294 1 59 7 0.119 TTGTCCGGAGA

MT946551.1 64138 64138 1 67 8 0.119 ATCTACGATCC

MT946551.1 66418 66418 1 67 8 0.119 CCCTCCGTTGA

MT946551.1 80485 80485 1 42 5 0.119 AAACACGTTAG

MT946551.1 118661 118661 1 59 7 0.119 TTCAGCGTGAT

MT946551.1 130548 130548 1 67 8 0.119 ATACCCGGCTA

MT946551.1 139960 139960 1 67 8 0.119 CATATCGCCAC

MT946551.1 141611 141611 1 67 8 0.119 AAGAACGGGTC

MT946551.1 149032 149032 1 42 5 0.119 CTCTCCGACAA

MT946551.1 2142 2142 1 34 4 0.118 AAATGCGTCAA

MT946551.1 4501 4501 1 51 6 0.118 CATGTCGTCTA

MT946551.1 10746 10746 1 68 8 0.118 TTATACGCTAC

MT946551.1 44497 44497 1 76 9 0.118 ATTCCCGTTTT

MT946551.1 57661 57661 1 68 8 0.118 GCATCCGTATA

MT946551.1 93875 93875 1 60 7 0.117 AGCAACGTGAT

MT946551.1 6820 6820 1 43 5 0.116 ATTTCCGAAGA

MT946551.1 35625 35625 1 86 10 0.116 TGGATCGTTCT

MT946551.1 83702 83702 1 69 8 0.116 TTAGTCGTGGT

MT946551.1 133761 133761 1 69 8 0.116 ACACACGGTTA

MT946551.1 154478 154478 1 43 5 0.116 CCATCCGGATG

MT946551.1 51370 51370 1 61 7 0.115 TCTAACGGGAT

MT946551.1 76184 76184 1 87 10 0.115 ATTGGCGTGTG

MT946551.1 77293 77293 1 52 6 0.115 GATTCCGAAGT

MT946551.1 97820 97820 1 78 9 0.115 CTTGACGCAAT

MT946551.1 8843 8843 1 44 5 0.114 CATACCGGGTT

MT946551.1 97422 97422 1 70 8 0.114 ATTAGCGAATT

MT946551.1 117354 117354 1 70 8 0.114 ATCAACGGAGC

MT946551.1 149970 149970 1 44 5 0.114 GATGTCGTTAA

MT946551.1 4117 4117 1 53 6 0.113 CTGGACGATCC

MT946551.1 19734 19734 1 53 6 0.113 TTTCACGAATT

MT946551.1 64885 64885 1 80 9 0.113 TTTAACGAATC

MT946551.1 76875 76875 1 71 8 0.113 CTTTACGATAG

MT946551.1 97339 97339 1 71 8 0.113 TCTATCGTATC

MT946551.1 141387 141389 2 142 16 0.113 TAGCACGCGTGTT

MT946551.1 6991 6991 1 54 6 0.111 ATATACGGGGA

MT946551.1 11230 11230 1 45 5 0.111 TTTTCCGGTTG

MT946551.1 20770 20770 1 63 7 0.111 AATGACGAATC

MT946551.1 51173 51173 1 72 8 0.111 ATTCTCGCTGA

MT946551.1 65124 65124 1 63 7 0.111 AGAGACGGAAA

MT946551.1 138540 138540 1 81 9 0.111 AGGACCGTATT

MT946551.1 45366 45366 1 82 9 0.11 ACAAACGGCTA

MT946551.1 87412 87412 1 73 8 0.11 ACTATCGTCAT

MT946551.1 147690 147690 1 73 8 0.11 CAAAACGAGGA

MT946551.1 149719 149719 1 64 7 0.109 TGTATCGACAA

MT946551.1 155964 155964 1 64 7 0.109 GCTATCGTCTC

MT946551.1 24402 24402 1 74 8 0.108 ATGATCGGATC

MT946551.1 26982 26982 1 83 9 0.108 TTCATCGGCAT

MT946551.1 35680 35680 1 83 9 0.108 ACAAACGAGTT

MT946551.1 45784 45784 1 65 7 0.108 TGCCACGGTTC

MT946551.1 97143 97143 1 74 8 0.108 ATTGTCGTACT

MT946551.1 125657 125657 1 83 9 0.108 TATGCCGGAGA

MT946551.1 146547 146547 1 74 8 0.108 AACAACGAGAT

MT946551.1 149198 149198 1 65 7 0.108 GATGACGTCAA

MT946551.1 20706 20706 1 84 9 0.107 CCTTGCGTTTC

MT946551.1 104333 104333 1 75 8 0.107 CTCTTCGTCAA

MT946551.1 132308 132308 1 75 8 0.107 GGAAACGGATG

MT946551.1 138131 138131 1 56 6 0.107 TGGAACGAGAA

MT946551.1 5119 5119 1 66 7 0.106 AGTATCGATAA

MT946551.1 12180 12180 1 47 5 0.106 AACTCCGGCCA

MT946551.1 46477 46477 1 66 7 0.106 TAATCCGGATG

MT946551.1 12855 12862 2 114 12 0.105 CTTTCCGACTGGCGCATT

MT946551.1 22745 22745 1 57 6 0.105 TCAAACGGTAA

MT946551.1 41233 41233 1 76 8 0.105 AATATCGTGAA

MT946551.1 82802 82802 1 57 6 0.105 GATTCCGGACT

MT946551.1 148279 148279 1 57 6 0.105 ATTGGCGATAA

MT946551.1 11395 11395 1 48 5 0.104 TCATCCGGTGT

MT946551.1 68494 68494 1 67 7 0.104 CTTGACGCATC

MT946551.1 94151 94151 1 77 8 0.104 AACTGCGGACT

MT946551.1 9501 9501 1 58 6 0.103 TAATTCGTGAC

MT946551.1 25843 25843 1 58 6 0.103 AAAGACGGAGG

MT946551.1 56565 56565 1 58 6 0.103 TCCTCCGTTGT

MT946551.1 64310 64310 1 68 7 0.103 ATGCCCGTTTC

MT946551.1 116463 116463 1 78 8 0.103 CAATTCGCTAA

MT946551.1 123893 123893 1 87 9 0.103 TTGGACGATGA

MT946551.1 149350 149350 1 68 7 0.103 TATGCCGTGGG

MT946551.1 113273 113273 1 88 9 0.102 GAACACGATGT

MT946551.1 127881 127881 1 88 9 0.102 TACAACGAATA

MT946551.1 128298 128298 1 88 9 0.102 TAGATCGTCAT

MT946551.1 145393 145393 1 88 9 0.102 CAAGACGTGGC

MT946551.1 32141 32141 1 79 8 0.101 GCCATCGGCAA

MT946551.1 40296 40296 1 69 7 0.101 ATTCTCGTTAA

MT946551.1 58283 58283 1 79 8 0.101 TATCTCGTCTC

MT946551.1 71126 71126 1 69 7 0.101 AATCCCGATGA

MT946551.1 118183 118183 1 69 7 0.101 ACCCACGTTAG

MT946551.1 121147 121147 1 79 8 0.101 ATATACGTAAT

MT946551.1 139253 139253 1 69 7 0.101 AATGGCGAACA

MT946551.1 145286 145286 1 69 7 0.101 ATACACGCTAA

MT946551.1 874 874 1 30 3 0.1 CTTACCGTGCA

MT946551.1 3493 3493 1 50 5 0.1 TTGTACGTCAC

MT946551.1 54051 54051 1 80 8 0.1 GTTCTCGTTTC

MT946551.1 67777 67777 1 70 7 0.1 ACAAACGCTGA

MT946551.1 73625 73625 1 70 7 0.1 AAGTACGGTAA

MT946551.1 79744 79744 1 50 5 0.1 TAATCCGTTAT

MT946551.1 105121 105121 1 70 7 0.1 GGAATCGGTAA

MT946551.1 110483 110483 1 90 9 0.1 ATCATCGACAC

MT946551.1 144500 144500 1 80 8 0.1 ACATCCGTAGA

MT946551.1 158814 158814 1 30 3 0.1 TTGCACGGTAA

MT946551.1 21593 21593 1 71 7 0.099 AACAACGAAAT

MT946551.1 101693 101693 1 81 8 0.099 TAGATCGTCAG

MT946551.1 145636 145636 1 81 8 0.099 GTATACGGCTA

MT946551.1 10466 10466 1 61 6 0.098 TAGGCCGAATC

MT946551.1 36076 36076 1 82 8 0.098 CCAGCCGTAGA

MT946551.1 46058 46058 1 82 8 0.098 GGTGACGACTA

MT946551.1 65517 65517 1 61 6 0.098 CAATCCGATAC

MT946551.1 89183 89183 1 92 9 0.098 ATTAACGATAG

MT946551.1 90384 90384 1 61 6 0.098 CTTTACGCTAT

MT946551.1 93559 93559 1 82 8 0.098 CTAGACGTTGC

MT946551.1 155481 155481 1 41 4 0.098 TGCATCGTGAA

MT946551.1 7638 7638 1 62 6 0.097 CATCACGTCTC

MT946551.1 8187 8187 1 72 7 0.097 AAAAACGAGAA

MT946551.1 23427 23427 1 93 9 0.097 AGCCTCGCTTA

MT946551.1 59976 59976 1 72 7 0.097 ATTGACGACTA

MT946551.1 127710 127710 1 62 6 0.097 AAATTCGGATC

MT946551.1 146951 146951 1 62 6 0.097 GCACTCGTTAT

MT946551.1 42223 42223 1 73 7 0.096 TTAGTCGAGAT

MT946551.1 43251 43251 1 52 5 0.096 AATTCCGCCAT

MT946551.1 72520 72520 1 73 7 0.096 CTTCTCGCAAA

MT946551.1 76829 76829 1 52 5 0.096 CTATCCGACTA

MT946551.1 94818 94818 1 52 5 0.096 ACTATCGGATT

MT946551.1 120768 120768 1 73 7 0.096 TCTACCGTTGT

MT946551.1 39891 39891 1 74 7 0.095 AATGACGTTGA

MT946551.1 45882 45882 1 63 6 0.095 AATACCGTTTG

MT946551.1 115476 115476 1 95 9 0.095 AGTAACGTATG

MT946551.1 117934 117934 1 63 6 0.095 TATGTCGTGAT

MT946551.1 54209 54209 1 64 6 0.094 TATTTCGGATT

MT946551.1 65629 65629 1 64 6 0.094 CTATCCGTACT

MT946551.1 76787 76787 1 64 6 0.094 AGGAACGTCAT

MT946551.1 89012 89012 1 85 8 0.094 TTCTACGTCAT

MT946551.1 94307 94307 1 64 6 0.094 ATTAACGATAA

MT946551.1 154757 154757 1 64 6 0.094 CTCACCGATAG

MT946551.1 156720 156720 1 53 5 0.094 TATATCGTCTC

MT946551.1 159115 159115 1 32 3 0.094 ATCATCGGTGG

MT946551.1 14031 14031 1 54 5 0.093 ATGTCCGTTAT

MT946551.1 25072 25072 1 54 5 0.093 ATAGTCGGAAT

MT946551.1 76386 76386 1 86 8 0.093 CTAGTCGTTTA

MT946551.1 105719 105719 1 75 7 0.093 ATCATCGTAAC

MT946551.1 138119 138119 1 86 8 0.093 TTCCTCGTCCT

MT946551.1 28691 28691 1 87 8 0.092 TCCAACGAGTC

MT946551.1 60195 60195 1 65 6 0.092 TTCTCCGATGG

MT946551.1 69521 69521 1 76 7 0.092 ATTAACGATAA

MT946551.1 78610 78610 1 76 7 0.092 AGTAGCGAAAA

MT946551.1 84378 84378 1 65 6 0.092 GTTTTCGTCAA

MT946551.1 92276 92276 1 87 8 0.092 TAGACCGTTTA

MT946551.1 2123 2123 1 44 4 0.091 GATAGCGTATA

MT946551.1 6452 6452 1 44 4 0.091 AAACACGATAG

MT946551.1 39124 39124 1 66 6 0.091 TTCACCGTGAC

MT946551.1 44887 44887 1 88 8 0.091 GCAGTCGGCAT

MT946551.1 54757 54757 1 77 7 0.091 TTTATCGATAG

MT946551.1 58849 58849 1 88 8 0.091 TTATTCGACTG

MT946551.1 94107 94107 1 66 6 0.091 TCAGACGATCC

MT946551.1 104460 104460 1 55 5 0.091 TTCTCCGGCCA

MT946551.1 106561 106561 1 88 8 0.091 CTAGACGAAAA

MT946551.1 109720 109720 1 55 5 0.091 TATTCCGGTAC

MT946551.1 117696 117696 1 88 8 0.091 ATAAACGAGTA

MT946551.1 142140 142140 1 66 6 0.091 AAATTCGGAAC

MT946551.1 157208 157208 1 44 4 0.091 TTTATCGTGGA

MT946551.1 158080 158080 1 22 2 0.091 GGTCACGTTTC

MT946551.1 27782 27782 1 56 5 0.089 AACATCGGAAA

MT946551.1 70019 70019 1 79 7 0.089 TCCATCGTCAA

MT946551.1 104592 104592 1 90 8 0.089 AGTATCGAAAA

MT946551.1 122396 122396 1 56 5 0.089 ACATCCGATTA

MT946551.1 124719 124719 1 79 7 0.089 GCAAACGATTG

MT946551.1 16920 16920 1 68 6 0.088 GGCCACGCTTT

MT946551.1 38371 38371 1 57 5 0.088 ATGTCCGTTAA

MT946551.1 77338 77338 1 57 5 0.088 TGGACCGTATA

MT946551.1 84119 84119 1 57 5 0.088 TTAATCGGATA

MT946551.1 84973 84973 1 68 6 0.088 TCCTACGAACC

MT946551.1 126085 126085 1 57 5 0.088 TTTGTCGGATT

MT946551.1 139350 139350 1 68 6 0.088 CTAGACGTGGA

MT946551.1 135 135 1 23 2 0.087 TAATTCGTGGT

MT946551.1 50615 50615 1 69 6 0.087 TGTCACGAATA

MT946551.1 52430 52430 1 80 7 0.087 TTCATCGATAA

MT946551.1 58947 58947 1 80 7 0.087 ACAATCGTTAA

MT946551.1 63064 63064 1 69 6 0.087 ACCACCGCAAT

MT946551.1 90249 90249 1 69 6 0.087 ATAATCGCTAA

MT946551.1 103100 103100 1 69 6 0.087 CATACCGTCCC

MT946551.1 108006 108006 1 69 6 0.087 TTTCCCGCAGT

MT946551.1 113732 113732 1 103 9 0.087 CTTATCGTTTA

MT946551.1 130753 130753 1 92 8 0.087 TTTTGCGTATA

MT946551.1 139654 139654 1 80 7 0.087 TTTGTCGATAA

MT946551.1 48457 48457 1 81 7 0.086 ATTTTCGCTCC

MT946551.1 50344 50344 1 70 6 0.086 TCATACGAAAA

MT946551.1 88649 88649 1 70 6 0.086 TTTGACGCTTT

MT946551.1 92037 92037 1 81 7 0.086 AGATACGTGAT

MT946551.1 97167 97167 1 58 5 0.086 ATTAACGATAT

MT946551.1 115554 115554 1 81 7 0.086 CCTGCCGAATT

MT946551.1 21859 21859 1 82 7 0.085 ATGGTCGTTAG

MT946551.1 76264 76264 1 71 6 0.085 AATACCGCAAT

MT946551.1 78935 78935 1 59 5 0.085 CTAAGCGGTAA

MT946551.1 120562 120562 1 59 5 0.085 TTGTCCGTAAA

MT946551.1 135418 135418 1 71 6 0.085 AGTAACGAGTC

MT946551.1 157256 157256 1 47 4 0.085 ATGGCCGTCTA

MT946551.1 127575 127575 1 95 8 0.084 TAAAACGATAA

MT946551.1 3217 3217 1 60 5 0.083 ATATTCGACTT

MT946551.1 15460 15460 1 61 5 0.082 AGACTCGGCAT

MT946551.1 18454 18454 1 85 7 0.082 GATTTCGTCTA

MT946551.1 62689 62689 1 73 6 0.082 TTTTGCGATCA

MT946551.1 79324 79324 1 97 8 0.082 TATATCGATAT

MT946551.1 87300 87300 1 85 7 0.082 ACAGACGAGAT

MT946551.1 105487 105487 1 85 7 0.082 TTCTACGTCTA

MT946551.1 118507 118507 1 61 5 0.082 AATTCCGTTCC

MT946551.1 128660 128660 1 97 8 0.082 GTTATCGTAAT

MT946551.1 150372 150372 1 61 5 0.082 AGTATCGTTGA

MT946551.1 155054 155054 1 49 4 0.082 CTTTCCGAATA

MT946551.1 12231 12231 1 62 5 0.081 ATTGACGGCTA

MT946551.1 48552 48552 1 62 5 0.081 ACCATCGGAGA

MT946551.1 77698 77698 1 86 7 0.081 AGCAACGATAA

MT946551.1 91806 91806 1 74 6 0.081 TGAATCGGATA

MT946551.1 137433 137433 1 86 7 0.081 TGAATCGACTA

MT946551.1 157779 157779 1 37 3 0.081 AGATACGCAAA

MT946551.1 4595 4595 1 50 4 0.08 AATATCGGATT

MT946551.1 34104 34104 1 88 7 0.08 GTGAACGATAT

MT946551.1 43600 43600 1 87 7 0.08 ATACACGTTCA

MT946551.1 53497 53497 1 75 6 0.08 ACATACGAATA

MT946551.1 67319 67319 1 75 6 0.08 TTCAGCGGTAA

MT946551.1 68294 68294 1 75 6 0.08 GGATTCGTTTA

MT946551.1 150721 150721 1 50 4 0.08 TCCCACGATAC

MT946551.1 153627 153627 1 50 4 0.08 GATGACGATGA

MT946551.1 51897 51897 1 76 6 0.079 AAAAACGAATC

MT946551.1 73679 73679 1 63 5 0.079 AGTTCCGCATT

MT946551.1 110631 110631 1 89 7 0.079 TCTAGCGCTGA

MT946551.1 146210 146210 1 63 5 0.079 TATCCCGAAAG

MT946551.1 146643 146643 1 63 5 0.079 AATAACGTCTT

MT946551.1 147014 147014 1 63 5 0.079 AGTCACGATGT

MT946551.1 6528 6528 1 51 4 0.078 TGCATCGTGCT

MT946551.1 42013 42013 1 64 5 0.078 ATTTTCGGGTT

MT946551.1 56339 56339 1 64 5 0.078 TATTCCGTATG

MT946551.1 63788 63788 1 64 5 0.078 TGTGTCGTTAG

MT946551.1 78544 78544 1 77 6 0.078 ATGGACGGAGA

MT946551.1 98354 98354 1 77 6 0.078 AATAACGTATT

MT946551.1 15486 15486 1 65 5 0.077 ATCCCCGTGGA

MT946551.1 19467 19467 1 78 6 0.077 TAGTTCGTAAA

MT946551.1 36588 36588 1 65 5 0.077 TCATCCGTCCT

MT946551.1 52944 52944 1 65 5 0.077 AACCACGAAAC

MT946551.1 55339 55339 1 91 7 0.077 ACAATCGATAA

MT946551.1 74889 74889 1 78 6 0.077 GTCACCGAGGG

MT946551.1 78179 78179 1 91 7 0.077 ACAAACGAAAG

MT946551.1 90717 90717 1 78 6 0.077 TTGATCGTAAG

MT946551.1 104854 104854 1 65 5 0.077 TTGAACGGGTC

MT946551.1 142834 142834 1 78 6 0.077 GATATCGTCAG

MT946551.1 12031 12031 1 66 5 0.076 AAGATCGCAAT

MT946551.1 32161 32161 1 66 5 0.076 GAATCCGAGTA

MT946551.1 83518 83518 1 79 6 0.076 TTTATCGTTGA

MT946551.1 97155 97155 1 79 6 0.076 TATATCGTGTT

MT946551.1 109592 109592 1 79 6 0.076 ACAAACGATTA

MT946551.1 114230 114230 1 105 8 0.076 ATCATCGACAA

MT946551.1 132556 132556 1 79 6 0.076 GTTGCCGTTCT

MT946551.1 143174 143174 1 79 6 0.076 AGTTACGTTTA

MT946551.1 155360 155360 1 66 5 0.076 AAAACCGATAA

MT946551.1 2968 2968 1 40 3 0.075 TGAGACGATAT

MT946551.1 21167 21167 1 80 6 0.075 ATGTACGATCC

MT946551.1 28676 28676 1 80 6 0.075 TTAAACGCAGA

MT946551.1 33255 33255 1 67 5 0.075 GAAACCGTGCA

MT946551.1 42758 42758 1 80 6 0.075 GGATTCGTCCT

MT946551.1 46458 46458 1 80 6 0.075 TCTGGCGGCTA

MT946551.1 52265 52265 1 67 5 0.075 GCTATCGTCAT

MT946551.1 58356 58356 1 67 5 0.075 AATGACGATAA

MT946551.1 75770 75770 1 80 6 0.075 GGCAACGACTA

MT946551.1 81949 81949 1 53 4 0.075 CTATCCGGATA

MT946551.1 113177 113177 1 93 7 0.075 CAAAACGAGTA

MT946551.1 118065 118065 1 93 7 0.075 TGATACGATCA

MT946551.1 121734 121734 1 80 6 0.075 CTAAACGGCAT

MT946551.1 139154 139154 1 53 4 0.075 TCTCACGGAGA

MT946551.1 149986 149986 1 53 4 0.075 ACAATCGTGTC

MT946551.1 156064 156064 1 53 4 0.075 ATATCCGCACT

MT946551.1 24958 24958 1 94 7 0.074 TGTATCGATTA

MT946551.1 34333 34333 1 81 6 0.074 TGGCTCGTCTA

MT946551.1 46768 46768 1 94 7 0.074 ATTATCGTTCC

MT946551.1 48956 48956 1 68 5 0.074 CTTTACGTGAT

MT946551.1 52891 52891 1 94 7 0.074 GAAATCGTATA

MT946551.1 54588 54588 1 68 5 0.074 ACTGACGATAT

MT946551.1 58095 58095 1 68 5 0.074 AGAACCGTCAT

MT946551.1 80238 80244 2 108 8 0.074 AGATCCGTATCCGAAAG

MT946551.1 88085 88085 1 68 5 0.074 CCAACCGGACT

MT946551.1 88916 88916 1 94 7 0.074 TTCAGCGAGTT

MT946551.1 98934 98934 1 68 5 0.074 ATATACGGACT

MT946551.1 127100 127100 1 95 7 0.074 TACCACGTTTG

MT946551.1 137821 137821 1 94 7 0.074 GTAGGCGGTAG

MT946551.1 31871 31871 1 82 6 0.073 AATGACGATAG

MT946551.1 54880 54880 1 55 4 0.073 GTTACCGGATT

MT946551.1 72173 72173 1 96 7 0.073 CTAATCGAGAT

MT946551.1 86863 86863 1 96 7 0.073 ATGAACGTATA

MT946551.1 108428 108428 1 82 6 0.073 ATAATCGTTTT

MT946551.1 10520 10520 1 69 5 0.072 ATCTACGAAAC

MT946551.1 16778 16778 1 69 5 0.072 GATACCGCATC

MT946551.1 31351 31351 1 83 6 0.072 TATACCGAATA

MT946551.1 43918 43918 1 97 7 0.072 GAATGCGTTCC

MT946551.1 59689 59689 1 69 5 0.072 TCAGCCGCTAC

MT946551.1 69968 69968 1 83 6 0.072 ACCAACGGTAT

MT946551.1 79918 79918 1 69 5 0.072 TCTAACGAGAA

MT946551.1 81236 81236 1 69 5 0.072 ACTGCCGTATA

MT946551.1 109767 109767 1 83 6 0.072 TTGCTCGGCAG

MT946551.1 138154 138154 1 69 5 0.072 CATTACGTTAA

MT946551.1 139136 139136 1 83 6 0.072 ATGCACGAGCA

MT946551.1 145014 145014 1 83 6 0.072 ATGAGCGACAA

MT946551.1 1234 1234 1 28 2 0.071 GAAGTCGCTAC

MT946551.1 14148 14148 1 42 3 0.071 ATTGTCGCCAA

MT946551.1 18036 18036 1 85 6 0.071 ATATACGATTA

MT946551.1 21524 21524 1 70 5 0.071 TACACCGGATC

MT946551.1 23967 23967 1 98 7 0.071 TTTTGCGCTAT

MT946551.1 38424 38424 1 84 6 0.071 CTATTCGTAAC

MT946551.1 52066 52066 1 70 5 0.071 TTAGACGCTTC

MT946551.1 59362 59362 1 56 4 0.071 GTTCCCGTCAA

MT946551.1 60372 60372 1 70 5 0.071 TACTTCGACCA

MT946551.1 67667 67667 1 56 4 0.071 AGAGACGGGAA

MT946551.1 81020 81022 2 84 6 0.071 AGATTCGCGTTTA

MT946551.1 82118 82118 1 56 4 0.071 ATTAACGATAT

MT946551.1 90063 90063 1 84 6 0.071 AATGGCGCTAT

MT946551.1 105224 105224 1 84 6 0.071 ACAATCGTCTG

MT946551.1 116640 116640 1 85 6 0.071 GCAACCGTTAG

MT946551.1 125529 125529 1 85 6 0.071 GTATCCGGCCA

MT946551.1 143693 143693 1 56 4 0.071 ACTAACGGGAT

MT946551.1 158454 158454 1 28 2 0.071 GGTAGCGACTT

MT946551.1 17176 17176 1 71 5 0.07 AAATCCGTAGA

MT946551.1 31858 31858 1 57 4 0.07 GTGTCCGATGA

MT946551.1 37849 37849 1 57 4 0.07 ATCAACGTCAT

MT946551.1 44237 44237 1 57 4 0.07 AGAATCGCTAG

MT946551.1 57878 57878 1 86 6 0.07 AAAATCGTGTT

MT946551.1 61539 61539 1 86 6 0.07 ATTATCGTTTG

MT946551.1 64093 64093 1 86 6 0.07 GATTGCGTGAT

MT946551.1 72373 72373 1 86 6 0.07 TACATCGTAAT

MT946551.1 75525 75525 1 71 5 0.07 AGTCCCGAAGA

MT946551.1 83569 83569 1 43 3 0.07 ATGACCGTGAT

MT946551.1 95498 95498 1 71 5 0.07 CCCTGCGGATA

MT946551.1 100358 100358 1 57 4 0.07 TATATCGTGGG

MT946551.1 132150 132150 1 86 6 0.07 TTAGCCGAACA

MT946551.1 139048 139048 1 71 5 0.07 AGATGCGGTGA

MT946551.1 152042 152042 1 71 5 0.07 ACAAACGATGT

MT946551.1 156195 156195 1 57 4 0.07 TGTGACGTACA

MT946551.1 6862 6862 1 58 4 0.069 AATTGCGGACA

MT946551.1 7919 7921 2 58 4 0.069 CTAACCGCGTCTA

MT946551.1 13627 13627 1 87 6 0.069 ATTGTCGAAGC

MT946551.1 34506 34506 1 87 6 0.069 ATCATCGTCTT

MT946551.1 44135 44135 1 87 6 0.069 ATTAGCGTATT

MT946551.1 60341 60341 1 87 6 0.069 ATACACGGTCT

MT946551.1 73126 73126 1 58 4 0.069 GTTAGCGTCAA

MT946551.1 96409 96409 1 72 5 0.069 TAATACGACTC

MT946551.1 137077 137077 1 72 5 0.069 GTATACGCTAC

MT946551.1 140061 140061 1 72 5 0.069 GAAAACGCTGT

MT946551.1 143004 143004 1 87 6 0.069 GTGCTCGACAG

MT946551.1 149904 149904 1 58 4 0.069 GAACCCGACAC

MT946551.1 151054 151054 1 72 5 0.069 TCCATCGATAC

MT946551.1 159571 159571 1 29 2 0.069 TGTACCGGACA

MT946551.1 8900 8900 1 59 4 0.068 TTTTACGAATT

MT946551.1 22922 22922 1 73 5 0.068 TTTGACGACAA

MT946551.1 60213 60213 1 59 4 0.068 TACCACGGATA

MT946551.1 66299 66299 1 74 5 0.068 ATTAACGTTCC

MT946551.1 72509 72509 1 88 6 0.068 TCCAACGATCC

MT946551.1 84957 84957 1 73 5 0.068 AGTACCGTTTA

MT946551.1 96389 96389 1 73 5 0.068 GTTCTCGCAAC

MT946551.1 111447 111447 1 103 7 0.068 TTATACGACTA

MT946551.1 126103 126103 1 74 5 0.068 GTACACGGAAT

MT946551.1 130612 130612 1 88 6 0.068 TTGCTCGTAAA

MT946551.1 136218 136225 2 146 10 0.068 TAATGCGGTATCCGATGT

MT946551.1 141984 141984 1 88 6 0.068 AACAACGTGGA

MT946551.1 153050 153050 1 73 5 0.068 ATCTTCGATAT

MT946551.1 153995 153995 1 59 4 0.068 GTAACCGTAGG

MT946551.1 154713 154713 1 74 5 0.068 TATATCGTATT

MT946551.1 157463 157463 1 44 3 0.068 CAATACGGCCA

MT946551.1 18868 18868 1 75 5 0.067 TAAGTCGAGAA

MT946551.1 25456 25456 1 60 4 0.067 AGCCCCGCAAT

MT946551.1 39834 39834 1 60 4 0.067 GCATCCGTTTC

MT946551.1 60151 60151 1 89 6 0.067 GAAAACGTCCA

MT946551.1 61617 61617 1 89 6 0.067 CTCATCGAAGA

MT946551.1 64801 64801 1 90 6 0.067 ACTACCGATAT

MT946551.1 65792 65792 1 75 5 0.067 ACTTTCGGTTC

MT946551.1 77191 77191 1 60 4 0.067 ACTGACGGAAT

MT946551.1 78096 78096 1 60 4 0.067 AGGACCGTTGG

MT946551.1 82261 82261 1 75 5 0.067 CCAAACGTTTG

MT946551.1 84666 84666 1 75 5 0.067 AGAAACGAATA

MT946551.1 95464 95464 1 90 6 0.067 CTGCACGTTTA

MT946551.1 105049 105049 1 75 5 0.067 AGTACCGATAT

MT946551.1 120137 120137 1 90 6 0.067 GACAGCGGTAG

MT946551.1 120509 120509 1 89 6 0.067 TTATTCGTCTT

MT946551.1 130028 130028 1 75 5 0.067 GATGACGATGA

MT946551.1 135970 135970 1 89 6 0.067 CTAAACGATTT

MT946551.1 146499 146499 1 60 4 0.067 TTTGCCGGAGG

MT946551.1 148747 148747 1 75 5 0.067 AGGCTCGTATA

MT946551.1 149796 149806 2 120 8 0.067 AAATACGCTATCATCCGGATA

MT946551.1 151766 151766 1 60 4 0.067 GGAATCGTCTA

MT946551.1 19174 19174 1 91 6 0.066 AATGCCGTACT

MT946551.1 36526 36531 2 152 10 0.066 AAATTCGGATCGTAAA

MT946551.1 38953 38953 1 61 4 0.066 TGTCTCGTCAA

MT946551.1 53034 53034 1 61 4 0.066 GCATTCGCTAA

MT946551.1 61405 61405 1 76 5 0.066 TGTTTCGGTGG

MT946551.1 65150 65150 1 76 5 0.066 TCTACCGTCCT

MT946551.1 68966 68966 1 61 4 0.066 TTTAGCGTCAT

MT946551.1 75196 75196 1 91 6 0.066 GAGATCGTATT

MT946551.1 75937 75937 1 91 6 0.066 ATAAGCGTATG

MT946551.1 93767 93767 1 91 6 0.066 TCTAGCGCTAA

MT946551.1 97542 97542 1 76 5 0.066 ACTAACGCATC

MT946551.1 114617 114617 1 91 6 0.066 AGATTCGATGG

MT946551.1 135070 135070 1 91 6 0.066 AATAACGAGTT

MT946551.1 135536 135536 1 91 6 0.066 TAAAGCGAGAC

MT946551.1 142072 142072 1 91 6 0.066 CATGTCGTAAT

MT946551.1 144670 144670 1 76 5 0.066 AATAACGTTTT

MT946551.1 148246 148246 1 61 4 0.066 TCTGCCGTGTT

MT946551.1 34974 34974 1 93 6 0.065 ATATACGAACA

MT946551.1 53356 53356 1 62 4 0.065 TGCATCGGTAA

MT946551.1 72952 72952 1 107 7 0.065 TGGTTCGCTCA

MT946551.1 75009 75009 1 93 6 0.065 TCAGACGATAA

MT946551.1 78971 78971 1 62 4 0.065 ATCCCCGTTCT

MT946551.1 83401 83401 1 62 4 0.065 AATGACGAATT

MT946551.1 85885 85885 1 77 5 0.065 TACAGCGTCTG

MT946551.1 92966 92966 1 93 6 0.065 TTTTTCGTATA

MT946551.1 101480 101480 1 77 5 0.065 TATGGCGCCAT

MT946551.1 132819 132819 1 92 6 0.065 ATCATCGTTTT

MT946551.1 146429 146429 1 93 6 0.065 ATGGTCGATTA

MT946551.1 150158 150158 1 62 4 0.065 AACAACGGACA

MT946551.1 156957 156957 1 46 3 0.065 AGAGACGTCAT

MT946551.1 23797 23797 1 78 5 0.064 TTAGCCGACTT

MT946551.1 29137 29137 1 47 3 0.064 TGTAACGGGAA

MT946551.1 79901 79901 1 78 5 0.064 GATACCGAAGA

MT946551.1 80332 80332 1 47 3 0.064 TTAACCGGAGT

MT946551.1 87658 87658 1 94 6 0.064 TTTAACGAGTG

MT946551.1 98976 98976 1 78 5 0.064 ACACACGATGA

MT946551.1 107954 107954 1 78 5 0.064 ATAATCGTCAG

MT946551.1 57306 57306 1 79 5 0.063 GAATGCGGTAG

MT946551.1 61922 61922 1 63 4 0.063 TAATACGGATA

MT946551.1 63313 63318 2 126 8 0.063 AGATACGGAACGGGAC

MT946551.1 74979 74979 1 79 5 0.063 TTTACCGATAA

MT946551.1 77435 77435 1 63 4 0.063 GTGATCGGATA

MT946551.1 97944 97944 1 79 5 0.063 CACAACGGCAC

MT946551.1 106253 106253 1 95 6 0.063 ATGTACGAGTG

MT946551.1 107340 107340 1 63 4 0.063 AATATCGGAAT

MT946551.1 119199 119199 1 95 6 0.063 AATGCCGGTGT

MT946551.1 119561 119561 1 63 4 0.063 TGTTTCGTCAC

MT946551.1 142173 142181 2 158 10 0.063 ATACACGGTATCTCGCTTA

MT946551.1 153611 153611 1 63 4 0.063 AGTGCCGTATA

MT946551.1 7186 7186 1 64 4 0.062 TTCACCGCATA

MT946551.1 26107 26107 1 64 4 0.062 CATGCCGTTTT

MT946551.1 31823 31823 1 80 5 0.062 TTATACGGCTA

MT946551.1 36256 36256 1 96 6 0.062 AACTTCGTAAA

MT946551.1 36968 36968 1 64 4 0.062 ACAATCGGATT

MT946551.1 37061 37061 1 80 5 0.062 AGTACCGATTC

MT946551.1 41071 41071 1 97 6 0.062 TCCATCGTACA

MT946551.1 41920 41920 1 65 4 0.062 CAGGTCGCTAT

MT946551.1 42887 42887 1 96 6 0.062 AATATCGATAT

MT946551.1 43946 43946 1 81 5 0.062 ATAGACGGCTT

MT946551.1 44629 44629 1 112 7 0.062 ATGATCGTCTT

MT946551.1 54862 54862 1 65 4 0.062 ACATCCGAAAT

MT946551.1 55107 55107 1 64 4 0.062 ATGGCCGTTGA

MT946551.1 57268 57268 1 65 4 0.062 TTTAGCGTGGC

MT946551.1 59961 59961 1 80 5 0.062 TAAAGCGTTGA

MT946551.1 63623 63623 1 96 6 0.062 CTAAACGATTG

MT946551.1 66002 66002 1 65 4 0.062 TGTTTCGGTCA

MT946551.1 72713 72713 1 81 5 0.062 GTAGACGATAC

MT946551.1 73396 73396 1 80 5 0.062 GTCTTCGGAAA

MT946551.1 99763 99763 1 80 5 0.062 GAACTCGCTGA

MT946551.1 99915 99915 1 65 4 0.062 TCATACGATCC

MT946551.1 124135 124135 1 64 4 0.062 AAATCCGTTCA

MT946551.1 124478 124478 1 97 6 0.062 TTAAGCGTAGA

MT946551.1 155849 155849 1 48 3 0.062 TGCAACGGAGC

MT946551.1 156897 156897 1 64 4 0.062 ACAAGCGATTT

MT946551.1 157894 157894 1 48 3 0.062 TTTACCGGCAT

MT946551.1 159585 159585 1 16 1 0.062 TATTCCGGTTT

MT946551.1 11085 11085 1 82 5 0.061 ATTCTCGTATA

MT946551.1 18231 18231 1 66 4 0.061 TTTCACGTTAA

MT946551.1 41212 41212 1 66 4 0.061 TTCATCGGTAA

MT946551.1 52611 52611 1 82 5 0.061 ATAGACGAAGG

MT946551.1 59606 59606 1 66 4 0.061 ATATCCGACAA

MT946551.1 63472 63472 1 99 6 0.061 GCAAACGAAGG

MT946551.1 109975 109975 1 66 4 0.061 GAATCCGATCC

MT946551.1 110509 110509 1 99 6 0.061 TTAGACGTTTG

MT946551.1 119102 119102 1 82 5 0.061 GGAAGCGGATA

MT946551.1 133201 133201 1 98 6 0.061 TTAATCGTTTT

MT946551.1 136765 136765 1 82 5 0.061 ATTAACGATGA

MT946551.1 140394 140394 1 82 5 0.061 CTATACGCCAT

MT946551.1 147419 147419 1 66 4 0.061 GTATTCGCCCC

MT946551.1 147617 147617 1 66 4 0.061 TACAACGAGGA

MT946551.1 5649 5649 1 50 3 0.06 GTTCACGATAA

MT946551.1 19576 19576 1 84 5 0.06 TCTGCCGAATA

MT946551.1 29300 29300 1 100 6 0.06 TATTTCGATAT

MT946551.1 30693 30693 1 84 5 0.06 ACCAACGAGGA

MT946551.1 40885 40885 1 67 4 0.06 TTCATCGGCAA

MT946551.1 59094 59094 1 84 5 0.06 GTCTGCGGACT

MT946551.1 70357 70357 1 83 5 0.06 TCAGACGTCTG

MT946551.1 82719 82719 1 67 4 0.06 AGATTCGCATT

MT946551.1 83075 83075 1 67 4 0.06 ATCTCCGTAAA

MT946551.1 99467 99467 1 83 5 0.06 AGTCTCGATAT

MT946551.1 103634 103634 1 83 5 0.06 TACACCGTTAA

MT946551.1 116789 116789 1 50 3 0.06 AGATTCGGAAG

MT946551.1 127839 127839 1 84 5 0.06 TGCCACGAAGA

MT946551.1 139295 139295 1 67 4 0.06 GATAACGGTAC

MT946551.1 148538 148538 1 67 4 0.06 AATCACGCATC

MT946551.1 149868 149868 1 67 4 0.06 TATACCGATAG

MT946551.1 729 729 1 34 2 0.059 AACATCGATTC

MT946551.1 4796 4796 1 51 3 0.059 AGTAACGATTT

MT946551.1 10707 10707 1 68 4 0.059 CCAAACGGTGA

MT946551.1 14198 14198 1 68 4 0.059 AACTTCGTTAT

MT946551.1 15589 15589 1 51 3 0.059 TTCATCGGAGG

MT946551.1 26267 26267 1 68 4 0.059 TACTCCGTTTT

MT946551.1 33864 33867 2 102 6 0.059 AAAAACGACGATTG

MT946551.1 60873 60873 1 68 4 0.059 ATGGCCGTGTA

MT946551.1 113648 113648 1 68 4 0.059 TAGACCGTTAC

MT946551.1 121195 121195 1 102 6 0.059 CCAATCGATTA

MT946551.1 128460 128460 1 85 5 0.059 AGATGCGCCAT

MT946551.1 143925 143925 1 68 4 0.059 ATCTCCGTTAT

MT946551.1 151574 151574 1 51 3 0.059 TTTTCCGCACT

MT946551.1 159492 159492 1 34 2 0.059 TGAATCGATAA

MT946551.1 4658 4658 1 52 3 0.058 ATGTCCGTACC

MT946551.1 6889 6889 1 52 3 0.058 AAATACGAATA

MT946551.1 8302 8302 1 86 5 0.058 CTGAACGTTCA

MT946551.1 22042 22042 1 86 5 0.058 TGATTCGATAA

MT946551.1 22882 22882 1 86 5 0.058 ATAATCGTTAA

MT946551.1 23463 23469 2 104 6 0.058 ACTGACGAATCCGTGAT

MT946551.1 36298 36298 1 104 6 0.058 TCTATCGATAA

MT946551.1 45070 45070 1 104 6 0.058 CAACTCGCCTC

MT946551.1 50465 50465 1 69 4 0.058 ATTAGCGAGGT

MT946551.1 66503 66503 1 69 4 0.058 ATGATCGTTAA

MT946551.1 75916 75916 1 69 4 0.058 AAGACCGATAA

MT946551.1 78465 78465 1 52 3 0.058 TCATCCGAGAC

MT946551.1 84175 84175 1 52 3 0.058 TATGGCGGATT

MT946551.1 84283 84283 1 69 4 0.058 GCTATCGTTAC

MT946551.1 91992 91992 1 86 5 0.058 TTCTTCGCCCT

MT946551.1 98319 98319 1 69 4 0.058 ATCCACGTTTT

MT946551.1 118244 118244 1 86 5 0.058 TCATACGCTAG

MT946551.1 138250 138250 1 69 4 0.058 TACAGCGGCTA

MT946551.1 138770 138770 1 86 5 0.058 TGGGTCGTTGG

MT946551.1 143891 143891 1 86 5 0.058 AGTGGCGTTAA

MT946551.1 146136 146136 1 86 5 0.058 TTAGACGATGT

MT946551.1 157929 157929 1 52 3 0.058 TGTCTCGCATA

MT946551.1 5005 5005 1 53 3 0.057 GTCTGCGCCAT

MT946551.1 11676 11676 1 53 3 0.057 TATAGCGGATC

MT946551.1 17194 17194 1 70 4 0.057 AGCCTCGGTAA

MT946551.1 26995 26995 1 88 5 0.057 TATTACGCTTA

MT946551.1 29074 29074 1 87 5 0.057 TATATCGACAT

MT946551.1 31647 31647 1 70 4 0.057 ATTCTCGGATA

MT946551.1 35956 35956 1 53 3 0.057 CCATCCGATTA

MT946551.1 38509 38512 2 174 10 0.057 TCTATCGACGGCTT

MT946551.1 43437 43437 1 53 3 0.057 TCTTCCGGAGA

MT946551.1 48784 48784 1 53 3 0.057 TGAATCGCTAA

MT946551.1 81598 81598 1 53 3 0.057 ACATCCGCATC

MT946551.1 85476 85476 1 70 4 0.057 ACTGCCGTTCT

MT946551.1 104796 104796 1 70 4 0.057 GACTCCGTATA

MT946551.1 105344 105344 1 53 3 0.057 AAGATCGTGAT

MT946551.1 107282 107282 1 88 5 0.057 CAGGACGAATA

MT946551.1 111716 111716 1 70 4 0.057 AGAAGCGGATT

MT946551.1 123860 123860 1 87 5 0.057 AATGACGTAGA

MT946551.1 124886 124886 1 105 6 0.057 ACCATCGACAA

MT946551.1 138334 138334 1 87 5 0.057 TTACTCGGTGT

MT946551.1 147270 147270 1 70 4 0.057 AAATACGATTC

MT946551.1 152799 152799 1 53 3 0.057 ATACCCGCTAT

MT946551.1 612 612 1 36 2 0.056 ATCCTCGACTG

MT946551.1 8793 8793 1 54 3 0.056 AAATCCGATAA

MT946551.1 9523 9528 2 108 6 0.056 TGAAACGAGACGCTAA

MT946551.1 10364 10364 1 71 4 0.056 ATTGTCGAGCA

MT946551.1 17267 17267 1 90 5 0.056 AAAAACGCCAA

MT946551.1 18182 18182 1 72 4 0.056 TAATTCGTGGA

MT946551.1 28460 28460 1 72 4 0.056 GTCCACGATGA

MT946551.1 32941 32941 1 89 5 0.056 TTATTCGTAGA

MT946551.1 33297 33297 1 90 5 0.056 GTTGCCGAATA

MT946551.1 42375 42375 1 89 5 0.056 ACTCTCGATAC

MT946551.1 50282 50282 1 89 5 0.056 ATAGACGAAGT

MT946551.1 50397 50397 1 71 4 0.056 GGGAACGGCTA

MT946551.1 55786 55786 1 72 4 0.056 TTTAACGATAG

MT946551.1 81353 81353 1 71 4 0.056 ACTTACGTGGA

MT946551.1 82514 82514 1 72 4 0.056 CCATGCGACAA

MT946551.1 82677 82677 1 90 5 0.056 TTAGACGAGGG

MT946551.1 83814 83814 1 71 4 0.056 ACCAACGATAA

MT946551.1 84799 84799 1 71 4 0.056 TATAACGATAC

MT946551.1 89196 89196 1 71 4 0.056 GTAGCCGATAA

MT946551.1 98693 98693 1 71 4 0.056 CATGTCGTTAA

MT946551.1 109418 109418 1 89 5 0.056 TAAAACGATTA

MT946551.1 125343 125343 1 89 5 0.056 TTCCACGTCTA

MT946551.1 132946 132946 1 90 5 0.056 GTTCTCGTCTA

MT946551.1 134076 134076 1 89 5 0.056 TTAGACGACAA

MT946551.1 139008 139008 1 89 5 0.056 AATATCGATTC

MT946551.1 140199 140199 1 90 5 0.056 GTGGACGTACA

MT946551.1 150869 150869 1 54 3 0.056 TATACCGTCTC

MT946551.1 152327 152327 1 54 3 0.056 CAAGACGGATA

MT946551.1 155102 155102 1 72 4 0.056 CTATGCGAGTA

MT946551.1 3228 3228 1 55 3 0.055 GAGTTCGTTAG

MT946551.1 20620 20620 1 73 4 0.055 AAACTCGGTTA

MT946551.1 56138 56138 1 91 5 0.055 TCCATCGATAG

MT946551.1 86417 86417 1 91 5 0.055 TGTTGCGGCAT

MT946551.1 109254 109254 1 55 3 0.055 TCCTCCGATGA

MT946551.1 110426 110426 1 73 4 0.055 ATCAACGGCCA

MT946551.1 111382 111382 1 91 5 0.055 TAATACGATCC

MT946551.1 122626 122626 1 91 5 0.055 GCTTGCGCCAA

MT946551.1 125815 125815 1 73 4 0.055 ATGCCCGTAAA

MT946551.1 134506 134506 1 91 5 0.055 ATGAACGATAA

MT946551.1 134633 134639 2 110 6 0.055 AAATCCGTCCACGAGAT

MT946551.1 145082 145082 1 91 5 0.055 ATCAACGATGA

MT946551.1 20753 20753 1 93 5 0.054 TTTGACGATAT

MT946551.1 21403 21403 1 93 5 0.054 ACCAACGATAA

MT946551.1 21967 21967 1 92 5 0.054 GATATCGAGAA

MT946551.1 32125 32125 1 56 3 0.054 ATTCTCGGACA

MT946551.1 36985 36985 1 92 5 0.054 TCTATCGTCCA

MT946551.1 48969 48969 1 93 5 0.054 ATCATCGATTT

MT946551.1 57975 57975 1 92 5 0.054 AGTGTCGATTG

MT946551.1 62541 62541 1 56 3 0.054 ATACCCGATCC

MT946551.1 75266 75266 1 92 5 0.054 GAAATCGTCCA

MT946551.1 89574 89574 1 56 3 0.054 ACACCCGTTTC

MT946551.1 98818 98818 1 74 4 0.054 TTAGTCGTTTA

MT946551.1 107885 107885 1 92 5 0.054 TGTCTCGTATA

MT946551.1 111493 111493 1 92 5 0.054 TAAAACGAATT

MT946551.1 120282 120285 2 148 8 0.054 ATGAACGACGGGAT

MT946551.1 123512 123512 1 74 4 0.054 TCAGACGGCAA

MT946551.1 124559 124559 1 56 3 0.054 TCTGTCGGAGG

MT946551.1 126336 126336 1 74 4 0.054 ATACACGGGTG

MT946551.1 136090 136090 1 74 4 0.054 CATGGCGAGGA

MT946551.1 136888 136888 1 93 5 0.054 GTAGGCGAATT

MT946551.1 151363 151363 1 37 2 0.054 TATAACGGGAT

MT946551.1 152338 152338 1 56 3 0.054 TACAGCGTTAC

MT946551.1 155643 155643 1 37 2 0.054 ATAAACGGCTA

MT946551.1 156460 156460 1 56 3 0.054 TCTAACGAACT

MT946551.1 8066 8066 1 76 4 0.053 TTATACGCATT

MT946551.1 44592 44592 1 95 5 0.053 TTGATCGATGA

MT946551.1 46600 46600 1 76 4 0.053 TGATCCGTGTT

MT946551.1 50647 50647 1 76 4 0.053 ATTACCGTATG

MT946551.1 60796 60796 1 76 4 0.053 AATAGCGATAT

MT946551.1 63640 63640 1 76 4 0.053 GAAACCGAGAT

MT946551.1 71461 71461 1 94 5 0.053 TCTAGCGATAT

MT946551.1 71971 71971 1 75 4 0.053 ATGTTCGTGAT

MT946551.1 72547 72547 1 95 5 0.053 AAAATCGTATA

MT946551.1 80213 80213 1 75 4 0.053 ATAAACGAGTA

MT946551.1 82527 82527 1 76 4 0.053 ATTATCGATAC

MT946551.1 85435 85435 1 95 5 0.053 TTTTTCGAAAT

MT946551.1 107657 107657 1 95 5 0.053 CAGGACGAAAT

MT946551.1 119168 119168 1 76 4 0.053 TGTGTCGTTGA

MT946551.1 124235 124235 1 76 4 0.053 GAACACGATGA

MT946551.1 135700 135700 1 76 4 0.053 GGACACGATGA

MT946551.1 150490 150500 2 114 6 0.053 ATGACCGATAAGAGACGTGTT

MT946551.1 150515 150515 1 38 2 0.053 CATCCCGGTTA

MT946551.1 153016 153016 1 57 3 0.053 AAGATCGTTGG

MT946551.1 154831 154831 1 76 4 0.053 ATGTACGGCTT

MT946551.1 26561 26561 1 77 4 0.052 CTTTTCGATAT

MT946551.1 33309 33309 1 77 4 0.052 ATGGACGAATT

MT946551.1 36868 36868 1 97 5 0.052 CTTATCGACAT

MT946551.1 38571 38571 1 97 5 0.052 ATAATCGTTTA

MT946551.1 50833 50833 1 77 4 0.052 AGGATCGCAAG

MT946551.1 66275 66275 1 77 4 0.052 TGTTTCGTCAA

MT946551.1 69725 69725 1 97 5 0.052 TTAAACGATGG

MT946551.1 84394 84394 1 58 3 0.052 ATGTCCGAATC

MT946551.1 120456 120456 1 77 4 0.052 TGCCTCGTTAA

MT946551.1 128106 128106 1 96 5 0.052 TTTGACGTATG

MT946551.1 132735 132735 1 97 5 0.052 ATCACCGATGA

MT946551.1 153283 153283 1 58 3 0.052 TAATTCGAAAA

MT946551.1 2225 2225 1 39 2 0.051 ATGGCCGTATT

MT946551.1 10161 10168 2 156 8 0.051 TTATACGGAAGGCGTACC

MT946551.1 14779 14779 1 78 4 0.051 ATCTTCGTCTA

MT946551.1 38173 38173 1 99 5 0.051 TAGATCGAAAA

MT946551.1 46020 46020 1 99 5 0.051 ATCATCGATCA

MT946551.1 52415 52415 1 78 4 0.051 GCTCTCGTATT

MT946551.1 70481 70481 1 78 4 0.051 CTAGCCGCTGG

MT946551.1 78499 78499 1 59 3 0.051 TTATCCGAACT

MT946551.1 94043 94043 1 99 5 0.051 AGGATCGTACA

MT946551.1 94527 94527 1 98 5 0.051 GGTGCCGCCTG

MT946551.1 119114 119114 1 78 4 0.051 AAAACCGTTTC

MT946551.1 120357 120357 1 59 3 0.051 TATAGCGGATA

MT946551.1 126187 126187 1 99 5 0.051 ATCAACGATAT

MT946551.1 134581 134581 1 79 4 0.051 GGATACGGATA

MT946551.1 137263 137263 1 78 4 0.051 TCTTACGACTC

MT946551.1 139580 139590 2 118 6 0.051 AATTACGACAAGTTTCGGCAC

MT946551.1 1888 1888 1 40 2 0.05 GGAGTCGATCC

MT946551.1 26346 26346 1 80 4 0.05 GAATGCGAAGG

MT946551.1 30022 30022 1 80 4 0.05 TGGAACGAATT

MT946551.1 67538 67538 1 80 4 0.05 ATTTACGGTTT

MT946551.1 87724 87724 1 60 3 0.05 AGATCCGAATT

MT946551.1 94020 94020 1 40 2 0.05 GCTCCCGGATT

MT946551.1 95869 95869 1 80 4 0.05 ACATTCGACAT

MT946551.1 97291 97291 1 60 3 0.05 ATACCCGTCTG

MT946551.1 100408 100408 1 80 4 0.05 ATGTTCGAACC

MT946551.1 102506 102506 1 80 4 0.05 TGCCTCGTTTT

MT946551.1 102542 102542 1 80 4 0.05 AGTGACGATGT

MT946551.1 103550 103550 1 80 4 0.05 ATAAGCGTTGT

MT946551.1 103862 103862 1 80 4 0.05 AAGATCGTAAA

MT946551.1 109636 109636 1 100 5 0.05 GGAGACGCCCA

MT946551.1 110015 110015 1 80 4 0.05 AAGAACGTGTA

MT946551.1 121574 121574 1 80 4 0.05 ACTAACGAAAT

MT946551.1 122647 122647 1 100 5 0.05 CAATACGATAA

MT946551.1 130013 130013 1 101 5 0.05 ATTATCGATAT

MT946551.1 141562 141562 1 100 5 0.05 TAATACGAATA

MT946551.1 148235 148235 1 80 4 0.05 ATGGGCGATAT

MT946551.1 150987 150987 1 60 3 0.05 GAATGCGATAC

MT946551.1 1451 1461 2 82 4 0.049 TGTTCCGAGACCACACGGAGA

MT946551.1 2028 2035 3 123 6 0.049 TATGACGACCGGCGATAT

MT946551.1 2844 2844 1 41 2 0.049 CTACACGTGTC

MT946551.1 22512 22512 1 102 5 0.049 TAGTTCGTGTA

MT946551.1 40945 40945 1 81 4 0.049 CTGTTCGTCCA

MT946551.1 43514 43514 1 61 3 0.049 TTTATCGCTAT

MT946551.1 50946 50946 1 82 4 0.049 TACAGCGAATA

MT946551.1 54326 54326 1 82 4 0.049 GGAAACGATGT

MT946551.1 56910 56910 1 81 4 0.049 CAAGCCGGCTA

MT946551.1 77995 77995 1 61 3 0.049 TTTAACGAGGA

MT946551.1 78009 78009 1 61 3 0.049 ACTATCGGATG

MT946551.1 88295 88295 1 82 4 0.049 AAGAACGTGAC

MT946551.1 101931 101931 1 82 4 0.049 TTACACGATGA

MT946551.1 105543 105543 1 82 4 0.049 GAAGACGAGGA

MT946551.1 106475 106475 1 82 4 0.049 GTTTTCGAAGA

MT946551.1 108462 108462 1 102 5 0.049 ATAAACGATTG

MT946551.1 114164 114164 1 82 4 0.049 ATTACCGACAA

MT946551.1 115170 115170 1 103 5 0.049 CTAGTCGCATC

MT946551.1 117825 117825 1 103 5 0.049 TAGTGCGAAAT

MT946551.1 121010 121010 1 61 3 0.049 TTGATCGCTAT

MT946551.1 122315 122315 1 61 3 0.049 AAATCCGATGT

MT946551.1 122904 122904 1 82 4 0.049 TGAAGCGTGTT

MT946551.1 127920 127920 1 82 4 0.049 TACTTCGGTTA

MT946551.1 143244 143244 1 103 5 0.049 AAATACGAAAA

MT946551.1 147374 147374 1 81 4 0.049 GAAACCGATGA

MT946551.1 152686 152686 1 61 3 0.049 AATGCCGATAG

MT946551.1 153787 153787 1 41 2 0.049 ATGTTCGGAGG

MT946551.1 157606 157606 1 61 3 0.049 AGATACGCCTT

MT946551.1 158047 158047 1 41 2 0.049 CCTAACGTATT

MT946551.1 21871 21871 1 84 4 0.048 AGGTACGGTAA

MT946551.1 24168 24168 1 83 4 0.048 ATTAACGGTTA

MT946551.1 28126 28126 1 84 4 0.048 TAATTCGATAC

MT946551.1 31445 31445 1 63 3 0.048 ATAGACGGTTT

MT946551.1 33330 33330 1 83 4 0.048 ATAGGCGTAAA

MT946551.1 42202 42202 1 83 4 0.048 TATCTCGACTT

MT946551.1 66843 66843 1 84 4 0.048 ATAAACGATTA

MT946551.1 69401 69401 1 84 4 0.048 AGTTACGAACC

MT946551.1 72484 72484 1 83 4 0.048 TACATCGGTAA

MT946551.1 72843 72843 1 84 4 0.048 TTAGTCGGTTA

MT946551.1 80199 80199 1 63 3 0.048 ACAACCGCTTA

MT946551.1 80580 80580 1 63 3 0.048 TCCAGCGGCTA

MT946551.1 99290 99290 1 63 3 0.048 TTGAACGCTAT

MT946551.1 105082 105082 1 62 3 0.048 GAGATCGGAGA

MT946551.1 110230 110230 1 84 4 0.048 TTTTGCGGCTC

MT946551.1 116516 116516 1 63 3 0.048 ATTATCGGATA

MT946551.1 121323 121323 1 83 4 0.048 AAATGCGTAAT

MT946551.1 121336 121336 1 83 4 0.048 TAGCCCGTCCT

MT946551.1 122739 122739 1 62 3 0.048 TAGACCGGATA

MT946551.1 128508 128508 1 105 5 0.048 GTGTTCGTCTA

MT946551.1 129756 129756 1 63 3 0.048 AATAGCGTCTA

MT946551.1 130798 130798 1 63 3 0.048 ATGGCCGATGA

MT946551.1 135456 135456 1 84 4 0.048 ACTAACGAGAA

MT946551.1 151729 151729 1 42 2 0.048 ATTTACGGGTA

MT946551.1 153485 153485 1 62 3 0.048 ATATACGAAAT

MT946551.1 155985 155985 1 63 3 0.048 TTCAACGATAA

MT946551.1 812 812 1 43 2 0.047 CAGTACGATAT

MT946551.1 1379 1379 1 43 2 0.047 CACATCGATAT

MT946551.1 12316 12316 1 43 2 0.047 TCATCCGATAA

MT946551.1 15124 15131 2 128 6 0.047 ATATACGATTCACGATAA

MT946551.1 21267 21267 1 86 4 0.047 TTAAACGATTT

MT946551.1 27188 27188 1 86 4 0.047 AACTACGTTCA

MT946551.1 35864 35864 1 85 4 0.047 TTAGTCGTCTC

MT946551.1 48739 48739 1 85 4 0.047 ATATACGATAA

MT946551.1 50163 50168 2 128 6 0.047 CTTTACGGTACGGATC

MT946551.1 53190 53190 1 86 4 0.047 TTATTCGGTAA

MT946551.1 54803 54803 1 64 3 0.047 AGAGACGTAAA

MT946551.1 58549 58549 1 86 4 0.047 CATGACGTGAT

MT946551.1 61038 61038 1 86 4 0.047 AAATTCGTTAG

MT946551.1 62636 62636 1 64 3 0.047 ATCTGCGTTAT

MT946551.1 69347 69347 1 85 4 0.047 GGGTTCGATTA

MT946551.1 71837 71837 1 86 4 0.047 TATTTCGACAA

MT946551.1 78166 78166 1 85 4 0.047 TTAGACGATTC

MT946551.1 78808 78808 1 86 4 0.047 AATGGCGCATC

MT946551.1 99560 99560 1 64 3 0.047 TATATCGGACA

MT946551.1 104822 104822 1 86 4 0.047 ATAAGCGTATA

MT946551.1 111655 111655 1 106 5 0.047 TAAAACGAAAA

MT946551.1 112697 112697 1 107 5 0.047 TATTTCGATAT

MT946551.1 113345 113345 1 85 4 0.047 CATACCGTTTT

MT946551.1 114215 114215 1 85 4 0.047 GGAAGCGTTGA

MT946551.1 117038 117038 1 85 4 0.047 CAAACCGTCTT

MT946551.1 119480 119480 1 64 3 0.047 ACATCCGAAAA

MT946551.1 148192 148192 1 86 4 0.047 GTTAGCGCTCA

MT946551.1 154448 154448 1 64 3 0.047 ATTGACGATGT

MT946551.1 25936 25936 1 65 3 0.046 ACACCCGTAGA

MT946551.1 26313 26313 1 65 3 0.046 AAAGCCGTTAA

MT946551.1 50423 50423 1 65 3 0.046 AATCCCGCATT

MT946551.1 54081 54081 1 87 4 0.046 CTAGTCGATCC

MT946551.1 55123 55123 1 87 4 0.046 AGTACCGATCA

MT946551.1 78118 78118 1 87 4 0.046 AACTACGTCAA

MT946551.1 85787 85787 1 87 4 0.046 TAATGCGGTTT

MT946551.1 98288 98288 1 87 4 0.046 ATCTACGTTCA

MT946551.1 100384 100384 1 87 4 0.046 ACTACCGATGA

MT946551.1 110384 110384 1 87 4 0.046 GACCTCGTTTA

MT946551.1 116161 116161 1 87 4 0.046 AGGTACGAGTA

MT946551.1 124855 124855 1 65 3 0.046 ATTTGCGAATA

MT946551.1 125003 125003 1 108 5 0.046 CCATTCGACTT

MT946551.1 143204 143204 1 65 3 0.046 TTCTTCGGATC

MT946551.1 13371 13371 1 44 2 0.045 GGAGACGGCTA

MT946551.1 24912 24912 1 89 4 0.045 TAAACCGATGA

MT946551.1 25740 25740 1 66 3 0.045 CTCACCGATAA

MT946551.1 28265 28265 1 67 3 0.045 AATAACGTGCT

MT946551.1 39657 39657 1 89 4 0.045 ACAATCGAAAT

MT946551.1 42274 42274 1 66 3 0.045 GCAAACGCTAT

MT946551.1 45926 45926 1 88 4 0.045 TGGCACGAATA

MT946551.1 46509 46509 1 89 4 0.045 TATTTCGGCTG

MT946551.1 58900 58900 1 88 4 0.045 TGGATCGGTTC

MT946551.1 66433 66433 1 67 3 0.045 TAGACCGGCTA

MT946551.1 81393 81396 2 134 6 0.045 TACAACGACGATTG

MT946551.1 95741 95741 1 88 4 0.045 GGAATCGTTCA

MT946551.1 98071 98071 1 89 4 0.045 ACCAACGTCAT

MT946551.1 99671 99671 1 89 4 0.045 TTCATCGTTTA

MT946551.1 101665 101665 1 67 3 0.045 TAGGACGGATC

MT946551.1 105641 105641 1 67 3 0.045 TTTGACGGCTG

MT946551.1 105753 105753 1 66 3 0.045 TTATCCGCCAT

MT946551.1 112493 112493 1 89 4 0.045 AGTTGCGATGA

MT946551.1 126169 126169 1 110 5 0.045 ACCAACGTATA

MT946551.1 131927 131927 1 88 4 0.045 TTATTCGGCAT

MT946551.1 145140 145140 1 88 4 0.045 TTGATCGTCAT

MT946551.1 147520 147520 1 88 4 0.045 AGAACCGAGGA

MT946551.1 155997 155997 1 66 3 0.045 TATATCGACAT

MT946551.1 1794 1794 1 45 2 0.044 GATGCCGGTAA

MT946551.1 2269 2269 1 45 2 0.044 ATAACCGCTTT

MT946551.1 8761 8761 1 68 3 0.044 TAAACCGGTTT

MT946551.1 8958 8958 1 90 4 0.044 TTGTTCGTATG

MT946551.1 14129 14129 1 68 3 0.044 CAATTCGGTTA

MT946551.1 29826 29826 1 90 4 0.044 TGAATCGTATA

MT946551.1 46203 46203 1 91 4 0.044 TTCAACGATCT

MT946551.1 60092 60092 1 91 4 0.044 CCAAGCGTATG

MT946551.1 62088 62088 1 68 3 0.044 CCTATCGGAGA

MT946551.1 72461 72461 1 90 4 0.044 TCCTCCGGCAT

MT946551.1 73090 73090 1 91 4 0.044 AAATTCGTTAG

MT946551.1 85413 85413 1 45 2 0.044 TTTAGCGATTC

MT946551.1 88214 88214 1 91 4 0.044 GTCTTCGTCTA

MT946551.1 89402 89402 1 90 4 0.044 ATCATCGTAAT

MT946551.1 94175 94175 1 68 3 0.044 GATACCGTTTA

MT946551.1 104732 104732 1 91 4 0.044 ATCAACGATCT

MT946551.1 106079 106079 1 68 3 0.044 AATGTCGAGAT

MT946551.1 107127 107130 2 136 6 0.044 AGATTCGTCGGTGC

MT946551.1 110360 110360 1 90 4 0.044 TGTATCGACAT

MT946551.1 111238 111238 1 90 4 0.044 TTAGGCGGCAA

MT946551.1 132695 132695 1 90 4 0.044 ATTGTCGCTTA

MT946551.1 138381 138381 1 68 3 0.044 AGCTTCGTTGT

MT946551.1 154204 154207 2 90 4 0.044 AATATCGACGATAT

MT946551.1 4759 4759 1 69 3 0.043 ATAATCGACTA

MT946551.1 10229 10229 1 92 4 0.043 AACTACGTTCA

MT946551.1 12167 12167 1 70 3 0.043 TGTATCGTACT

MT946551.1 24837 24837 1 92 4 0.043 TTCTACGTACA

MT946551.1 25693 25693 1 93 4 0.043 ACTATCGATAG

MT946551.1 34276 34276 1 92 4 0.043 GTAGACGGCTG

MT946551.1 34320 34320 1 69 3 0.043 TTAGACGGACA

MT946551.1 34463 34463 1 70 3 0.043 TCCATCGCTAA

MT946551.1 34806 34806 1 92 4 0.043 AGTGACGAATC

MT946551.1 38794 38794 1 70 3 0.043 ACATCCGTCTG

MT946551.1 42842 42842 1 93 4 0.043 TGTAGCGCTTC

MT946551.1 61935 61935 1 69 3 0.043 ACTGCCGTCAT

MT946551.1 69044 69052 2 138 6 0.043 AATTTCGTTGAACCGGAAG

MT946551.1 70253 70253 1 93 4 0.043 GAATACGTTAG

MT946551.1 70757 70757 1 70 3 0.043 TGTTCCGATTT

MT946551.1 78028 78028 1 93 4 0.043 CAATACGCCAA

MT946551.1 81679 81679 1 46 2 0.043 TGTTCCGTTGG

MT946551.1 82135 82135 1 47 2 0.043 ATTAACGGATG

MT946551.1 82301 82301 1 69 3 0.043 TGAGACGGGTC

MT946551.1 86527 86527 1 93 4 0.043 TTAAACGAATA

MT946551.1 86573 86573 1 94 4 0.043 TGCTGCGTGTA

MT946551.1 93421 93421 1 69 3 0.043 AGACTCGCAAA

MT946551.1 104922 104922 1 92 4 0.043 TAAAACGATTA

MT946551.1 111298 111298 1 69 3 0.043 TAGATCGTATA

MT946551.1 111514 111514 1 92 4 0.043 TAGGACGAGAC

MT946551.1 113332 113332 1 93 4 0.043 TAAGTCGTTGG

MT946551.1 116445 116445 1 94 4 0.043 TCCATCGATAC

MT946551.1 120297 120297 1 94 4 0.043 TTGTTCGATTG

MT946551.1 132331 132331 1 69 3 0.043 TATAGCGTGAA

MT946551.1 136947 136947 1 92 4 0.043 TCTTTCGGTGG

MT946551.1 159181 159181 1 23 1 0.043 GAATCCGCTGA

MT946551.1 18750 18750 1 96 4 0.042 GATACCGCACT

MT946551.1 24639 24639 1 71 3 0.042 ATATCCGTATC

MT946551.1 31460 31460 1 95 4 0.042 CTATACGATAG

MT946551.1 36037 36037 1 96 4 0.042 TTTTACGTACC

MT946551.1 37086 37086 1 95 4 0.042 TTCTACGTCCT

MT946551.1 37667 37667 1 96 4 0.042 GTACTCGCTCT

MT946551.1 38647 38647 1 96 4 0.042 CTTATCGAAGT

MT946551.1 41647 41647 1 72 3 0.042 AATATCGGAAT

MT946551.1 50690 50690 1 96 4 0.042 ATAGACGAAAT

MT946551.1 52750 52750 1 72 3 0.042 TATCACGAACT

MT946551.1 60654 60654 1 95 4 0.042 CACATCGGTGA

MT946551.1 76939 76939 1 72 3 0.042 TTACACGCTAT

MT946551.1 79075 79075 1 71 3 0.042 TTTGTCGGGTA

MT946551.1 80971 80971 1 71 3 0.042 TTTAACGGCTA

MT946551.1 86051 86051 1 71 3 0.042 TGATCCGATAC

MT946551.1 92550 92550 1 72 3 0.042 GCCAACGAATC

MT946551.1 100200 100200 1 72 3 0.042 ATTTCCGGCTG

MT946551.1 110330 110330 1 96 4 0.042 TATATCGTCTC

MT946551.1 113491 113491 1 96 4 0.042 AAGATCGACTA

MT946551.1 128445 128445 1 71 3 0.042 TCCTCCGTTTG

MT946551.1 130645 130645 1 71 3 0.042 TTCTCCGTAAA

MT946551.1 131530 131530 1 72 3 0.042 ATTAGCGTGTG

MT946551.1 138633 138633 1 95 4 0.042 TTCAACGTCTA

MT946551.1 156568 156568 1 48 2 0.042 GGTCTCGGATT

MT946551.1 2753 2753 1 49 2 0.041 ATTAACGATTT

MT946551.1 6244 6244 1 73 3 0.041 GGCATCGATGT

MT946551.1 14549 14549 1 74 3 0.041 ATCTACGATAT

MT946551.1 16483 16483 1 74 3 0.041 AATATCGGCAT

MT946551.1 23144 23144 1 74 3 0.041 TGAGTCGTCAA

MT946551.1 25672 25672 1 74 3 0.041 ATCCACGTAAC

MT946551.1 26001 26001 1 74 3 0.041 TATAACGTCTT

MT946551.1 42236 42236 1 73 3 0.041 ATGGACGCAGA

MT946551.1 44536 44536 1 98 4 0.041 ATACTCGTTAG

MT946551.1 58531 58531 1 73 3 0.041 TTAAACGTGGA

MT946551.1 65102 65102 1 98 4 0.041 CTCTTCGTCTT

MT946551.1 67640 67640 1 74 3 0.041 GCATACGCCAA

MT946551.1 71653 71653 1 98 4 0.041 ATCATCGAACT

MT946551.1 84136 84136 1 74 3 0.041 CTCAACGGAAA

MT946551.1 85558 85558 1 73 3 0.041 AATAGCGGTAA

MT946551.1 93254 93254 1 49 2 0.041 TCATCCGTGGA

MT946551.1 98040 98040 1 73 3 0.041 AATAGCGCATA

MT946551.1 98867 98867 1 73 3 0.041 GAAGGCGTCAA

MT946551.1 116558 116558 1 73 3 0.041 TGAAACGGATA

MT946551.1 136165 136165 1 73 3 0.041 ATTGGCGGACA

MT946551.1 157591 157591 1 49 2 0.041 TTATTCGCCTG

MT946551.1 21933 21933 1 101 4 0.04 TGGGACGTCCT

MT946551.1 21984 21984 1 99 4 0.04 TGATACGATAT

MT946551.1 22253 22253 1 100 4 0.04 ACCAACGATTT

MT946551.1 65239 65239 1 75 3 0.04 TCAGTCGCTGA

MT946551.1 81223 81223 1 99 4 0.04 CAGATCGATAG

MT946551.1 89049 89049 1 99 4 0.04 ATGTACGAACA

MT946551.1 91531 91531 1 75 3 0.04 TTATCCGTATT

MT946551.1 93950 93950 1 100 4 0.04 ACCATCGTCTG

MT946551.1 110696 110696 1 75 3 0.04 TTCTGCGGCTA

MT946551.1 117877 117877 1 100 4 0.04 AAATGCGTATA

MT946551.1 123412 123412 1 101 4 0.04 AGAAGCGTATA

MT946551.1 125355 125355 1 100 4 0.04 TAGCTCGTGGT

MT946551.1 131913 131913 1 75 3 0.04 ATATCCGGCAA

MT946551.1 148844 148847 2 100 4 0.04 AGTGTCGACGCTAT

MT946551.1 156378 156378 1 50 2 0.04 AACTCCGCTAG

MT946551.1 3310 3310 1 51 2 0.039 CCTAGCGGAGT

MT946551.1 3917 3917 1 51 2 0.039 TTCATCGAGAG

MT946551.1 13477 13477 1 76 3 0.039 ATCATCGATCT

MT946551.1 19041 19041 1 77 3 0.039 GTTTCCGCCAT

MT946551.1 23634 23637 2 154 6 0.039 ATATACGTCGTTCT

MT946551.1 25222 25222 1 77 3 0.039 TGAAACGGTGA

MT946551.1 28911 28911 1 77 3 0.039 TTAACCGTTTC

MT946551.1 34586 34586 1 77 3 0.039 CAAGACGGATA

MT946551.1 45192 45192 1 102 4 0.039 AATACCGATAG

MT946551.1 47837 47837 1 77 3 0.039 CCAACCGAATG

MT946551.1 51292 51292 1 103 4 0.039 TGATACGTATA

MT946551.1 52443 52443 1 51 2 0.039 GATACCGTTAA

MT946551.1 58663 58663 1 51 2 0.039 GGACCCGGTGG

MT946551.1 66811 66811 1 77 3 0.039 ATTAGCGTCAA

MT946551.1 98444 98444 1 76 3 0.039 ATCTTCGTTAA

MT946551.1 107064 107064 1 77 3 0.039 CCCAACGTATT

MT946551.1 109817 109817 1 77 3 0.039 TATGTCGAGAA

MT946551.1 113631 113631 1 76 3 0.039 AAAGCCGGAGT

MT946551.1 114662 114662 1 102 4 0.039 TGAGACGTTGG

MT946551.1 118768 118768 1 77 3 0.039 TTAGCCGTTGT

MT946551.1 124841 124841 1 102 4 0.039 ATATGCGATCA

MT946551.1 124958 124958 1 102 4 0.039 AGCATCGAGCA

MT946551.1 133546 133546 1 77 3 0.039 AAACCCGACTT

MT946551.1 135202 135202 1 102 4 0.039 GTAGACGAACA

MT946551.1 143124 143124 1 76 3 0.039 AATAACGCTAA

MT946551.1 155206 155206 1 51 2 0.039 CTGTCCGTCTT

MT946551.1 156270 156270 1 76 3 0.039 CAATGCGCTAC

MT946551.1 5138 5138 1 53 2 0.038 ACTCACGCATA

MT946551.1 7685 7685 1 53 2 0.038 ATATCCGTGTC

MT946551.1 11125 11125 1 78 3 0.038 GGCAACGAATA

MT946551.1 24129 24129 1 78 3 0.038 ACAACCGAGTA

MT946551.1 26643 26643 1 79 3 0.038 ATCATCGAATT

MT946551.1 51494 51494 1 79 3 0.038 AGACACGACTA

MT946551.1 88309 88309 1 105 4 0.038 TGAGACGAATG

MT946551.1 96079 96079 1 78 3 0.038 GGTAACGTCTG

MT946551.1 102750 102750 1 78 3 0.038 ATAGCCGTTTC

MT946551.1 119984 119984 1 105 4 0.038 AAAATCGTATA

MT946551.1 123812 123812 1 79 3 0.038 GATAGCGAATC

MT946551.1 124343 124343 1 79 3 0.038 GTATTCGGCAT

MT946551.1 129955 129955 1 106 4 0.038 ATTAGCGCAAT

MT946551.1 129971 129971 1 104 4 0.038 GCTGTCGAAGA

MT946551.1 131497 131497 1 106 4 0.038 AAAGGCGTTAT

MT946551.1 135405 135405 1 78 3 0.038 GTTGACGAATA

MT946551.1 135889 135889 1 53 2 0.038 CATACCGCAAA

MT946551.1 136723 136723 1 53 2 0.038 ACTTACGCAAA

MT946551.1 137358 137358 1 106 4 0.038 GACATCGCCTA

MT946551.1 145602 145604 2 104 4 0.038 ACAAACGCGGTGA

MT946551.1 153568 153568 1 79 3 0.038 TCATGCGATGT

MT946551.1 5526 5526 1 54 2 0.037 GTTTACGTAAA

MT946551.1 13265 13265 1 54 2 0.037 ATTCACGTTAT

MT946551.1 14070 14070 1 82 3 0.037 GTATGCGATAG

MT946551.1 14116 14116 1 81 3 0.037 TACATCGTCTA

MT946551.1 16748 16748 1 82 3 0.037 AGAGCCGCAGT

MT946551.1 19422 19422 1 82 3 0.037 ATTATCGGGTT

MT946551.1 27690 27690 1 82 3 0.037 ATAATCGTAAA

MT946551.1 45815 45815 1 82 3 0.037 CAGGCCGTTTG

MT946551.1 48538 48538 1 80 3 0.037 TGATTCGTGTT

MT946551.1 56793 56793 1 81 3 0.037 TTTATCGTTGA

MT946551.1 64648 64648 1 81 3 0.037 TATTTCGTTTC

MT946551.1 69844 69844 1 80 3 0.037 AGCCCCGCTTC

MT946551.1 70570 70570 1 107 4 0.037 TCTGCCGCAAG

MT946551.1 73720 73720 1 81 3 0.037 ACTAACGATTA

MT946551.1 80005 80005 1 82 3 0.037 AGATACGTATA

MT946551.1 81139 81139 1 82 3 0.037 AGACACGTATA

MT946551.1 87357 87357 1 81 3 0.037 AGAAACGATTA

MT946551.1 89140 89140 1 81 3 0.037 CTGGTCGTAGT

MT946551.1 97070 97070 1 82 3 0.037 AAAAACGATAT

MT946551.1 98992 98992 1 80 3 0.037 TACTTCGATGA

MT946551.1 101502 101502 1 81 3 0.037 AATAGCGCCAG

MT946551.1 103943 103943 1 81 3 0.037 TCCAGCGTTAA

MT946551.1 107409 107409 1 81 3 0.037 AGGATCGTTTC

MT946551.1 108153 108153 1 81 3 0.037 GAATTCGTAAA

MT946551.1 108807 108807 1 80 3 0.037 AAATGCGCCAA

MT946551.1 109138 109138 1 82 3 0.037 ACAAACGATGC

MT946551.1 122493 122493 1 82 3 0.037 GAAATCGCTTA

MT946551.1 124589 124589 1 82 3 0.037 AATGACGTCAA

MT946551.1 126849 126849 1 108 4 0.037 AAAGTCGAATT

MT946551.1 129158 129158 1 81 3 0.037 TAGAACGTGTA

MT946551.1 134712 134712 1 82 3 0.037 AGAATCGCATC

MT946551.1 137884 137884 1 81 3 0.037 ATTACCGCATT

MT946551.1 138142 138142 1 82 3 0.037 GGTGTCGATTA

MT946551.1 147208 147208 1 82 3 0.037 TAGTTCGTTCT

MT946551.1 147546 147546 1 82 3 0.037 ATAAACGTCTT

MT946551.1 154975 154975 1 80 3 0.037 ACTATCGATGA

MT946551.1 155027 155027 1 81 3 0.037 TCCATCGATGA

MT946551.1 117 117 1 28 1 0.036 GTGTCCGGTAC

MT946551.1 6283 6283 1 55 2 0.036 CTTATCGTTAA

MT946551.1 20382 20382 1 83 3 0.036 ACAAACGAAAG

MT946551.1 26953 26953 1 83 3 0.036 CTATGCGATTA

MT946551.1 32737 32737 1 83 3 0.036 GAATCCGCTAC

MT946551.1 38534 38534 1 55 2 0.036 TGTTCCGTTAA

MT946551.1 46294 46294 1 84 3 0.036 ATAACCGAGTT

MT946551.1 49194 49194 1 83 3 0.036 CATACCGATAG

MT946551.1 53403 53403 1 83 3 0.036 CATTTCGCTGT

MT946551.1 55645 55645 1 84 3 0.036 GATTTCGAAAA

MT946551.1 55920 55920 1 83 3 0.036 TTATACGAAAA

MT946551.1 63330 63330 1 84 3 0.036 ATGGACGCATG

MT946551.1 68949 68949 1 84 3 0.036 TTGAACGATTC

MT946551.1 72271 72271 1 111 4 0.036 TTGTTCGATGT

MT946551.1 74078 74080 2 110 4 0.036 ATATTCGCGTCTA

MT946551.1 76539 76539 1 84 3 0.036 TAAGCCGCCAT

MT946551.1 79538 79538 1 84 3 0.036 CAGCACGGTGG

MT946551.1 80993 80993 1 84 3 0.036 ATTATCGAGGT

MT946551.1 86972 86972 1 83 3 0.036 TCAATCGTTGG

MT946551.1 96334 96334 1 83 3 0.036 TGTAGCGGTTG

MT946551.1 101428 101428 1 83 3 0.036 TTGGTCGTAGC

MT946551.1 105734 105734 1 83 3 0.036 TCTAACGGCTA

MT946551.1 107788 107788 1 55 2 0.036 TCTCCCGGAGA

MT946551.1 121625 121625 1 56 2 0.036 ATAAACGGGTG

MT946551.1 123968 123968 1 84 3 0.036 ATTTACGATAG

MT946551.1 129585 129585 1 83 3 0.036 TGTATCGTGTC

MT946551.1 129781 129781 1 84 3 0.036 ATATACGTGTT

MT946551.1 138950 138950 1 83 3 0.036 AAGCACGTAGG

MT946551.1 139782 139782 1 84 3 0.036 ATCATCGACTG

MT946551.1 145652 145652 1 55 2 0.036 ACATTCGGAGA

MT946551.1 153655 153655 1 84 3 0.036 TATTTCGTATC

MT946551.1 157270 157270 1 55 2 0.036 ATGTACGGTCT

MT946551.1 157419 157419 1 56 2 0.036 CAAAGCGGTTA

MT946551.1 6312 6312 1 57 2 0.035 GCTATCGCATT

MT946551.1 8602 8602 1 57 2 0.035 TACACCGCTAT

MT946551.1 19664 19664 1 86 3 0.035 ACATGCGGTTA

MT946551.1 21922 21922 1 86 3 0.035 TTTAACGTAGT

MT946551.1 28229 28229 1 86 3 0.035 GATATCGATCT

MT946551.1 31285 31285 1 86 3 0.035 ATAATCGTTGT

MT946551.1 40459 40459 1 85 3 0.035 AAGAGCGACAG

MT946551.1 42034 42034 1 86 3 0.035 AATATCGACAA

MT946551.1 45317 45317 1 113 4 0.035 CAAATCGCCCA

MT946551.1 45671 45671 1 115 4 0.035 TCACTCGTTTC

MT946551.1 52302 52302 1 86 3 0.035 TCATGCGATAG

MT946551.1 52790 52790 1 86 3 0.035 CCCACCGTTAT

MT946551.1 54925 54925 1 85 3 0.035 AAAATCGATAA

MT946551.1 63301 63301 1 86 3 0.035 TCTAACGATAA

MT946551.1 66563 66563 1 85 3 0.035 GAATACGATGA

MT946551.1 71422 71422 1 86 3 0.035 TAACTCGGTAA

MT946551.1 82983 82983 1 85 3 0.035 GGACACGATAT

MT946551.1 106692 106692 1 86 3 0.035 TTTATCGATTC

MT946551.1 106722 106722 1 57 2 0.035 TTAAACGTGAA

MT946551.1 119881 119881 1 86 3 0.035 TTAAACGATAT

MT946551.1 129609 129609 1 85 3 0.035 GGCAACGAGTA

MT946551.1 141801 141801 1 85 3 0.035 GATGCCGAAGA

MT946551.1 149519 149519 1 57 2 0.035 AATTACGTGGA

MT946551.1 154432 154432 1 57 2 0.035 GTCAACGTCAT

MT946551.1 157284 157284 1 57 2 0.035 ATTATCGAGAT

MT946551.1 3405 3405 1 59 2 0.034 GGAGACGTAAC

MT946551.1 3431 3431 1 58 2 0.034 TTCCTCGTCTA

MT946551.1 3693 3693 1 58 2 0.034 GCCATCGCTAT

MT946551.1 8919 8919 1 59 2 0.034 AATAGCGTTTA

MT946551.1 14423 14423 1 87 3 0.034 ATAATCGAGTA

MT946551.1 22151 22151 1 89 3 0.034 GAAAACGAAAA

MT946551.1 23241 23241 1 89 3 0.034 CATTTCGTGCC

MT946551.1 23616 23616 1 88 3 0.034 TCTGGCGGTTG

MT946551.1 24569 24569 1 87 3 0.034 AAAGTCGACAT

MT946551.1 25828 25828 1 87 3 0.034 CAAGACGTCAT

MT946551.1 33659 33659 1 58 2 0.034 TGATGCGGATA

MT946551.1 34233 34233 1 58 2 0.034 GAGAACGGAAT

MT946551.1 35488 35488 1 88 3 0.034 AGATACGCAAA

MT946551.1 42422 42422 1 87 3 0.034 ATCTTCGTAAA

MT946551.1 45646 45646 1 87 3 0.034 ATCAACGACCA

MT946551.1 50728 50728 1 89 3 0.034 TGTATCGATAA

MT946551.1 52463 52463 1 87 3 0.034 GTTGCCGATAG

MT946551.1 57360 57360 1 87 3 0.034 TGTGGCGTTAC

MT946551.1 63032 63032 1 89 3 0.034 TGATGCGATTC

MT946551.1 71905 71905 1 88 3 0.034 AAACACGTCAC

MT946551.1 76578 76578 1 88 3 0.034 CTCTACGATTC

MT946551.1 82281 82281 1 88 3 0.034 GCTATCGGTGA

MT946551.1 84303 84303 1 87 3 0.034 GAAATCGTCTT

MT946551.1 85332 85332 1 89 3 0.034 AACATCGAAGA

MT946551.1 96794 96797 2 118 4 0.034 TTAAACGACGCTAT

MT946551.1 97494 97494 1 89 3 0.034 GAGAACGTATT

MT946551.1 101875 101875 1 87 3 0.034 AAATACGTTCT

MT946551.1 105875 105875 1 89 3 0.034 GGAGGCGAATT

MT946551.1 113459 113459 1 59 2 0.034 AAATCCGAGAA

MT946551.1 115487 115487 1 89 3 0.034 TGAATCGGTTC

MT946551.1 115760 115760 1 89 3 0.034 GATTGCGGCTA

MT946551.1 117142 117146 2 174 6 0.034 AGAATCGGACGTCTG

MT946551.1 127170 127178 2 176 6 0.034 ATCTTCGAAGGACCGTCTG

MT946551.1 132713 132713 1 88 3 0.034 GATGTCGAATT

MT946551.1 156257 156257 1 59 2 0.034 ATAGACGAGGA

MT946551.1 2808 2808 1 61 2 0.033 TAATACGCCTC

MT946551.1 5045 5045 1 61 2 0.033 CTATGCGACTT

MT946551.1 11589 11589 1 60 2 0.033 CATAGCGATAG

MT946551.1 15779 15779 1 60 2 0.033 ATTTGCGTGGT

MT946551.1 20956 20956 1 61 2 0.033 GTTGCCGTAAA

MT946551.1 21703 21703 1 92 3 0.033 TAAACCGTTTT

MT946551.1 24227 24227 1 92 3 0.033 TCCCTCGTTCA

MT946551.1 26417 26417 1 91 3 0.033 ACCTTCGTCCC

MT946551.1 26966 26966 1 91 3 0.033 TTCATCGATGA

MT946551.1 29541 29541 1 90 3 0.033 TAGGACGAACT

MT946551.1 35785 35785 1 92 3 0.033 AGATACGTCTA

MT946551.1 38558 38558 1 90 3 0.033 ATGAGCGTAAA

MT946551.1 42166 42166 1 60 2 0.033 TTCCTCGGCCA

MT946551.1 44508 44508 1 90 3 0.033 TAGACCGAGAC

MT946551.1 44922 44922 1 91 3 0.033 AAGACCGTATG

MT946551.1 48834 48834 1 90 3 0.033 CACTTCGGATC

MT946551.1 50868 50868 1 91 3 0.033 ATCAACGAAAA

MT946551.1 60069 60069 1 61 2 0.033 GGCAGCGTTGT

MT946551.1 61341 61341 1 90 3 0.033 CTTTTCGTAGG

MT946551.1 63406 63406 1 61 2 0.033 TTCTCCGTGAT

MT946551.1 63678 63678 1 90 3 0.033 TCAATCGGTGT

MT946551.1 70435 70435 1 60 2 0.033 ATTAACGGATA

MT946551.1 83128 83128 1 61 2 0.033 AATACCGGAAT

MT946551.1 85160 85160 1 92 3 0.033 TAAGACGTATC

MT946551.1 86987 86987 1 90 3 0.033 TTTATCGCATA

MT946551.1 87472 87472 1 61 2 0.033 TTATCCGGGTG

MT946551.1 93478 93478 1 61 2 0.033 ACAACCGCTAC

MT946551.1 96490 96490 1 90 3 0.033 ATTGACGACTT

MT946551.1 96747 96747 1 90 3 0.033 AGATGCGCATT

MT946551.1 100115 100115 1 92 3 0.033 AATATCGTCCT

MT946551.1 104048 104048 1 60 2 0.033 AGTTACGGATC

MT946551.1 106225 106225 1 91 3 0.033 ATAAACGCCAT

MT946551.1 107314 107314 1 91 3 0.033 TATCTCGCATC

MT946551.1 108721 108721 1 92 3 0.033 TTTTACGCCTC

MT946551.1 110778 110783 2 184 6 0.033 AATGTCGAGACGTCAA

MT946551.1 110888 110888 1 90 3 0.033 TTATTCGAAGG

MT946551.1 111745 111745 1 91 3 0.033 ACATGCGGTTC

MT946551.1 112140 112140 1 91 3 0.033 TACATCGGCTG

MT946551.1 115395 115395 1 60 2 0.033 TTAGACGACAT

MT946551.1 118848 118848 1 92 3 0.033 GCAGCCGTTGT

MT946551.1 139337 139337 1 92 3 0.033 GGATACGTTGT

MT946551.1 142480 142480 1 92 3 0.033 CTAAACGACCA

MT946551.1 143858 143858 1 91 3 0.033 AGAATCGTTAG

MT946551.1 145463 145463 1 91 3 0.033 GATTTCGTATC

MT946551.1 156880 156880 1 61 2 0.033 AGAGGCGTATT

MT946551.1 40 40 1 31 1 0.032 ATAATCGTATC

MT946551.1 247 247 1 31 1 0.032 ATCATCGGTTG

MT946551.1 6703 6703 1 63 2 0.032 CTTAACGAAAG

MT946551.1 17122 17122 1 95 3 0.032 AGTATCGATCA

MT946551.1 23209 23209 1 94 3 0.032 ATCATCGTATA

MT946551.1 24623 24623 1 93 3 0.032 ATCTTCGTCTA

MT946551.1 26433 26433 1 62 2 0.032 AGATGCGAGAG

MT946551.1 27205 27205 1 62 2 0.032 CCTGGCGAATA

MT946551.1 30409 30409 1 95 3 0.032 CTTATCGAATA

MT946551.1 34860 34860 1 95 3 0.032 AGATACGAAGA

MT946551.1 40362 40371 2 186 6 0.032 TTTTCCGTGTAAGGCGTATC

MT946551.1 42512 42512 1 94 3 0.032 AAAGGCGCCCA

MT946551.1 46631 46636 2 188 6 0.032 TAATACGAGACGTGAT

MT946551.1 50051 50051 1 62 2 0.032 TTTCACGAAAG

MT946551.1 50579 50579 1 62 2 0.032 TCATCCGGTAT

MT946551.1 64201 64201 1 63 2 0.032 CCAAACGAGGA

MT946551.1 68057 68057 1 94 3 0.032 GATATCGTCTG

MT946551.1 72747 72747 1 95 3 0.032 ACAAACGAAGA

MT946551.1 78246 78246 1 94 3 0.032 GATTGCGTTAT

MT946551.1 84880 84880 1 62 2 0.032 GTAAACGGTCA

MT946551.1 90949 90949 1 95 3 0.032 ATTCTCGAGAT

MT946551.1 99113 99113 1 63 2 0.032 TATGCCGTTAT

MT946551.1 104938 104938 1 63 2 0.032 TATCACGAAAG

MT946551.1 110874 110874 1 95 3 0.032 GGGAGCGTCTT

MT946551.1 111812 111812 1 62 2 0.032 TAACCCGAGTA

MT946551.1 114376 114376 1 94 3 0.032 CTAAACGAATT

MT946551.1 115310 115310 1 95 3 0.032 TGGAGCGGGAA

MT946551.1 115373 115373 1 62 2 0.032 GTGTCCGGAGT

MT946551.1 116407 116407 1 95 3 0.032 AAAACCGACCT

MT946551.1 118108 118108 1 95 3 0.032 AGAATCGTATA

MT946551.1 129273 129273 1 93 3 0.032 ATTGACGATGT

MT946551.1 136039 136039 1 62 2 0.032 GTGGTCGCCAT

MT946551.1 136839 136839 1 95 3 0.032 ATCATCGTGTG

MT946551.1 137584 137584 1 95 3 0.032 GACACCGTCAC

MT946551.1 138609 138609 1 94 3 0.032 TAACTCGTTTT

MT946551.1 142243 142243 1 95 3 0.032 TAGTTCGTCAT

MT946551.1 143223 143223 1 93 3 0.032 GTCTGCGAAAC

MT946551.1 150284 150284 1 63 2 0.032 AGGTACGGGAT

MT946551.1 159210 159210 1 31 1 0.032 TCTATCGACAT

MT946551.1 229 229 1 32 1 0.031 TGAATCGATGA

MT946551.1 6130 6130 1 64 2 0.031 AACATCGTTTT

MT946551.1 8808 8817 2 128 4 0.031 TTTTACGAATTCTACGGGAT

MT946551.1 26477 26477 1 64 2 0.031 GAGGACGGACA

MT946551.1 33509 33509 1 65 2 0.031 GTTTGCGGATA

MT946551.1 38227 38227 1 96 3 0.031 CATGCCGCCTA

MT946551.1 51688 51688 1 98 3 0.031 TACCTCGTACT

MT946551.1 56755 56755 1 64 2 0.031 TCACTCGTGTC

MT946551.1 59068 59068 1 98 3 0.031 ATGATCGATTA

MT946551.1 63987 63987 1 65 2 0.031 GCTCCCGGTAC

MT946551.1 66867 66867 1 98 3 0.031 GAATACGCCAA

MT946551.1 68023 68023 1 96 3 0.031 CTTTTCGATGC

MT946551.1 72083 72083 1 97 3 0.031 TTTAGCGGTAT

MT946551.1 73216 73216 1 96 3 0.031 TTCATCGATCT

MT946551.1 73441 73441 1 96 3 0.031 ATATACGTACT

MT946551.1 75509 75509 1 64 2 0.031 ACTGACGGATG

MT946551.1 75698 75698 1 64 2 0.031 AATAGCGTTAA

MT946551.1 89098 89098 1 96 3 0.031 CTAGACGTTTA

MT946551.1 94955 94955 1 96 3 0.031 AATACCGAATA

MT946551.1 95391 95391 1 98 3 0.031 TCATGCGTGGA

MT946551.1 96809 96809 1 97 3 0.031 CTAGACGTATA

MT946551.1 98115 98115 1 96 3 0.031 TGATGCGCTAA

MT946551.1 102923 102923 1 64 2 0.031 ACCTCCGACTA

MT946551.1 108080 108080 1 98 3 0.031 TCTTTCGATGT

MT946551.1 108350 108350 1 96 3 0.031 CAAGACGAACA

MT946551.1 110141 110141 1 64 2 0.031 GATGTCGGAAA

MT946551.1 113669 113669 1 97 3 0.031 TGTATCGTATT

MT946551.1 113752 113752 1 98 3 0.031 CAGAACGAATT

MT946551.1 119678 119678 1 96 3 0.031 TTCTACGAAGA

MT946551.1 123320 123320 1 97 3 0.031 GCATGCGACTA

MT946551.1 124531 124534 2 192 6 0.031 ATTATCGACGATTA

MT946551.1 125171 125171 1 96 3 0.031 TAAAACGAAAA

MT946551.1 129035 129035 1 98 3 0.031 GAACACGTATG

MT946551.1 131023 131023 1 98 3 0.031 AAAGTCGTTTA

MT946551.1 143718 143721 2 192 6 0.031 TGTATCGACGGTAA

MT946551.1 148409 148409 1 64 2 0.031 AGTGCCGTATA

MT946551.1 445 445 1 33 1 0.03 GATGTCGCTGT

MT946551.1 1626 1629 2 66 2 0.03 CTTACCGTCGTCCA

MT946551.1 5494 5494 1 66 2 0.03 ATTATCGATTT

MT946551.1 6043 6043 1 67 2 0.03 TATATCGATGT

MT946551.1 7371 7377 2 132 4 0.03 GATGTCGAATTCGTGCT

MT946551.1 12795 12795 1 67 2 0.03 TGGGTCGTATT

MT946551.1 17560 17560 1 101 3 0.03 CTGTTCGATAT

MT946551.1 23183 23183 1 100 3 0.03 ATCATCGACAT

MT946551.1 24307 24307 1 100 3 0.03 CTAGTCGTACT

MT946551.1 29158 29158 1 66 2 0.03 TGATTCGCAAA

MT946551.1 34896 34896 1 67 2 0.03 GATCACGAGTT

MT946551.1 38692 38692 1 99 3 0.03 GATATCGAATC

MT946551.1 39512 39512 1 66 2 0.03 AGTCACGACTG

MT946551.1 42458 42458 1 100 3 0.03 TCTATCGACTC

MT946551.1 45570 45570 1 100 3 0.03 TCTTTCGTATA

MT946551.1 54712 54718 2 132 4 0.03 AAATACGTAAACGGATG

MT946551.1 69946 69946 1 100 3 0.03 AATATCGGTAG

MT946551.1 72106 72106 1 101 3 0.03 TCTCTCGATTT

MT946551.1 75139 75139 1 99 3 0.03 AAGGACGTAGA

MT946551.1 77054 77054 1 67 2 0.03 TATCACGTCAT

MT946551.1 96439 96439 1 100 3 0.03 TCAATCGCTTC

MT946551.1 97396 97396 1 100 3 0.03 ATCAACGTATA

MT946551.1 100329 100329 1 67 2 0.03 AATTGCGGACT

MT946551.1 100828 100828 1 67 2 0.03 AAACTCGGATA

MT946551.1 111150 111153 2 132 4 0.03 TTTGACGACGAGTT

MT946551.1 112731 112731 1 100 3 0.03 GCTCTCGTACA

MT946551.1 113421 113421 1 99 3 0.03 CATTACGATCT

MT946551.1 113849 113849 1 67 2 0.03 AGCCACGTTCA

MT946551.1 114048 114048 1 100 3 0.03 TCTGTCGAAAA

MT946551.1 114321 114321 1 101 3 0.03 TCATACGATAT

MT946551.1 123929 123929 1 101 3 0.03 GATAACGAATC

MT946551.1 130936 130936 1 99 3 0.03 ATTATCGGTGT

MT946551.1 132196 132196 1 100 3 0.03 GACTACGATAC

MT946551.1 139087 139087 1 99 3 0.03 AAGGTCGGTGA

MT946551.1 152633 152633 1 67 2 0.03 CAACACGATAT

MT946551.1 159300 159300 1 33 1 0.03 AGTTACGAAGA

MT946551.1 12463 12463 1 69 2 0.029 TTATCCGGATT

MT946551.1 25874 25874 1 69 2 0.029 AATCACGTACA

MT946551.1 31186 31192 2 138 4 0.029 AGGTTCGTCAGCGGCTC

MT946551.1 33915 33915 1 69 2 0.029 ATTAACGGTAA

MT946551.1 35409 35409 1 68 2 0.029 TAGACCGTTAA

MT946551.1 36552 36552 1 70 2 0.029 AAGAACGGAGA

MT946551.1 41366 41366 1 69 2 0.029 TCAACCGTTAG

MT946551.1 44816 44816 1 69 2 0.029 CTCTCCGAGTG

MT946551.1 64458 64458 1 68 2 0.029 TCAGACGCTGT

MT946551.1 65218 65218 1 68 2 0.029 ATAGCCGATAG

MT946551.1 72322 72322 1 103 3 0.029 TGTATCGAAAA

MT946551.1 88262 88262 1 103 3 0.029 GTGCACGTTTA

MT946551.1 92298 92298 1 102 3 0.029 CCCACCGATCA

MT946551.1 93613 93613 1 104 3 0.029 AGTATCGCCCT

MT946551.1 112196 112196 1 103 3 0.029 GAGATCGAAAA

MT946551.1 114354 114356 2 70 2 0.029 GTAAACGCGATAA

MT946551.1 117157 117157 1 103 3 0.029 ATATTCGAAAA

MT946551.1 121049 121049 1 69 2 0.029 TATAGCGTTTA

MT946551.1 127738 127738 1 102 3 0.029 CCAAACGAGTT

MT946551.1 142318 142318 1 105 3 0.029 ACTATCGTACT

MT946551.1 152083 152083 1 69 2 0.029 TGTAACGATGA

MT946551.1 153185 153185 1 70 2 0.029 AAATACGATGG

MT946551.1 153867 153867 1 70 2 0.029 TTTATCGCACA

MT946551.1 153941 153941 1 70 2 0.029 ATAAACGTGTT

MT946551.1 159243 159243 1 35 1 0.029 GACAGCGACAT

MT946551.1 159459 159459 1 34 1 0.029 CTCATCGATTC

MT946551.1 8008 8008 1 71 2 0.028 ATAATCGTGTA

MT946551.1 13135 13135 1 71 2 0.028 CCTAACGATGA

MT946551.1 13795 13798 2 142 4 0.028 ATGACCGACGATAA

MT946551.1 16083 16083 1 71 2 0.028 TAGTCCGTATT

MT946551.1 27032 27032 1 72 2 0.028 TCTAACGTATT

MT946551.1 33242 33242 1 109 3 0.028 ACAATCGTACA

MT946551.1 44012 44012 1 107 3 0.028 TCCATCGCTTC

MT946551.1 44124 44124 1 106 3 0.028 ATAGACGAACA

MT946551.1 51332 51341 2 142 4 0.028 TATAACGGAACAAACGCTAC

MT946551.1 67115 67115 1 72 2 0.028 AAAGCCGTAAT

MT946551.1 68357 68357 1 71 2 0.028 GTATTCGTCAA

MT946551.1 75969 75969 1 71 2 0.028 TTCTTCGGAGA

MT946551.1 78892 78892 1 72 2 0.028 GATGTCGAAGA

MT946551.1 81532 81532 1 71 2 0.028 TACAACGGTAC

MT946551.1 81938 81938 1 72 2 0.028 GAGACCGATAC

MT946551.1 90144 90144 1 109 3 0.028 AGTATCGATTA

MT946551.1 100630 100636 2 144 4 0.028 AGATGCGTTGACGCTGT

MT946551.1 100648 100648 1 71 2 0.028 AAAGACGTCAC

MT946551.1 102480 102480 1 71 2 0.028 TCAAACGCTGA

MT946551.1 107746 107746 1 109 3 0.028 TCTATCGACAT

MT946551.1 108681 108681 1 71 2 0.028 TGAACCGGGAT

MT946551.1 120996 120996 1 72 2 0.028 TTTGACGATGA

MT946551.1 126926 126926 1 108 3 0.028 TGATACGATAC

MT946551.1 128838 128838 1 108 3 0.028 ATTATCGAGGA

MT946551.1 129288 129288 1 106 3 0.028 AATGCCGGTAT

MT946551.1 135436 135436 1 106 3 0.028 ACTGACGTATT

MT946551.1 135550 135550 1 109 3 0.028 TTGAACGAGGG

MT946551.1 142902 142902 1 106 3 0.028 CCTAACGAAAT

MT946551.1 153811 153811 1 71 2 0.028 ATAGCCGCTCT

MT946551.1 158594 158597 2 72 2 0.028 TCTAGCGTCGACAT

MT946551.1 158732 158732 1 36 1 0.028 CCACCCGCTTT

MT946551.1 159076 159076 1 36 1 0.028 TCAGTCGAGGA

MT946551.1 388 388 1 37 1 0.027 ATCTTCGTAAC

MT946551.1 956 956 1 37 1 0.027 AAAAGCGGGTG

MT946551.1 7653 7653 1 73 2 0.027 TTCTACGATAG

MT946551.1 10434 10441 2 146 4 0.027 TTAACCGCATCTCGTCTA

MT946551.1 12276 12276 1 75 2 0.027 AATCTCGTTTA

MT946551.1 12450 12450 1 73 2 0.027 CACTGCGATTG

MT946551.1 16801 16801 1 74 2 0.027 TCTAACGGTTT

MT946551.1 38014 38014 1 74 2 0.027 TGCAGCGGCTT

MT946551.1 40331 40338 2 150 4 0.027 AACATCGTTGACCGATTA

MT946551.1 60854 60854 1 73 2 0.027 CTCTACGGGCT

MT946551.1 63205 63205 1 74 2 0.027 ATAATCGGCCC

MT946551.1 64059 64063 2 148 4 0.027 ATTGACGGCCGCCAT

MT946551.1 66550 66550 1 74 2 0.027 GAAGGCGGTAA

MT946551.1 66713 66713 1 73 2 0.027 ATCACCGTTAA

MT946551.1 67400 67403 2 146 4 0.027 GCAAACGACGTAGA

MT946551.1 69377 69377 1 74 2 0.027 CTTCTCGCTAG

MT946551.1 73649 73649 1 74 2 0.027 TTGAACGGTAT

MT946551.1 74354 74354 1 73 2 0.027 TAAAGCGGATA

MT946551.1 78192 78192 1 74 2 0.027 ATTGGCGATTG

MT946551.1 80550 80550 1 75 2 0.027 ATCCCCGGTAA

MT946551.1 82073 82076 2 148 4 0.027 AAGTTCGTCGAAGA

MT946551.1 90607 90617 2 150 4 0.027 ACATGCGTTCCCTCCCGGATA

MT946551.1 90862 90862 1 73 2 0.027 GTTGTCGGCCT

MT946551.1 97809 97809 1 75 2 0.027 ATCTTCGGTAT

MT946551.1 102690 102690 1 75 2 0.027 TAGTACGGACT

MT946551.1 115242 115242 1 74 2 0.027 TTCTTCGGTAA

MT946551.1 119664 119664 1 75 2 0.027 AACCACGAATA

MT946551.1 119822 119832 2 148 4 0.027 GTCTTCGTTTATTACCGTACC

MT946551.1 125562 125562 1 113 3 0.027 TACAGCGCATG

MT946551.1 126601 126601 1 112 3 0.027 TTAAACGATGG

MT946551.1 127197 127197 1 110 3 0.027 TGGTTCGATAT

MT946551.1 131680 131680 1 111 3 0.027 TCATACGTAAT

MT946551.1 141250 141250 1 112 3 0.027 TGATTCGCTTC

MT946551.1 149233 149233 1 73 2 0.027 TTGGTCGTCCT

MT946551.1 149761 149761 1 74 2 0.027 GAAAACGAGCC

MT946551.1 152194 152194 1 73 2 0.027 CTTATCGCATA

MT946551.1 153308 153308 1 74 2 0.027 CTCAACGAGTT

MT946551.1 158959 158959 1 37 1 0.027 GGAATCGATGT

MT946551.1 159441 159441 1 37 1 0.027 GCAACCGATGA

MT946551.1 4212 4212 1 38 1 0.026 CATGCCGCCAC

MT946551.1 7247 7247 1 76 2 0.026 CTGGGCGTATA

MT946551.1 7285 7285 1 76 2 0.026 TCATTCGAAAT

MT946551.1 9671 9671 1 77 2 0.026 TTTCACGTACC

MT946551.1 13293 13293 1 77 2 0.026 CAGTTCGTTTG

MT946551.1 16110 16110 1 77 2 0.026 ACACTCGACAC

MT946551.1 25989 25989 1 78 2 0.026 TTCATCGTTTT

MT946551.1 29339 29339 1 78 2 0.026 AGTTTCGTAAT

MT946551.1 37941 37941 1 78 2 0.026 CTTTACGAATT

MT946551.1 49436 49436 1 78 2 0.026 AACTGCGTTAA

MT946551.1 51252 51252 1 78 2 0.026 ATTTACGAATT

MT946551.1 52343 52346 2 154 4 0.026 ATAATCGTCGCTAT

MT946551.1 55592 55592 1 77 2 0.026 AACCACGCAAG

MT946551.1 59718 59718 1 76 2 0.026 AACACCGGAAC

MT946551.1 62120 62120 1 76 2 0.026 ATATGCGGGAA

MT946551.1 65440 65440 1 77 2 0.026 CTACTCGTTTG

MT946551.1 77461 77469 2 152 4 0.026 AAATACGAAGGTCCGTTTA

MT946551.1 88739 88739 1 78 2 0.026 TAATGCGGATT

MT946551.1 99587 99587 1 77 2 0.026 TTTTACGTTAT

MT946551.1 100568 100568 1 76 2 0.026 CACTACGATTC

MT946551.1 108733 108741 2 156 4 0.026 CCTTCCGTAAAGACGAGTC

MT946551.1 118949 118949 1 78 2 0.026 CTCTGCGTTAT

MT946551.1 119847 119847 1 78 2 0.026 CTAAACGCTAT

MT946551.1 120690 120690 1 78 2 0.026 TGTAACGATTC

MT946551.1 130516 130516 1 77 2 0.026 CAACACGTGCA

MT946551.1 144190 144190 1 78 2 0.026 AGTAACGCCAT

MT946551.1 144927 144927 1 76 2 0.026 TTCAACGCAGA

MT946551.1 150256 150256 1 76 2 0.026 TATAACGTAAC

MT946551.1 152267 152267 1 76 2 0.026 GATATCGATGA

MT946551.1 155164 155171 2 152 4 0.026 ATCAACGAATATCGATAA

MT946551.1 158876 158876 1 38 1 0.026 TATATCGTACT

MT946551.1 8697 8697 1 80 2 0.025 TTCCACGTAGT

MT946551.1 10065 10065 1 80 2 0.025 TGAAACGACCA

MT946551.1 10780 10780 1 79 2 0.025 GTAGACGATAG

MT946551.1 16375 16375 1 80 2 0.025 AATATCGTAGA

MT946551.1 25262 25262 1 81 2 0.025 GATTCCGTATT

MT946551.1 28060 28060 1 80 2 0.025 TAACTCGTTTT

MT946551.1 35890 35896 2 158 4 0.025 TTTACCGGTCCCGTTCC

MT946551.1 36848 36848 1 80 2 0.025 TCTTTCGCTAT

MT946551.1 48921 48921 1 79 2 0.025 CTTGACGACCT

MT946551.1 52496 52496 1 81 2 0.025 AGTTTCGTACA

MT946551.1 55069 55069 1 81 2 0.025 TGTCTCGAGGC

MT946551.1 59757 59757 1 81 2 0.025 TGCTGCGTTAA

MT946551.1 62237 62237 1 81 2 0.025 AGAATCGTATA

MT946551.1 66677 66677 1 79 2 0.025 CAGACCGCTAG

MT946551.1 67576 67579 2 158 4 0.025 AAATTCGACGTTCA

MT946551.1 70618 70628 2 158 4 0.025 AAAGGCGGCAAAGGTCGATCC

MT946551.1 74944 74944 1 79 2 0.025 AAATGCGTGCA

MT946551.1 78481 78481 1 79 2 0.025 GCTACCGTCAT

MT946551.1 83906 83906 1 81 2 0.025 CTAGACGACAG

MT946551.1 84897 84897 1 80 2 0.025 TGTATCGAATA

MT946551.1 85001 85001 1 79 2 0.025 ACTTTCGTAAA

MT946551.1 85448 85448 1 80 2 0.025 TAATGCGTATG

MT946551.1 87153 87153 1 81 2 0.025 ATAGACGACTA

MT946551.1 88445 88445 1 79 2 0.025 TTCTCCGTTAG

MT946551.1 89115 89115 1 81 2 0.025 AATAGCGATTG

MT946551.1 96019 96019 1 81 2 0.025 GTAACCGTTGG

MT946551.1 100288 100288 1 79 2 0.025 GGGCTCGTTCT

MT946551.1 100708 100708 1 81 2 0.025 ACTATCGCATC

MT946551.1 100869 100869 1 79 2 0.025 TGAACCGTATT

MT946551.1 101968 101968 1 81 2 0.025 TAGAACGATTA

MT946551.1 103134 103134 1 79 2 0.025 ATAGCCGTAGA

MT946551.1 107232 107232 1 80 2 0.025 ATGATCGTTAC

MT946551.1 115725 115725 1 79 2 0.025 CCAATCGTGAT

MT946551.1 117492 117492 1 79 2 0.025 TTCTCCGCATC

MT946551.1 128635 128635 1 81 2 0.025 AATAACGAGTA

MT946551.1 135502 135507 2 160 4 0.025 GTATACGAACCGGGAA

MT946551.1 142749 142749 1 81 2 0.025 AGATACGGTAA

MT946551.1 144436 144436 1 81 2 0.025 AATAGCGTATC

MT946551.1 147125 147125 1 80 2 0.025 GATAACGATGG

MT946551.1 148313 148313 1 81 2 0.025 ATTGTCGCACT

MT946551.1 148499 148499 1 81 2 0.025 GTAGACGTAAG

MT946551.1 149541 149544 2 158 4 0.025 TATTCCGGCGTATG

MT946551.1 150360 150360 1 80 2 0.025 TATTGCGTGTA

MT946551.1 150802 150802 1 80 2 0.025 AATTGCGGTAT

MT946551.1 157863 157869 2 80 2 0.025 AAAATCGATTCCGTCCA

MT946551.1 158147 158147 1 40 1 0.025 TCTCCCGATCA

MT946551.1 158639 158639 1 40 1 0.025 GACTACGAAAC

MT946551.1 4089 4089 1 42 1 0.024 ACCTCCGTTAA

MT946551.1 14057 14057 1 85 2 0.024 ATCATCGCATA

MT946551.1 16321 16321 1 84 2 0.024 TTTTTCGTGTA

MT946551.1 22704 22706 2 170 4 0.024 AAATTCGCGCCCA

MT946551.1 25150 25150 1 82 2 0.024 TGTTTCGAAAC

MT946551.1 27748 27748 1 82 2 0.024 GTATACGATCT

MT946551.1 34045 34045 1 84 2 0.024 TTAAACGATTT

MT946551.1 36737 36737 1 83 2 0.024 TTCATCGTTGT

MT946551.1 41318 41318 1 85 2 0.024 TGTAGCGTAGC

MT946551.1 41893 41893 1 84 2 0.024 CAATGCGTCCA

MT946551.1 47209 47209 1 84 2 0.024 TTAGACGCTTA

MT946551.1 48038 48038 1 85 2 0.024 GCATTCGTTTA

MT946551.1 49516 49516 1 84 2 0.024 ATTGGCGGTGT

MT946551.1 52865 52865 1 82 2 0.024 GTATTCGTTTT

MT946551.1 53387 53387 1 85 2 0.024 CTAGTCGGTAT

MT946551.1 54527 54527 1 85 2 0.024 CAGTACGTTCT

MT946551.1 58692 58692 1 82 2 0.024 ACAATCGTATA

MT946551.1 60895 60895 1 84 2 0.024 TAACACGACTG

MT946551.1 61776 61776 1 82 2 0.024 CATAACGAGAA

MT946551.1 67163 67163 1 82 2 0.024 AAACACGATAT

MT946551.1 74023 74023 1 83 2 0.024 GATAACGAAAA

MT946551.1 76338 76338 1 83 2 0.024 GATTACGAATA

MT946551.1 79254 79254 1 82 2 0.024 TCTCTCGAAGA

MT946551.1 81863 81863 1 85 2 0.024 TCAAGCGAATT

MT946551.1 85503 85503 1 85 2 0.024 GGTATCGAATC

MT946551.1 85573 85573 1 84 2 0.024 AAACACGAATA

MT946551.1 85862 85862 1 85 2 0.024 ATTGGCGTTGG

MT946551.1 86731 86731 1 83 2 0.024 TTAGACGGTTG

MT946551.1 89926 89926 1 85 2 0.024 AATTCCGTATC

MT946551.1 94544 94544 1 83 2 0.024 CATATCGTAAC

MT946551.1 95042 95042 1 84 2 0.024 CATATCGGTAA

MT946551.1 100345 100345 1 82 2 0.024 TATATCGCATA

MT946551.1 105329 105329 1 82 2 0.024 TGAGTCGATAC

MT946551.1 109456 109456 1 82 2 0.024 TATACCGAGAA

MT946551.1 109541 109541 1 85 2 0.024 TTAATCGCATT

MT946551.1 116367 116367 1 85 2 0.024 ACAGCCGCATA

MT946551.1 121071 121071 1 83 2 0.024 ATGTGCGAATA

MT946551.1 121448 121448 1 82 2 0.024 TTTATCGCCTA

MT946551.1 124702 124702 1 83 2 0.024 TAAAGCGGATA

MT946551.1 132867 132870 2 170 4 0.024 ATAGACGACGATTT

MT946551.1 139693 139693 1 84 2 0.024 GATCTCGTGTG

MT946551.1 147392 147392 1 83 2 0.024 TTCTTCGTTAT

MT946551.1 150238 150238 1 85 2 0.024 ATATACGGTGG

MT946551.1 1728 1728 1 44 1 0.023 AAAACCGAACA

MT946551.1 2097 2097 1 43 1 0.023 ACAGGCGAATA

MT946551.1 2731 2731 1 44 1 0.023 TATGACGTCTC

MT946551.1 9184 9193 2 172 4 0.023 GAGGACGTCATGATCGCATC

MT946551.1 10193 10193 1 87 2 0.023 GTCATCGATTG

MT946551.1 11620 11627 2 172 4 0.023 ATAAACGAAGATCGTTGC

MT946551.1 13639 13639 1 87 2 0.023 ATGAGCGAAGG

MT946551.1 14679 14679 1 86 2 0.023 ATATACGTTGA

MT946551.1 15669 15669 1 87 2 0.023 TCCACCGATGA

MT946551.1 15914 15914 1 87 2 0.023 TTCTTCGCTTT

MT946551.1 16870 16870 1 87 2 0.023 ACCCTCGTCTC

MT946551.1 24019 24019 1 87 2 0.023 GCCAGCGGGGG

MT946551.1 30091 30091 1 87 2 0.023 TAATGCGATAG

MT946551.1 30122 30122 1 87 2 0.023 CCAAACGCTTT

MT946551.1 30384 30384 1 87 2 0.023 CATCTCGTTTA

MT946551.1 32015 32015 1 86 2 0.023 CATTACGAGAC

MT946551.1 33639 33639 1 87 2 0.023 TTAAACGAAAG

MT946551.1 34620 34620 1 44 1 0.023 AGACACGGTAG

MT946551.1 36280 36280 1 87 2 0.023 ACTGCCGAGTT

MT946551.1 38713 38713 1 87 2 0.023 TTTAGCGAAAC

MT946551.1 40803 40803 1 86 2 0.023 TGGGACGTCTA

MT946551.1 53179 53179 1 86 2 0.023 AAATACGAACT

MT946551.1 54129 54129 1 88 2 0.023 GGAAGCGGAAC

MT946551.1 55961 55961 1 86 2 0.023 AAGAACGAATG

MT946551.1 58211 58211 1 88 2 0.023 TGATGCGTTTT

MT946551.1 61511 61511 1 86 2 0.023 CACCTCGTTTG

MT946551.1 73549 73549 1 87 2 0.023 AATACCGATGT

MT946551.1 73961 73961 1 87 2 0.023 TCTATCGTAGT

MT946551.1 74485 74485 1 86 2 0.023 CCAAACGAGCC

MT946551.1 86330 86330 1 86 2 0.023 GGTTTCGACTC

MT946551.1 86754 86760 2 174 4 0.023 ATCAACGATATCGATCC

MT946551.1 90195 90195 1 87 2 0.023 TTTTTCGTTTA

MT946551.1 91017 91017 1 88 2 0.023 AATATCGGTCT

MT946551.1 91086 91086 1 86 2 0.023 TGATTCGGCTG

MT946551.1 91368 91368 1 86 2 0.023 AGAAACGATAC

MT946551.1 95666 95666 1 86 2 0.023 GACCACGGCTT

MT946551.1 96301 96301 1 87 2 0.023 GGAACCGGCTG

MT946551.1 100010 100010 1 88 2 0.023 GCTATCGTTTT

MT946551.1 102062 102062 1 87 2 0.023 GATTTCGTTAG

MT946551.1 103538 103538 1 88 2 0.023 ATCTGCGATGT

MT946551.1 105070 105070 1 86 2 0.023 ATTATCGATGT

MT946551.1 106598 106598 1 86 2 0.023 TAATACGAATA

MT946551.1 108524 108524 1 86 2 0.023 GAAGTCGTCAA

MT946551.1 119550 119550 1 87 2 0.023 TAAGACGATAC

MT946551.1 121880 121880 1 88 2 0.023 CACATCGTGAC

MT946551.1 129850 129850 1 87 2 0.023 AATTACGGTTG

MT946551.1 135808 135808 1 87 2 0.023 ATTCCCGATTT

MT946551.1 136561 136561 1 86 2 0.023 GTAACCGATAA

MT946551.1 138875 138875 1 87 2 0.023 TTTACCGAACA

MT946551.1 139100 139106 2 176 4 0.023 TTGATCGGAATCGAAAT

MT946551.1 149689 149689 1 88 2 0.023 TCATTCGTTCA

MT946551.1 157800 157800 1 44 1 0.023 AGGATCGACTC

MT946551.1 2010 2010 1 45 1 0.022 TATAGCGCTTT

MT946551.1 3611 3611 1 46 1 0.022 GTCAACGTCTA

MT946551.1 10013 10013 1 91 2 0.022 ATTTTCGATAG

MT946551.1 15966 15966 1 91 2 0.022 TTCATCGCTTT

MT946551.1 16814 16814 1 89 2 0.022 TCACTCGGTGG

MT946551.1 17296 17296 1 93 2 0.022 TTTTTCGTCTG

MT946551.1 18077 18077 1 92 2 0.022 ATAACCGGCAA

MT946551.1 19197 19197 1 89 2 0.022 TTTTTCGATTT

MT946551.1 19708 19708 1 89 2 0.022 ACTGCCGGATA

MT946551.1 20597 20597 1 91 2 0.022 GCATACGAATT

MT946551.1 22370 22370 1 90 2 0.022 TTATACGCATC

MT946551.1 22860 22860 1 90 2 0.022 ACTTACGATAC

MT946551.1 28519 28519 1 91 2 0.022 AATGTCGAATA

MT946551.1 28606 28606 1 89 2 0.022 CTTTTCGTATG

MT946551.1 28900 28900 1 91 2 0.022 ATATTCGAACA

MT946551.1 29111 29111 1 93 2 0.022 ACTAACGTAGA

MT946551.1 35654 35654 1 92 2 0.022 ATCTTCGAAAG

MT946551.1 36953 36953 1 91 2 0.022 GGCAACGATGT

MT946551.1 42689 42689 1 93 2 0.022 AACATCGATAA

MT946551.1 46446 46446 1 93 2 0.022 TGATACGGCTA

MT946551.1 47256 47256 1 92 2 0.022 GATATCGCTTT

MT946551.1 47618 47618 1 92 2 0.022 TGTATCGAATG

MT946551.1 48474 48474 1 93 2 0.022 ACATTCGAAAA

MT946551.1 49969 49969 1 92 2 0.022 TAAGACGTCAC

MT946551.1 52276 52276 1 89 2 0.022 TAATACGTGTA

MT946551.1 54167 54174 2 184 4 0.022 TTATTCGGTAACCGGTGG

MT946551.1 55447 55447 1 91 2 0.022 TTAGACGATGT

MT946551.1 55666 55666 1 89 2 0.022 GATATCGATGT

MT946551.1 55689 55696 2 180 4 0.022 ATCTTCGATCTTCGGATA

MT946551.1 56221 56221 1 93 2 0.022 GAGTTCGAATA

MT946551.1 56315 56315 1 91 2 0.022 TGATACGTCAT

MT946551.1 60049 60049 1 93 2 0.022 GTTATCGGTGT

MT946551.1 62657 62657 1 92 2 0.022 TAAACCGATAG

MT946551.1 64350 64350 1 45 1 0.022 CCACACGGTAA

MT946551.1 64947 64947 1 91 2 0.022 AACATCGTATC

MT946551.1 67013 67013 1 93 2 0.022 GCTACCGAAGG

MT946551.1 67607 67607 1 93 2 0.022 ATCAACGTAGA

MT946551.1 69775 69775 1 93 2 0.022 ATACACGTATA

MT946551.1 73910 73910 1 91 2 0.022 ATATACGAAAA

MT946551.1 73977 73977 1 90 2 0.022 ACAATCGTATA

MT946551.1 74302 74311 3 135 3 0.022 ATTATCGCGATATCCGTTAA

MT946551.1 74628 74628 1 90 2 0.022 GAAAGCGTGGC

MT946551.1 74718 74718 1 93 2 0.022 AGAAGCGATCT

MT946551.1 75951 75951 1 93 2 0.022 TCTCTCGCAAT

MT946551.1 80125 80125 1 90 2 0.022 TATAACGAAGT

MT946551.1 81174 81174 1 90 2 0.022 TGAAACGAACA

MT946551.1 82194 82194 1 93 2 0.022 TTATGCGGTGC

MT946551.1 82905 82905 1 93 2 0.022 TTGTACGATAA

MT946551.1 86558 86558 1 90 2 0.022 AGACACGCATG

MT946551.1 86636 86636 1 93 2 0.022 CCAAGCGATAT

MT946551.1 92227 92227 1 93 2 0.022 TATTGCGGCAT

MT946551.1 93019 93019 1 90 2 0.022 GATATCGAATA

MT946551.1 93819 93828 2 186 4 0.022 TGTCTCGAAATCATCGACAT

MT946551.1 95135 95135 1 89 2 0.022 GTCACCGATTC

MT946551.1 95513 95513 1 93 2 0.022 GTCATCGTCTA

MT946551.1 97750 97753 2 186 4 0.022 GTAGTCGACGCTCT

MT946551.1 98576 98576 1 91 2 0.022 AAAATCGATTT

MT946551.1 100165 100170 2 178 4 0.022 TCATTCGAAGCGTCAA

MT946551.1 101197 101197 1 93 2 0.022 ATCAACGAGTT

MT946551.1 102041 102041 1 93 2 0.022 TGAGGCGTTTA

MT946551.1 102968 102968 1 90 2 0.022 CTCTGCGGAGA

MT946551.1 104833 104833 1 89 2 0.022 GCCATCGTTAA

MT946551.1 107244 107254 2 180 4 0.022 AAAGACGTATAATGCCGTATC

MT946551.1 112530 112530 1 90 2 0.022 AAGAACGCATT

MT946551.1 116219 116219 1 92 2 0.022 TAGAACGAGTC

MT946551.1 123395 123395 1 91 2 0.022 TTTAACGCTAC

MT946551.1 123484 123484 1 93 2 0.022 ATTGTCGTTTA

MT946551.1 124008 124008 1 90 2 0.022 ATGATCGTAAT

MT946551.1 124094 124094 1 91 2 0.022 CCTTTCGTAAA

MT946551.1 134055 134055 1 93 2 0.022 CAGACCGAATT

MT946551.1 137326 137326 1 92 2 0.022 GATGCCGGTAC

MT946551.1 138417 138420 2 178 4 0.022 TTCATCGTCGTGTG

MT946551.1 140043 140043 1 93 2 0.022 CATCACGTACT

MT946551.1 140379 140379 1 89 2 0.022 TGAAACGTTTG

MT946551.1 143907 143907 1 92 2 0.022 ATGGGCGTCAT

MT946551.1 145781 145781 1 89 2 0.022 TTTCTCGAAAA

MT946551.1 147078 147078 1 90 2 0.022 CAACTCGTTAT

MT946551.1 151019 151021 2 90 2 0.022 ACACACGCGAACT

MT946551.1 152618 152618 1 46 1 0.022 ACTTCCGACTA

MT946551.1 154602 154602 1 45 1 0.022 AATGTCGTGGA

MT946551.1 157678 157678 1 45 1 0.022 TAAAGCGCTAT

MT946551.1 1926 1935 2 94 2 0.021 TGTGGCGTTTGCTTCGTTTA

MT946551.1 3194 3201 2 94 2 0.021 GCAGACGTAAGTCGAAAT

MT946551.1 4279 4279 1 47 1 0.021 AATCACGAGTA

MT946551.1 18791 18791 1 94 2 0.021 CTCAGCGATAG

MT946551.1 21487 21487 1 96 2 0.021 ACTAACGTATA

MT946551.1 21561 21561 1 95 2 0.021 ATGCTCGGCAA

MT946551.1 30331 30331 1 95 2 0.021 GATGACGTAAC

MT946551.1 36148 36148 1 96 2 0.021 TGCTTCGTAAA

MT946551.1 43890 43890 1 94 2 0.021 TCCACCGAAAG

MT946551.1 44666 44666 1 97 2 0.021 AAACTCGCTTA

MT946551.1 47568 47568 1 97 2 0.021 CAATACGAGTC

MT946551.1 49168 49168 1 95 2 0.021 TGCCCCGACTC

MT946551.1 53944 53946 2 194 4 0.021 ATGAGCGCGTCCC

MT946551.1 54005 54017 3 282 6 0.021 TATGGCGTATACAACGTCGCATA

MT946551.1 56075 56075 1 94 2 0.021 ATATGCGCCTA

MT946551.1 57084 57084 1 97 2 0.021 CTCACCGATGA

MT946551.1 60433 60438 2 194 4 0.021 TTTTACGGTACGTCTA

MT946551.1 68335 68338 2 194 4 0.021 ATTGGCGCCGTTTA

MT946551.1 68807 68807 1 96 2 0.021 AATTTCGAATT

MT946551.1 69590 69590 1 94 2 0.021 TTTATCGACTT

MT946551.1 72804 72804 1 96 2 0.021 CCTGACGTCTA

MT946551.1 74745 74754 2 188 4 0.021 GCTACCGACAATATCGTTAA

MT946551.1 75494 75494 1 97 2 0.021 TAAACCGCTAT

MT946551.1 76282 76291 2 190 4 0.021 ATACTCGTATCAGTCGAGAT

MT946551.1 78430 78430 1 97 2 0.021 ATCATCGACTG

MT946551.1 86625 86625 1 94 2 0.021 TCATTCGCATT

MT946551.1 89514 89514 1 97 2 0.021 CAATTCGTATA

MT946551.1 94584 94584 1 95 2 0.021 ACCAACGTAGG

MT946551.1 94703 94703 1 94 2 0.021 ATCATCGTTAC

MT946551.1 95405 95408 2 194 4 0.021 TACTTCGTCGATGG

MT946551.1 97204 97204 1 96 2 0.021 TCCTTCGATAG

MT946551.1 101835 101835 1 94 2 0.021 AACATCGATGC

MT946551.1 103274 103274 1 95 2 0.021 AAGTACGATGT

MT946551.1 107628 107628 1 96 2 0.021 TACCTCGTTCT

MT946551.1 108912 108912 1 95 2 0.021 CTTGGCGTGTG

MT946551.1 112610 112610 1 94 2 0.021 TATTGCGACAT

MT946551.1 114307 114307 1 97 2 0.021 TTAGACGTAGT

MT946551.1 115667 115675 2 190 4 0.021 GAGCTCGGATATACGAAAT

MT946551.1 118460 118460 1 94 2 0.021 TTAAACGCAGT

MT946551.1 125066 125066 1 96 2 0.021 TTGTACGGTAG

MT946551.1 126438 126438 1 96 2 0.021 GGATGCGGAAC

MT946551.1 128962 128962 1 97 2 0.021 CTAATCGTAAT

MT946551.1 136419 136425 2 192 4 0.021 TAAAGCGTGTGCGTGTA

MT946551.1 137275 137275 1 97 2 0.021 CCATACGATGA

MT946551.1 145926 145926 1 96 2 0.021 TTTCTCGGTAG

MT946551.1 147221 147221 1 97 2 0.021 AATTACGTAAT

MT946551.1 155706 155706 1 48 1 0.021 TGCCACGCTAC

MT946551.1 157565 157565 1 48 1 0.021 ATATACGCTAT

MT946551.1 157960 157960 1 48 1 0.021 TTGTTCGGTTT

MT946551.1 2660 2660 1 50 1 0.02 GTTATCGATGA

MT946551.1 14404 14409 2 100 2 0.02 GGTCCCGTTCCGTTGA

MT946551.1 16346 16346 1 98 2 0.02 GTGTGCGAAAG

MT946551.1 19557 19557 1 100 2 0.02 TAGCTCGGTAT

MT946551.1 22266 22266 1 98 2 0.02 AATTGCGTTTA

MT946551.1 36381 36381 1 101 2 0.02 AATATCGTTCT

MT946551.1 36716 36719 2 196 4 0.02 GAATTCGACGATGC

MT946551.1 36782 36782 1 101 2 0.02 TATACCGCCTC

MT946551.1 36834 36834 1 101 2 0.02 TCAGTCGTTCT

MT946551.1 37307 37315 2 200 4 0.02 TCTATCGATCTATCGAAAA

MT946551.1 38132 38132 1 100 2 0.02 TTGTACGAATC

MT946551.1 38678 38678 1 99 2 0.02 AAGTACGATCT

MT946551.1 40741 40741 1 99 2 0.02 ATGTGCGAGAA

MT946551.1 41268 41268 1 101 2 0.02 ATATACGAAGA

MT946551.1 49094 49096 2 100 2 0.02 GGTAACGCGTCTT

MT946551.1 58733 58733 1 100 2 0.02 TTAATCGTACT

MT946551.1 60272 60287 3 300 6 0.02 GTTATCGCTGATCGTCTAGACGATAT

MT946551.1 61222 61222 1 98 2 0.02 CCCACCGCCTT

MT946551.1 63138 63138 1 100 2 0.02 AAAGTCGAATA

MT946551.1 69230 69230 1 98 2 0.02 TCTTTCGATTT

MT946551.1 70545 70545 1 101 2 0.02 CTATGCGATTG

MT946551.1 70584 70584 1 99 2 0.02 TTAGGCGAGGT

MT946551.1 70744 70744 1 102 2 0.02 GTCACCGCATT

MT946551.1 71567 71567 1 100 2 0.02 ATAAACGCATA

MT946551.1 73862 73862 1 99 2 0.02 AAACTCGTACT

MT946551.1 79003 79003 1 100 2 0.02 TAGAGCGATTT

MT946551.1 79093 79093 1 100 2 0.02 TTCTACGTCTT

MT946551.1 91155 91155 1 99 2 0.02 TGTATCGATGA

MT946551.1 95152 95152 1 98 2 0.02 TAGCTCGAGAA

MT946551.1 101852 101852 1 102 2 0.02 ATCATCGCCTA

MT946551.1 103818 103818 1 49 1 0.02 GTATCCGTCAA

MT946551.1 106159 106161 2 200 4 0.02 TGGGGCGCGCAGT

MT946551.1 108768 108772 2 196 4 0.02 AACAACGACCGATAA

MT946551.1 110178 110178 1 99 2 0.02 GAACACGTCAT

MT946551.1 117120 117120 1 99 2 0.02 ATGGGCGTGGA

MT946551.1 124177 124185 2 200 4 0.02 TAATCCGTTTAGACGAGCA

MT946551.1 125787 125787 1 98 2 0.02 GATCTCGAGAA

MT946551.1 126513 126513 1 101 2 0.02 GGATACGCTCC

MT946551.1 131741 131741 1 99 2 0.02 TGACACGTTTA

MT946551.1 133343 133343 1 98 2 0.02 TAACTCGTAAA

MT946551.1 135711 135711 1 98 2 0.02 TAATACGTTAA

MT946551.1 136153 136153 1 98 2 0.02 AAGAACGTTCA

MT946551.1 142397 142397 1 100 2 0.02 TGAGACGATTA

MT946551.1 143649 143655 2 202 4 0.02 TCTATCGGTGGCGTTAT

MT946551.1 152560 152560 1 49 1 0.02 TAATCCGAATT

MT946551.1 152915 152915 1 51 1 0.02 AAACACGCTAC

MT946551.1 154150 154150 1 51 1 0.02 CTTTACGCAAA

MT946551.1 158499 158499 1 49 1 0.02 AATTTCGAGAT

MT946551.1 2082 2082 1 54 1 0.019 TAAGGCGTATC

MT946551.1 2322 2322 1 52 1 0.019 GTTATCGATCA

MT946551.1 3282 3282 1 52 1 0.019 TTTTACGTATC

MT946551.1 3463 3463 1 53 1 0.019 TGACACGTTTC

MT946551.1 5232 5232 1 54 1 0.019 ATACTCGACAA

MT946551.1 12004 12004 1 54 1 0.019 AGATCCGCTGC

MT946551.1 15250 15250 1 54 1 0.019 AATTCCGCATT

MT946551.1 23908 23908 1 105 2 0.019 TGATACGCAGT

MT946551.1 31113 31113 1 107 2 0.019 GATTGCGAAAT

MT946551.1 31163 31163 1 104 2 0.019 CTACACGCATG

MT946551.1 33133 33133 1 106 2 0.019 TGGTACGATGT

MT946551.1 36755 36755 1 106 2 0.019 TTCTTCGATTG

MT946551.1 38587 38587 1 104 2 0.019 TAAATCGATAT

MT946551.1 38609 38609 1 104 2 0.019 GTATGCGAGAT

MT946551.1 40999 40999 1 54 1 0.019 GGCAACGGATT

MT946551.1 43709 43709 1 105 2 0.019 GAAGACGATAG

MT946551.1 45463 45463 1 103 2 0.019 TTACACGCATC

MT946551.1 48307 48307 1 52 1 0.019 TTATCCGCTAA

MT946551.1 52626 52626 1 53 1 0.019 ATCTCCGGACA

MT946551.1 69745 69745 1 103 2 0.019 AAATGCGCCTC

MT946551.1 70336 70336 1 108 2 0.019 TAGTGCGCTAT

MT946551.1 71263 71263 1 103 2 0.019 AAGCTCGGTTT

MT946551.1 72727 72727 1 108 2 0.019 ACTTTCGAATA

MT946551.1 82925 82925 1 53 1 0.019 TCTTCCGTTGA

MT946551.1 87785 87785 1 105 2 0.019 AAACTCGAATG

MT946551.1 92794 92794 1 107 2 0.019 TTACTCGCTTA

MT946551.1 94433 94433 1 105 2 0.019 GCCATCGAATG

MT946551.1 104161 104161 1 103 2 0.019 CTATACGAAAT

MT946551.1 104872 104872 1 106 2 0.019 TCAGTCGAGGA

MT946551.1 113838 113838 1 107 2 0.019 TATGACGCATT

MT946551.1 114175 114175 1 105 2 0.019 GATATCGCCAT

MT946551.1 117578 117578 1 103 2 0.019 TCATGCGTCTT

MT946551.1 123104 123104 1 107 2 0.019 GATCTCGATAT

MT946551.1 123720 123720 1 108 2 0.019 AGATACGTACT

MT946551.1 125029 125029 1 108 2 0.019 GCAGTCGTACA

MT946551.1 129309 129309 1 105 2 0.019 TTCATCGATTT

MT946551.1 129688 129688 1 103 2 0.019 GGATACGATTC

MT946551.1 131712 131712 1 108 2 0.019 ACTGTCGAATT

MT946551.1 132597 132597 1 108 2 0.019 TCCAACGCATT

MT946551.1 135144 135144 1 105 2 0.019 CATACCGAAAG

MT946551.1 137158 137158 1 106 2 0.019 GACTACGTTGT

MT946551.1 137218 137221 2 208 4 0.019 AAAAGCGACGTCTT

MT946551.1 145259 145259 1 103 2 0.019 ATTCTCGCAGT

MT946551.1 157725 157725 1 54 1 0.019 TCAGGCGTCTC

MT946551.1 2791 2791 1 56 1 0.018 TAAATCGCTTG

MT946551.1 3142 3142 1 56 1 0.018 TAATTCGTATT

MT946551.1 3324 3324 1 55 1 0.018 TCCTTCGTTGT

MT946551.1 3368 3368 1 55 1 0.018 ACAGTCGAACA

MT946551.1 5664 5664 1 55 1 0.018 AATAACGCATC

MT946551.1 17962 17962 1 55 1 0.018 CTTAGCGTCAC

MT946551.1 27651 27651 1 55 1 0.018 ATGTCCGTGTA

MT946551.1 45528 45533 2 224 4 0.018 CCAGACGACTCGAGCC

MT946551.1 72793 72793 1 109 2 0.018 TTTTTCGTATT

MT946551.1 77821 77821 1 57 1 0.018 AAGTCCGTGGT

MT946551.1 83668 83676 2 112 2 0.018 ATGTCCGAAGAGACGATAG

MT946551.1 84850 84850 1 57 1 0.018 GAAGACGCTAG

MT946551.1 89704 89704 1 114 2 0.018 TTCTGCGCAAT

MT946551.1 105019 105019 1 55 1 0.018 AAACTCGGATT

MT946551.1 111212 111212 1 110 2 0.018 CATATCGTATA

MT946551.1 112073 112073 1 110 2 0.018 TAGAACGTCAG

MT946551.1 112318 112318 1 111 2 0.018 AGATACGTATT

MT946551.1 113159 113159 1 110 2 0.018 TTGTTCGAGGA

MT946551.1 113692 113692 1 110 2 0.018 TGCATCGATTG

MT946551.1 117002 117002 1 109 2 0.018 TCCTTCGTTTT

MT946551.1 117732 117732 1 109 2 0.018 AGTATCGAAAA

MT946551.1 123905 123905 1 111 2 0.018 AGCTTCGCTGG

MT946551.1 125977 125977 1 112 2 0.018 GATAACGAATG

MT946551.1 126945 126945 1 114 2 0.018 TTCTTCGATAG

MT946551.1 127518 127518 1 111 2 0.018 TACAGCGACAC

MT946551.1 127766 127768 2 110 2 0.018 TTAGACGCGGATA

MT946551.1 141213 141213 1 109 2 0.018 TACATCGTAAT

MT946551.1 143991 143991 1 55 1 0.018 GCTACCGTGAA

MT946551.1 147146 147146 1 56 1 0.018 ATTATCGGAAA

MT946551.1 150824 150824 1 57 1 0.018 AAGTACGTAAA

MT946551.1 151980 151980 1 56 1 0.018 ACAGACGACAA

MT946551.1 153512 153512 1 56 1 0.018 ATTAACGATCT

MT946551.1 156283 156283 1 56 1 0.018 TGTTACGTCTC

MT946551.1 156487 156494 2 112 2 0.018 GATTTCGACTTACGTCTG

MT946551.1 156546 156546 1 57 1 0.018 GAATACGAATT

MT946551.1 3085 3102 4 232 4 0.017 GGTCTCGGATTAGGCGTCGTTACGTATA

MT946551.1 3241 3241 1 59 1 0.017 GAATTCGAATA

MT946551.1 5701 5701 1 60 1 0.017 TATTACGTCCA

MT946551.1 11428 11428 1 59 1 0.017 GTATCCGTAAA

MT946551.1 16682 16687 3 180 3 0.017 CTATCCGCGACGAATT

MT946551.1 38724 38731 2 120 2 0.017 AGTTACGTGGATCGTCAC

MT946551.1 41689 41689 1 60 1 0.017 TAGTCCGTGTA

MT946551.1 42701 42701 1 60 1 0.017 TTCATCGGGAA

MT946551.1 45756 45763 2 234 4 0.017 AAGATCGAGGAACGCCAT

MT946551.1 46126 46126 1 115 2 0.017 TTGTACGTCTC

MT946551.1 55552 55557 2 116 2 0.017 GAGACCGTTCCGTTAC

MT946551.1 72010 72010 1 58 1 0.017 ATCTCCGAATC

MT946551.1 74040 74040 1 60 1 0.017 TATTTCGTGAG

MT946551.1 111531 111531 1 60 1 0.017 GTTAACGGAAA

MT946551.1 115347 115347 1 60 1 0.017 GATAACGGAGA

MT946551.1 120378 120378 1 60 1 0.017 TACCACGGATA

MT946551.1 148179 148179 1 60 1 0.017 GAACACGGATA

MT946551.1 151833 151836 2 120 2 0.017 TGAGTCGTCGTCTG

MT946551.1 153920 153923 2 116 2 0.017 ATAGACGGCGACAG

MT946551.1 156225 156225 1 59 1 0.017 TGAAACGTGTC

MT946551.1 3547 3547 1 62 1 0.016 CTGAACGATGG

MT946551.1 4834 4834 1 61 1 0.016 GTGATCGTCTA

MT946551.1 5677 5677 1 62 1 0.016 TTTTTCGTGTG

MT946551.1 6160 6160 1 61 1 0.016 GGTAACGTATG

MT946551.1 6507 6507 1 63 1 0.016 CTTCTCGTTTT

MT946551.1 6727 6727 1 62 1 0.016 GTCTACGGTGT

MT946551.1 8168 8168 1 63 1 0.016 AGTAACGAGTA

MT946551.1 11889 11889 1 62 1 0.016 ACTTACGTTAA

MT946551.1 11923 11926 2 126 2 0.016 TAATACGACGGATA

MT946551.1 17650 17650 1 63 1 0.016 ACTATCGGACT

MT946551.1 38809 38809 1 61 1 0.016 TATCCCGTTAG

MT946551.1 52928 52928 1 63 1 0.016 TTTTCCGTTAG

MT946551.1 54658 54661 2 124 2 0.016 ATAGCCGTCGCCAA

MT946551.1 61320 61320 1 62 1 0.016 ATATCCGTCAT

MT946551.1 96619 96619 1 64 1 0.016 GAATCCGATGA

MT946551.1 106662 106665 2 126 2 0.016 ATAGACGACGGGTT

MT946551.1 113951 113951 1 62 1 0.016 AATTCCGCTAC

MT946551.1 114744 114746 2 124 2 0.016 ACTTACGCGGTAA

MT946551.1 120005 120007 2 128 2 0.016 ATTATCGCGTTCA

MT946551.1 121716 121716 1 61 1 0.016 CCTCCCGTTAA

MT946551.1 138860 138860 1 61 1 0.016 GCTAACGGATC

MT946551.1 155522 155522 1 64 1 0.016 GAAACCGAAAG

MT946551.1 2922 2922 1 65 1 0.015 GTTATCGATCA

MT946551.1 4867 4867 1 68 1 0.015 ACATTCGATGG

MT946551.1 5847 5847 1 68 1 0.015 CAGCACGTCTA

MT946551.1 6014 6014 1 66 1 0.015 CCTTTCGAAAT

MT946551.1 7077 7077 1 68 1 0.015 GAGTTCGTTCT

MT946551.1 11375 11375 1 65 1 0.015 GTTGACGAAGA

MT946551.1 12562 12562 1 66 1 0.015 TCATCCGTTTC

MT946551.1 13401 13401 1 67 1 0.015 CATGGCGACTA

MT946551.1 14486 14486 1 65 1 0.015 ATAGTCGTGAT

MT946551.1 15193 15193 1 65 1 0.015 TCCCTCGGATA

MT946551.1 36454 36454 1 65 1 0.015 ATCTGCGGATT

MT946551.1 46351 46351 1 68 1 0.015 ATAATCGGAGA

MT946551.1 47798 47798 1 67 1 0.015 CACCACGTGTG

MT946551.1 49042 49048 2 136 2 0.015 AAATACGCTAACGGTAT

MT946551.1 55504 55504 1 65 1 0.015 TTTTCCGCATT

MT946551.1 56051 56051 1 65 1 0.015 GGATCCGGTTA

MT946551.1 67181 67181 1 67 1 0.015 GCCCCCGTTTA

MT946551.1 74273 74273 1 68 1 0.015 GAAAGCGAGAA

MT946551.1 81261 81261 1 65 1 0.015 CTCTTCGGGTT

MT946551.1 82061 82061 1 67 1 0.015 TTTGACGATAC

MT946551.1 93293 93293 1 66 1 0.015 AGTTCCGAGAT

MT946551.1 97794 97794 1 67 1 0.015 GGAATCGGATA

MT946551.1 107005 107014 2 134 2 0.015 TTAGACGTAAGTTCCGCTTG

MT946551.1 107032 107032 1 67 1 0.015 TTTAACGCTAA

MT946551.1 111642 111642 1 68 1 0.015 AAATCCGGAAC

MT946551.1 121287 121289 2 130 2 0.015 TTTTCCGCGTCTG

MT946551.1 122060 122062 2 130 2 0.015 ATGTCCGCGTTTC

MT946551.1 126618 126618 1 67 1 0.015 TTTGACGGACC

MT946551.1 138964 138964 1 68 1 0.015 TATCACGTGCA

MT946551.1 147318 147324 2 132 2 0.015 GGACACGGGAACGTGGT

MT946551.1 147886 147886 1 66 1 0.015 ATACACGTTAG

MT946551.1 150390 150400 2 134 2 0.015 CACAACGGATGCAGACGTCTT

MT946551.1 151439 151439 1 68 1 0.015 TTTGTCGCCCT

MT946551.1 151636 151636 1 68 1 0.015 CTCATCGAGAT

MT946551.1 152099 152103 2 134 2 0.015 AGTAGCGGACGTATG

MT946551.1 156320 156320 1 66 1 0.015 TTGTTCGACTG

MT946551.1 156447 156447 1 65 1 0.015 CTATTCGAATT

MT946551.1 157070 157070 1 67 1 0.015 TCCATCGTACA

MT946551.1 6067 6067 1 70 1 0.014 ATATTCGAGTA

MT946551.1 6562 6562 1 69 1 0.014 ATCCTCGCTTT

MT946551.1 7206 7206 1 70 1 0.014 AAATACGGTTA

MT946551.1 7906 7906 1 72 1 0.014 ACCATCGTAAT

MT946551.1 10089 10089 1 70 1 0.014 GCAGCCGTAAT

MT946551.1 11808 11808 1 71 1 0.014 TAAAGCGGTTA

MT946551.1 13500 13500 1 74 1 0.014 TATTACGTGGA

MT946551.1 14571 14571 1 74 1 0.014 AACATCGACAA

MT946551.1 22399 22399 1 72 1 0.014 ACCATCGGATG

MT946551.1 36509 36509 1 72 1 0.014 GGAGACGATCT

MT946551.1 38102 38104 2 138 2 0.014 CTGAACGCGTTCT

MT946551.1 39780 39780 1 73 1 0.014 AACTCCGAGGC

MT946551.1 42548 42548 1 74 1 0.014 GCTAGCGATCC

MT946551.1 47475 47475 1 70 1 0.014 TCATCCGATAA

MT946551.1 52589 52589 1 72 1 0.014 TTTACCGTTTG

MT946551.1 64837 64837 1 70 1 0.014 ATAACCGTTAG

MT946551.1 68684 68684 1 73 1 0.014 GGTGCCGTCAA

MT946551.1 77176 77176 1 69 1 0.014 TATGCCGTAAC

MT946551.1 80591 80599 2 148 2 0.014 GAGACCGCCAATTCGAGAA

MT946551.1 81610 81610 1 74 1 0.014 ACTATCGTTGG

MT946551.1 82030 82030 1 73 1 0.014 ATATACGTGTA

MT946551.1 84081 84081 1 71 1 0.014 GGTAACGTTAA

MT946551.1 84346 84346 1 71 1 0.014 GAAAACGATGA

MT946551.1 85808 85811 2 148 2 0.014 TTAGACGACGACAA

MT946551.1 93463 93463 1 70 1 0.014 TATACCGTTGT

MT946551.1 94199 94206 2 146 2 0.014 TGATACGTCAATCGGAGT

MT946551.1 95654 95654 1 72 1 0.014 AACATCGCTAT

MT946551.1 99519 99519 1 69 1 0.014 TTATACGTTAT

MT946551.1 100070 100070 1 74 1 0.014 ATCAACGTAAC

MT946551.1 102374 102374 1 73 1 0.014 TCCTTCGGGAG

MT946551.1 104256 104256 1 69 1 0.014 AGTGACGGATA

MT946551.1 104962 104971 2 140 2 0.014 ATTATCGTCACTAACGTGAT

MT946551.1 106761 106769 2 142 2 0.014 CTACCCGATGAATCGGACA

MT946551.1 107517 107517 1 71 1 0.014 TGCCACGCTAT

MT946551.1 114902 114902 1 70 1 0.014 TTATCCGGATC

MT946551.1 116718 116718 1 74 1 0.014 AAGTTCGGAGA

MT946551.1 123988 123988 1 71 1 0.014 AAGCACGCTGC

MT946551.1 127225 127225 1 73 1 0.014 AACATCGGGAA

MT946551.1 127789 127789 1 72 1 0.014 GAATCCGAAAT

MT946551.1 140491 140491 1 73 1 0.014 TATACCGAGTA

MT946551.1 150934 150934 1 70 1 0.014 GTCATCGTTAA

MT946551.1 152593 152593 1 70 1 0.014 ATTAACGCCTA

MT946551.1 152852 152852 1 69 1 0.014 ACACACGTAAT

MT946551.1 152871 152879 2 138 2 0.014 TTATACGTATCAACGGAGA

MT946551.1 153700 153700 1 70 1 0.014 GACATCGAAAA

MT946551.1 154496 154496 1 72 1 0.014 GGATTCGATTT

MT946551.1 156766 156766 1 69 1 0.014 TTGATCGATAA

MT946551.1 7891 7891 1 77 1 0.013 TAAATCGTATA

MT946551.1 8447 8450 2 156 2 0.013 TACATCGCCGTCAT

MT946551.1 8513 8513 1 80 1 0.013 TTGATCGTTGT

MT946551.1 11244 11244 1 77 1 0.013 CTCATCGCAGT

MT946551.1 13576 13592 3 231 3 0.013 AAGTTCGATGATGTCGCTATACGGTAC

MT946551.1 16300 16300 1 75 1 0.013 ACCCTCGTAAC

MT946551.1 17041 17041 1 79 1 0.013 CCAATCGGCCT

MT946551.1 17729 17729 1 77 1 0.013 ACAAACGTGAG

MT946551.1 20088 20091 2 156 2 0.013 AAAATCGTCGTTTA

MT946551.1 22099 22099 1 77 1 0.013 ATTCTCGGATA

MT946551.1 23599 23601 2 158 2 0.013 CACAACGCGTCTA

MT946551.1 27303 27303 1 76 1 0.013 TTTCCCGTAAT

MT946551.1 29005 29012 2 154 2 0.013 GTATTCGGTTATCGGATC

MT946551.1 35110 35110 1 77 1 0.013 ATCTCCGCACA

MT946551.1 35523 35523 1 76 1 0.013 CATTTCGTTGG

MT946551.1 36356 36356 1 80 1 0.013 TTAGCCGGACA

MT946551.1 37447 37453 2 152 2 0.013 AACAACGACACCGACTA

MT946551.1 39150 39150 1 78 1 0.013 TGCACCGAACA

MT946551.1 39555 39555 1 75 1 0.013 CTACACGGCAT

MT946551.1 41783 41792 2 152 2 0.013 TTCCTCGCAAATTCCGGATA

MT946551.1 47426 47426 1 79 1 0.013 TTTGACGCTAG

MT946551.1 48612 48612 1 78 1 0.013 GCCTCCGGAGT

MT946551.1 49599 49599 1 78 1 0.013 AAGCACGTGGA

MT946551.1 49808 49818 2 154 2 0.013 AATTCCGTAGGTAAACGAACA

MT946551.1 49899 49899 1 76 1 0.013 CACAACGAGAG

MT946551.1 51202 51202 1 76 1 0.013 GAGGACGGGAG

MT946551.1 53419 53419 1 78 1 0.013 AATAGCGTTTT

MT946551.1 56004 56004 1 77 1 0.013 CTCAACGTAAC

MT946551.1 58300 58300 1 78 1 0.013 AACTACGAGGA

MT946551.1 59278 59278 1 75 1 0.013 ACTAGCGTGAA

MT946551.1 59590 59590 1 75 1 0.013 GAAATCGGAAA

MT946551.1 64705 64705 1 78 1 0.013 TACAACGTAAA

MT946551.1 65186 65186 1 76 1 0.013 TTTTCCGCAAT

MT946551.1 67070 67073 2 150 2 0.013 GATTTCGACGGAGA

MT946551.1 70598 70600 2 150 2 0.013 GGAGACGCGTGGA

MT946551.1 78733 78739 2 154 2 0.013 TTTAACGAATACGGATT

MT946551.1 81035 81035 1 77 1 0.013 GAATGCGGTGC

MT946551.1 81909 81909 1 78 1 0.013 ACATACGAGAC

MT946551.1 84502 84505 2 160 2 0.013 TCTTACGCCGATAT

MT946551.1 84541 84553 3 231 3 0.013 TTAGCCGCCGTATATTCCGATTT

MT946551.1 85064 85064 1 79 1 0.013 AATGTCGAGCT

MT946551.1 85736 85744 3 228 3 0.013 ACTTGCGTGTCGTCGTTCA

MT946551.1 86200 86200 1 78 1 0.013 ATAATCGTCTT

MT946551.1 90302 90308 2 156 2 0.013 CATCTCGTTCACGTTTG

MT946551.1 92741 92741 1 79 1 0.013 TTTAGCGGGCT

MT946551.1 92875 92875 1 79 1 0.013 ATACTCGTGTC

MT946551.1 94077 94077 1 78 1 0.013 AGAATCGTATA

MT946551.1 94830 94837 2 158 2 0.013 TCCTCCGTAAGACGATTA

MT946551.1 96283 96283 1 77 1 0.013 GGAGCCGGTGT

MT946551.1 96454 96460 2 154 2 0.013 TTTTTCGTATCCGGATC

MT946551.1 97635 97635 1 76 1 0.013 AGAGTCGAATA

MT946551.1 101405 101408 2 156 2 0.013 AACAACGACGAACC

MT946551.1 101955 101955 1 78 1 0.013 CCAATCGTTTG

MT946551.1 104139 104147 2 152 2 0.013 AGATGCGGAAGAACGGTAG

MT946551.1 106119 106119 1 77 1 0.013 AATATCGCTAT

MT946551.1 107200 107202 2 160 2 0.013 TAATTCGCGCACC

MT946551.1 111970 111970 1 75 1 0.013 AACTCCGTTAG

MT946551.1 113447 113447 1 76 1 0.013 AATTACGTGAC

MT946551.1 114248 114248 1 75 1 0.013 TATAACGGTCA

MT946551.1 118094 118094 1 78 1 0.013 TTGCTCGTTAT

MT946551.1 121848 121848 1 79 1 0.013 AAGGTCGTTAG

MT946551.1 125392 125398 2 160 2 0.013 CTTTGCGTTGACGGAAA

MT946551.1 132979 132981 2 152 2 0.013 TTGCACGCGACTT

MT946551.1 134231 134231 1 78 1 0.013 AATAGCGGATA

MT946551.1 135945 135945 1 78 1 0.013 TAAAACGTGGA

MT946551.1 136592 136592 1 79 1 0.013 ACCAGCGTCTA

MT946551.1 138911 138917 2 158 2 0.013 CCATCCGTTATCGAAGA

MT946551.1 142329 142332 2 160 2 0.013 ATTTACGACGCTGT

MT946551.1 145550 145561 3 231 3 0.013 GAATACGACGACCATCCGTATG

MT946551.1 147492 147492 1 79 1 0.013 ACTCTCGAGGA

MT946551.1 150430 150430 1 79 1 0.013 TATTACGAAGA

MT946551.1 150694 150698 2 160 2 0.013 ATTGACGAACGTATT

MT946551.1 152421 152421 1 76 1 0.013 CCAAGCGAATT

MT946551.1 153401 153401 1 77 1 0.013 ATGTACGTGTC

MT946551.1 153529 153529 1 75 1 0.013 TATATCGTATC

MT946551.1 155376 155376 1 79 1 0.013 TACTACGTACT

MT946551.1 9437 9437 1 86 1 0.012 ACATCCGCCTT

MT946551.1 10757 10757 1 82 1 0.012 TATACCGGCAT

MT946551.1 11772 11782 2 172 2 0.012 ATAAACGAATTTACTCGCATT

MT946551.1 15402 15402 1 85 1 0.012 CATTACGTATA

MT946551.1 18217 18217 1 84 1 0.012 TCATTCGTAAA

MT946551.1 19452 19452 1 84 1 0.012 GTTGTCGCAGC

MT946551.1 22354 22354 1 84 1 0.012 TAAACCGCAAG

MT946551.1 23083 23083 1 82 1 0.012 TATTTCGTCCA

MT946551.1 24662 24669 2 168 2 0.012 TTGACCGATTATCGAGTT

MT946551.1 25005 25005 1 82 1 0.012 TATTTCGAATT

MT946551.1 25512 25512 1 81 1 0.012 CATCTCGGTAC

MT946551.1 25539 25539 1 85 1 0.012 AGAAGCGCATT

MT946551.1 25641 25641 1 82 1 0.012 CCTGCCGTTAC

MT946551.1 27505 27505 1 81 1 0.012 TATGACGTCCC

MT946551.1 29941 29941 1 85 1 0.012 GTCCACGTTCT

MT946551.1 31365 31365 1 83 1 0.012 GATAGCGAAGG

MT946551.1 38027 38054 6 498 6 0.012 CCTTTCGCGTCCGTAGTATCGTCACCGATGAACGTCAT

MT946551.1 38871 38871 1 85 1 0.012 TGCTGCGTTTC

MT946551.1 40386 40386 1 85 1 0.012 CAGATCGTATA

MT946551.1 42582 42587 2 168 2 0.012 ATTGACGATTCGTCTC

MT946551.1 42928 42935 2 170 2 0.012 GAATTCGTTTTTCGACAT

MT946551.1 44779 44779 1 85 1 0.012 AGATGCGGATT

MT946551.1 45903 45903 1 84 1 0.012 GGTACCGGAGA

MT946551.1 47493 47502 2 168 2 0.012 ATTTCCGACACCAACGGTTT

MT946551.1 47993 47993 1 84 1 0.012 TCCATCGTTGC

MT946551.1 50984 50984 1 83 1 0.012 TTGTACGCTAA

MT946551.1 51011 51011 1 85 1 0.012 CTCCCCGAAGA

MT946551.1 51320 51320 1 84 1 0.012 TTATTCGGTGT

MT946551.1 51666 51666 1 86 1 0.012 AGAGACGTTTT

MT946551.1 55708 55708 1 83 1 0.012 TCTAACGAAAA

MT946551.1 56595 56595 1 81 1 0.012 CTTAACGTTGT

MT946551.1 57740 57743 2 164 2 0.012 AGACTCGACGGATG

MT946551.1 58050 58050 1 81 1 0.012 TCTTACGTGTT

MT946551.1 58800 58800 1 85 1 0.012 AGAATCGTGTT

MT946551.1 58813 58819 2 172 2 0.012 CCAGACGTTCACGACAC

MT946551.1 60778 60778 1 85 1 0.012 TCCATCGTTTG

MT946551.1 60923 60930 2 164 2 0.012 AGAGCCGGTTGCCGCAAA

MT946551.1 63118 63118 1 82 1 0.012 TCTAACGTGAT

MT946551.1 63518 63525 2 162 2 0.012 TTCAACGTAAACCGTTTA

MT946551.1 63800 63800 1 86 1 0.012 TAAACCGTTTA

MT946551.1 63937 63942 2 164 2 0.012 TACAGCGACACGGTAT

MT946551.1 64114 64119 2 168 2 0.012 TGACTCGGTTCGTTGA

MT946551.1 64281 64286 2 168 2 0.012 AACCCCGTGGCGTCTA

MT946551.1 65413 65413 1 84 1 0.012 GATAGCGGACA

MT946551.1 65492 65492 1 83 1 0.012 GGAAACGTTTC

MT946551.1 65588 65588 1 81 1 0.012 TGGCTCGTACC

MT946551.1 66967 66967 1 83 1 0.012 ACAGCCGTCTC

MT946551.1 68420 68420 1 85 1 0.012 CATAACGTCAT

MT946551.1 69148 69158 2 172 2 0.012 TTATACGGGATATGACGACTT

MT946551.1 70837 70837 1 82 1 0.012 TTTAACGTTAA

MT946551.1 71008 71013 2 170 2 0.012 GTTTTCGAAACGGTAT

MT946551.1 71371 71377 2 172 2 0.012 AATAACGTTATCGTTTT

MT946551.1 72934 72934 1 86 1 0.012 ATGAACGAAAT

MT946551.1 74798 74801 2 170 2 0.012 AGTATCGACGGTTC

MT946551.1 75086 75086 1 85 1 0.012 ACAAACGTGGG

MT946551.1 75293 75293 1 83 1 0.012 GTTGGCGGTTT

MT946551.1 76737 76745 2 168 2 0.012 AGTGACGGAAAATCGTCTT

MT946551.1 79686 79686 1 83 1 0.012 ATAAACGATTG

MT946551.1 81512 81512 1 86 1 0.012 GATCTCGATGA

MT946551.1 82166 82166 1 81 1 0.012 TTGTACGAAAA

MT946551.1 82389 82389 1 83 1 0.012 TGTAGCGAACT

MT946551.1 85707 85707 1 86 1 0.012 CTGCTCGTATA

MT946551.1 86911 86911 1 81 1 0.012 CAACACGGGAT

MT946551.1 89427 89427 1 86 1 0.012 TCCTTCGTTAT

MT946551.1 91786 91791 2 170 2 0.012 GTTACCGTTACGGTAA

MT946551.1 94692 94692 1 83 1 0.012 TCTACCGCATC

MT946551.1 95895 95895 1 85 1 0.012 CATATCGTCTA

MT946551.1 96357 96357 1 82 1 0.012 GCTGGCGGCCA

MT946551.1 96831 96831 1 84 1 0.012 AATAGCGGGTA

MT946551.1 99692 99692 1 83 1 0.012 ATTACCGGTCA

MT946551.1 101247 101247 1 84 1 0.012 GGATACGCTAC

MT946551.1 102212 102212 1 82 1 0.012 ACTCTCGATAA

MT946551.1 102339 102345 2 168 2 0.012 CTTATCGGTTCCGAGTC

MT946551.1 103484 103484 1 86 1 0.012 ATCTACGTTTG

MT946551.1 109242 109242 1 81 1 0.012 TTTAGCGTATT

MT946551.1 109842 109842 1 86 1 0.012 TAGGACGGTAT

MT946551.1 109958 109958 1 81 1 0.012 GTTATCGGAAA

MT946551.1 110858 110858 1 82 1 0.012 CTTACCGGTAT

MT946551.1 111728 111728 1 83 1 0.012 TATAACGTTAG

MT946551.1 115773 115773 1 83 1 0.012 TGCTTCGGCCA

MT946551.1 116608 116608 1 84 1 0.012 ACTTGCGTCTG

MT946551.1 118375 118375 1 85 1 0.012 AACTTCGTGAA

MT946551.1 118472 118472 1 85 1 0.012 ATGCTCGCAAC

MT946551.1 119394 119394 1 84 1 0.012 TTACACGCATC

MT946551.1 119646 119646 1 86 1 0.012 AAACACGATTT

MT946551.1 119921 119925 2 166 2 0.012 TAACTCGGTCGGTTT

MT946551.1 120598 120598 1 85 1 0.012 TCAATCGTTCC

MT946551.1 121386 121386 1 81 1 0.012 ATAGACGTGTT

MT946551.1 122167 122167 1 82 1 0.012 GGTTGCGTCTA

MT946551.1 124453 124453 1 81 1 0.012 TGAAACGGTAC

MT946551.1 125629 125629 1 81 1 0.012 GGTAACGTCAT

MT946551.1 126741 126741 1 82 1 0.012 ACTATCGGCTC

MT946551.1 129077 129089 3 249 3 0.012 ATCTCCGATCGTTATAGCGAGAA

MT946551.1 131264 131264 1 85 1 0.012 GACCACGATAA

MT946551.1 135021 135021 1 86 1 0.012 GTTTGCGGAAG

MT946551.1 144526 144532 2 168 2 0.012 TTGTACGGATACGCATC

MT946551.1 147758 147758 1 84 1 0.012 AAAAACGAAAG

MT946551.1 148086 148095 2 162 2 0.012 TCCAGCGTCAATCTCGTCAG

MT946551.1 148475 148475 1 84 1 0.012 TTTTACGTATC

MT946551.1 148796 148796 1 85 1 0.012 GTGTTCGGTTC

MT946551.1 148864 148864 1 84 1 0.012 CAAAACGTATA

MT946551.1 149012 149012 1 85 1 0.012 TTTTTCGTTGG

MT946551.1 149261 149261 1 86 1 0.012 CAATGCGCTGG

MT946551.1 150021 150021 1 81 1 0.012 TATCTCGTATC

MT946551.1 9245 9251 2 188 2 0.011 ATCATCGAATACGAATC

MT946551.1 9710 9710 1 90 1 0.011 TACACCGTACC

MT946551.1 10282 10282 1 91 1 0.011 CATTGCGTGTT

MT946551.1 10868 10868 1 88 1 0.011 ATGTTCGGTAT

MT946551.1 11348 11348 1 88 1 0.011 AATCACGCATA

MT946551.1 11848 11848 1 90 1 0.011 TGTTGCGGTGC

MT946551.1 14351 14359 2 180 2 0.011 CAAGTCGTCTAAACGTTGT

MT946551.1 14726 14726 1 88 1 0.011 TTAAACGTACA

MT946551.1 15534 15534 1 89 1 0.011 TACTACGTATA

MT946551.1 16945 16954 2 188 2 0.011 GGAAGCGAATGATCCGGAAA

MT946551.1 17066 17066 1 88 1 0.011 GGCATCGTTTC

MT946551.1 19647 19647 1 90 1 0.011 ATGTTCGAAAA

MT946551.1 20108 20108 1 92 1 0.011 AAAATCGTTTA

MT946551.1 22063 22063 1 89 1 0.011 TATATCGTTGG

MT946551.1 22691 22691 1 94 1 0.011 TCTAACGAGGA

MT946551.1 23377 23377 1 88 1 0.011 TTTAACGATTT

MT946551.1 23718 23718 1 93 1 0.011 AATGTCGGGCC

MT946551.1 24295 24295 1 95 1 0.011 ATAAGCGCTCC

MT946551.1 25473 25473 1 91 1 0.011 CATTACGTTTC

MT946551.1 27141 27144 2 176 2 0.011 TGAAACGTCGGATC

MT946551.1 27236 27236 1 89 1 0.011 ATTATCGTTTT

MT946551.1 27348 27348 1 89 1 0.011 GTCCTCGAATT

MT946551.1 30480 30480 1 95 1 0.011 CAATACGAAAA

MT946551.1 30640 30640 1 94 1 0.011 TATGGCGTCAG

MT946551.1 30732 30742 2 184 2 0.011 CACTACGTTGAAGATCGTACA

MT946551.1 31516 31516 1 94 1 0.011 TGATTCGAATT

MT946551.1 33681 33681 1 95 1 0.011 AGACACGCCAT

MT946551.1 33941 33948 2 176 2 0.011 TAGAACGTTTAACGAGGA

MT946551.1 34849 34849 1 91 1 0.011 ATTTTCGTAAC

MT946551.1 38337 38337 1 89 1 0.011 CTAGACGATTA

MT946551.1 39277 39285 2 186 2 0.011 AGAAGCGTGTAAACGAAAA

MT946551.1 39996 39996 1 95 1 0.011 CAGTACGCCAC

MT946551.1 40210 40218 2 176 2 0.011 ATAAGCGTCTTCTCGATAG

MT946551.1 40644 40644 1 88 1 0.011 TGGTGCGTCTG

MT946551.1 41713 41713 1 94 1 0.011 TAACACGAGCT

MT946551.1 42000 42000 1 91 1 0.011 AGTCACGCAAT

MT946551.1 43232 43232 1 91 1 0.011 GAAAACGAGTT

MT946551.1 43689 43694 2 174 2 0.011 GTTTCCGTTCCGGATC

MT946551.1 43829 43829 1 89 1 0.011 GGATCCGTTTA

MT946551.1 47061 47061 1 92 1 0.011 GTAACCGACTT

MT946551.1 47670 47670 1 93 1 0.011 TTGAACGTATT

MT946551.1 49655 49657 2 176 2 0.011 CTAACCGCGAAGC

MT946551.1 50816 50816 1 89 1 0.011 GTCCCCGCTCC

MT946551.1 51725 51728 2 180 2 0.011 AGTGACGTCGTATC

MT946551.1 55458 55458 1 88 1 0.011 CTACTCGTATG

MT946551.1 55732 55732 1 88 1 0.011 GTGTTCGGCAT

MT946551.1 56403 56403 1 90 1 0.011 AATGCCGGTAT

MT946551.1 57444 57444 1 93 1 0.011 TGACTCGCAAC

MT946551.1 57672 57682 2 184 2 0.011 AAAATCGATAAACTGCGCCAA

MT946551.1 58382 58382 1 88 1 0.011 GTTTTCGGATA

MT946551.1 58561 58561 1 88 1 0.011 GCTCCCGCTAA

MT946551.1 58674 58674 1 93 1 0.011 TCTTTCGGCAT

MT946551.1 61174 61174 1 90 1 0.011 AGCATCGATCC

MT946551.1 61449 61449 1 87 1 0.011 TATATCGTTGT

MT946551.1 62724 62734 2 190 2 0.011 CTTAACGATGTTCTTCGCAGA

MT946551.1 63010 63010 1 93 1 0.011 TTGTTCGACTT

MT946551.1 63697 63700 2 174 2 0.011 TGTTACGTCGGCTC

MT946551.1 63864 63864 1 94 1 0.011 GAGGTCGCAAA

MT946551.1 63878 63878 1 92 1 0.011 ACTGCCGTATC

MT946551.1 64419 64419 1 88 1 0.011 TATACCGGTGA

MT946551.1 64441 64441 1 90 1 0.011 TGACTCGAGTT

MT946551.1 64758 64762 2 174 2 0.011 AGATCCGTACGCAGT

MT946551.1 65429 65429 1 92 1 0.011 ATTCTCGGTAG

MT946551.1 67292 67292 1 89 1 0.011 GAGTTCGATCC

MT946551.1 68850 68850 1 95 1 0.011 CTCTTCGAAAA

MT946551.1 70387 70387 1 89 1 0.011 ATTAACGAGAT

MT946551.1 70398 70398 1 88 1 0.011 ATATGCGAATT

MT946551.1 70523 70526 2 184 2 0.011 GATTTCGGCGCCTA

MT946551.1 70727 70727 1 89 1 0.011 TATAGCGGTTA

MT946551.1 72593 72593 1 91 1 0.011 TCTACCGTTTT

MT946551.1 73515 73515 1 95 1 0.011 CATATCGCACA

MT946551.1 74056 74056 1 91 1 0.011 GTCATCGTAGG

MT946551.1 74613 74613 1 95 1 0.011 GTCAGCGACAT

MT946551.1 75098 75098 1 95 1 0.011 GGAGGCGTTAA

MT946551.1 77491 77494 2 182 2 0.011 GAAGTCGTCGATAT

MT946551.1 77749 77752 2 176 2 0.011 TATAACGGCGTTAA

MT946551.1 78712 78712 1 91 1 0.011 AAGAACGATAT

MT946551.1 78760 78769 2 180 2 0.011 GATAACGTTGATTTCGCTAC

MT946551.1 78785 78785 1 92 1 0.011 TAGAACGAAGT

MT946551.1 78850 78850 1 95 1 0.011 TTTTTCGAACT

MT946551.1 82250 82250 1 87 1 0.011 AAAGTCGACAA

MT946551.1 83293 83293 1 95 1 0.011 TTGTTCGCCTT

MT946551.1 84705 84705 1 92 1 0.011 TGAAACGTATT

MT946551.1 85464 85464 1 94 1 0.011 GGCAGCGAAGT

MT946551.1 85976 85985 2 190 2 0.011 TCAAACGGTGCAGACGTATT

MT946551.1 87240 87254 3 276 3 0.011 ATAAACGAGTATCCGTGTCCGCTAT

MT946551.1 87749 87749 1 94 1 0.011 CAATACGAAAT

MT946551.1 87815 87815 1 92 1 0.011 TATTACGAATC

MT946551.1 89033 89033 1 87 1 0.011 AAACTCGTTAT

MT946551.1 89333 89333 1 92 1 0.011 AATGACGCATA

MT946551.1 89997 89997 1 88 1 0.011 AGTGACGGACT

MT946551.1 90812 90812 1 93 1 0.011 CTACTCGAACA

MT946551.1 91648 91648 1 94 1 0.011 ATAAACGATGG

MT946551.1 93333 93333 1 95 1 0.011 ATCTCCGCAAT

MT946551.1 93489 93489 1 91 1 0.011 ATAATCGTAGA

MT946551.1 93661 93661 1 89 1 0.011 TTATCCGCACA

MT946551.1 95686 95686 1 91 1 0.011 ATATTCGTAGT

MT946551.1 95826 95835 2 186 2 0.011 CAGATCGTTTGTTTCGGCCT

MT946551.1 96569 96569 1 91 1 0.011 AATTGCGATTA

MT946551.1 96866 96866 1 94 1 0.011 TTCAACGAGGA

MT946551.1 96996 97003 2 180 2 0.011 GTTGTCGGTAAACGATTT

MT946551.1 97121 97121 1 95 1 0.011 AGTCTCGCATG

MT946551.1 97933 97933 1 89 1 0.011 TATACCGATTT

MT946551.1 99716 99716 1 87 1 0.011 TTCATCGTATC

MT946551.1 101028 101032 2 188 2 0.011 TAGTACGGGCGCTGT

MT946551.1 102294 102294 1 94 1 0.011 GTATACGCAGA

MT946551.1 102859 102859 1 88 1 0.011 ACTCTCGTATA

MT946551.1 103214 103229 3 264 3 0.011 TTGCACGTTTGGAAGCGCCACGGTTA

MT946551.1 103339 103339 1 93 1 0.011 GATATCGCATA

MT946551.1 103790 103790 1 91 1 0.011 ACCACCGATAA

MT946551.1 105707 105707 1 94 1 0.011 TGTTTCGATAT

MT946551.1 105932 105932 1 88 1 0.011 TACAGCGCATA

MT946551.1 106092 106092 1 91 1 0.011 ATGGACGATAA

MT946551.1 107931 107931 1 88 1 0.011 AGTAACGGCTG

MT946551.1 108607 108607 1 91 1 0.011 TATGCCGAACT

MT946551.1 110195 110201 2 182 2 0.011 ATCATCGTCATCGATAA

MT946551.1 110822 110822 1 89 1 0.011 TAAACCGTTTA

MT946551.1 111093 111093 1 89 1 0.011 GAGTACGGATT

MT946551.1 111327 111327 1 87 1 0.011 CCTAGCGTTAT

MT946551.1 113593 113598 2 188 2 0.011 ATCAACGACTCGGATA

MT946551.1 113967 113967 1 94 1 0.011 GGATACGGGAA

MT946551.1 115802 115802 1 90 1 0.011 AATGTCGTACA

MT946551.1 116100 116100 1 90 1 0.011 TATACCGATAT

MT946551.1 116433 116433 1 94 1 0.011 AGTAACGAAAC

MT946551.1 116663 116663 1 89 1 0.011 AAAGACGAAGC

MT946551.1 116747 116747 1 92 1 0.011 AATAGCGCATG

MT946551.1 117097 117104 2 182 2 0.011 AAATTCGAGATCCGAATT

MT946551.1 117808 117808 1 93 1 0.011 AAATACGTAAA

MT946551.1 119228 119228 1 92 1 0.011 ATATTCGAATG

MT946551.1 120111 120111 1 87 1 0.011 TCTACCGCAGT

MT946551.1 121269 121273 2 174 2 0.011 GGCATCGATCGTAGC

MT946551.1 121502 121502 1 94 1 0.011 TTGTGCGACAG

MT946551.1 123284 123284 1 89 1 0.011 TGTGCCGCATA

MT946551.1 124196 124196 1 92 1 0.011 CATAGCGATGA

MT946551.1 124783 124786 2 186 2 0.011 TTATTCGCCGTTGT

MT946551.1 128018 128018 1 93 1 0.011 TTTTACGTGGT

MT946551.1 131358 131363 2 188 2 0.011 TTCATCGTTACGGAGA

MT946551.1 131414 131414 1 88 1 0.011 TTTATCGGTAA

MT946551.1 133462 133462 1 92 1 0.011 AGATACGCATT

MT946551.1 133705 133705 1 87 1 0.011 GAATTCGAGGA

MT946551.1 134740 134746 2 182 2 0.011 GATATCGCATCCGTTTG

MT946551.1 137039 137039 1 93 1 0.011 TGACACGATTA

MT946551.1 138290 138293 2 178 2 0.011 AACATCGACGGTGT

MT946551.1 139705 139705 1 92 1 0.011 ACAACCGAAAT

MT946551.1 139986 139998 3 273 3 0.011 CTAAGCGGAATCGGGTACGAGAG

MT946551.1 140829 140839 2 176 2 0.011 ATTCACGGTGGGTTACGACTA

MT946551.1 143026 143026 1 93 1 0.011 TTTAACGCTCA

MT946551.1 144854 144860 3 282 3 0.011 TCATGCGACGTCGGATT

MT946551.1 144893 144893 1 87 1 0.011 ATAAACGACTA

MT946551.1 145748 145748 1 92 1 0.011 GTGTGCGTGAC

MT946551.1 146015 146015 1 95 1 0.011 AAAAACGAAAT

MT946551.1 146047 146047 1 94 1 0.011 GTTAACGCCCT

MT946551.1 146274 146282 2 186 2 0.011 ATATACGAAACACCGATAA

MT946551.1 146573 146573 1 89 1 0.011 TAGAACGAATA

MT946551.1 147305 147305 1 88 1 0.011 ACTATCGACAA

MT946551.1 10821 10821 1 96 1 0.01 AATAACGCAAT

MT946551.1 17783 17783 1 97 1 0.01 TCTATCGTGTG

MT946551.1 18560 18560 1 102 1 0.01 TGGAACGTATA

MT946551.1 22187 22187 1 100 1 0.01 TCTAACGCTCC

MT946551.1 23274 23274 1 105 1 0.01 AGTTACGCAAT

MT946551.1 24002 24002 1 99 1 0.01 TTCTACGAAGA

MT946551.1 24737 24737 1 104 1 0.01 ATAGGCGGTGT

MT946551.1 24873 24886 4 384 4 0.01 CACACCGACGATGGCGGCCGCCAC

MT946551.1 26877 26877 1 96 1 0.01 TCACTCGATAA

MT946551.1 28040 28040 1 96 1 0.01 CAGAGCGATAG

MT946551.1 31247 31247 1 97 1 0.01 GGACACGGTGT

MT946551.1 31531 31531 1 98 1 0.01 TTCTTCGATAT

MT946551.1 31660 31667 2 196 2 0.01 AGTAGCGGCTACCGCCAT

MT946551.1 32679 32679 1 102 1 0.01 CTGTTCGTAGG

MT946551.1 33075 33075 1 96 1 0.01 CAACACGTTAT

MT946551.1 34946 34946 1 99 1 0.01 AGAAACGCAAC

MT946551.1 35641 35641 1 104 1 0.01 AGATACGTATC

MT946551.1 37184 37184 1 102 1 0.01 TTGTACGTATA

MT946551.1 37909 37909 1 99 1 0.01 TAGTACGACTT

MT946551.1 38776 38783 2 192 2 0.01 TTGCTCGTCCAACGAGTA

MT946551.1 43466 43469 2 206 2 0.01 TATAGCGGCGTACA

MT946551.1 43561 43561 1 98 1 0.01 GATTACGAGCC

MT946551.1 45036 45036 1 102 1 0.01 TGTATCGTATG

MT946551.1 45732 45732 1 103 1 0.01 TTCCTCGTTTC

MT946551.1 46746 46755 2 204 2 0.01 TTTGACGTATGTAGCGTACA

MT946551.1 46941 46941 1 96 1 0.01 TAGAACGGTTA

MT946551.1 47373 47373 1 96 1 0.01 TCAAACGAATA

MT946551.1 49000 49000 1 101 1 0.01 TCTATCGCCTT

MT946551.1 49741 49745 2 194 2 0.01 CAATTCGTTCGATGA

MT946551.1 52254 52254 1 97 1 0.01 AGTATCGACTT

MT946551.1 52366 52366 1 98 1 0.01 TAATACGTTGA

MT946551.1 53235 53251 3 288 3 0.01 ATGTTCGTATGTCGTTATCATCGTATA

MT946551.1 57220 57220 1 97 1 0.01 TCTGCCGCATA

MT946551.1 59199 59199 1 103 1 0.01 GTGTTCGAATG

MT946551.1 59734 59734 1 103 1 0.01 GCATACGTACC

MT946551.1 59887 59887 1 98 1 0.01 TGTTACGGAGC

MT946551.1 61123 61123 1 96 1 0.01 ACTTGCGGGGT

MT946551.1 61742 61742 1 103 1 0.01 TAGAACGATGT

MT946551.1 63461 63461 1 98 1 0.01 GTGAGCGTATG

MT946551.1 65083 65083 1 101 1 0.01 ACAGGCGATGG

MT946551.1 70202 70202 1 103 1 0.01 TATAACGTACA

MT946551.1 70502 70502 1 99 1 0.01 ATATTCGAATT

MT946551.1 70956 70956 1 99 1 0.01 GATAACGTTTA

MT946551.1 71341 71354 3 297 3 0.01 CAAGACGGGACGAAGAAACGTAAT

MT946551.1 72238 72242 2 204 2 0.01 TGGTTCGACCGATGG

MT946551.1 72559 72568 2 196 2 0.01 AACCACGGAGATGGCGTATT

MT946551.1 72659 72659 1 102 1 0.01 ACTAACGCAAC

MT946551.1 72921 72921 1 103 1 0.01 TAAAGCGCTGA

MT946551.1 74500 74510 2 196 2 0.01 CTCCTCGTAAACCAGCGGCTA

MT946551.1 74911 74911 1 105 1 0.01 TTGTTCGTAAA

MT946551.1 75219 75229 2 210 2 0.01 TATAACGTTATGAAACGAATT

MT946551.1 75556 75556 1 102 1 0.01 GTGAACGAAAG

MT946551.1 76021 76021 1 101 1 0.01 TTAAACGCCAT

MT946551.1 76080 76086 2 206 2 0.01 GATTTCGAATACGATAA

MT946551.1 76237 76246 2 202 2 0.01 CTCCCCGTCTTAAACGATTT

MT946551.1 80629 80629 1 96 1 0.01 ATCAACGTTTT

MT946551.1 85612 85612 1 99 1 0.01 CCCTTCGATAT

MT946551.1 86954 86954 1 98 1 0.01 CATGGCGTGTT

MT946551.1 87573 87573 1 102 1 0.01 ACACTCGGTTT

MT946551.1 88047 88055 2 208 2 0.01 TTAGACGATAGATCGATGG

MT946551.1 88873 88873 1 103 1 0.01 CCATACGTCTC

MT946551.1 89820 89820 1 102 1 0.01 GTTTTCGATAC

MT946551.1 89896 89896 1 105 1 0.01 CTATTCGAAAA

MT946551.1 90423 90423 1 101 1 0.01 TAGATCGCTTT

MT946551.1 91420 91420 1 103 1 0.01 CCAGTCGGTGG

MT946551.1 93208 93208 1 99 1 0.01 TCTCTCGTTAG

MT946551.1 95479 95479 1 105 1 0.01 TGGTACGTTGA

MT946551.1 95786 95788 2 192 2 0.01 CCCTACGCGTATA

MT946551.1 96901 96906 2 208 2 0.01 TCTGACGAAACGATGA

MT946551.1 96973 96982 2 204 2 0.01 GTCATCGAAAAGAACGGAGA

MT946551.1 98160 98169 2 194 2 0.01 GGTGACGAACTCATCGTATA

MT946551.1 100043 100043 1 98 1 0.01 ATATACGAAGG

MT946551.1 100245 100245 1 96 1 0.01 ATGGGCGACTT

MT946551.1 101714 101714 1 96 1 0.01 TGCATCGTCTA

MT946551.1 104269 104269 1 104 1 0.01 CAAAACGATTT

MT946551.1 104389 104392 2 206 2 0.01 AATGCCGCCGTAGT

MT946551.1 105590 105593 2 194 2 0.01 ACATGCGCCGGCAG

MT946551.1 106143 106143 1 99 1 0.01 GACTCCGCTAA

MT946551.1 106290 106300 2 200 2 0.01 AAAGGCGAAGATACACGAAAG

MT946551.1 107529 107529 1 104 1 0.01 ACTATCGCCCA

MT946551.1 109200 109200 1 102 1 0.01 ACATACGTATA

MT946551.1 109649 109649 1 98 1 0.01 ATAGACGTACT

MT946551.1 109699 109699 1 104 1 0.01 ATATTCGTATC

MT946551.1 115055 115055 1 101 1 0.01 ACTAACGTCTG

MT946551.1 115294 115294 1 98 1 0.01 AAATTCGCATT

MT946551.1 116681 116681 1 103 1 0.01 ACAGGCGAATG

MT946551.1 118597 118600 2 192 2 0.01 ATGGACGCCGTCCA

MT946551.1 118644 118644 1 104 1 0.01 ATAGCCGCTCT

MT946551.1 118793 118802 2 200 2 0.01 ATTGTCGTCTTCATCGGCTT

MT946551.1 119034 119034 1 96 1 0.01 TGCATCGTGTT

MT946551.1 119313 119319 2 196 2 0.01 CACACCGCAGCCGTAGC

MT946551.1 119444 119449 2 200 2 0.01 CATATCGAACCGATGG

MT946551.1 121537 121537 1 96 1 0.01 TAGTTCGTATC

MT946551.1 125515 125515 1 99 1 0.01 AAAACCGTTAA

MT946551.1 125763 125770 2 200 2 0.01 TACAACGGATATCGATGT

MT946551.1 126019 126019 1 100 1 0.01 ATTGTCGTCTT

MT946551.1 126322 126325 2 210 2 0.01 TTAGACGACGTATT

MT946551.1 126867 126870 2 204 2 0.01 GATATCGACGGAAG

MT946551.1 127541 127541 1 97 1 0.01 TAGAACGTAAA

MT946551.1 127679 127679 1 101 1 0.01 AAGGACGTAAT

MT946551.1 129142 129147 2 192 2 0.01 CCAGACGTGACGTGTA

MT946551.1 130283 130283 1 103 1 0.01 TTCAACGTATA

MT946551.1 130450 130450 1 104 1 0.01 GTAATCGTAGC

MT946551.1 131333 131338 2 206 2 0.01 AAATCCGGAACGTATA

MT946551.1 132291 132297 2 204 2 0.01 TCTGCCGTAACCGACAG

MT946551.1 132495 132495 1 97 1 0.01 AGACCCGCATA

MT946551.1 133105 133110 2 208 2 0.01 CTAATCGTCTCGGATG

MT946551.1 133145 133145 1 103 1 0.01 TTATACGCATA

MT946551.1 135216 135222 2 200 2 0.01 TGTACCGCTCCCGTTTG

MT946551.1 135574 135574 1 98 1 0.01 TCTGCCGATTT

MT946551.1 137138 137138 1 100 1 0.01 GTAATCGAAAT

MT946551.1 137758 137766 2 194 2 0.01 ACCACCGATGATGCGGATC

MT946551.1 137781 137781 1 104 1 0.01 TGATACGTACA

MT946551.1 139483 139483 1 97 1 0.01 TACTGCGACTT

MT946551.1 142653 142653 1 98 1 0.01 TGTTTCGATGA

MT946551.1 143793 143796 2 202 2 0.01 GGTCCCGACGATGA

MT946551.1 144278 144278 1 102 1 0.01 AATCACGTGTT

MT946551.1 145804 145804 1 104 1 0.01 GAGTTCGAAAG

MT946551.1 18413 18428 3 330 3 0.009 TTAAACGTATAAAAACGTTCCGTATC

MT946551.1 18714 18720 2 222 2 0.009 ACCTCCGCATACGCATT

MT946551.1 19975 19978 2 218 2 0.009 AACTCCGCCGTAGT

MT946551.1 21202 21202 1 107 1 0.009 GGATACGAAGA

MT946551.1 21312 21312 1 107 1 0.009 TAGGACGCCTA

MT946551.1 21664 21664 1 107 1 0.009 AACTGCGTTCA

MT946551.1 23117 23117 1 107 1 0.009 GGAGGCGATGA

MT946551.1 31904 31912 2 214 2 0.009 CAAATCGATAGTGCGGAGT

MT946551.1 37570 37570 1 107 1 0.009 CTTACCGCCTT

MT946551.1 43806 43806 1 111 1 0.009 CCAGTCGAAAC

MT946551.1 44193 44193 1 107 1 0.009 CTAATCGATTT

MT946551.1 45006 45019 3 345 3 0.009 AATACCGTTACTGGCGTACGATCC

MT946551.1 45084 45084 1 107 1 0.009 TGCACCGTAAT

MT946551.1 50906 50906 1 108 1 0.009 ACATACGTATA

MT946551.1 58705 58705 1 107 1 0.009 GGAACCGCACC

MT946551.1 59403 59407 2 214 2 0.009 ACATTCGTTCGACCT

MT946551.1 69242 69248 2 214 2 0.009 GAAAACGTCAACGTGTA

MT946551.1 70277 70277 1 108 1 0.009 GTAGGCGTACA

MT946551.1 71056 71059 2 216 2 0.009 TTTGGCGGCGTTAT

MT946551.1 71474 71474 1 110 1 0.009 CTTTTCGTTTC

MT946551.1 71793 71793 1 108 1 0.009 TAATACGAGTT

MT946551.1 71992 71992 1 107 1 0.009 TTCTTCGAATG

MT946551.1 72389 72392 2 212 2 0.009 TCTAACGCCGATCT

MT946551.1 75629 75629 1 108 1 0.009 TTATACGTTTT

MT946551.1 75827 75831 2 220 2 0.009 AACTACGTTCGATGA

MT946551.1 76495 76501 2 218 2 0.009 TCTACCGCATACGAAAT

MT946551.1 77950 77950 1 106 1 0.009 ATAGTCGAACA

MT946551.1 88479 88479 1 114 1 0.009 GCCACCGCCTT

MT946551.1 91863 91863 1 108 1 0.009 ATAAACGTATA

MT946551.1 92160 92169 2 224 2 0.009 AATTTCGAAGCTAGCGATAG

MT946551.1 102176 102176 1 108 1 0.009 CATTGCGCAGA

MT946551.1 104523 104528 2 218 2 0.009 TCCTACGTTTCGTAGA

MT946551.1 111175 111183 2 234 2 0.009 TAGAACGCTCTTTCGATGA

MT946551.1 111849 111849 1 106 1 0.009 CAGTTCGTCTC

MT946551.1 111910 111910 1 111 1 0.009 ATATGCGCCTT

MT946551.1 115533 115533 1 108 1 0.009 CAATACGATTT

MT946551.1 115863 115869 2 214 2 0.009 AGAGGCGGTCTCGATAT

MT946551.1 117370 117370 1 109 1 0.009 CAATGCGAGGA

MT946551.1 123613 123613 1 112 1 0.009 TAGTACGTATA

MT946551.1 124991 124991 1 107 1 0.009 AACCTCGAGCA

MT946551.1 126288 126288 1 114 1 0.009 TGAAACGAGTG

MT946551.1 126676 126676 1 116 1 0.009 GTTTACGAGGA

MT946551.1 129102 129102 1 110 1 0.009 CATACCGCACT

MT946551.1 129886 129886 1 116 1 0.009 CATATCGTATA

MT946551.1 130130 130130 1 107 1 0.009 CTACTCGATTA

MT946551.1 132758 132758 1 112 1 0.009 GTTATCGGGAT

MT946551.1 132794 132794 1 110 1 0.009 ACCCTCGATCA

MT946551.1 134201 134217 3 333 3 0.009 AACTACGTATGCCGAAGTACTCGGTTC

MT946551.1 143306 143313 2 216 2 0.009 TAAACCGCTATACGAAGT

MT946551.1 143346 143349 2 220 2 0.009 TGCAACGGCGAAAC

MT946551.1 144257 144257 1 108 1 0.009 TAGTACGTACT

MT946551.1 144382 144382 1 110 1 0.009 TTTTTCGATTA

MT946551.1 45587 45597 3 378 3 0.008 ACAATCGTTCGAACTCGTCTC

MT946551.1 92134 92134 1 120 1 0.008 TTAAGCGCAGT

MT946551.1 130049 130058 2 238 2 0.008 GAAAGCGCATAATACGATCT

MT946551.1 134991 134991 1 118 1 0.008 TAAGGCGTTTA

MT946551.1 74 76 2 28 0 0 GAAGTCGCGAGAG

MT946551.1 103 103 1 20 0 0 CAAACCGGAAT

MT946551.1 151 155 2 66 0 0 TGAATCGTTCGATTA

MT946551.1 196 196 1 30 0 0 GTTATCGATTC

MT946551.1 266 274 3 81 0 0 GATGCCGATCCGTCGACAT

MT946551.1 319 328 3 93 0 0 GTCTCCGATACGAACGCTAA

MT946551.1 361 376 4 132 0 0 TGTATCGACGATCGTTGAACCGATGA

MT946551.1 573 573 1 31 0 0 TCCACCGATGA

MT946551.1 645 645 1 22 0 0 TGACTCGGACT

MT946551.1 661 666 2 62 0 0 ATTTCCGTTACGGATT

MT946551.1 687 691 2 62 0 0 ATCATCGGTCGGTGT

MT946551.1 760 766 2 74 0 0 TCTTCCGTACACGATGA

MT946551.1 908 908 1 23 0 0 TTTTACGAGAA

MT946551.1 993 1002 2 56 0 0 TATATCGCAACTACCGGGCA

MT946551.1 1049 1049 1 36 0 0 AGTTTCGTAGT

MT946551.1 1091 1094 2 64 0 0 GATGTCGACGCTAG

MT946551.1 1112 1118 2 88 0 0 GTCTCCGAATGCGGCAT

MT946551.1 1145 1145 1 32 0 0 GCTTTCGTTAA

MT946551.1 1189 1189 1 39 0 0 AATCTCGAAAT

MT946551.1 1253 1259 2 76 0 0 GAAGACGGGATCGCAGT

MT946551.1 1293 1296 2 84 0 0 AGTTCCGACGTTGA

MT946551.1 1310 1349 6 246 0 0 GGATTCGCTGAGACCGGTAGTGGTCGTCCGAGTACACGATGTGTCGTTAA

MT946551.1 1406 1412 2 70 0 0 GGGTACGGGTTCGCATT

MT946551.1 1424 1433 2 68 0 0 ATCTGCGGAAGAGACGGTGT

MT946551.1 1475 1490 3 135 0 0 GATGACGTCTCCGGATACTCCGTATC

MT946551.1 1572 1590 5 175 0 0 TGAGTCGTGTTACACGATCGCGTCGACAT

MT946551.1 1641 1641 1 46 0 0 AAATACGTTAG

MT946551.1 1665 1676 4 152 0 0 ACTGTCGCGAACGATGCGTCCA

MT946551.1 1759 1759 1 46 0 0 TTATGCGAGAC

MT946551.1 1780 1780 1 35 0 0 AACTCCGTGTT

MT946551.1 1819 1825 2 76 0 0 TTGGACGGAATCGATTT

MT946551.1 1963 1963 1 45 0 0 GGAGACGCCTG

MT946551.1 2237 2250 3 147 0 0 ATTATCGAGCGTGACACTCGCTCC

MT946551.1 2297 2303 3 150 0 0 GACATCGACCGCGTAAT

MT946551.1 2388 2388 1 48 0 0 ATACCCGCAAT

MT946551.1 2404 2404 1 53 0 0 AATCTCGATAA

MT946551.1 2418 2418 1 53 0 0 CAGACCGTACA

MT946551.1 2461 2463 2 88 0 0 ACAGCCGCGTGTC

MT946551.1 2492 2507 4 180 0 0 CCTTACGACGTTTACATCGACGAGAT

MT946551.1 2535 2538 2 64 0 0 TTGTCCGTCGTCTT

MT946551.1 2582 2585 2 112 0 0 CATATCGCCGTAAT

MT946551.1 2618 2618 1 54 0 0 TTGTACGATGG

MT946551.1 2690 2694 2 102 0 0 GGAATCGTACGAAGT

MT946551.1 2707 2717 3 171 0 0 TACTACGCATTACGTCGACAA

MT946551.1 2828 2831 2 106 0 0 GCTGACGTCGTATA

MT946551.1 3023 3036 3 195 0 0 AGGTACGAAATACGTAACCGGTGC

MT946551.1 3120 3120 1 48 0 0 CAATCCGAGAC

MT946551.1 3154 3164 3 165 0 0 TTGGACGGACGTATCCGTTTA

MT946551.1 3345 3353 3 159 0 0 TCTCTCGACTCCGCGAGAG

MT946551.1 3418 3418 1 62 0 0 TGTAGCGCATT

MT946551.1 3766 3766 1 60 0 0 TTTTGCGTAAT

MT946551.1 3803 3809 2 120 0 0 TTTGACGACCTCGACAT

MT946551.1 3842 3842 1 69 0 0 GTATGCGTGTA

MT946551.1 4418 4424 3 213 0 0 AGGAACGCGTACGAGAA

MT946551.1 4451 4451 1 55 0 0 TCTAACGGTAG

MT946551.1 4467 4474 2 114 0 0 ATAAACGATTATCGTCAA

MT946551.1 4611 4611 1 55 0 0 CATATCGTTTC

MT946551.1 4680 4688 2 102 0 0 TTTGACGCATATCCGCAAT

MT946551.1 4702 4708 2 124 0 0 TTTTTCGCAGTCGGTTA

MT946551.1 5098 5098 1 55 0 0 ATATACGGCTT

MT946551.1 5421 5421 1 73 0 0 TATTACGTAAA

MT946551.1 5453 5464 3 201 0 0 TAAAACGTATCACGGTCGAATA

MT946551.1 5578 5587 3 201 0 0 ACTATCGAATAACGCGTCAA

MT946551.1 5601 5611 2 106 0 0 TTTCCCGTTTAATATCGCATT

MT946551.1 5797 5803 2 126 0 0 GTAGTCGTAATCGTAAT

MT946551.1 5959 5959 1 52 0 0 TGTAGCGTATT

MT946551.1 6100 6107 2 136 0 0 ATAGTCGACTGACGACAA

MT946551.1 6357 6357 1 74 0 0 TAGCACGTTTG

MT946551.1 6580 6580 1 69 0 0 ATATTCGTCCC

MT946551.1 6628 6634 2 106 0 0 GTATACGCTAACGGTAT

MT946551.1 6671 6671 1 66 0 0 ATAACCGTAAT

MT946551.1 6967 6976 2 102 0 0 CTATACGGGATTAACGGATG

MT946551.1 7149 7159 2 162 0 0 TATTACGTAATAGAACGTGTC

MT946551.1 7224 7234 3 186 0 0 GACCTCGTCGATTTCCGACCA

MT946551.1 7308 7308 1 56 0 0 GCATCCGTAGT

MT946551.1 7345 7347 2 100 0 0 CTCAACGCGATAT

MT946551.1 7407 7420 4 292 0 0 ATGAACGAATACCGACGGCGTTAA

MT946551.1 7578 7583 2 150 0 0 TATATCGCTACGAATT

MT946551.1 7723 7729 2 132 0 0 TATTCCGGCAGCGAACT

MT946551.1 7858 7868 2 154 0 0 ACTGTCGAATGACATCGCATT

MT946551.1 7934 7943 2 174 0 0 TTAAACGTTTCCATCGCTTT

MT946551.1 7954 7960 2 138 0 0 AAAGACGTTTCCGATAG

MT946551.1 8048 8053 2 146 0 0 GAAAACGAATCGGCTT

MT946551.1 8100 8100 1 78 0 0 TTTTTCGTTGT

MT946551.1 8223 8225 2 142 0 0 TAGGACGCGTAAT

MT946551.1 8339 8354 3 234 0 0 AATAGCGATCATTCGCTTAGCGTCTT

MT946551.1 8402 8412 3 207 0 0 TGGACCGTCGGACAGCGTCAT

MT946551.1 8724 8748 4 352 0 0 CTCATCGTACAGGACGTTCTCGTACAGGACGTAAC

MT946551.1 8947 8947 1 85 0 0 TCAATCGCCAT

MT946551.1 9065 9065 1 96 0 0 AGGTGCGTTGT

MT946551.1 9117 9117 1 70 0 0 ACCTTCGGGTA

MT946551.1 9361 9371 2 182 0 0 TCCAGCGTTCAACAACGTACA

MT946551.1 9384 9388 2 182 0 0 CTAGACGCACGTTAT

MT946551.1 9611 9611 1 77 0 0 GCTATCGCAAA

MT946551.1 9825 9828 2 178 0 0 GATCTCGCCGTAGC

MT946551.1 9872 9875 2 172 0 0 TTGTTCGTCGACAT

MT946551.1 9942 9945 2 164 0 0 TTAAACGACGCATT

MT946551.1 9983 9990 2 186 0 0 CATTTCGGCTATCGACTT

MT946551.1 10245 10256 3 276 0 0 GGAACCGTTTTCGTACCGTACT

MT946551.1 10397 10405 2 190 0 0 CATACCGGATCTACGTTCT

MT946551.1 10491 10494 2 150 0 0 TAATACGACGGTTT

MT946551.1 10548 10548 1 89 0 0 GTGTACGTATA

MT946551.1 10677 10677 1 71 0 0 AATAGCGACAT

MT946551.1 10695 10695 1 83 0 0 GAAAACGAATA

MT946551.1 10791 10806 3 243 0 0 GTAGACGCTGAACACGTTAACGATAG

MT946551.1 11109 11113 2 198 0 0 TACATCGCCCGCATT

MT946551.1 11154 11160 2 158 0 0 GTTAACGGGCTCGTAAA

MT946551.1 11313 11313 1 84 0 0 AGTATCGTACA

MT946551.1 11470 11478 2 174 0 0 TAGGCCGTTTTATCGGTTA

MT946551.1 11655 11655 1 50 0 0 CTTAACGGATA

MT946551.1 11725 11731 2 186 0 0 TTACCCGCCTTCGAATA

MT946551.1 12081 12081 1 76 0 0 GTTAGCGCTTC

MT946551.1 12245 12245 1 79 0 0 TAACTCGTACT

MT946551.1 12289 12301 3 210 0 0 ATTATCGTGCCGAAATTCGTACC

MT946551.1 12657 12657 1 71 0 0 AACATCGCTAA

MT946551.1 12694 12694 1 77 0 0 AGAGTCGGGGT

MT946551.1 13046 13046 1 87 0 0 TACCACGTGCT

MT946551.1 13158 13166 2 168 0 0 TAGTTCGTCATAACGTCCA

MT946551.1 13463 13465 2 114 0 0 GTAGTCGCGACTC

MT946551.1 13693 13693 1 81 0 0 TTATTCGCTAC

MT946551.1 13867 13867 1 85 0 0 CATAACGCATA

MT946551.1 13880 13880 1 83 0 0 TATTACGTTAG

MT946551.1 13987 13989 2 158 0 0 ATTCACGCGTGCT

MT946551.1 14380 14380 1 89 0 0 AGTATCGAACA

MT946551.1 14920 14926 2 194 0 0 ATAATCGTAAGCGCTAT

MT946551.1 14944 14957 3 264 0 0 ATATCCGGCAGCACCCGGCGATTG

MT946551.1 15282 15294 3 252 0 0 TCTACCGAACACTAACGCGCATG

MT946551.1 15497 15497 1 96 0 0 ACCAACGCTCA

MT946551.1 15552 15561 2 186 0 0 GTTAGCGGGTATCCCGCTAG

MT946551.1 16196 16196 1 81 0 0 AAGCACGTAAT

MT946551.1 16209 16212 2 134 0 0 ATTGACGACGTTAT

MT946551.1 16572 16579 2 174 0 0 CCACACGTTTTTCGAAAA

MT946551.1 16849 16855 2 200 0 0 ACATGCGAAGTCGCAGT

MT946551.1 16888 16906 3 279 0 0 CAATTCGTTGGAAAACGTGAGTCCGGGCA

MT946551.1 17008 17011 2 174 0 0 ATAACCGGCGCTGT

MT946551.1 17220 17225 2 164 0 0 AAAATCGTTCCGCCAA

MT946551.1 17529 17545 4 424 0 0 TACATCGATATCGCCGCAATTCGGTTT

MT946551.1 17611 17611 1 53 0 0 AAATCCGTGGT

MT946551.1 17868 17875 2 180 0 0 CTACACGTGCTTCGGATT

MT946551.1 17995 17999 2 188 0 0 CATGACGAGCGTCAT

MT946551.1 18274 18287 3 294 0 0 GATGGCGGTGTACGTGGACGAGGC

MT946551.1 18314 18332 4 360 0 0 GTAGTCGCCGACTGTCGTGTCTGCGTTTA

MT946551.1 18352 18360 3 213 0 0 ATTATCGCGATTGCGTAGA

MT946551.1 18519 18545 5 540 0 0 AACCACGGATCGTAATACGGCAATCGGTTTTCGATAA

MT946551.1 18612 18614 2 206 0 0 GCTTCCGCGGATC

MT946551.1 18653 18663 3 324 0 0 TTGACCGGGCGGAGCCGAACA

MT946551.1 18764 18764 1 101 0 0 TACATCGTCTA

MT946551.1 18898 18905 3 246 0 0 TTATTCGGAGCGCGCAAT

MT946551.1 19185 19185 1 96 0 0 AAATTCGATAT

MT946551.1 19245 19245 1 111 0 0 GAAATCGTAAT

MT946551.1 19326 19346 3 321 0 0 AAAGTCGTTTAATGCCGATTTAATACGTTCA

MT946551.1 19437 19437 1 95 0 0 ATCTGCGTGTA

MT946551.1 19617 19623 2 206 0 0 TCTTTCGTATTCGTAGT

MT946551.1 19758 19765 2 196 0 0 TCTCTCGTTAAACGCCTT

MT946551.1 19853 19853 1 76 0 0 GAACACGTTTA

MT946551.1 19906 19914 2 220 0 0 GCCCACGCACACACGATTA

MT946551.1 20013 20013 1 109 0 0 GGCCTCGAATA

MT946551.1 20724 20740 3 333 0 0 GAATGCGAGCAGCGATTTTGGCGTTAT

MT946551.1 21100 21100 1 68 0 0 ATGACCGGTTA

MT946551.1 21751 21758 2 208 0 0 TAATTCGACTATCGAATA

MT946551.1 21790 21797 3 258 0 0 AATATCGACGGACGTCAA

MT946551.1 21835 21835 1 86 0 0 ATCATCGTGAT

MT946551.1 21887 21889 2 166 0 0 CACAACGCGATAT

MT946551.1 22204 22213 2 216 0 0 TGTCTCGAATGCCTCGTACA

MT946551.1 22294 22302 2 214 0 0 TAAATCGAATGGTCGGCTC

MT946551.1 22466 22474 2 212 0 0 AGCAACGGTATATCGAATA

MT946551.1 22598 22604 2 178 0 0 TTACCCGGATACGTGGA

MT946551.1 22660 22663 2 216 0 0 TGATACGGCGATGG

MT946551.1 22762 22769 3 318 0 0 AATTTCGGGACGCGTATA

MT946551.1 22969 22974 2 212 0 0 AATACCGTATCGTTCT

MT946551.1 23005 23036 5 555 0 0 CTGTTCGTGAGAACGCTTTCGTATAATATCGATCAACGGATA

MT946551.1 23344 23353 2 226 0 0 GATGGCGTTCTATACGTTTC

MT946551.1 23490 23503 3 273 0 0 GGTTCCGTCGAAATTTGCCGAAGT

MT946551.1 23523 23529 3 222 0 0 ATATTCGTCGTCGACTA

MT946551.1 23739 23747 3 273 0 0 ATTACCGTCGACACGTGTA

MT946551.1 23935 23935 1 105 0 0 CAAGCCGCAGA

MT946551.1 24046 24055 2 218 0 0 CCTAACGTTTTAATCGTATG

MT946551.1 24082 24084 2 114 0 0 AATGACGCGTTTC

MT946551.1 24372 24372 1 87 0 0 AATTTCGTTAA

MT946551.1 24457 24466 2 182 0 0 GCTCCCGCAGGTACCGGTGC

MT946551.1 24539 24548 2 188 0 0 ATCATCGGCTATATCGCTAA

MT946551.1 24858 24858 1 100 0 0 CAATACGTTAG

MT946551.1 25034 25041 2 138 0 0 TCATCCGTAAACCGATAA

MT946551.1 25297 25297 1 81 0 0 GATGTCGATGG

MT946551.1 25337 25337 1 97 0 0 AGCATCGATTT

MT946551.1 25575 25583 3 246 0 0 ATTGACGAATCGTCGAAAA

MT946551.1 25707 25710 2 172 0 0 TGCATCGACGCTTC

MT946551.1 25802 25812 2 190 0 0 TACTACGATTGTATGCGTTGA

MT946551.1 25910 25910 1 94 0 0 TATTGCGCATC

MT946551.1 26130 26148 3 309 0 0 AGATGCGTCTAGAAGCGATGCTACGCTAG

MT946551.1 26325 26325 1 89 0 0 GTATGCGATGT

MT946551.1 26367 26367 1 87 0 0 TCCTGCGTACT

MT946551.1 26397 26399 2 96 0 0 TCACCCGCGTGTG

MT946551.1 26499 26511 4 264 0 0 ATGTTCGCGGCTAATCGCGATAA

MT946551.1 26576 26581 2 152 0 0 AAATCCGTGTCGTCCA

MT946551.1 26834 26855 4 376 0 0 ACTCTCGAAATCACACGATGTGTCGGCGTTAA

MT946551.1 27055 27057 2 156 0 0 CCATACGCGCATT

MT946551.1 27374 27382 3 288 0 0 TATATCGCCGTACCGTATA

MT946551.1 27419 27419 1 74 0 0 TGATCCGTAAC

MT946551.1 27445 27445 1 91 0 0 TATGGCGTCTA

MT946551.1 27536 27539 2 188 0 0 TACTGCGACGTTCA

MT946551.1 27890 27890 1 87 0 0 ATATTCGTTCA

MT946551.1 28100 28100 1 100 0 0 GAGCTCGCTCT

MT946551.1 28337 28337 1 81 0 0 GATACCGCATT

MT946551.1 28542 28542 1 92 0 0 TTCTTCGCTCT

MT946551.1 28729 28737 2 180 0 0 ATATACGATGTTACGATTT

MT946551.1 28818 28820 2 150 0 0 TAGTACGCGGAAA

MT946551.1 29041 29053 3 282 0 0 AGTATCGATGTCGATCTCGTCTA

MT946551.1 29194 29203 2 230 0 0 TAATACGTCTTGCACGTAAT

MT946551.1 29372 29372 1 100 0 0 ATATTCGCATA

MT946551.1 29419 29419 1 93 0 0 TTCAACGTATG

MT946551.1 29523 29530 2 180 0 0 AGAAACGGATAGCGTCCC

MT946551.1 30106 30106 1 96 0 0 GTCCTCGTGCA

MT946551.1 30158 30165 2 206 0 0 AGAAACGCTCTACGAATA

MT946551.1 30207 30209 2 108 0 0 CTAGTCGCGTTAA

MT946551.1 30421 30424 2 150 0 0 TCTTCCGTCGATAT

MT946551.1 30616 30618 2 188 0 0 TTTTTCGCGGGAT

MT946551.1 30662 30677 3 222 0 0 GCATCCGGCTTATCCGCCTCCGTTGT

MT946551.1 30706 30709 2 144 0 0 AATATCGTCGGAGC

MT946551.1 30814 30814 1 106 0 0 TCCTTCGATTC

MT946551.1 30836 30836 1 91 0 0 ATAGCCGCACA

MT946551.1 30853 30865 3 324 0 0 CTCTGCGTCAGAACGCTCGTCAA

MT946551.1 30964 30976 3 300 0 0 TGGTTCGTCGTTTCTATCGAATG

MT946551.1 30997 30997 1 80 0 0 TAACCCGTCAT

MT946551.1 31088 31094 2 210 0 0 TGTATCGATTGCGTTGT

MT946551.1 31214 31217 2 216 0 0 ATCATCGGCGTAGT

MT946551.1 31264 31273 3 282 0 0 TTTCTCGTCGAGAACGTTAA

MT946551.1 31381 31387 2 170 0 0 TTTTTCGGTGCCGCTAG

MT946551.1 32232 32232 1 98 0 0 TCATTCGTTAT

MT946551.1 32581 32591 2 204 0 0 CAAAGCGTACAGATACGATGA

MT946551.1 33150 33168 4 448 0 0 GTGTACGAAGCGGTTTTACGACACGCATT

MT946551.1 33185 33192 2 204 0 0 ACCCACGTTGTACGTTAA

MT946551.1 33221 33231 2 212 0 0 TTTATCGTTTAGTAACGCAAT

MT946551.1 33584 33584 1 98 0 0 AGAAACGTTTA

MT946551.1 33739 33741 2 120 0 0 AAGGACGCGTTAA

MT946551.1 33984 34003 3 294 0 0 GATCCCGAATATTTCGATAATGAACGAATT

MT946551.1 34025 34034 2 178 0 0 CTCTACGAGTGCTGCGGACA

MT946551.1 34155 34166 3 255 0 0 TTTAACGAGGATACGTCGTATT

MT946551.1 34206 34211 2 162 0 0 TTGCACGAGTCGGATC

MT946551.1 34416 34426 3 303 0 0 GTATCCGCATTCGTTCGATTT

MT946551.1 35049 35049 1 61 0 0 TATAACGGATG

MT946551.1 35144 35146 2 126 0 0 TGTAACGCGTCTG

MT946551.1 35323 35341 5 435 0 0 GCCACCGTTCCGCGTTTTGACGACGTGTA

MT946551.1 35443 35445 2 194 0 0 AATTACGCGTATG

MT946551.1 35545 35545 1 107 0 0 AAGTTCGAGTT

MT946551.1 35814 35814 1 105 0 0 CCTGGCGTTGG

MT946551.1 36115 36118 2 212 0 0 AAGAGCGACGTACA

MT946551.1 36229 36240 3 321 0 0 TATTTCGGACGTCAATCGTTTA

MT946551.1 36480 36480 1 106 0 0 GCATTCGACTC

MT946551.1 36898 36904 2 190 0 0 AAACACGGAGTCGGTAT

MT946551.1 36919 36930 3 324 0 0 ATACACGCTACGAAAACGAAAT

MT946551.1 37023 37033 2 194 0 0 CAAACCGTAACATACCGTTAG

MT946551.1 37112 37117 2 188 0 0 TTAGCCGAAGCGTATG

MT946551.1 37343 37343 1 94 0 0 AGGTTCGGTAG

MT946551.1 37367 37367 1 84 0 0 TGAACCGTAAT

MT946551.1 37466 37466 1 75 0 0 GTTTCCGGAGA

MT946551.1 37678 37678 1 98 0 0 ATATTCGAATA

MT946551.1 37712 37720 3 231 0 0 GTTGACGCACCCGCGTCTG

MT946551.1 38203 38210 2 174 0 0 ATTAACGTGAAACGTAGT

MT946551.1 38383 38396 4 412 0 0 GGTAACGACGTAGTCGAACGTTAG

MT946551.1 38460 38464 2 176 0 0 GTTCTCGTTCGTAAT

MT946551.1 38905 38908 2 190 0 0 ATCTGCGACGGAGC

MT946551.1 38984 38990 2 218 0 0 CCCAACGGCCTCGCATT

MT946551.1 39028 39040 3 297 0 0 TATTTCGTCCGTAACTACGTAAT

MT946551.1 39063 39087 4 392 0 0 GAAATCGTATAAATACGGTCTCGCCATTTCGACAT

MT946551.1 39247 39263 3 270 0 0 ACTTTCGCAAGCGGGTTTAGACGGCAA

MT946551.1 39333 39347 4 324 0 0 GTAGACGGCACGCGACTATCGCTAT

MT946551.1 39377 39377 1 80 0 0 TCTAGCGATGA

MT946551.1 39446 39452 3 291 0 0 AAATACGCGATCGATGT

MT946551.1 39525 39527 2 96 0 0 ATAACCGCGTGAT

MT946551.1 39588 39588 1 54 0 0 ACCCCCGTTAC

MT946551.1 39794 39797 2 168 0 0 TACATCGTCGGTAT

MT946551.1 39854 39854 1 74 0 0 ATTAACGAGTT

MT946551.1 39924 39924 1 75 0 0 AGGTACGGTAC

MT946551.1 40418 40418 1 98 0 0 CTGTGCGTAGA

MT946551.1 40430 40430 1 100 0 0 CACATCGTACA

MT946551.1 40541 40541 1 87 0 0 TAACACGGTAT

MT946551.1 41032 41035 2 148 0 0 GTCCTCGTCGATAA

MT946551.1 41510 41510 1 70 0 0 GGTCCCGATAC

MT946551.1 41593 41593 1 76 0 0 CTTAACGATAT

MT946551.1 41605 41605 1 71 0 0 AGTGACGTTTA

MT946551.1 41809 41812 2 172 0 0 CATGTCGGCGACAT

MT946551.1 42294 42297 2 160 0 0 AATTACGACGAGCA

MT946551.1 42476 42480 2 164 0 0 AATAGCGTTCGTCAA

MT946551.1 42631 42633 2 176 0 0 TAGTACGCGAAAT

MT946551.1 43102 43102 1 102 0 0 GAATGCGTAAT

MT946551.1 43355 43355 1 83 0 0 ACAACCGGTAT

MT946551.1 43622 43628 2 196 0 0 GAGTCCGATAACGTTAG

MT946551.1 43670 43670 1 116 0 0 GAGTGCGAATG

MT946551.1 43980 43982 2 214 0 0 CTAGCCGCGGTAA

MT946551.1 44159 44165 2 210 0 0 TGTAACGTATTCGATAC

MT946551.1 44217 44217 1 113 0 0 ACACCCGCACA

MT946551.1 44403 44409 2 178 0 0 GATTTCGGAATCGCAAA

MT946551.1 44463 44480 4 476 0 0 GGGAGCGGATGCGGGTTTCGTACGTAGA

MT946551.1 44554 44554 1 79 0 0 TAACTCGGATC

MT946551.1 44839 44848 2 224 0 0 GTATACGGTTCCACCGACTC

MT946551.1 44968 44971 2 172 0 0 AGATCCGACGGTAC

MT946551.1 45099 45099 1 107 0 0 GGTTTCGAAGA

MT946551.1 45163 45169 3 210 0 0 ATAAACGCGTCCGCTAA

MT946551.1 45517 45517 1 120 0 0 ATAATCGCTTT

MT946551.1 45846 45846 1 98 0 0 TCTAGCGGTGT

MT946551.1 45978 45991 3 363 0 0 TAATGCGCATCGTTTTAACGTGTC

MT946551.1 46069 46069 1 113 0 0 GTTCCCGCATT

MT946551.1 46092 46096 2 194 0 0 AGAAGCGTGCGTAAA

MT946551.1 46170 46175 2 216 0 0 TACTGCGACACGCATT

MT946551.1 46277 46281 2 198 0 0 AGTCTCGTTCGTAAA

MT946551.1 46534 46534 1 92 0 0 TCTACCGTTGT

MT946551.1 46676 46676 1 101 0 0 CATTTCGTTTA

MT946551.1 46810 46820 3 324 0 0 AAAATCGTTACGGCTCGATTA

MT946551.1 46884 46887 2 186 0 0 CATGGCGGCGCCAG

MT946551.1 46926 46930 2 216 0 0 TATGCCGATCGCAGT

MT946551.1 47243 47243 1 109 0 0 CACACCGCCTC

MT946551.1 47322 47324 2 100 0 0 GGACTCGCGTTTA

MT946551.1 47340 47352 3 321 0 0 GTGAACGTCTTAGGCGTCGGAAA

MT946551.1 47516 47526 2 192 0 0 TATTTCGATGATATACGGTAG

MT946551.1 47631 47640 2 204 0 0 AAAAACGAAATTATCGACAT

MT946551.1 47816 47826 4 292 0 0 ATCATCGTCGACGCCCGTCAA

MT946551.1 47915 47921 2 190 0 0 CTTCTCGTAGGCGAATG

MT946551.1 48126 48129 2 142 0 0 TTCATCGTCGTTTA

MT946551.1 48206 48206 1 97 0 0 CCATACGTTTA

MT946551.1 48228 48241 3 270 0 0 TCCTGCGTTATCCGTTTCCGTTAT

MT946551.1 48495 48495 1 104 0 0 AACATCGATGT

MT946551.1 48763 48765 2 120 0 0 ATAAACGCGTCAA

MT946551.1 48875 48875 1 89 0 0 TACTTCGATTG

MT946551.1 49019 49025 2 176 0 0 TTTCCCGTAGTCGAGGT

MT946551.1 49107 49110 2 182 0 0 TAACTCGTCGGTTA

MT946551.1 49131 49140 3 279 0 0 TTGATCGAGTTCGTCGCTAC

MT946551.1 49218 49221 2 188 0 0 TTTGCCGGCGGTTG

MT946551.1 49257 49257 1 61 0 0 ATATCCGTTAC

MT946551.1 49492 49497 2 134 0 0 TAAAACGGAACGTGAT

MT946551.1 49550 49556 2 198 0 0 CATAACGCACACGTTGT

MT946551.1 49637 49637 1 91 0 0 AAACCCGCACA

MT946551.1 50329 50329 1 57 0 0 AGTAGCGAGAA

MT946551.1 50762 50772 2 198 0 0 TCCAGCGTTACTATACGCAAT

MT946551.1 51076 51080 2 194 0 0 AGACTCGTTCGATAT

MT946551.1 51539 51539 1 93 0 0 ATAATCGTATA

MT946551.1 51864 51864 1 71 0 0 TACATCGCAGC

MT946551.1 51952 51962 2 194 0 0 TTACACGTATTCCTTCGGCAT

MT946551.1 52026 52039 4 352 0 0 AGAGACGCGTCTCCGTATCGTATA

MT946551.1 52121 52121 1 87 0 0 GGTTGCGTCAA

MT946551.1 52397 52402 2 166 0 0 AGCCTCGCCACGAAGA

MT946551.1 52569 52569 1 65 0 0 GGAGACGGAGT

MT946551.1 52703 52716 3 267 0 0 AATTACGATATTCGATCCCGATAT

MT946551.1 52802 52818 3 291 0 0 TAAATCGCCTCCGCTAAGAAACGTTAG

MT946551.1 52964 52974 3 225 0 0 GATTGCGTCCGATTCCGTTGC

MT946551.1 53117 53123 2 160 0 0 ATTCTCGAATCCGAAAT

MT946551.1 53161 53165 2 138 0 0 TCATCCGATCGTTGA

MT946551.1 53333 53344 4 300 0 0 ATTTGCGCACTCGCGACGGTTG

MT946551.1 53800 53804 2 160 0 0 GTATACGGCCGTGTT

MT946551.1 54237 54245 2 208 0 0 ACCAGCGATTTATCGGTTT

MT946551.1 54345 54345 1 96 0 0 AGATGCGCTGC

MT946551.1 54371 54380 3 261 0 0 GGTTCCGCGCTTTACGTCAC

MT946551.1 54504 54512 2 176 0 0 CAATACGGTTTACCGAAAA

MT946551.1 54561 54561 1 79 0 0 AAAATCGTTAC

MT946551.1 54696 54696 1 82 0 0 AACTACGGTAT

MT946551.1 54787 54787 1 81 0 0 CAAGACGTTAG

MT946551.1 55043 55057 3 255 0 0 ATGAACGAGACGCAGAATTCGTTAT

MT946551.1 55209 55209 1 78 0 0 TCCCTCGGCAG

MT946551.1 55239 55242 2 178 0 0 ATTAACGGCGTTAG

MT946551.1 55396 55406 3 279 0 0 TCTACCGTAGACGTGCGTAAT

MT946551.1 55419 55435 3 267 0 0 TGTTACGTTTATAAACGATTACGCTAA

MT946551.1 55865 55865 1 85 0 0 CAACTCGTGTG

MT946551.1 55876 55880 2 184 0 0 CTCTACGTGCGGCAA

MT946551.1 55898 55902 2 158 0 0 TCTCACGAACGATAT

MT946551.1 56171 56180 2 182 0 0 TGGTACGTTATTATCGGAAC

MT946551.1 56417 56425 2 188 0 0 TACATCGTCTAAACGAATA

MT946551.1 56580 56580 1 87 0 0 TCCCTCGTTCT

MT946551.1 56655 56659 2 162 0 0 CATATCGGACGCAGA

MT946551.1 57323 57331 2 196 0 0 TAAACCGTTTTAACGAATT

MT946551.1 57376 57386 2 182 0 0 TGAGCCGAATGACATCGAAGA

MT946551.1 57484 57490 2 182 0 0 TTTTTCGATGCCGGACA

MT946551.1 57602 57605 2 166 0 0 TAGAACGACGCTGT

MT946551.1 57768 57777 2 208 0 0 TTTGCCGCCTTGGGCGGATC

MT946551.1 57829 57837 3 309 0 0 TCTACCGCGGAGCCGAAAA

MT946551.1 58421 58421 1 96 0 0 TATGACGTTTT

MT946551.1 58487 58500 3 279 0 0 CTAAACGGGATCCGCCTCCGGGAG

MT946551.1 58721 58721 1 95 0 0 GCTGTCGCACT

MT946551.1 58869 58873 2 204 0 0 GGATACGTTCGATAG

MT946551.1 59033 59035 2 176 0 0 ATTTACGCGCACA

MT946551.1 59166 59174 2 176 0 0 ATATGCGGAACCTCGAGAA

MT946551.1 59228 59228 1 98 0 0 TAACACGTAAT

MT946551.1 59264 59264 1 109 0 0 TTGTACGATGT

MT946551.1 59485 59487 2 194 0 0 GGTGCCGCGGCAA

MT946551.1 59502 59505 2 190 0 0 ACAGACGACGGTGA

MT946551.1 59521 59532 3 327 0 0 CTCAGCGAACGTATCTCGTCTA

MT946551.1 59553 59557 2 206 0 0 AGAAGCGAACGCTAG

MT946551.1 59652 59658 3 204 0 0 CTCTGCGGACGCGGATG

MT946551.1 59772 59772 1 107 0 0 TCAGACGAGTG

MT946551.1 59833 59842 4 364 0 0 TCTAGCGCGGTCGTCGATAA

MT946551.1 59866 59866 1 103 0 0 CAAAACGTAAT

MT946551.1 60310 60310 1 78 0 0 TATAGCGGATG

MT946551.1 60472 60481 2 186 0 0 GTTGGCGTTGGTGGCGCTAG

MT946551.1 61055 61055 1 67 0 0 TGCCACGTTTA

MT946551.1 61190 61198 2 220 0 0 AAATACGTTTAGCCGCCTT

MT946551.1 61243 61246 2 146 0 0 ACCAACGACGATAG

MT946551.1 61551 61556 2 172 0 0 AACATCGGTACGGGTA

MT946551.1 61683 61686 2 188 0 0 ATTGCCGGCGCAAA

MT946551.1 62019 62026 2 180 0 0 ATTTTCGAAACTCGTAAA

MT946551.1 62064 62069 3 186 0 0 GAATCCGCGTCGACTG

MT946551.1 62405 62405 1 84 0 0 GCCCACGTTTA

MT946551.1 62530 62530 1 91 0 0 CTAAACGAAAA

MT946551.1 62560 62563 2 186 0 0 GATTACGACGTGCT

MT946551.1 62576 62586 2 188 0 0 TCTAGCGTGTGAAGACGATAA

MT946551.1 62869 62869 1 77 0 0 GAAGCCGTGGG

MT946551.1 63095 63106 4 248 0 0 ATCCTCGTCGCGATATCGCATT

MT946551.1 63181 63184 2 184 0 0 ATGAACGGCGGACA

MT946551.1 63245 63254 2 204 0 0 TTAGACGAGTTAGACGTTAT

MT946551.1 63280 63280 1 86 0 0 AAATGCGTGAC

MT946551.1 63496 63502 2 202 0 0 GTAGCCGCACTCGATGG

MT946551.1 63840 63840 1 90 0 0 GATTACGAACC

MT946551.1 63963 63963 1 92 0 0 GCCACCGTAGT

MT946551.1 64223 64226 2 172 0 0 TAGTACGGCGGATT

MT946551.1 64245 64245 1 89 0 0 AATTACGCTCT

MT946551.1 64500 64504 2 186 0 0 AAGATCGTCCGTAAC

MT946551.1 64780 64780 1 103 0 0 AGAAACGTACT

MT946551.1 65346 65346 1 69 0 0 GCTAACGCTAG

MT946551.1 65361 65361 1 68 0 0 CCAATCGGAAG

MT946551.1 65565 65575 3 303 0 0 AACAACGCATCGCTTCGTTTA

MT946551.1 65665 65672 2 168 0 0 TACATCGTATATCGTCTT

MT946551.1 65708 65712 2 168 0 0 TTGTTCGTCCGTCAT

MT946551.1 65845 65845 1 94 0 0 GGTTACGTATA

MT946551.1 65912 65927 4 324 0 0 AATGACGACGATATCGGTACCGTTAA

MT946551.1 66072 66072 1 97 0 0 TTATTCGTTTA

MT946551.1 66101 66134 6 594 0 0 CACTGCGGATTATTGCGTTCACGAGAACCGTATTCCGACGATAT

MT946551.1 66157 66164 2 194 0 0 GTTATCGGGACACGCTCT

MT946551.1 66623 66623 1 72 0 0 ACATCCGGCAA

MT946551.1 66653 66663 4 392 0 0 TATATCGTCGCCCGGCGAAAA

MT946551.1 66734 66734 1 82 0 0 ATGCCCGCATA

MT946551.1 66986 66992 2 192 0 0 TACAACGTCATCGCTTC

MT946551.1 67144 67150 3 216 0 0 GTATCCGACGACGTTAC

MT946551.1 67208 67216 2 216 0 0 GAAATCGTAGCAGCGTATT

MT946551.1 67471 67475 2 198 0 0 TAAGTCGAACGTGGA

MT946551.1 67655 67655 1 85 0 0 CTCAACGATGT

MT946551.1 67702 67702 1 94 0 0 AGAGGCGGACT

MT946551.1 67822 67822 1 100 0 0 CAAAGCGGGTT

MT946551.1 67908 67908 1 91 0 0 AGACTCGAGTA

MT946551.1 68073 68078 2 190 0 0 CATCACGTACCGGAAC

MT946551.1 68126 68135 3 249 0 0 GTGGTCGACGGATACGGACA

MT946551.1 68158 68158 1 114 0 0 TAATACGCTCA

MT946551.1 68171 68174 2 188 0 0 AAGTACGCCGCCAA

MT946551.1 68405 68405 1 97 0 0 CTGTACGATAT

MT946551.1 68533 68533 1 77 0 0 AGAAACGGCTA

MT946551.1 68705 68708 2 192 0 0 GGTTTCGACGAGTT

MT946551.1 68747 68747 1 83 0 0 AAGACCGAAAT

MT946551.1 68768 68768 1 83 0 0 GTATCCGATGA

MT946551.1 68921 68933 3 297 0 0 ATTACCGAATTAGTCGTCGAATA

MT946551.1 69091 69098 2 138 0 0 TCTTCCGGGTGCCGCCAA

MT946551.1 69275 69300 5 545 0 0 ACATACGATATCTTCGGTATCGAGGCCGCTCGTGAA

MT946551.1 69314 69314 1 112 0 0 TTGTGCGAAGC

MT946551.1 69431 69434 2 198 0 0 AAGTTCGGCGCAGC

MT946551.1 69460 69464 2 202 0 0 AGCTACGTTCGGAGA

MT946551.1 69488 69490 2 110 0 0 TTAAACGCGGCTC

MT946551.1 69860 69860 1 105 0 0 ACTCCCGCAGC

MT946551.1 69880 69891 3 324 0 0 CAACACGGGCTCGTTTCGTTGA

MT946551.1 69916 69925 3 318 0 0 AAAGGCGGTTACGTCGTCAA

MT946551.1 70158 70158 1 104 0 0 TTGAACGTAGT

MT946551.1 70228 70237 3 318 0 0 TTTATCGGTAAACGCGTGTA

MT946551.1 70411 70418 2 176 0 0 AAATACGGTTAACGATTG

MT946551.1 70792 70798 2 192 0 0 AAATGCGGTAACGAATG

MT946551.1 70934 70936 2 162 0 0 GTAAACGCGTACA

MT946551.1 70970 70970 1 78 0 0 GCTGCCGTTCC

MT946551.1 70994 70994 1 95 0 0 AAATTCGGTTT

MT946551.1 71031 71038 2 174 0 0 CAAGTCGAGGATCGTGTT

MT946551.1 71092 71098 2 208 0 0 CATTTCGTTTTCGATTC

MT946551.1 71161 71174 3 339 0 0 ATCTACGATGTTCAGCGCCGTAGT

MT946551.1 71747 71750 2 214 0 0 TTCACCGCCGCCAT

MT946551.1 71806 71806 1 106 0 0 ATTTTCGTCTA

MT946551.1 72022 72022 1 114 0 0 GAAAACGTATA

MT946551.1 72190 72197 2 200 0 0 ATCTTCGTCACACGTTAG

MT946551.1 72355 72360 3 324 0 0 CTCTTCGCCGCGACTT

MT946551.1 72632 72632 1 101 0 0 AAGTCCGCACT

MT946551.1 72977 72985 2 218 0 0 TTAAACGTGGAAACGATAA

MT946551.1 73006 73012 2 158 0 0 AATCACGTCAGCGGCAT

MT946551.1 73169 73174 2 208 0 0 TCACACGGTGCGAATT

MT946551.1 73242 73242 1 91 0 0 TTTTGCGTTTC

MT946551.1 73261 73270 3 210 0 0 AGGCTCGTCTCTCGCGATGA

MT946551.1 73940 73942 2 84 0 0 TCATCCGCGTCAA

MT946551.1 74000 74005 2 190 0 0 ATAGTCGATACGTTAT

MT946551.1 74325 74333 2 202 0 0 TGTATCGTAATGGCGTGGT

MT946551.1 74430 74430 1 88 0 0 AAAAACGAGGA

MT946551.1 74646 74658 3 324 0 0 GAGCTCGATGATAGCGACGGGGA

MT946551.1 75178 75183 2 208 0 0 AGAATCGCAACGCTAA

MT946551.1 75567 75567 1 107 0 0 GTATACGAATG

MT946551.1 75607 75611 2 168 0 0 ATCTCCGAACGTATG

MT946551.1 75852 75861 2 122 0 0 ATAGTCGTAGATCACGTTAA

MT946551.1 76158 76173 3 309 0 0 ACTCACGCATCGAGTATAGCCGAACA

MT946551.1 76215 76215 1 72 0 0 ATTAGCGAATA

MT946551.1 76443 76461 3 276 0 0 TGCAACGTATATAGACGCTTTAGCGAAGA

MT946551.1 76590 76606 3 324 0 0 GGAATCGTTTATTCGATTTACCGTTAC

MT946551.1 76776 76776 1 95 0 0 ACTATCGACAG

MT946551.1 76909 76909 1 68 0 0 GGATCCGGTGC

MT946551.1 77043 77043 1 75 0 0 ATTGACGACTC

MT946551.1 77097 77109 3 279 0 0 TTCTCCGCCTATTAACGCGCCTA

MT946551.1 78075 78083 2 190 0 0 TTTTACGAATAAACGAACT

MT946551.1 78208 78213 2 182 0 0 GGAAACGGTGCGGACC

MT946551.1 78258 78267 3 279 0 0 GGTAGCGACGGATCCGGATG

MT946551.1 78368 78373 2 178 0 0 CTATTCGATCCGATAC

MT946551.1 79150 79150 1 75 0 0 CTTGGCGATTC

MT946551.1 79373 79388 4 340 0 0 AAAGTCGATAACGACGATAACGAAGT

MT946551.1 79508 79511 2 166 0 0 TTATGCGTCGAATT

MT946551.1 79663 79672 2 176 0 0 TTATACGTTTGATTCGTTCA

MT946551.1 79756 79764 2 182 0 0 GTCTCCGTATTATCGTAAT

MT946551.1 79951 79961 2 192 0 0 TAAGGCGTTTGTATTCGCTTG

MT946551.1 79973 79981 3 243 0 0 TTTAACGGCGTTTCGGAAA

MT946551.1 80056 80058 2 126 0 0 TCACACGCGCCAT

MT946551.1 80147 80160 4 344 0 0 TGCTACGAGACGAGACGTCGCCTA

MT946551.1 80265 80269 2 178 0 0 TGTACCGTTCGAATC

MT946551.1 80374 80374 1 60 0 0 ATAGACGGGGT

MT946551.1 80431 80433 2 146 0 0 AGTCACGCGATCT

MT946551.1 80523 80531 2 182 0 0 TTTCTCGAATATACGGGCA

MT946551.1 80779 80785 2 108 0 0 TGTCCCGTCAGCGTGCA

MT946551.1 80831 80834 2 146 0 0 AAATGCGACGAGTT

MT946551.1 80879 80879 1 95 0 0 CTATTCGAAAG

MT946551.1 80942 80954 3 237 0 0 TTAGACGCGTGTCTAGACGAAAT

MT946551.1 81319 81319 1 92 0 0 ACCACCGCATG

MT946551.1 81449 81455 2 182 0 0 TCATTCGAAGACGCTAT

MT946551.1 81554 81554 1 101 0 0 GATTACGTAAC

MT946551.1 81582 81586 2 170 0 0 AAAAACGATCGCATA

MT946551.1 81746 81750 2 176 0 0 TTAACCGAACGAGGA

MT946551.1 81797 81797 1 94 0 0 AACAGCGAAGA

MT946551.1 81877 81885 2 124 0 0 CTGTCCGAGGAAACGAAAG

MT946551.1 82478 82478 1 61 0 0 AAGACCGTGTT

MT946551.1 82566 82566 1 95 0 0 GATAACGCATT

MT946551.1 82587 82594 3 264 0 0 ATTGCCGTCGTGCGATTC

MT946551.1 82650 82656 3 240 0 0 AATGACGCGTACGATAA

MT946551.1 83281 83281 1 89 0 0 ATAATCGCTTT

MT946551.1 83464 83470 2 140 0 0 TATAACGATAACGTCAT

MT946551.1 83485 83495 2 142 0 0 TATAACGGATTATCTCGCTAC

MT946551.1 83734 83738 2 186 0 0 CTTAACGAACGAGGT

MT946551.1 83788 83788 1 89 0 0 TATTACGAAAT

MT946551.1 83827 83831 2 106 0 0 TATCACGGACGTAAG

MT946551.1 83950 83950 1 87 0 0 TATAACGTGTC

MT946551.1 84055 84064 2 194 0 0 AATGGCGTGTTATACGGAGA

MT946551.1 84259 84259 1 67 0 0 ATAAACGGCAA

MT946551.1 84565 84583 3 237 0 0 AATGACGAAGTGACGTCATTAAACGATTA

MT946551.1 84934 84940 2 182 0 0 AAATACGAAAACGATAT

MT946551.1 85015 85015 1 82 0 0 TATAACGTGGT

MT946551.1 85110 85118 2 172 0 0 ACACACGAGCTGACGTTAG

MT946551.1 85210 85218 2 116 0 0 AAATTCGTGAAGACGTGGA

MT946551.1 85233 85236 2 174 0 0 CCTCTCGGCGAAAT

MT946551.1 85266 85271 2 164 0 0 ATAAACGTTCCGTGTG

MT946551.1 85287 85299 3 288 0 0 ACCTACGCGTATTATCACGTTGG

MT946551.1 85524 85533 3 186 0 0 TTTCTCGCGAATATCGTTGA

MT946551.1 85594 85594 1 89 0 0 AATTGCGAATG

MT946551.1 85654 85654 1 76 0 0 CAAATCGGACA

MT946551.1 85681 85685 3 216 0 0 GTTCTCGCGCGCTGG

MT946551.1 86223 86223 1 102 0 0 ATATACGTAGT

MT946551.1 86354 86368 4 308 0 0 TCTAACGGCTTCAATCGCGCGTTAG

MT946551.1 86589 86597 2 140 0 0 AGTGACGGATATCCGTTAA

MT946551.1 86692 86692 1 99 0 0 TATTACGGTTT

MT946551.1 87048 87060 3 234 0 0 ATTAACGACGATAAATACGAATA

MT946551.1 87176 87176 1 74 0 0 ATTTGCGAGGA

MT946551.1 87205 87212 2 156 0 0 ACTCACGGCATTCGTTAA

MT946551.1 87890 87900 2 174 0 0 TATAACGGGATTGTCCGGCAT

MT946551.1 87923 87950 4 436 0 0 GTTACCGTCTACATCGTATACTCGACCATCTACGCCTT

MT946551.1 88097 88101 2 210 0 0 ATTGTCGATCGGTGA

MT946551.1 88276 88276 1 106 0 0 ATCTACGTTCT

MT946551.1 88333 88346 3 255 0 0 TCTGACGAAGAGACGCCTCGTTCC

MT946551.1 88403 88403 1 114 0 0 ACCCTCGCCTC

MT946551.1 88424 88424 1 99 0 0 GAATACGCATG

MT946551.1 88689 88689 1 112 0 0 TCAGCCGCACT

MT946551.1 88806 88825 5 540 0 0 TCTTGCGCGTATTCGCCCGGATCACGATTT

MT946551.1 88850 88860 3 348 0 0 GCTATCGAACGTAAACGTAGT

MT946551.1 88953 88953 1 81 0 0 TTTTTCGACAT

MT946551.1 88964 88969 2 220 0 0 ATTAACGTATCGCATT

MT946551.1 88991 88991 1 85 0 0 CTTAGCGAATG

MT946551.1 89228 89228 1 95 0 0 TAATGCGATGG

MT946551.1 89260 89269 2 166 0 0 CTGAACGAGTAGGACGGATC

MT946551.1 89656 89663 2 212 0 0 TGAGGCGTACTACGTCTG

MT946551.1 89717 89729 3 300 0 0 ATAATCGATATAGGCCGCGTGTG

MT946551.1 89860 89868 2 234 0 0 AGCAACGAGTGAACGTAAT

MT946551.1 89883 89883 1 118 0 0 AGCTTCGTATA

MT946551.1 90084 90090 2 222 0 0 GCTATCGCTTACGCATT

MT946551.1 90159 90159 1 91 0 0 TCTACCGAATA

MT946551.1 90218 90228 3 288 0 0 ATGGTCGAAACACGTCGAAGG

MT946551.1 90330 90355 5 565 0 0 GTGTGCGCTGAGGTCGGCAACTACGGCGTCCGCTTT

MT946551.1 90468 90472 2 198 0 0 CATGTCGGTCGGCAT

MT946551.1 90525 90525 1 88 0 0 AGCTCCGTATT

MT946551.1 90850 90850 1 79 0 0 TTGGCCGTAAT

MT946551.1 90960 90960 1 77 0 0 GGGTCCGTTCT

MT946551.1 91104 91118 3 273 0 0 ATCTCCGTTATAGACGTTACGTTGA

MT946551.1 91230 91230 1 89 0 0 AATGGCGGAAA

MT946551.1 91271 91271 1 84 0 0 TTTTTCGTGTT

MT946551.1 91398 91408 2 178 0 0 AATCTCGGCAGACTCCGGATA

MT946551.1 91513 91513 1 107 0 0 GGATACGAAAT

MT946551.1 91567 91576 2 230 0 0 GAGAGCGATGTTGCCGCATG

MT946551.1 91597 91600 2 206 0 0 ACAGTCGCCGTTGG

MT946551.1 91726 91726 1 106 0 0 TTGAACGTTTC

MT946551.1 91737 91764 5 570 0 0 AAAGTCGAACGAAGAGTCACGAATAACGATATCGGATA

MT946551.1 92016 92023 2 202 0 0 AACACCGTTACACGAAGA

MT946551.1 92070 92076 2 210 0 0 TGGTACGTAACCGAATC

MT946551.1 92193 92193 1 102 0 0 ACCATCGTTTG

MT946551.1 92256 92262 2 232 0 0 ACTATCGACTGCGAAGA

MT946551.1 92414 92423 3 321 0 0 GCTTCCGTAAACGCCGTCTT

MT946551.1 92525 92525 1 102 0 0 AGCTACGTGAG

MT946551.1 92723 92723 1 98 0 0 ACAAGCGAGTG

MT946551.1 92904 92904 1 100 0 0 TTACACGTTTT

MT946551.1 93512 93522 3 288 0 0 CTCTACGTATCTCGTCGTCCA

MT946551.1 93630 93637 2 184 0 0 GTTACCGTCCTCCGCCAA

MT946551.1 93680 93688 2 184 0 0 AACACCGCTTAGGCGGATT

MT946551.1 93714 93714 1 99 0 0 TTTTTCGTACC

MT946551.1 93857 93860 2 166 0 0 AAGATCGTCGTCAA

MT946551.1 93920 93923 2 172 0 0 TGAACCGTCGCCAT

MT946551.1 93994 93994 1 86 0 0 GAGCTCGCATC

MT946551.1 94133 94133 1 70 0 0 GTCTCCGAGTT

MT946551.1 94235 94235 1 85 0 0 AAAGCCGGTGT

MT946551.1 94273 94276 2 174 0 0 TCTGACGTCGTTCT

MT946551.1 94406 94406 1 104 0 0 AGGAGCGAACA

MT946551.1 94473 94481 2 116 0 0 GAAATCGGACTGTCGGATG

MT946551.1 94926 94938 4 428 0 0 ACATACGCGTTCTCGAGCGCAGG

MT946551.1 95065 95073 2 164 0 0 GGAATCGTGTAGTCGACAG

MT946551.1 95246 95246 1 104 0 0 TAGCTCGTTTC

MT946551.1 95376 95376 1 103 0 0 AATCTCGTACT

MT946551.1 95633 95633 1 100 0 0 TCCTGCGTTAG

MT946551.1 95699 95705 2 186 0 0 TTACTCGAAAGCGTGAT

MT946551.1 95811 95815 2 194 0 0 ATATACGTTCGTCAC

MT946551.1 96103 96112 3 222 0 0 GATGGCGGTGTCGTCGTCTT

MT946551.1 96163 96172 3 249 0 0 TTGCTCGGCGTCAACGCTGG

MT946551.1 96190 96196 3 270 0 0 GAACTCGCCGGCGCATC

MT946551.1 96598 96607 2 176 0 0 ACAGACGATATTATCGACTA

MT946551.1 96631 96637 2 184 0 0 CTCACCGAATACGAGGA

MT946551.1 97087 97094 2 216 0 0 TTATACGAATATCGTTCT

MT946551.1 97365 97375 3 285 0 0 AACATCGACATTCGACGTCAA

MT946551.1 97732 97737 3 294 0 0 TGATACGCCGCGTTCT

MT946551.1 98007 98007 1 89 0 0 AGTTACGATTT

MT946551.1 98130 98132 2 96 0 0 AGTCTCGCGTTCA

MT946551.1 98262 98265 2 176 0 0 TCTAACGTCGGTAT

MT946551.1 98387 98397 2 168 0 0 AGCTACGGCATCAGACGCAAT

MT946551.1 98423 98433 2 170 0 0 TGTAACGTATTTAGACGACAG

MT946551.1 98496 98498 2 90 0 0 ATTCCCGCGTCAG

MT946551.1 98663 98669 3 258 0 0 TATATCGGACGCGTTCA

MT946551.1 98752 98758 2 172 0 0 TAGTTCGTAAACGAACT

MT946551.1 98957 98960 2 172 0 0 CTCAACGTCGATTT

MT946551.1 99414 99414 1 66 0 0 TTATCCGAGGA

MT946551.1 99438 99441 2 186 0 0 TTAGTCGCCGTCTC

MT946551.1 99538 99538 1 88 0 0 TGATGCGTTCC

MT946551.1 99602 99624 5 400 0 0 CATCACGGTATCGCCGTCGCTAGATAACGCTAA

MT946551.1 99732 99732 1 78 0 0 AACAACGACTT

MT946551.1 99805 99805 1 95 0 0 TGGTACGAAAT

MT946551.1 99941 99945 2 204 0 0 TGTATCGCACGCTCT

MT946551.1 100225 100225 1 86 0 0 GACTTCGTAAA

MT946551.1 100511 100528 3 249 0 0 TATCCCGTAGTAGACGTCTATTCGCCCA

MT946551.1 100678 100683 2 186 0 0 ATTATCGTGGCGCCTT

MT946551.1 100898 100898 1 92 0 0 CTATTCGAGAT

MT946551.1 101234 101236 2 108 0 0 GCAAACGCGATAA

MT946551.1 101292 101302 2 194 0 0 ATAGACGACTCATTTCGTTTA

MT946551.1 101729 101737 3 267 0 0 GCATCCGCTTCGTCGAATA

MT946551.1 101754 101754 1 92 0 0 AGCATCGCTTT

MT946551.1 102122 102129 2 200 0 0 GGATACGGTACACGTACC

MT946551.1 102310 102310 1 68 0 0 TACCACGAGTA

MT946551.1 103025 103025 1 85 0 0 AAGACCGAATA

MT946551.1 103088 103088 1 89 0 0 TTCCTCGTAAT

MT946551.1 103259 103261 2 86 0 0 AGGATCGCGAGTA

MT946551.1 103561 103567 2 214 0 0 CAAAGCGGGGTCGATCT

MT946551.1 103763 103763 1 94 0 0 TGAGTCGGAGC

MT946551.1 103975 103980 2 162 0 0 ATTCACGTGACGTGGT

MT946551.1 104078 104090 3 312 0 0 TATATCGAACGGATGAACGAAGG

MT946551.1 104241 104245 2 204 0 0 AATGACGACCGTACC

MT946551.1 104356 104365 2 172 0 0 ACTCACGTTAACAACGTGGT

MT946551.1 104473 104477 2 188 0 0 ATTATCGAACGAGTA

MT946551.1 104547 104564 3 270 0 0 GATATCGGAACAACGTCAACAACGAGAA

MT946551.1 104998 104998 1 94 0 0 ATTATCGAGCA

MT946551.1 105094 105094 1 90 0 0 GACTGCGATGC

MT946551.1 105169 105176 2 164 0 0 ATATTCGTTAAACGAATT

MT946551.1 105247 105261 3 225 0 0 CCTGTCGTCTCATACCGGACGACAT

MT946551.1 105359 105359 1 47 0 0 CTCTCCGACCA

MT946551.1 105386 105395 2 160 0 0 TTGGCCGTCTCTGACGGTAC

MT946551.1 105453 105462 2 184 0 0 TCCATCGCATTGTACGATGT

MT946551.1 105656 105663 2 192 0 0 TCCACCGATTTGCGCTAG

MT946551.1 105946 105949 2 148 0 0 GATGGCGACGGTAA

MT946551.1 105961 105970 2 190 0 0 TGCTACGCAAATACCGATCA

MT946551.1 106326 106338 3 300 0 0 TCCAGCGATTAGCACGCCGGAAA

MT946551.1 106397 106400 2 196 0 0 TAAATCGCCGAATG

MT946551.1 106532 106537 2 172 0 0 AGCAACGGCTCGTAAT

MT946551.1 106624 106624 1 102 0 0 TTATGCGATGT

MT946551.1 106748 106748 1 82 0 0 CATAACGATGA

MT946551.1 106806 106820 3 279 0 0 ATCAACGAATTATCGTCTACGAACA

MT946551.1 106853 106855 2 160 0 0 TAGTACGCGAATA

MT946551.1 106909 106911 2 188 0 0 ATGGCCGCGCCCA

MT946551.1 106968 106984 4 400 0 0 ATGTACGTCACCAACGGCGGACGTAGT

MT946551.1 107046 107053 2 172 0 0 GTTAACGTTAATCGTGCA

MT946551.1 107359 107375 3 303 0 0 ATAGCCGGTTTCCGTTTTGCACGTAAC

MT946551.1 107564 107571 2 134 0 0 GACATCGTTGCCCGGATG

MT946551.1 107592 107592 1 54 0 0 AGTAGCGTGTT

MT946551.1 107809 107809 1 115 0 0 TAGTACGTATA

MT946551.1 108365 108372 2 200 0 0 CAAACCGAGTAACGTTCT

MT946551.1 108513 108513 1 91 0 0 GCACCCGCAGA

MT946551.1 108652 108667 3 318 0 0 TTTAACGAAGACGGGGATTTCGTAGA

MT946551.1 108830 108838 2 198 0 0 TTCAACGCAAAGTCGTATC

MT946551.1 108874 108884 2 208 0 0 ATGATCGAATCAAAACGTCCT

MT946551.1 108939 108939 1 90 0 0 TACCACGTGTT

MT946551.1 108973 108982 2 140 0 0 AAAACCGTCATTTGCGTACC

MT946551.1 109565 109565 1 83 0 0 AACTACGTGAA

MT946551.1 109609 109609 1 82 0 0 TTTTTCGGATC

MT946551.1 109900 109907 2 170 0 0 AATTTCGTTCAACGAATT

MT946551.1 110040 110048 2 214 0 0 TAACTCGCAAAATCGTTAA

MT946551.1 110064 110081 4 424 0 0 TTAAGCGACGATCCGCATGCTGCGCAGG

MT946551.1 110243 110248 2 188 0 0 CATTCCGGAACGCACA

MT946551.1 110307 110311 2 182 0 0 TAGCACGATCGTGCA

MT946551.1 110522 110539 5 490 0 0 GTTTACGTATGCGGCTCGTTCGCGTCTC

MT946551.1 110757 110757 1 100 0 0 ATAGGCGTGCA

MT946551.1 110837 110837 1 72 0 0 TGATCCGGGCT

MT946551.1 110950 110957 2 212 0 0 ATATGCGAAGTGCGTGTA

MT946551.1 110979 110985 2 176 0 0 GACAGCGATAACGGAAT

MT946551.1 111462 111462 1 82 0 0 GGAAACGGAAC

MT946551.1 111603 111612 2 216 0 0 GAGTGCGCAACTGTCGATGA

MT946551.1 111867 111873 2 168 0 0 AATATCGTCACCGATGT

MT946551.1 111894 111894 1 112 0 0 TTATTCGAGTG

MT946551.1 112089 112096 3 321 0 0 AAGGTCGCCGTACGTCAA

MT946551.1 112159 112164 2 202 0 0 ATTTGCGTCTCGCCTG

MT946551.1 112209 112213 2 228 0 0 GAGATCGGTCGAAGC

MT946551.1 112243 112271 5 565 0 0 ACATTCGGATTGCGAGACTCCGTTCCGGATAGACGCAAA

MT946551.1 112282 112290 2 206 0 0 TTAGACGATGTAGCGGATA

MT946551.1 112397 112406 2 212 0 0 AATAACGTTAGTAACGCCAT

MT946551.1 112648 112664 3 261 0 0 GAATACGTATCCGGTATTTAACGATTC

MT946551.1 112981 112983 2 146 0 0 AGTAACGCGTGGA

MT946551.1 113213 113227 3 324 0 0 AACCACGTACTCACGGCTACGGCAT

MT946551.1 113521 113535 3 300 0 0 AGAAACGGAATCGGTAGATCGTCTA

MT946551.1 113712 113719 2 164 0 0 TTGACCGTCAAGCGGCCA

MT946551.1 113811 113813 2 96 0 0 ATAATCGCGAGTA

MT946551.1 114131 114141 2 180 0 0 TTTCTCGTTTTCCTCCGTATC

MT946551.1 114467 114474 2 200 0 0 GTTAACGATCAACGAGTT

MT946551.1 114631 114645 3 330 0 0 GTCATCGCATCCTAACGTACGGCAA

MT946551.1 114703 114708 2 186 0 0 ACATCCGAAACGATAT

MT946551.1 114828 114838 2 214 0 0 TCAGACGGTAGTTATCGAACA

MT946551.1 114977 114986 3 201 0 0 TTCATCGGACGTTCCGGAAA

MT946551.1 115219 115228 2 208 0 0 TTAGACGTAGAAAACGGATG

MT946551.1 115430 115440 2 210 0 0 GTTTCCGCATGTCATCGAAAT

MT946551.1 115947 115947 1 94 0 0 TCTAACGCCTA

MT946551.1 116004 116020 3 246 0 0 GAATCCGGAGACGCTATAGCACGAAAT

MT946551.1 116088 116088 1 80 0 0 GTTTCCGAAAA

MT946551.1 116116 116125 3 267 0 0 AATCTCGCGTTGAACGAGTA

MT946551.1 116238 116258 5 470 0 0 ACAGTCGCGTATATCGCGGATGAAACGGAAC

MT946551.1 116271 116271 1 92 0 0 CCATACGATGA

MT946551.1 116492 116492 1 69 0 0 AAATCCGAAAT

MT946551.1 116692 116698 2 170 0 0 AGGGACGAAAACGTGGA

MT946551.1 116921 116925 2 194 0 0 TACCACGCACGTATC

MT946551.1 116955 116964 2 200 0 0 ATGAACGCCAGAGGCGTAAA

MT946551.1 116985 116989 2 232 0 0 GATTTCGAACGAAGG

MT946551.1 117251 117261 2 196 0 0 TGGTTCGACATCCACCGATGG

MT946551.1 117282 117303 4 396 0 0 CTAATCGGTTCGGTAACGTCTGTGGACGATGG

MT946551.1 117445 117451 2 190 0 0 GATACCGATCACGTTCT

MT946551.1 117521 117525 2 168 0 0 CTTCACGTTCGCATG

MT946551.1 117540 117540 1 90 0 0 ACCTCCGCAGT

MT946551.1 117551 117563 3 354 0 0 TTTTACGAGCGATTTCACGTTCA

MT946551.1 117596 117609 3 270 0 0 GAATTCGCTTATCGTTATCGGGTT

MT946551.1 117913 117921 2 226 0 0 AAAACCGGCATTACGATCA

MT946551.1 117952 117958 2 132 0 0 TGATCCGTTTACGTTAA

MT946551.1 118129 118129 1 62 0 0 ACCATCGTGAT

MT946551.1 118208 118208 1 106 0 0 TTTAACGTTTT

MT946551.1 118420 118420 1 112 0 0 TGCTTCGATTT

MT946551.1 118493 118493 1 79 0 0 TGAACCGTTGG

MT946551.1 118529 118529 1 69 0 0 ATGTTCGCCAT

MT946551.1 118691 118696 2 190 0 0 TTCATCGTTGCGTTTA

MT946551.1 118820 118822 2 180 0 0 TGCTTCGCGTTTA

MT946551.1 118886 118895 2 162 0 0 AAGATCGTCATCTCCGGGGA

MT946551.1 120233 120245 3 285 0 0 GGGTTCGAGTTCAACGACGATTG

MT946551.1 120262 120264 2 164 0 0 CTTCCCGCGGATG

MT946551.1 120319 120319 1 92 0 0 TTTTTCGACTT

MT946551.1 120391 120405 4 428 0 0 GTAGACGCGTATTGCGCATCGATAT

MT946551.1 120611 120617 3 258 0 0 AAAGTCGACGGCGGACA

MT946551.1 120920 120927 2 182 0 0 GGGATCGTCTATCGATAG

MT946551.1 121118 121136 4 368 0 0 TATAACGTCCTTATCGGCGGTATCGGTAC

MT946551.1 121169 121178 2 186 0 0 AAATACGGAGTCTTCGATAA

MT946551.1 121249 121249 1 79 0 0 CCTTTCGTTTC

MT946551.1 121352 121352 1 86 0 0 TGGTACGTGTC

MT946551.1 121922 121925 2 162 0 0 GAAAACGACGAAGA

MT946551.1 121949 121949 1 88 0 0 TTCTCCGCTAC

MT946551.1 121989 121997 3 234 0 0 ATAAACGCAAACGCGTGAT

MT946551.1 122076 122076 1 100 0 0 TAGTGCGCCTA

MT946551.1 122120 122132 3 303 0 0 ACTGACGCCGCTGTTGCCGTTGC

MT946551.1 122179 122179 1 93 0 0 CACTACGCAAT

MT946551.1 122263 122263 1 57 0 0 ATTCTCGGATG

MT946551.1 122287 122287 1 79 0 0 CACTGCGGAAT

MT946551.1 122532 122542 3 264 0 0 GTTGTCGGTGCCGGTCGCTAT

MT946551.1 122697 122697 1 105 0 0 TAATGCGGTTT

MT946551.1 122889 122892 2 166 0 0 CAGTACGACGGATG

MT946551.1 123019 123046 7 798 0 0 TAAGTCGTTAACGTACGCCGCCATGGACGCCGCGTTTG

MT946551.1 123174 123174 1 93 0 0 ATGTACGGGAT

MT946551.1 123655 123670 3 312 0 0 TATCACGGTGACCGTAGTTGCGGGAA

MT946551.1 123946 123946 1 106 0 0 TATGGCGCCTA

MT946551.1 124308 124313 2 182 0 0 GCTGCCGTCACGTTGT

MT946551.1 124732 124742 2 210 0 0 AACATCGTTTACAAACGACAT

MT946551.1 124809 124817 2 196 0 0 TTAGACGAGTTACCGACTA

MT946551.1 125694 125694 1 116 0 0 TGCTCCGCATA

MT946551.1 125910 125910 1 119 0 0 ATCCCCGCATG

MT946551.1 126262 126262 1 99 0 0 GCTAACGGTAT

MT946551.1 126351 126351 1 109 0 0 TGGGGCGGTAT

MT946551.1 126450 126450 1 83 0 0 AATAACGGAAA

MT946551.1 126477 126486 2 166 0 0 ATAGACGGTTCAGACGACCC

MT946551.1 126555 126555 1 61 0 0 CACAACGGATG

MT946551.1 126644 126654 3 357 0 0 ATACACGGCGGATAACGTAAT

MT946551.1 126687 126690 2 174 0 0 GCATTCGTCGATAA

MT946551.1 126792 126795 2 226 0 0 CTAAACGACGAAGG

MT946551.1 126833 126836 2 198 0 0 ATGGTCGACGTTTC

MT946551.1 127268 127272 2 228 0 0 ATATTCGTTCGATAC

MT946551.1 127294 127315 5 645 0 0 AAACTCGTAGCTCGCAAGTCGATGCGCGACTA

MT946551.1 127340 127346 2 230 0 0 AACTTCGAAACCGTTAT

MT946551.1 127421 127434 3 342 0 0 TTAATCGAGTACACCGCACGACAA

MT946551.1 127480 127489 2 214 0 0 TGCTGCGTAATATGCGGTCT

MT946551.1 127658 127658 1 111 0 0 CTGATCGAAAA

MT946551.1 127950 127958 3 324 0 0 AATATCGCCGTACCGTTCT

MT946551.1 128085 128094 2 204 0 0 GTTTCCGCACTCATCGTATA

MT946551.1 128121 128127 2 222 0 0 CACATCGTTTACGCAAT

MT946551.1 128326 128326 1 89 0 0 GGTTCCGTAGT

MT946551.1 128356 128372 5 515 0 0 AACTTCGCGTATACGGTATCGCGATTA

MT946551.1 128478 128488 2 210 0 0 GATTTCGGATTCACACGTTGT

MT946551.1 128526 128535 2 230 0 0 TTCTACGTAAACTTCGATTC

MT946551.1 128565 128572 2 242 0 0 AGAATCGCATACCGATTT

MT946551.1 128681 128681 1 118 0 0 GTGTTCGAGTG

MT946551.1 128865 128880 3 351 0 0 AAGTTCGAGGATGCCGCCATCGTTGA

MT946551.1 129063 129063 1 123 0 0 GGGTACGCCCC

MT946551.1 129171 129184 3 336 0 0 GATCACGCTACCCGTGTACGTGCA

MT946551.1 129402 129402 1 95 0 0 TAATCCGCATA

MT946551.1 129434 129445 3 303 0 0 TTAATCGCACGTTAACCGTAGA

MT946551.1 129460 129460 1 97 0 0 CTAGACGATAA

MT946551.1 129659 129666 2 152 0 0 TACATCGGTCCACGTGTA

MT946551.1 130179 130189 3 345 0 0 ATTTCCGTGTTCGTTCGAATG

MT946551.1 130201 130204 2 186 0 0 CACATCGACGTTAA

MT946551.1 130251 130251 1 111 0 0 TGGTACGCACC

MT946551.1 130343 130345 2 220 0 0 TTGCACGCGTACA

MT946551.1 130426 130436 3 336 0 0 CATATCGTACGAACACGATGG

MT946551.1 130559 130578 4 444 0 0 TGTACCGCGTTATCATCGACTGTACGATAC

MT946551.1 130594 130594 1 73 0 0 GTTACCGTGTT

MT946551.1 130702 130702 1 89 0 0 GATTACGGAAT

MT946551.1 130732 130740 2 222 0 0 TTGCTCGGCCATACGTTTA

MT946551.1 130813 130813 1 104 0 0 ATCACCGTGTT

MT946551.1 131063 131063 1 108 0 0 ACTTTCGAGGT

MT946551.1 131164 131164 1 103 0 0 AAATCCGGCAC

MT946551.1 131180 131188 3 318 0 0 GTAACCGCGTACACGGCCA

MT946551.1 131591 131600 2 200 0 0 ATTAACGAGAATGGCGTTTG

MT946551.1 131621 131621 1 92 0 0 TAATGCGTCTA

MT946551.1 131817 131831 4 468 0 0 GGTTGCGCGGTTAATACGCCGGTTA

MT946551.1 132012 132017 2 178 0 0 GACTCCGATTCGGGAC

MT946551.1 132220 132222 2 194 0 0 ATCTACGCGGGCA

MT946551.1 132267 132275 2 178 0 0 GAAGACGATGATTCGTCTA

MT946551.1 132677 132684 2 234 0 0 GAACTCGAATGCCGCCTC

MT946551.1 133266 133275 2 204 0 0 TATACCGGCAAACACGATAA

MT946551.1 133479 133500 5 545 0 0 AGTAGCGTATGCCGCCGCTAAAGGCGCGTCAA

MT946551.1 133594 133600 3 303 0 0 AGAAACGTCGGCGAGGA

MT946551.1 133860 133872 3 324 0 0 GTACTCGGATACGTGTCCGATAT

MT946551.1 133884 133890 2 212 0 0 CATACCGAACTCGCATC

MT946551.1 134091 134091 1 106 0 0 ATTATCGATTG

MT946551.1 134257 134257 1 102 0 0 TGATACGTGAG

MT946551.1 134290 134296 3 300 0 0 ATATCCGCGAACGATAT

MT946551.1 134343 134353 4 252 0 0 AATGACGTCGCTTCGCGAATT

MT946551.1 134386 134386 1 100 0 0 TATCACGCATC

MT946551.1 134476 134485 2 174 0 0 CTTCCCGGATTAGACGATAG

MT946551.1 134688 134698 2 176 0 0 AACTACGTTATCATCCGTAAC

MT946551.1 134811 134833 4 468 0 0 TAAAGCGGGCCCTCGGTACGTACTTAACGCTAT

MT946551.1 134964 134970 2 162 0 0 GTTAGCGGAATCGTGTG

MT946551.1 135165 135172 2 210 0 0 AGCTACGTCTATCGTATT

MT946551.1 135283 135283 1 90 0 0 TTGTTCGTGTT

MT946551.1 135313 135321 2 198 0 0 GGATTCGATATGACGGACA

MT946551.1 135337 135341 2 210 0 0 TTGTACGAACGAAGA

MT946551.1 135652 135664 3 258 0 0 TGTTACGACGATGAATCCGGTAA

MT946551.1 135678 135678 1 84 0 0 GAAGACGGTTA

MT946551.1 136072 136074 2 148 0 0 CTAAACGCGTTAG

MT946551.1 136531 136536 2 202 0 0 TCTATCGATGCGGCAC

MT946551.1 136603 136611 2 182 0 0 CATGACGAGCTTCCGAGTT

MT946551.1 137520 137544 6 504 0 0 TTGCTCGTCGGTATTCGAAATCGCGACTCCGGAAC

MT946551.1 137647 137655 2 166 0 0 ACAGACGAGACTCCGGAAC

MT946551.1 137901 137905 2 160 0 0 ATTGTCGGCCGTGGC

MT946551.1 137945 137951 2 178 0 0 ATAAACGTTCACGTAAA

MT946551.1 138030 138030 1 99 0 0 TTGAGCGGGCC

MT946551.1 138187 138190 2 198 0 0 ATAGCCGCCGGAAC

MT946551.1 138464 138473 2 200 0 0 GAAGCCGGTCTATTCGACAC

MT946551.1 138668 138668 1 86 0 0 TACCACGTAAA

MT946551.1 139168 139168 1 96 0 0 TAAAGCGAGTA

MT946551.1 139223 139223 1 74 0 0 GATTACGGATT

MT946551.1 139716 139750 6 648 0 0 CATGGCGATGTTTTACGCACACGCTCTCGGTGGGTACGACGAGAA

MT946551.1 140148 140157 2 198 0 0 ATCATCGATTACCACGTCTG

MT946551.1 140243 140252 2 174 0 0 GGGTGCGGTGCTTCCGATCC

MT946551.1 140318 140330 3 321 0 0 TATATCGGCGGCACCATCGAGGT

MT946551.1 140464 140464 1 85 0 0 GCATACGCATT

MT946551.1 140557 140557 1 82 0 0 CTTAGCGAACT

MT946551.1 140744 140744 1 92 0 0 GAACACGTATA

MT946551.1 140934 140939 2 206 0 0 GTACACGTGTCGATTC

MT946551.1 140979 140992 3 348 0 0 TACCTCGACATACGTGTGCGCTAT

MT946551.1 141437 141449 3 348 0 0 AATCTCGTTCGAACATACGAGAA

MT946551.1 141935 141939 2 210 0 0 AGATACGTACGGAAA

MT946551.1 141972 141972 1 92 0 0 AAACACGATAT

MT946551.1 142127 142129 2 220 0 0 TAATGCGCGTAAT

MT946551.1 142349 142358 2 230 0 0 TAATGCGTATAATACGTTGG

MT946551.1 142427 142437 2 152 0 0 TATTTCGGAAGCAGTCGCAAA

MT946551.1 142577 142587 2 192 0 0 TACTGCGTGTATGACCGATTA

MT946551.1 142785 142807 5 575 0 0 AGTCTCGACGTATACGGAAGTCGTGTACGTAAT

MT946551.1 142917 142923 2 194 0 0 TATAACGATAACGAACT

MT946551.1 143048 143073 4 436 0 0 GAAAACGATTTCCGTTGTTACGTTGTTATGCGTACT

MT946551.1 143135 143150 3 306 0 0 ATTAACGTCTACCGAAACATCGTTTA

MT946551.1 143365 143370 2 190 0 0 ATTTTCGTTGCGAAGA

MT946551.1 143411 143411 1 105 0 0 TGTTACGTGTC

MT946551.1 143423 143423 1 79 0 0 TAATGCGGAAT

MT946551.1 143451 143456 2 182 0 0 GAACACGGATCGTGTC

MT946551.1 143540 143540 1 119 0 0 TGCTTCGTACA

MT946551.1 143755 143763 2 222 0 0 GTGTACGATCTAACGAAAA

MT946551.1 143822 143829 2 208 0 0 ACTCTCGAAAGACGTTGT

MT946551.1 144121 144127 2 222 0 0 ATCTACGATGCCGTTAG

MT946551.1 144229 144244 4 444 0 0 AGATGCGCCGTTATACCGTTCGATGA

MT946551.1 144329 144331 2 170 0 0 ATGAACGCGATGT

MT946551.1 144346 144355 2 194 0 0 GTAAGCGTTGGCACCGGAAT

MT946551.1 144472 144484 3 321 0 0 AGAAGCGCTTTTAACGACGATGG

MT946551.1 144555 144559 2 212 0 0 TGGCACGATCGCTGG

MT946551.1 144940 144940 1 90 0 0 TCACACGTGTA

MT946551.1 145433 145442 2 168 0 0 ACATACGACATATCCGCATT

MT946551.1 145493 145493 1 112 0 0 CAAGGCGATTA

MT946551.1 145519 145528 2 218 0 0 TTGTACGGGACCACCGACTG

MT946551.1 145685 145702 3 327 0 0 GATATCGATGACTACGATTGCACGTCTA

MT946551.1 145844 145844 1 96 0 0 ACATGCGCAAT

MT946551.1 146310 146319 2 184 0 0 GTGTCCGCTGTTTCCGTTAA

MT946551.1 146337 146346 2 150 0 0 ATTTTCGTAACTGGCGGATT

MT946551.1 146375 146379 2 180 0 0 TAGCACGATCGTGGT

MT946551.1 146463 146474 3 279 0 0 TATCACGGCATCGACTCGACAT

MT946551.1 146511 146517 2 178 0 0 CTATCCGTTACCGAACA

MT946551.1 146700 146700 1 66 0 0 CTCCCCGCTAT

MT946551.1 147446 147455 2 190 0 0 GTCTTCGAAGCATGCGTCAC

MT946551.1 147472 147472 1 70 0 0 AAACACGTTAG

MT946551.1 147734 147734 1 91 0 0 TGGTTCGGTGG

MT946551.1 147924 147924 1 96 0 0 GACTACGTAGA

MT946551.1 147981 147981 1 53 0 0 TGTTACGGATA

MT946551.1 148015 148034 4 312 0 0 TCTATCGCGCTACACGACCAATATCGATTA

MT946551.1 148435 148442 2 184 0 0 GTTGACGCCATTCGAAAA

MT946551.1 148520 148523 2 182 0 0 ATGTACGGCGAGCT

MT946551.1 148559 148559 1 70 0 0 TCATTCGGTAA

MT946551.1 148705 148712 2 164 0 0 AGCTACGTTTATCGATGT

MT946551.1 149096 149096 1 79 0 0 CAGGTCGTTAT

MT946551.1 149128 149128 1 59 0 0 GATGACGGCCA

MT946551.1 149147 149147 1 70 0 0 ACCCACGTCTT

MT946551.1 149247 149247 1 88 0 0 CATACCGCAAC

MT946551.1 149305 149305 1 58 0 0 TGATGCGGAAT

MT946551.1 149321 149330 3 252 0 0 TTCATCGACATTCGCGAGCT

MT946551.1 149385 149385 1 64 0 0 TCATCCGTTGT

MT946551.1 149486 149486 1 95 0 0 CTGAACGCATT

MT946551.1 149704 149706 2 142 0 0 TTTAACGCGCCTG

MT946551.1 149840 149847 2 140 0 0 AAAAACGAGGAGCGGATA

MT946551.1 149943 149947 2 166 0 0 TACCACGAACGAAAC

MT946551.1 150097 150103 2 152 0 0 AGTAACGTAAACGCAGA

MT946551.1 150121 150131 3 273 0 0 TGGAGCGGGCATCGACGCCTT

MT946551.1 150448 150454 2 118 0 0 GGGGACGGAGACGGTAG

MT946551.1 150555 150570 3 249 0 0 TACAACGTTTACGTGTATGGCGTTTA

MT946551.1 151136 151142 2 142 0 0 TTGCCCGAAAGCGGTGC

MT946551.1 151157 151166 2 126 0 0 ATCTGCGTTATATCCGTTCT

MT946551.1 151217 151223 2 132 0 0 TTGTTCGGCAACGTTAG

MT946551.1 151240 151240 1 61 0 0 CTTGACGAATG

MT946551.1 151268 151270 2 118 0 0 TGATCCGCGCATC

MT946551.1 151297 151297 1 73 0 0 TTTAACGCCCT

MT946551.1 151511 151525 5 390 0 0 TCTAACGCGCATCGTCGTACGTTTA

MT946551.1 151609 151609 1 64 0 0 ACTATCGTTAT

MT946551.1 151697 151697 1 47 0 0 CAATCCGTGTT

MT946551.1 151863 151865 2 114 0 0 TCAACCGCGAATC

MT946551.1 152306 152306 1 61 0 0 GGAATCGATCC

MT946551.1 152363 152369 2 158 0 0 TGTCTCGCACACGTTTA

MT946551.1 152398 152404 2 116 0 0 AAAACCGATAACGATAA

MT946551.1 152462 152464 2 118 0 0 CTAAACGCGTTAG

MT946551.1 152697 152714 4 320 0 0 ATGAGCGTCGTATAATCGTATTCGAGTT

MT946551.1 152739 152752 3 186 0 0 CTACACGTCCGGCAGATTCGATAA

MT946551.1 152825 152825 1 77 0 0 TTACACGTAGC

MT946551.1 152893 152900 2 106 0 0 AAATGCGTTAACCGACAA

MT946551.1 153119 153129 2 118 0 0 AGACACGTTAGGAATCGCAAT

MT946551.1 153142 153152 3 237 0 0 TAAAACGTATCGATACGCATT

MT946551.1 153172 153172 1 53 0 0 CATGTCGTTTG

MT946551.1 153329 153329 1 67 0 0 AATAACGATAA

MT946551.1 153688 153688 1 60 0 0 AGTTACGCCAT

MT946551.1 154012 154012 1 69 0 0 TGTACCGTAAC

MT946551.1 154288 154303 4 304 0 0 TGTTACGTTCATTACGACGACGTTAG

MT946551.1 154330 154342 3 216 0 0 ACTTACGGTTATACCGTCGCAAG

MT946551.1 154385 154390 2 146 0 0 ATCAACGTAACGATAG

MT946551.1 154532 154546 3 267 0 0 AGAGGCGGTATCACCGAGGCGACCT

MT946551.1 154729 154731 2 74 0 0 TATAACGCGACTA

MT946551.1 155113 155113 1 74 0 0 TGATACGATAC

MT946551.1 155135 155145 2 134 0 0 TCTAGCGAGAGATGTCGTTAG

MT946551.1 155192 155192 1 60 0 0 TTATACGTTAA

MT946551.1 155461 155461 1 58 0 0 GAAAACGTCAT

MT946551.1 155562 155562 1 81 0 0 AACTACGTATC

MT946551.1 155730 155739 3 189 0 0 AATACCGTGGCCGACGTCAG

MT946551.1 155802 155802 1 69 0 0 GAAAGCGCAGG

MT946551.1 155916 155929 3 216 0 0 AAAACCGCCGAAGAACTTCGTGCA

MT946551.1 156016 156016 1 63 0 0 TGCTGCGAAAG

MT946551.1 156030 156036 2 124 0 0 CTGAACGAGAACGACTA

MT946551.1 156298 156298 1 58 0 0 AAATGCGATAC

MT946551.1 156335 156343 3 177 0 0 TCTCTCGCGGAGTCGAGAG

MT946551.1 156364 156364 1 61 0 0 AACAACGAAGG

MT946551.1 156406 156406 1 54 0 0 AGATACGTAAA

MT946551.1 156471 156471 1 58 0 0 CAAGTCGAATA

MT946551.1 156524 156534 3 147 0 0 ATAAACGGATACGTCCGTCCA

MT946551.1 156586 156603 4 256 0 0 ATATACGTAACGACGCCTAATCCGAGAC

MT946551.1 156652 156665 3 204 0 0 AGCACCGGTTACGTATTTCGTACC

MT946551.1 156844 156844 1 45 0 0 GGACACGTGTA

MT946551.1 156857 156860 2 118 0 0 GTATACGACGTCAG

MT946551.1 156935 156935 1 50 0 0 GAAATCGTTAA

MT946551.1 156971 156981 3 171 0 0 CTTGTCGACGTAATGCGTAGT

MT946551.1 156994 156998 2 128 0 0 TACTTCGTACGATTC

MT946551.1 157103 157106 2 128 0 0 CATTACGGCGATAT

MT946551.1 157150 157153 2 76 0 0 TAAGACGACGGACA

MT946551.1 157181 157196 4 196 0 0 AATCTCGTCGATGTAAACGTCGTAAG

MT946551.1 157225 157227 2 84 0 0 GGACACGCGGCTG

MT946551.1 157300 157300 1 56 0 0 AATTGCGGGTA

MT946551.1 157366 157366 1 61 0 0 ATGATCGATAA

MT946551.1 157385 157391 3 189 0 0 TATTACGCGGTCGATGT

MT946551.1 157438 157451 3 165 0 0 TGGAGCGAGTGTCACGCTCGATAA

MT946551.1 157653 157660 3 129 0 0 GATATCGCCGGTCGTCAT

MT946551.1 157753 157762 2 116 0 0 TTAAACGAAGCAAACGCCAC

MT946551.1 157908 157908 1 33 0 0 AAACACGGAGT

MT946551.1 158012 158023 4 168 0 0 ATGGACGCATCGTTCGCGACAG

MT946551.1 158059 158062 2 72 0 0 ATGGACGACGGTAA

MT946551.1 158098 158116 5 215 0 0 AATGTCGACGCGATCGTGTAACACGACTC

MT946551.1 158198 158213 3 129 0 0 GGATACGGAGTATCCGGAGACGTCAT

MT946551.1 158227 158237 2 76 0 0 TTCTCCGTGTGGTCTCGGAAC

MT946551.1 158255 158264 2 76 0 0 CACACCGTCTCTTCCGCAGA

MT946551.1 158276 158282 2 72 0 0 AAATGCGAACCCGTACC

MT946551.1 158309 158309 1 49 0 0 TATATCGATGT

MT946551.1 158339 158378 6 288 0 0 GTTAACGACACATCGTGTACTCGGACGACCACTACCGGTCTCAGCGAATC

MT946551.1 158392 158395 2 86 0 0 CTCAACGTCGGAAC

MT946551.1 158429 158435 2 70 0 0 GACTGCGATCCCGTCTT

MT946551.1 158543 158543 1 26 0 0 GTTAACGAAAG

MT946551.1 158570 158576 2 70 0 0 CATGCCGCATTCGGAGA

MT946551.1 158686 158695 2 54 0 0 ATGCCCGGTAGTTGCGATAT

MT946551.1 158922 158928 2 68 0 0 CTCATCGTGTACGGAAG

MT946551.1 158997 159001 2 58 0 0 AACACCGACCGATGA

MT946551.1 159022 159027 2 68 0 0 CAATCCGTAACGGAAA

MT946551.1 159043 159043 1 19 0 0 GAGTCCGAGTC

MT946551.1 159225 159225 1 29 0 0 TGTAGCGAAGA

MT946551.1 159267 159267 1 28 0 0 GTACTCGGGTC

MT946551.1 159312 159327 4 128 0 0 ATCATCGGTTCAACGATCGTCGATAC

MT946551.1 159360 159369 3 114 0 0 TTTAGCGTTCGTATCGGAGA

MT946551.1 159414 159422 3 99 0 0 TATGTCGACGGATCGGCAT

MT946551.1 159533 159537 2 84 0 0 TTAATCGAACGATTC

MT946551.1 159612 159614 2 42 0 0 CCTCTCGCGACTT

MT946551.1 159648 159648 1 38 0 0 TGATACGATTA
