# Supplementary material for: Nanopore sequencing and de novo assembly of a misidentified Camelpox vaccine reveals putative epigenetic modifications and alternate protein signal peptides
Source: Sci Rep. 2021 Sep 7;11:17758. doi: 10.1038/s41598-021-97158-x (PMC8423768; doi:10.1038/s41598-021-97158-x)
Supplement: Supplementary file 7 — Supplementary Information 7. [file 41598_2021_97158_MOESM7_ESM.docx]

**Nanopore sequencing and *de novo* assembly of a misidentified Camelpox vaccine reveals putative epigenetic modifications and alternate protein signal peptides**

**Zack Saud^1^*, Matthew D. Hitchings^2^, Tariq M. Butt^1^**

*^1^ Department of Biosciences, College of Science, Swansea University, Singleton Park, Swansea, SA2 8PP, Wales, United Kingdom*

*^2^ Swansea University Medical School, Swansea University, Singleton Park, Swansea, Sa2 8PP, Wales, United Kingdom*

*** Corresponding author

* Z. Saud: [zack.saud@swansea.ac.uk](mailto:zack.saud@swansea.ac.uk)

**Supplementary Information 7- Megalodon 30 highest scoring reads, none contained a positive value that is indicative of an epigenetic modifications.**

| score | score_pos | score_mod | score_read |
| --- | --- | --- | --- |
| -0.011080121 | 43586 | 1 | 573 |
| -0.011976537 | 238204 | 1 | 677 |
| -0.01211965 | 246091 | 1 | 603 |
| -0.012228882 | 115665 | 1 | 314 |
| -0.013419646 | 296384 | 1 | 162 |
| -0.013998209 | 184828 | 1 | 229 |
| -0.014424055 | 30584 | 1 | 555 |
| -0.014570912 | 37629 | 1 | 246 |
| -0.014872344 | 189663 | 1 | 506 |
| -0.01492361 | 46857 | 1 | 448 |
| -0.015184481 | 24578 | 1 | 421 |
| -0.015237581 | 269622 | 1 | 438 |
| -0.015453149 | 47484 | 1 | 661 |
| -0.015562869 | 229495 | 1 | 176 |
| -0.015562869 | 122810 | 1 | 637 |
| -0.015729929 | 87836 | 1 | 6 |
| -0.015842985 | 99035 | 1 | 679 |
| -0.016131679 | 124131 | 1 | 172 |
| -0.016131679 | 259376 | 1 | 451 |
| -0.016190478 | 85873 | 1 | 338 |
| -0.016674072 | 27170 | 1 | 379 |
| -0.016674072 | 100326 | 1 | 683 |
| -0.016798748 | 95082 | 1 | 243 |
| -0.017052807 | 27797 | 1 | 332 |
| -0.017052807 | 249407 | 1 | 452 |
| -0.01724757 | 235102 | 1 | 666 |
| -0.01737947 | 79054 | 1 | 351 |
| -0.017446047 | 71910 | 1 | 683 |
| -0.017513048 | 309204 | 1 | 153 |
| -0.017513048 | 252524 | 1 | 451 |
